# Association between cannabis use and brain structure and function: an observational and Mendelian randomisation study

**DOI:** 10.1136/bmjment-2024-301065

**Published:** 2024-10-30

**Authors:** Saba Ishrat, Daniel F Levey, Joel Gelernter, Klaus Ebmeier, Anya Topiwala

**Affiliations:** 1 Department of Psychiatry, University of Oxford, Warneford Hospital, Oxford, UK; 2 Nuffield Department of Population Health, Big Data Institute, University of Oxford, Oxford, UK; 3 Division of Human Genetics, Department of Psychiatry, Yale University School of Medicine, New Haven, CT, USA; 4 Department of Psychiatry, Veterans Affairs Connecticut Healthcare Center, West Haven, CT, USA; 5 Wellcome Centre for Integrative Neuroimaging (WIN), Department of Psychiatry, University of Oxford, Warneford Hospital, Oxford, UK

**Keywords:** Cross-Sectional Studies, Substance misuse

## Abstract

**Background:**

Cannabis use during adolescence and young adulthood has been associated with brain harm, yet despite a rapid increase in cannabis use among older adults in the past decade, the impact on brain health in this population remains understudied.

**Objective:**

To explore observational and genetic associations between cannabis use and brain structure and function.

**Methods:**

We examined 3641 lifetime cannabis users (mean (SD) age 61.0 (7.1) years) and 12 255 controls (mean (SD) age 64.5 (7.5) years) from UK Biobank. Brain structure and functional connectivity were measured using multiple imaging-derived phenotypes. Associations with cannabis use were assessed using multiple linear regression controlling for potential confounds. Bidirectional two-sample Mendelian randomisation analyses were used to investigate potential causal relationships.

**Findings:**

Cannabis use was associated with multiple measures of brain structure and function. Participants with a history of cannabis use had poorer white matter integrity, as assessed by lower fractional anisotropy and higher mean diffusivity in the genu of the corpus callosum, as well as weaker resting-state functional connectivity in brain regions underlying the default mode and central executive networks. Mendelian randomisation analyses found no support for causal relationships underlying associations between cannabis use and brain structure or function.

**Conclusions:**

Associations between lifetime cannabis use and brain structure and function in later life are probably not causal in nature and might represent residual confounding.

**Clinical implications:**

Cannabis use is associated with differences in brain structure and function. Further research is needed to understand the mechanisms underlying these associations, which do not appear to be causal.

**WHAT IS ALREADY KNOWN ON THIS TOPIC**

- Cannabis use has been associated with brain structure and functional connectivity in adolescents and young adults.

**WHAT THIS STUDY ADDS**

- This study explores the impact of cannabis use in mid- to old-age adults, expanding our understanding beyond the previously established associations in younger samples.

**HOW THIS STUDY MIGHT AFFECT RESEARCH, PRACTICE OR POLICY**

- These findings shed light on the role of cannabis in brain health, providing potentially useful information for future public health initiatives.

## Background

 In the past decade cannabis use has increased worldwide following its legalisation for medical and recreational purposes. This legalisation has occurred without a comprehensive understanding of the potential effect of cannabis on the brain. Between 2006 and 2013, there was a 250% increase in reported past-year cannabis use among adults aged 65 and older in the United States.^1^ While cannabis use has increased in older adults, studies on health-related outcomes in this group are still limited. There are reports of adverse cannabis effects on neurocognitive performance, brain structure and function.^2^ Whether there is a safe threshold of cannabis use is unknown.

Endogenous cannabinoids, lipid-based retrograde neurotransmitters that bind to cannabinoid receptors, play a crucial role in various brain functions, such as cognition, memory, reward processing, mood regulation and stress sensitivity.^3^ One way in which tetrahydrocannabinol (THC), the primary psychoactive compound found in cannabis, influences the brain’s resting-state functional connectivity is by interacting with cannabinoid receptor type 1 (CB1). This interaction can disrupt the signalling of these naturally occurring endogenous cannabinoids, potentially affecting various brain functions.^4^ These acute effects of THC on the brain might be associated with chronic changes that can be detected in past cannabis users. Such effects are likely to be greater with the increased concentrations of THC found in cannabis sold after the legalisation of cannabis in different parts of the world.^5^ Other cannabis harms relate to smoking, the usual method of administration, and might be attributable to psychoactive substances other than THC in cannabis.

Past use of cannabis has been linked with multiple aspects of brain structure and function in adult and adolescent populations.^2^ The most consistent brain regions linked to cannabis use are the subcortical regions. Smaller hippocampal grey matter volume with cannabis use has been observed with long-term heavy cannabis use^6^ as well as with recent cannabis use.^7^ Diffusion tensor imaging (DTI) studies have also reported associations with white matter microstructure in the corpus callosum inferred by a lower fractional anisotropy (FA) and a higher mean diffusivity (MD).^8^ Functional MRI studies have observed differences in functional connectivity in regions underlying the default mode network and central executive network.^9^ Whether similar brain aspects are affected in mid- to late-life adults is unclear, as only a few studies have included these age groups,^10 11^ and the focus has been on heavy or dependent users who appear to show abnormalities in structural and functional connectivity in different brain regions.^2 6^ Further, most studies have limited their analysis to a narrow range of brain regions and networks.

However, these observational studies have been unable to distinguish between causal or confounded relationships. Mendelian randomisation (MR) is a quasi-experimental method that uses genetic data to investigate potential causality, mitigating certain limitations associated with traditional epidemiological approaches. The genetic variants used as instrumental variables in MR are randomly allocated to offspring and do not change thereafter, allowing for estimation of the causal effect without the influence of confounding factors or reverse causation.

## Objective

Here, we investigate associations between cannabis use and a rich set of measures of structure and function across the brain in a large cohort of older adults. We employ both hypothesis-driven and agnostic approaches and triangulate our observational findings with MR.^12^

## Methods

### Study sample

The UK Biobank is a prospective cohort study involving approximately 500 000 participants from the UK. These individuals were aged 40 to 69 years at the time of their baseline assessment visit, conducted between 2006 and 2010. Participants provided informed consent via electronic signature at the time of recruitment. The ethical approval for UK Biobank has been granted by the National Information Governance Board for Health and Social Care and the NHS North-West Multi-centre Research Ethics Committee.

The study comprised participants from the UK Biobank listed in the supplementary material (online supplemental figures 1 and 2). The phenotypic data used in this study were from the first repeat assessment visit (2012–2013), and the first imaging visit (2014–2019). Self-reported cannabis use data (described below) from the assessment visit were available for 157 316 participants. MRI data and sociodemographic measures used in the study were collected during the imaging visit.

The exclusion criteria established by the UK Biobank team for MRI scanning include fairly standard MRI safety/quality criteria, such as exclusions for metal implants, recent surgery or health conditions directly problematic for MRI scanning—for example, hearing problems, breathing issues or extreme claustrophobia. Additionally, the raw MRI data that had the wrong dimensions, were corrupted, missing, or otherwise unusable were not processed any further.

In our study, out of the initial 157 316 data entries related to cannabis use, 141 420 were excluded due to either lacking MRI data, or missing confounding variables. No other exclusion criteria were applied. Consequently, the final analysis comprised 15 896 participants with complete cases.

### MRI acquisition and data processing

The imaging data were obtained on Siemens Skyra 3T MRI scanners equipped with 32-channel head coils. The UK Biobank team performed image processing, quality control checks and automated brain tissue volume computations; their imaging-derived phenotypes (IDPs) were made available to the researchers. The brain imaging protocol used in the UK Biobank includes structural, diffusion and functional imaging from six distinct modalities: T1-weighted, T2-weighted flair, diffusion MRI, susceptibility-weighted imaging, task functional MRI and resting-state functional MRI time series data.^13^

T1-weighted measures estimated grey matter and cortical measure. T2-weighted flair identified white matter hyperintensities and periventricular white matter hyperintensities. Diffusion MRI derived measures of white matter volume and white matter microstructure (such as FA, MD, axial diffusivity (L1), radial diffusivities (L2, L3) and mode of anisotropy from DTI, and intracellular volume fraction, isotropic volume fraction, and orientation dispersion). Task functional MRI employed the Hariri faces/shapes ‘emotion’ task, which represented summary measures of activation in regions chosen from the group-level activation map. Resting‐state functional MRI conducted at two distinct dimensionalities (25 and 100), resulting in 21 and 55 signal networks, provided information on measures of both within-network and between-network functional connectivity. Additional details on the acquisition parameters, image processing, and specific measurements derived from each imaging modality is in online supplemental methods. A total of 3921 brain measures of structural and functional connectivity were used in the analysis (online supplemental table 1).

### Cannabis use data

Cannabis use was self-reported at the online follow-up during the first repeat assessment visit. Participants reported if they had ‘Ever taken cannabis’. Possible answers were: ‘no’, ‘prefer not to say’, ‘yes, 1–2 times’, ‘yes, 3–10 times’, ‘yes, 11–100 times’ and ‘yes, more than 100 times’ (online supplemental figure 1). All participants who responded ‘yes’ were categorised as lifetime cannabis users, and ‘no’ responders were categorised as controls. Cannabis users were further divided into two subgroups: (a) low-frequency cannabis use (lifetime cannabis use of 1–10 times), and (b) high-frequency cannabis use (lifetime cannabis use of 11–100+ times). This subgroup categorisation for cannabis users was introduced in a previous study.^14^ Participants also reported their ‘Age when last taken cannabis’ and we computed years since the participants last had cannabis by the difference between the age when last cannabis was used and the age when subjects were scanned. Additionally, only two participants were identified with cannabis use disorder in UK Biobank, which was insufficient for conducting an analysis.

### Genetic variants

We examined summary genome-wide data based on two different cannabis phenotypes. Detailed information on the single nucleotide polymorphisms (SNPs) used to instrument these phenotypes is provided in online supplemental table 2. In order to fulfil a key MR assumption that genetic variants are robustly associated with the exposure (cannabis), we selected only variants at genome-wide significance, from the largest available genome-wide association study (GWAS) worldwide. F statistics were calculated as a quantitative measure of instrument strength (online supplemental table 2).

For the first exposure variable, we used the GWAS summary statistics for *cannabis dependence or abuse* (n=1 054 365) from individuals of European, African, admixed American and East Asian ancestries. Ancestry-specific linkage disequilibrium clumping was performed using PLINK v2.0 with the respective 1000 Genomes Project phase III linkage disequilibrium reference panels. Lead variants were identified within 10 000 kb and LD r^2^=0.001. This GWAS identified 23 genome-wide significant independent SNPs. The IDPs used as our outcome variable had 20 matching SNPs, so we identified proxy SNPs (R^2^ > 0.9) from LDlink (https://ldlink.nci.nih.gov/). Of the missing three SNPs, one SNP was monoallelic leaving a total of 22 SNPs in our analysis (online supplemental table 2a).^15^

The second exposure variable consisted of the GWAS summary statistics for *lifetime cannabis use* from the International Cannabis Consortium, 23andMe and UK Biobank (n=1 84 765) from individuals of European ancestry. Participants reported if they had ever used cannabis during their lifetime and the response was recorded as yes or no. This GWAS identified eight genome-wide significant independent SNPs. The estimated SNP heritability (h^2^_SNP_) for *lifetime cannabis use* was 11% (online supplemental table 2a).^16^

We obtained summary statistics for each of the brain IDPs as the outcome variable from the GWAS performed by the UK Biobank, which included approximately 33 000 participants.^17^

For the reverse MR analysis, the brain IDPs GWAS identified one SNP associated at genome-wide significance (p<5×10^-8^) with the FA of the genu of the corpus callosum, two SNPs associated with resting-state functional MRI connectivity (ICA25 edge 21 and ICA100 edge 55) that matched with SNPs in *cannabis dependence or abuse* GWAS. Notably, only one SNP associated with resting-state functional MRI connectivity (ICA25 edge 21) had a matching SNP in *lifetime cannabis use* GWAS (online supplemental table 2b).

### Confounds

We adjusted for potential confounds, which were self-reported at the time of the MRI scan. Age at first scan (in years), sex (male and female), and also age^∧^2, age^∧^3, and age-by-sex interaction were controlled for. Townsend deprivation is a measure of material deprivation based on census information. Current employment status was recorded as: in paid employment/self-employed, retired, looking after home and/or family, unable to work because of sickness or disability, unemployed, doing unpaid or voluntary work, full-time or part-time student or none of these. Educational qualifications were recorded as: college or university degree, A level/AS levels or equivalent, O levels/GCSEs or equivalent, CSEs or equivalent, NVQ or HND or HNC or equivalent, other professional qualifications or none of the above. Smoking and alcohol drinking status was reported as: current, previous or never. Systolic and diastolic blood pressure were measured in mm Hg and body mass index in kg/m^2^. For measurement of mental health status, participants were asked if they had ‘seen a psychiatrist for nerves, anxiety, tension or depression’ and the response was noted as ‘yes’, or ‘no’.

We also accounted for a set of 613 brain imaging-related confounds in this sample as described in Alfaro-Almagro *et al* (2020).^18^ These included: assessment centre, intracranial volume, head motion, table position and scanner acquisition parameters (site, scanner software, protocol, scan ramp, head coil) (online supplemental table 3).

### Statistical analyses

All statistical analysis was performed in R (version 4.0.0) and visualisations were performed in MATLAB (version R2018_a). Independent samples t tests and Χ^2^ analyses were performed to assess potential univariate differences in the sociodemographic characteristics between the cannabis users and controls. Multiple linear regression was performed to determine the relationship between cannabis use and brain measures, accounting for confounds.

To begin with, in a hypothesis-driven approach, we examined the association between cannabis use and grey matter volume of the hippocampus for two main reasons. First, past studies on adolescents and young adults have consistently indicated alterations in hippocampal volume associated with cannabis use. Second, cannabinoid CB1 receptors are known to be expressed in this region. Subsequently, we employed an exploratory approach to examine the association between cannabis use and brain structure and function by using all brain IDPs. A total of 3921 brain IDPs were tested with an adjusted cut-off p value of 0.05 using Bonferroni correction. For comparison, p values in the analyses were also corrected for multiple testing using false discovery rate (FDR, 5%).

We then performed sensitivity analyses controlling for the covariates. These were performed for the cannabis–IDPs associations that remained statistically significant after adjusting for multiple comparisons using the FDR test. We performed two sensitivity analyses among cannabis users to assess whether: (1) years of cannabis abstinence, and (2) cannabis dose (low vs high frequency), modified cannabis–brain associations. Additionally, we conducted a sex-stratified analysis as a result of a peer review suggestion.

Finally, we performed two-sample MR analyses by using the *TwoSampleMR* in an R package to investigate whether significant observed associations between cannabis use and brain IDPs were causal. P values in the analyses were additionally adjusted for multiple testing FDR. To test for the presence of horizontal pleiotropy, a violation of a key MR assumption, we used the MR-Egger intercept test. Additional details on other robust MR methods performed are provided in the online supplemental methods.

## Findings

### Demographics

There were 3641 cannabis users and 12 255 control subjects with complete data (online supplemental figure 2). Individuals with complete as opposed to incomplete cannabis data had a lower proportion of men, a slightly lower proportion were in employment and a higher proportion had college degrees (online supplemental table 4a). Only two individuals had cannabis abuse or dependence ICD codes in the linked electronic health record. Cannabis users were significantly younger than non-users (online supplemental table 4b). While the subjects were well matched for body mass index and diastolic BP, the user group had significantly lower systolic BP and were less socially deprived than the control group. There was a slightly higher proportion of men in the user group, a higher proportion of the user group were in employment and had college degrees. A higher proportion of cannabis users than controls drank alcohol and smoked cigarettes. Additionally, a higher proportion of the user group complained of nerves/anxiety/tension/depression (online supplemental table 4b).

### Observational analysis

In view of previously observed associations with hippocampal volume, we examined this region as a region of interest in a hypothesis-driven approach. There were no significant associations observed with cannabis use (online supplemental table 5).

**Figure 1:**
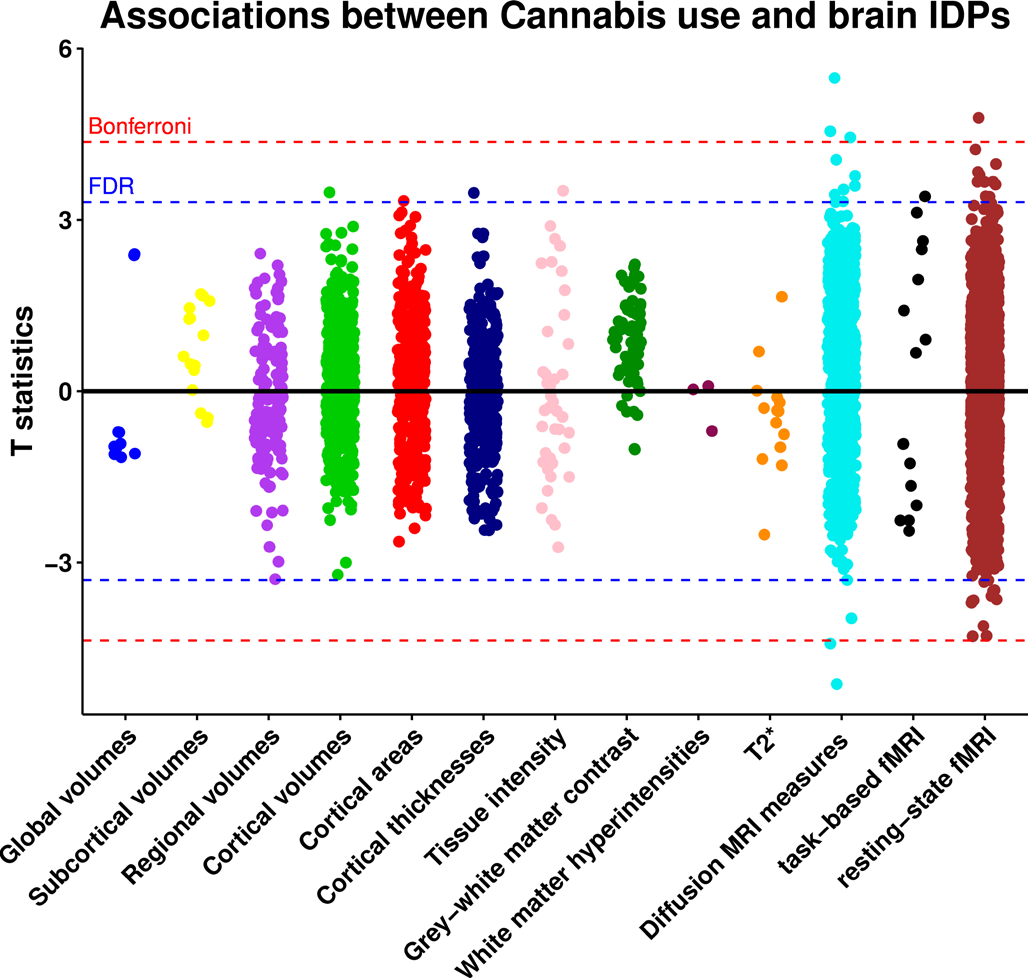
Associations between cannabis use and brain image-derived phenotypes. Estimates were generated using multiple linear regression models adjusted for: age, sex, Townsend deprivation index, employment status, educational qualifications, alcohol drinking status, smoking status, body mass index, systolic and diastolic blood pressure, assessment centre, nerves/anxiety/tension/depression status and brain imaging confounds including assessment centre, intracranial volume, head motion, table position, and scanner acquisition parameters (site, scanner software, protocol, scan ramp, head coil). Red line indicates the Bonferroni threshold (3921 tests, p=1.28 × 10^-5^, T statistics=4.36) and blue line indicates the false discovery rate threshold (3921 tests, p=9.38 × 10^-4^, T statistics=3.31). Abbreviations: FDR, false discovery rate; fMRI, functional MRI; IDPs, image-derived phenotypes.

**Figure 2:**
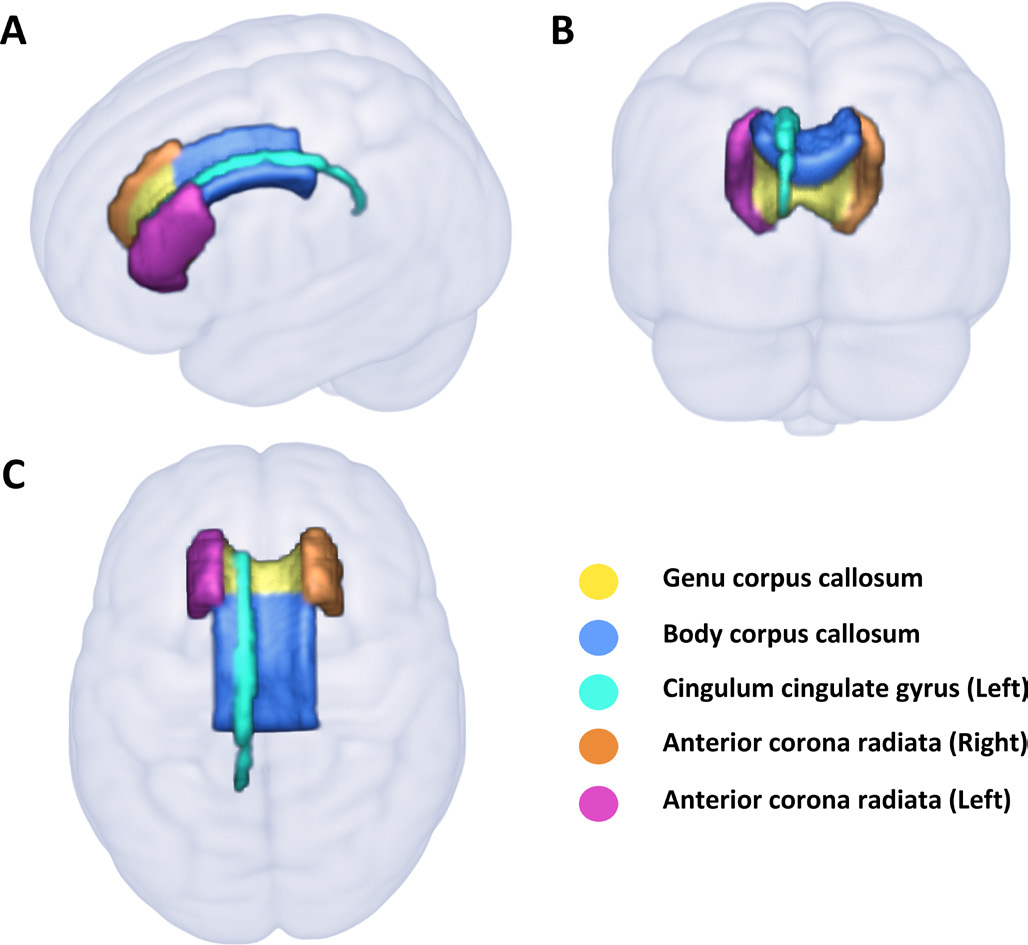
White matter regions significantly associated with cannabis use. Structures delineated by the JHU White Matter Atlas (ICBM-DTI-81) are presented in (A) sagittal, (**B**) coronal and C) axial views.

Out of 3921 brain IDPs, cannabis use was significantly associated with 40 brain IDPs after FDR correction (0.05%, p=0.009) (figure 1, table 1, online supplemental table 6). The strongest associations were with measures of white matter microstructure. Most significant associations identified in the DTI metrics were found in the genu and body of the corpus callosum, demonstrating lower FA and intracellular volume fraction, as well as higher MD, and radial diffusivities (L2, L3). Furthermore, a higher MD was observed in the left cingulum cingulate gyrus, while increased L2 was detected in the cingulum bundle, and higher L2 and L3 were observed in the anterior corona radiate (figure 2).

**Table 1:** Summary of the association between cannabis use and brain measures after false discovery rate correction (5%)

Brain measures	Regions/tasks/networks		Direction/strength of association with cannabis use
Volume	Right inferior lateral ventricles		Positive
Area	Left frontal pole		Positive
Thickness	Left posterior ventral cingulum gyrus		Positive
Tissue Intensity	Right pallidum		Positive
Diffusion tensor imaging	FA	Genu and body of corpus callosum	Negative
	ICVF	Genu of corpus callosum	
	MD	Genu of corpus callosum, cingulum cingulate gyrus	Positive
	L2	Genu and body of corpus callosum, and left cingulum cingulate gyrus	
	L3	Genu and body of corpus callosum, genu and right and left anterior corona radiata	
Task-based functional MRI	BOLD fMRI activity to emotional faces shapes in whole brain		Stronger
Resting state functional MRI	Default mode network, central executive network, salience network, and motor network		Stronger
	Default mode network, central executive network, salience network, motor network, visual network, subcortical-cerebellum network, attention network and limbic network		Weaker

BOLD, Blood oxygenation level dependent; FA, fractional anisotropy; ICVF, intracellular volume fraction; L2 and L3, radial diffusivities; MD, mean diffusivity.

**Figure 3:**
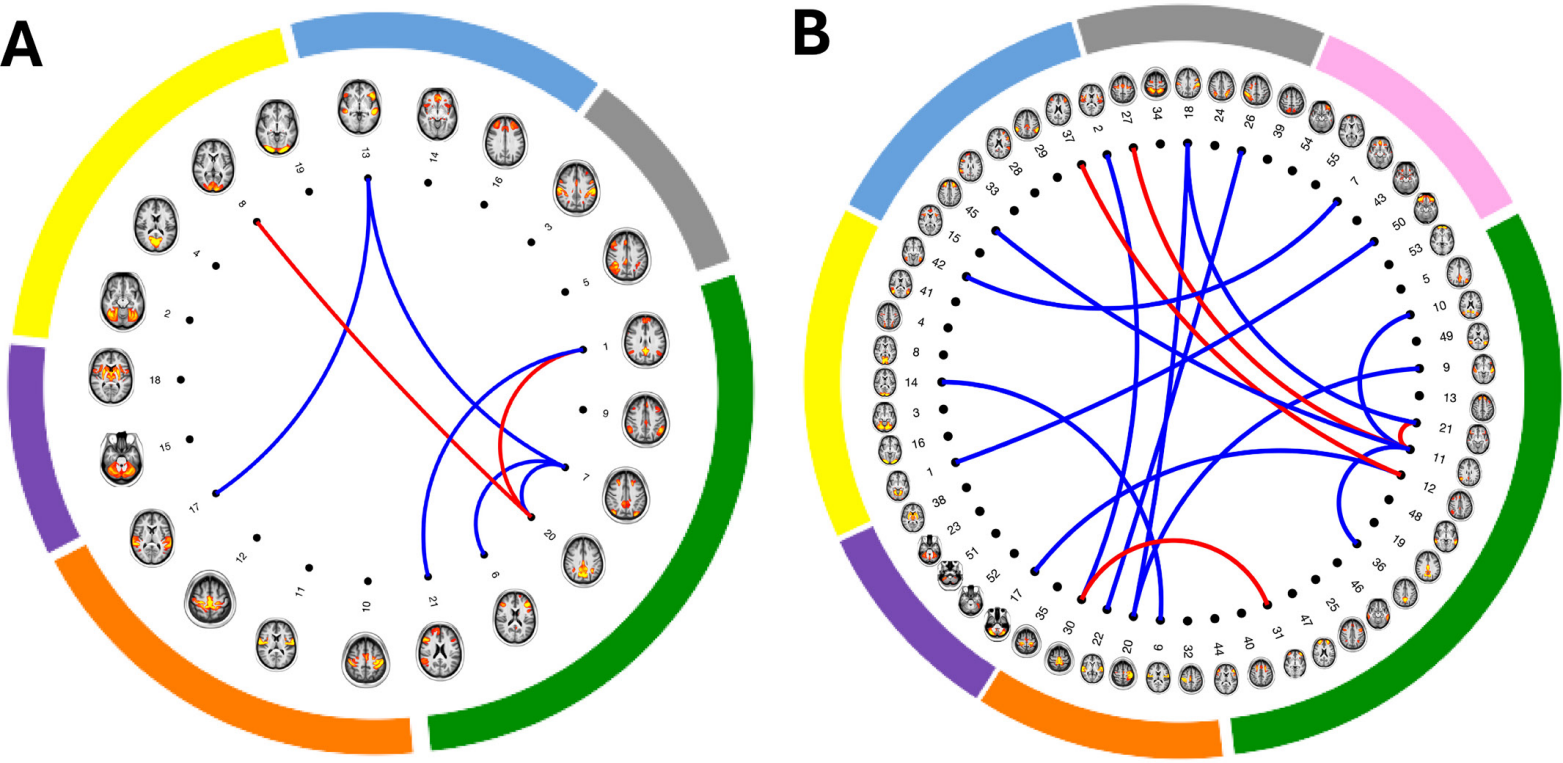
Resting state functional connectivity significantly associated with cannabis use. Spatial maps of resting state network nodes (n=21 and n=55) are illustrated in brain images. These were identified as non-noise components from: (A) 25-component group-independent component analysis proposed by Miller *et al*.^13^ Nodes are grouped by networks as follows: default mode/central executive network (green), motor network (orange) subcortical-cerebellum network (purple), visual network (yellow), salience/default mode/central executive network (blue) and attention/default mode/central executive network (grey). (B) 100-component group-independent component analysis. Nodes are grouped by networks as follows: default mode/central executive network (green), motor/attention network (orange) subcortical-cerebellum network (purple), visual/attention network (yellow), salience/default mode/central executive network (blue), attention/salience/central executive network (grey) and limbic/default mode network (pink). Connecting lines indicate partial correlations between network nodes significantly associated with cannabis (red lines indicate stronger connectivity and blue lines indicate weaker connectivity).

A wide range of associations was observed across various analyses of resting-state functional connectivity, particularly indicating either weaker or stronger connectivity between multiple networks. These networks predominantly included brain regions associated with the default mode, central executive and salience network. A visual representation of the resting-state networks as nodes and their connections that are significantly associated with cannabis use for both 21 and 55 resting-state networks obtained, respectively, from 25-component and 100-component group-ICA, is presented in figure 3 and online supplemental table 7.

Cannabis use was also associated with specific brain IDPs, including a larger volume of the right inferior lateral ventricles, a larger surface area of the frontal pole, greater thickness in the posterior ventral cingulate gyrus, higher tissue intensity in the right pallidum, and enhanced group-average blood oxygenation level dependent (BOLD) activation to emotional faces/shapes in whole brain, during task functional MRI.

Associations with six brain IDPs additionally survived the more stringent *Bonferroni-corrected* threshold (p=1.275 × 10^-5^). Cannabis users were characterised by lower FA and intracellular volume fraction, and higher MD, L2 and L3 in the genu of the corpus callosum. Additionally, cannabis users revealed lower functional connectivity between brain regions in the inferior frontal, middle frontal and precuneus regions, all of which are associated with the default mode and central executive network (online supplemental table 8).

### Sensitivity analyses

We assessed whether the duration of abstinence or dose had an impact on the relationships between cannabis use and brain IDPs that survived the FDR correction in the main analysis. Neither the duration of cannabis abstinence nor the frequency of cannabis dosage (as assessed through low- and high-frequency use) significantly moderated the associations between cannabis use and brain measures.

There were notable sex differences. After FDR correction, while significant associations were observed in six brain regions among men, women exhibited a more widespread effect across 24 brain structures and functional regions. Among male cannabis users, most associations were observed in functional connectivity, whereas in women, associations were primarily seen in diffusion MRI measures of white matter, with the genu and body of corpus callosum showing the most significant association (online supplemental tables 9 and 10).

### Mendelian randomisation

After correcting for multiple comparisons, there were no significant associations between genetically predicted *cannabis dependence/abuse* (inverse-variance weighted (IVW) beta=0.01 (95% CI −0.09 to 0.11), p=0.85) or *lifetime cannabis use* (IVW beta=−0.05 (95% CI -0.15 to 0.05), p=0.33), respectively, and FA in genu of the corpus callosum. No significant association was observed with other brain IDPs, either. There was no indication of horizontal pleiotropy as determined by the MR-Egger intercept test for any of the outcomes (online supplemental table 11a and online supplemental figure 3a). Reverse MR analysis did not show any significant association between brain IDPs and *cannabis dependence or abuse* or *lifetime cannabis use* (online supplemental STable 11b and online supplemental figure 3b).

## Discussion

To the best of our knowledge, this is the largest observational study of relationships between cannabis use and brain structure and function to date, and the first Mendelian randomisation investigation. Cannabis users had significant differences in brain structure and function, most markedly for markers of lower white matter microstructure integrity. Genetic analyses found no support for causal relationships underlying these observed associations.

Cannabis users showed lower fractional anisotropy and higher mean diffusivity in the genu of the corpus callosum compared with non-users. Previous studies of the frequent use of high-potency cannabis by adolescents and young adults have reported disruption in corpus callosum integrity. Taken together, this might suggest that the corpus callosum is particularly sensitive to high tetrahydrocannabinol concentration.^19^ Cannabis users had lower white matter integrity, as measured by higher radial diffusivity (L2, L3) of the anterior corona radiata and higher mean diffusivity in the left cingulum. Although no associations in these diffusion metrics have been observed in past studies, disrupted microstructural integrity in the cingulum was reported with other diffusion measures showing higher fractional anisotropy of the cingulum.^20^

Cannabis use is significantly associated with resting-state functional connectivity, mainly in the brain regions underlying default mode, central executive and salience networks. The brain regions underlying these networks were primarily located in the frontal lobe, temporal lobe, occipital cortex, supplementary motor area, precuneus and cerebellum. These regions are characterised by a high density of cannabinoid receptor type 1, and have also been implicated in younger cannabis users.^21 22^ Higher functional connectivity between prefrontal cortex and occipital cortex has been reported in young adult chronic cannabis users compared with controls.^22^ Altered patterns of functional connectivity between cerebellum to cerebral cortex that might have an impact on behaviour and cognition have been reported previously.^23^ Additionally, increased functional connectivity has been reported in regions underlying orbito-frontal cortex with precuneus and cerebellar regions, which might indicate an impairment in decision-making capacity and an increase in impulsive behaviour.^21^ Our findings, however, suggest a complex pattern of higher or lower functional connectivity between these regions with cannabis use.

A few other brain structures also showed associations with cannabis use. Cannabis users showed a higher surface area of the left frontal pole and higher tissue intensity in the right pallidum. The literature has not previously reported any similar associations with these measures, but others have reported a higher volume of the pallidum,^24^ and in contrast, a decreased gyrification of the frontal pole.^25^ We also observed novel associations with left inferior lateral ventricle volume and left posterior ventral cingulum gyrus thickness.

Insights derived from animal studies have shown structural changes associated with THC, the principal psychoactive compound of cannabis, in brain regions rich in CB1 receptors.^26 27^ These changes observed in hippocampal morphology resemble those observed following ischaemic or toxic damage.^27^

We did not replicate previously observed associations between cannabis use and grey matter volume in the hippocampus. One possible explanation is the differing age range of subjects. Previous studies examined adolescents and young adults, whereas our sample comprised middle- to late-life adults. White matter microstructural changes might also be more sensitive to cannabis effects than the grey matter measures used in this study.

We found no influence between the duration of abstinence from cannabis use prior to the brain scan and structural or functional brain connectivity. Additionally, we did not find any significant differences in structural and functional connectivity between low- and high-frequency cannabis users, thus indicating the absence of a dose–response relationship. This might be a result of our sample characteristics, consisting of healthy volunteers and a few high cannabis users. However, past studies suggest that both frequency and duration of cannabis use might have an impact brain function and structure. Specifically, regular cannabis use during adolescence was associated with a higher risk of developing cannabis dependence, and the risk of developing cannabis use disorders was higher among those who started early and used frequently during adolescence.^28^ Additionally, the prevalence of cannabis use disorder increases over time since initiation of use, with a steeper increase observed among youth compared with emerging adults, indicating that duration of use might also contribute to changes in brain function and structure.^29^ However, further research is needed to elucidate the specific patterns and mechanisms underlying these effects.

The examination of sex differences with cannabis use showed intriguing insights. The observed differences in brain regions and functional measures suggest potential variations in how cannabis affects men and women. The more widespread impact seen in women, particularly in white matter, might indicate differential vulnerability to cannabis-related neurotoxicity. Further research is warranted to elucidate the underlying mechanisms driving these sex-specific differences and their implications for the long-term effects of cannabis use on brain structure and function. While the impact of gender on cannabis effects remains unclear, a systematic review revealed mixed results.^30^ Among the studies examined (n=11), the majority found no sex-specific interactions, yet a subset (n=8) suggested that females might exhibit increased vulnerability to cannabis-related neurotoxicity. Additionally, studies lacking sex differences often had fewer females, affecting statistical power, and underscoring the need for a more in-depth exploration into the underlying mechanisms driving these sex-specific differences.^30^

In our study, the observed disparity between regions with structural and functional alterations is noteworthy. Structural and functional changes in the brain might occur at different time scales, potentially leading to disparities in imaging results. For example, functional alterations might precede or follow structural changes. The most significant association observed in our study was in the corpus callosum microstructure, which plays a crucial role in interhemispheric communication. Changes in this structure might have widespread, yet varied effects on brain function, affecting different networks and regions differently. Differences in structural and functional alterations might also stem from the diverse functional roles of various brain regions. Overall, our results illuminate the intricate interplay between structural and functional brain alterations, emphasising the need for comprehensive investigations to uncover their underlying mechanisms.

Mendelian randomisation provided no support for a causal effect of cannabis use or dependence on brain structure or function, nor a causal effect of brain structure or function on cannabis use. The disparity between observational and Mendelian randomisation findings could result from several mechanisms. First, the observational associations might be confounded by an unmeasured variable, such as family history, dietary intake or use of certain medications. Second, our Mendelian randomisation analyses had less statistical power than our observational analyses to detect small effects. However, despite potential concerns about weak instrument bias in Mendelian randomisation studies, our SNPs demonstrated robust instrument strength, evident from the F-statistics. Nevertheless, future larger-scale neuroimaging GWAS will be helpful in distinguishing between these two hypotheses. Finally, Mendelian randomisation assesses the lifelong impact of cannabis use, while changes in observational studies might be due to factors occurring at different points in an individual’s life rather than as the sum of lifelong impact.

### Limitations

Our study has several limitations. First, although our sample size was bigger than that of previous studies, UK Biobank is healthier than the general population. It suffers from selection bias with respect to sociodemographic variables, such as physical, lifestyle and health-related characteristics, as well as a lower prevalence of cannabis use disorders compared with a population sample. Furthermore, this sample is not suitable for examining cannabis use disorders due to the limited number of participants with this diagnosis. Second, as the age of cannabis initiation was not recorded, we were unable to examine the time points during life critical for cannabis effects. Third, participants are susceptible to recall and reporting bias concerning the amount or frequency of cannabis intake in their lifetime, and their response might be affected by external biases arising from social desirability. Determining historical cannabis use is challenging: urine drug screens have a limited detection period, typically just a few days, while hair analyses cover a longer period of months, which is still limited by hair length. Fourth, the self-report did not include the potency of cannabis consumed, which might differ across the participants and over time. Fifth, despite our effort to account for potential confounds, the presence of unmeasured confounding variables (residual confounding) cannot be excluded. Sixth, our categorisation of high-frequency cannabis users is likely to result in a heterogeneous group due to the broad nature of the classification. Our ability to investigate the heterogeneity of cannabis effects according to use patterns is thus constrained by the data available. Seventh, equally we were not able to distinguish between dose-dependent and frequency-dependent effects. Eighth, neuroimaging measures were cross-sectional, and longitudinal relationships cannot be inferred. Finally, MR relies on several assumptions, for which we have tried to test where possible. The assumption that there is no unmeasured confounding between genetic variants and outcomes is not testable in an observational study.

## Clinical implications

Lifetime cannabis use was associated with several measures of brain structure and function in later life, particularly in the corpus callosum. Genetic analysis did not provide support that these associations result from causal relationships. Discrepancies between observational and genetic analyses could be explained by residual confounding—that is, confounding variables unaccounted for in the observational analysis. Thus, our results need to be interpreted with careful consideration. Additional research is needed to understand the effects of heavy cannabis use in this population, including considerations of potency and related information to inform public policy.

## Supplementary material

10.1136/bmjment-2024-301065 online supplemental file 1

10.1136/bmjment-2024-301065 online supplemental file 2

10.1136/bmjment-2024-301065 online supplemental file 3

## Data Availability

Data may be obtained from a third party and are not publicly available.
