# Supplementary material for: Association between cannabis use and brain structure and function: an observational and Mendelian randomisation study
Source: BMJ Ment Health. 2024 Oct 30;27(1):e301065. doi: 10.1136/bmjment-2024-301065 (PMC11529520; doi:10.1136/bmjment-2024-301065)

# Supplementary figures

Table of Contents

**SFigure 1: Lifetime cannabis use in UK Biobank participants analysed. ..............1**

**SFigure 2: Flow chart of the participants included in the final analysis. ...............2**

**SFigure 3a: Two-sample linear MR plot for the causal effect of Cannabis dependence or abuse/ Lifetime Cannabis use on brain measures ...........................3**

**SFigure 3b: Reverse two-sample linear MR plot for the causal effect of brain measures on Cannabis dependence or abuse .........................................................10**

**SFigure 1: Lifetime cannabis use in UK Biobank participants analysed.**

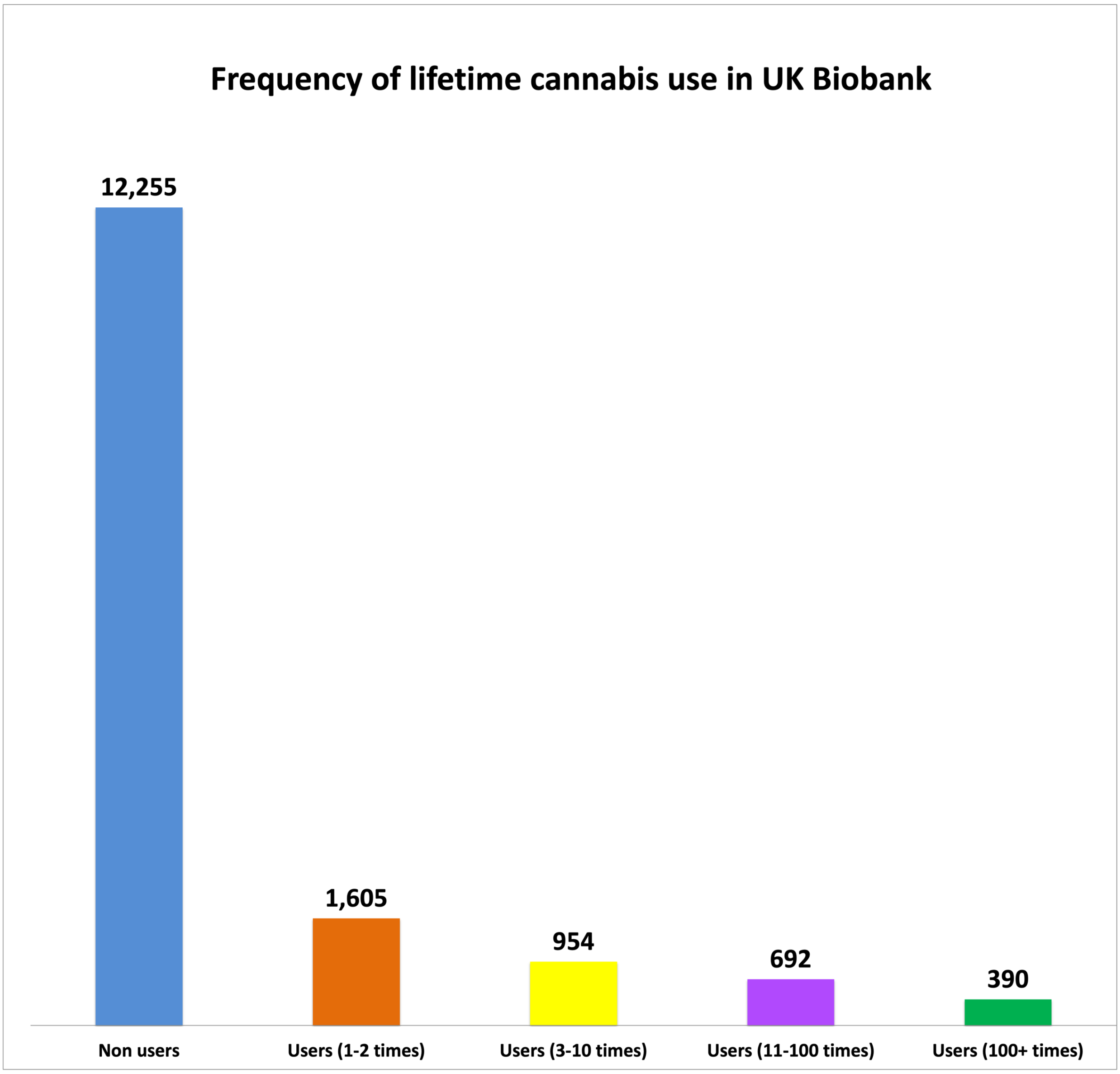

**SFigure 2: Flow chart of the participants included in the final analysis.**

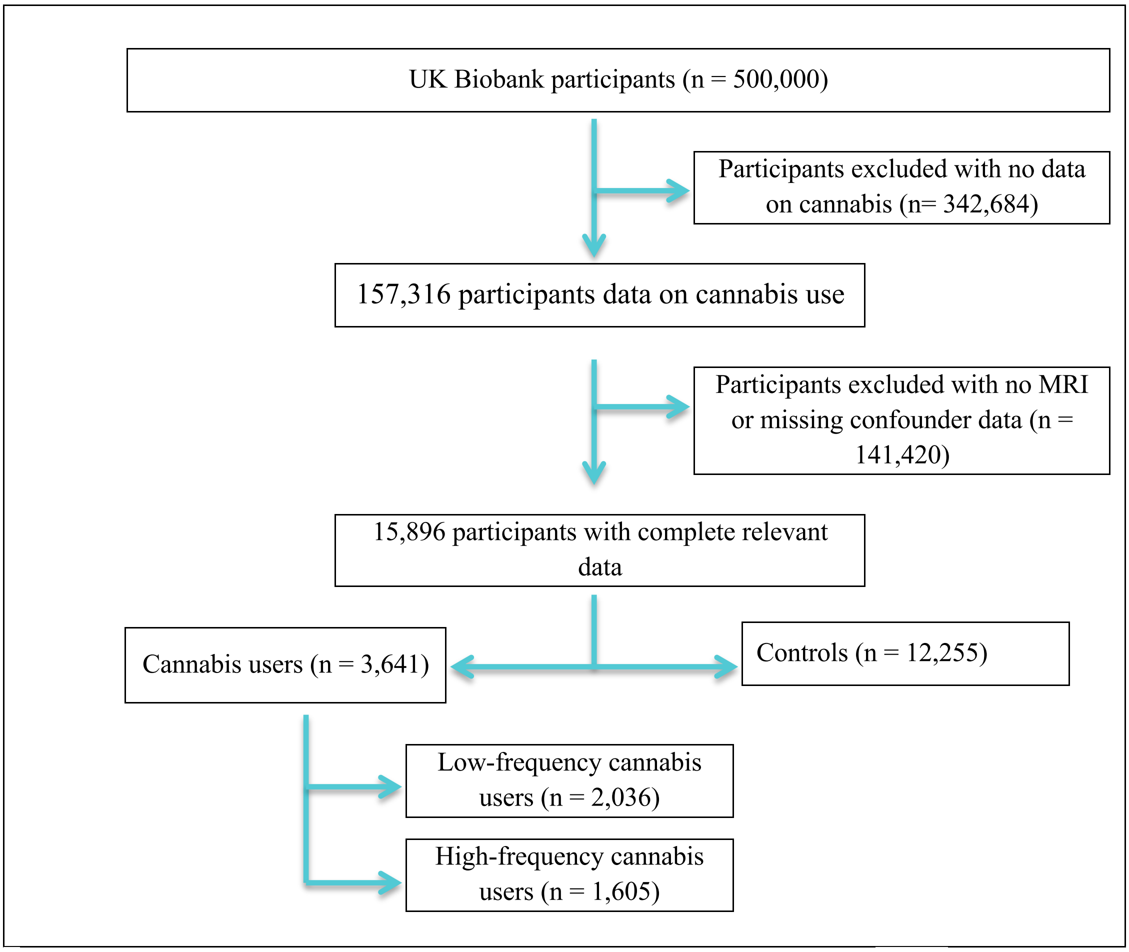


Low-frequency cannabis use defined as lifetime cannabis use of 1-10 times, and high-frequency cannabis use defined as lifetime cannabis use of 11-100+ times.
Abbreviations: MRI, magnetic resonance imaging.

## SFigure 3a: Two-sample linear MR plot for the causal effect of Cannabis dependence or abuse/ Lifetime Cannabis use on brain measures

### Cannabis dependence or abuse and brain measures

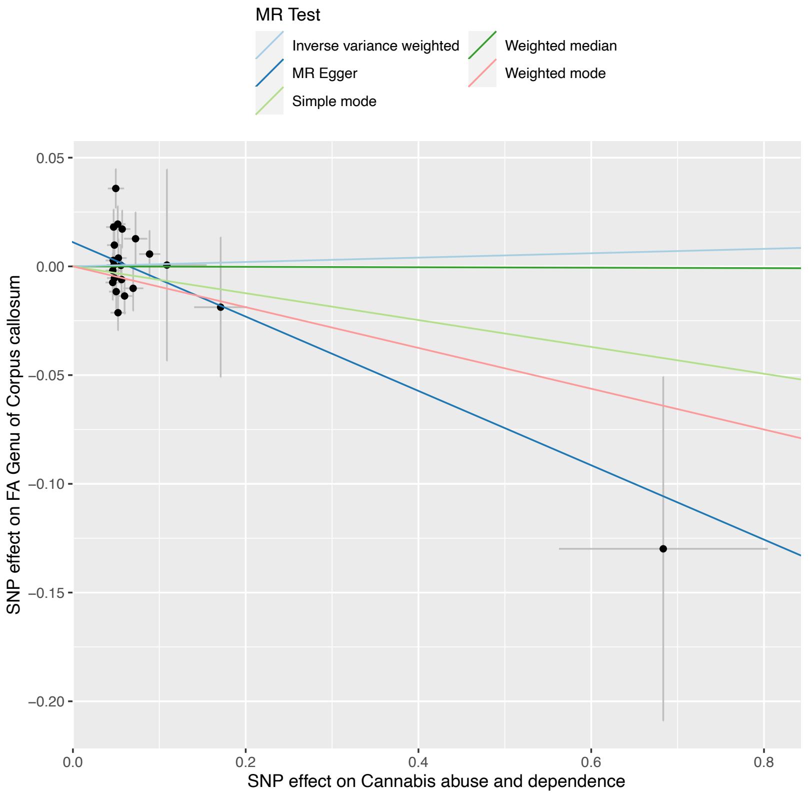


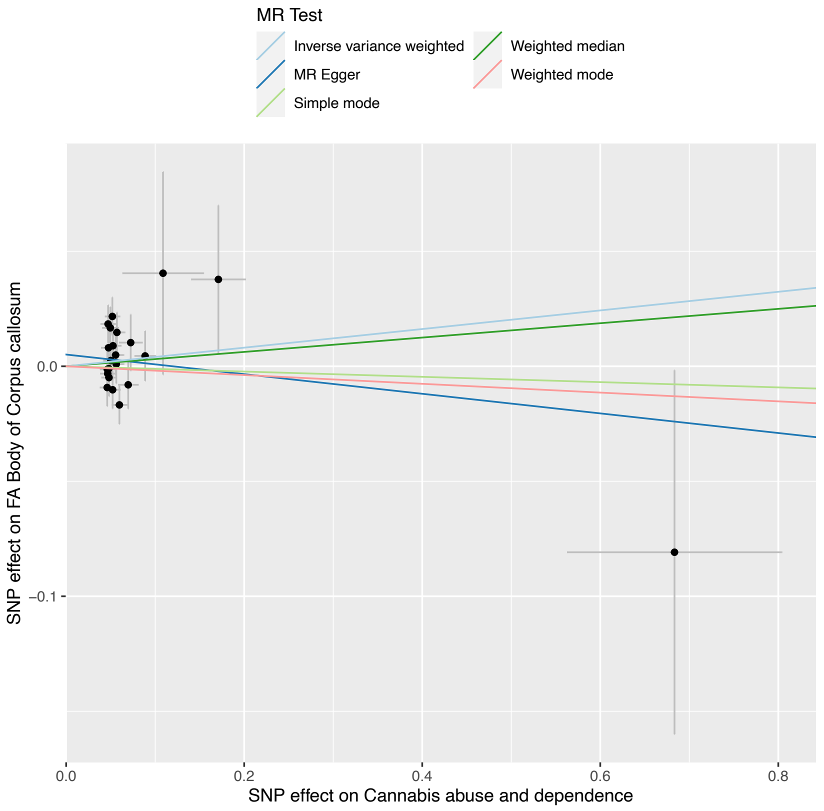

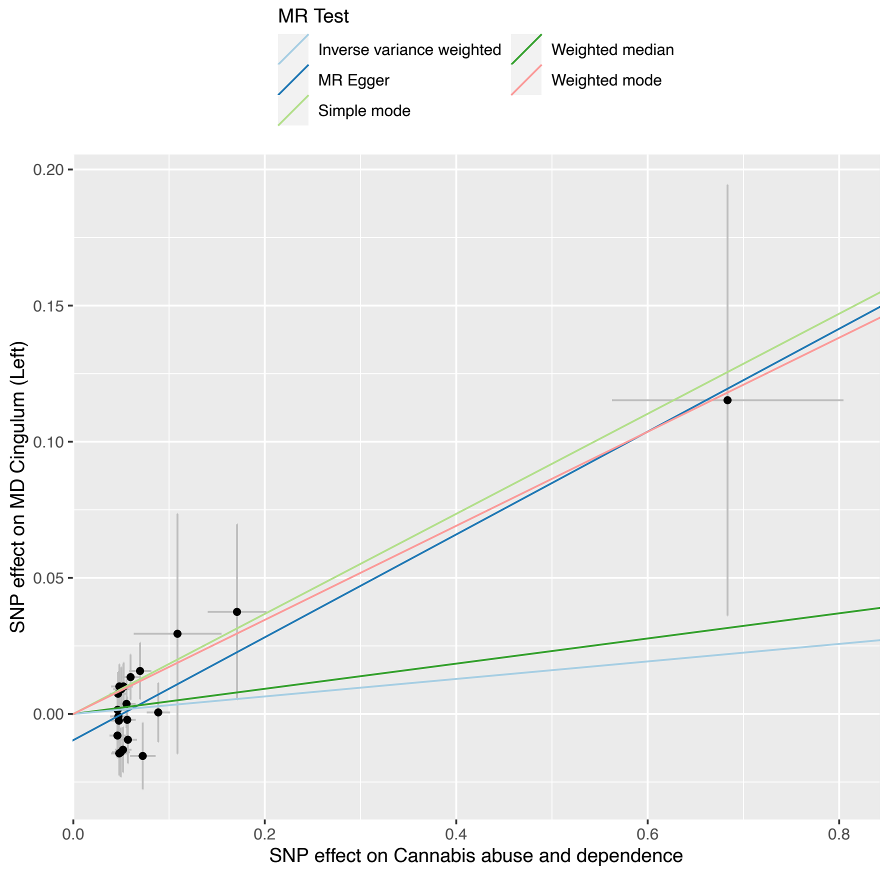

MR Test
Inverse variance weighted
MR Egger
Simple mode
Weighted median
Weighted mode
0.20
0.15
0.10
0.05
0.00
SNP effect on MD Cingulum (Left)
0.0
0.2
0.4
0.6
0.8
SNP effect on Cannabis abuse and dependence


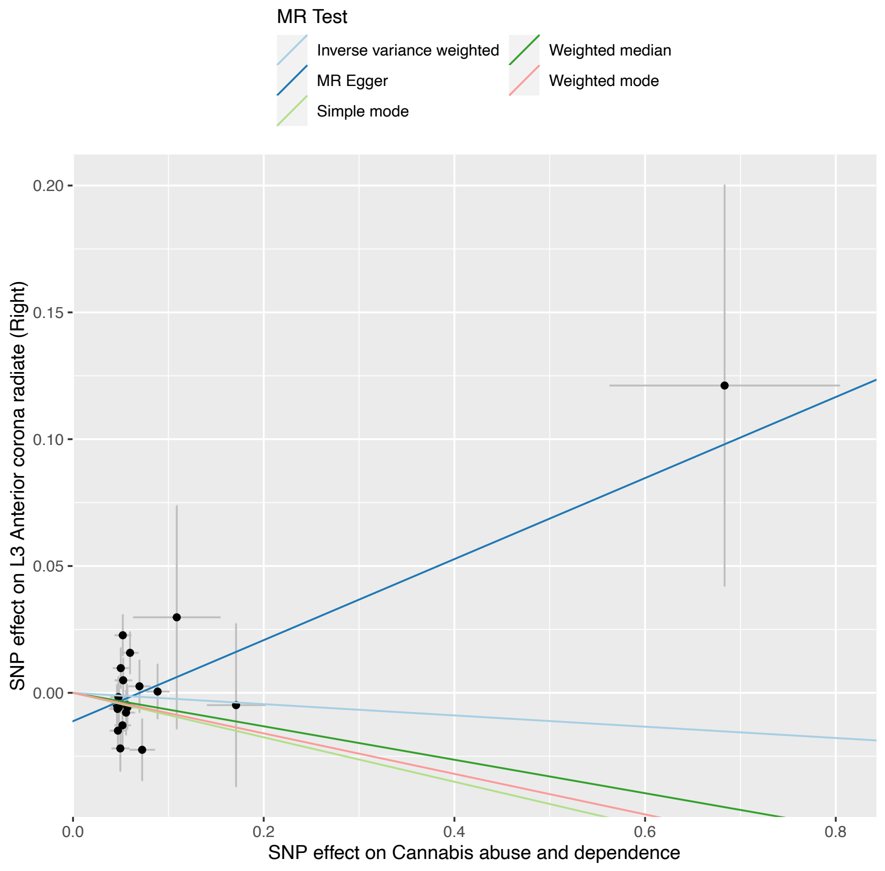

MR Test
Inverse variance weighted
MR Egger
Simple mode
Weighted median
Weighted mode
0.20
0.15
0.10
0.05
0.00
SNP effect on L3 Anterior corona radiate (Right)
0.0
0.2
0.4
0.6
0.8
SNP effect on Cannabis abuse and dependence

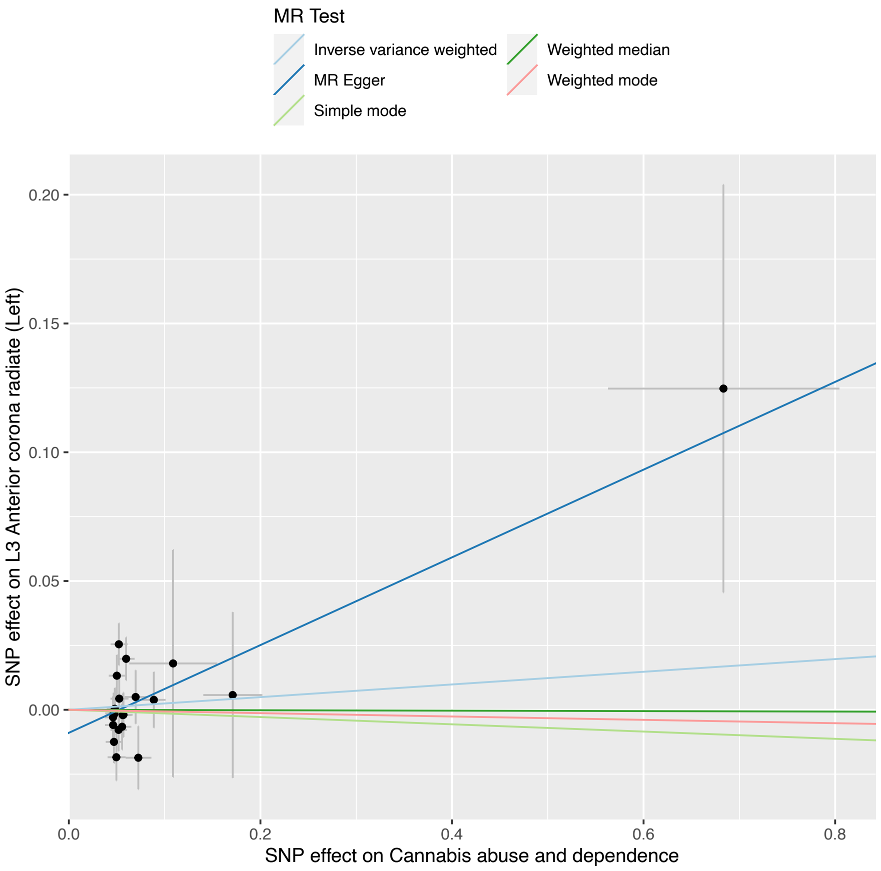
MR Test
Inverse variance weighted
MR Egger
Simple mode
Weighted median
Weighted mode
0.20
0.15
0.10
0.05
0.00
0.0
0.2
0.4
0.6
0.8
SNP effect on L3 Anterior corona radiate (Left)
SNP effect on Cannabis abuse and dependence

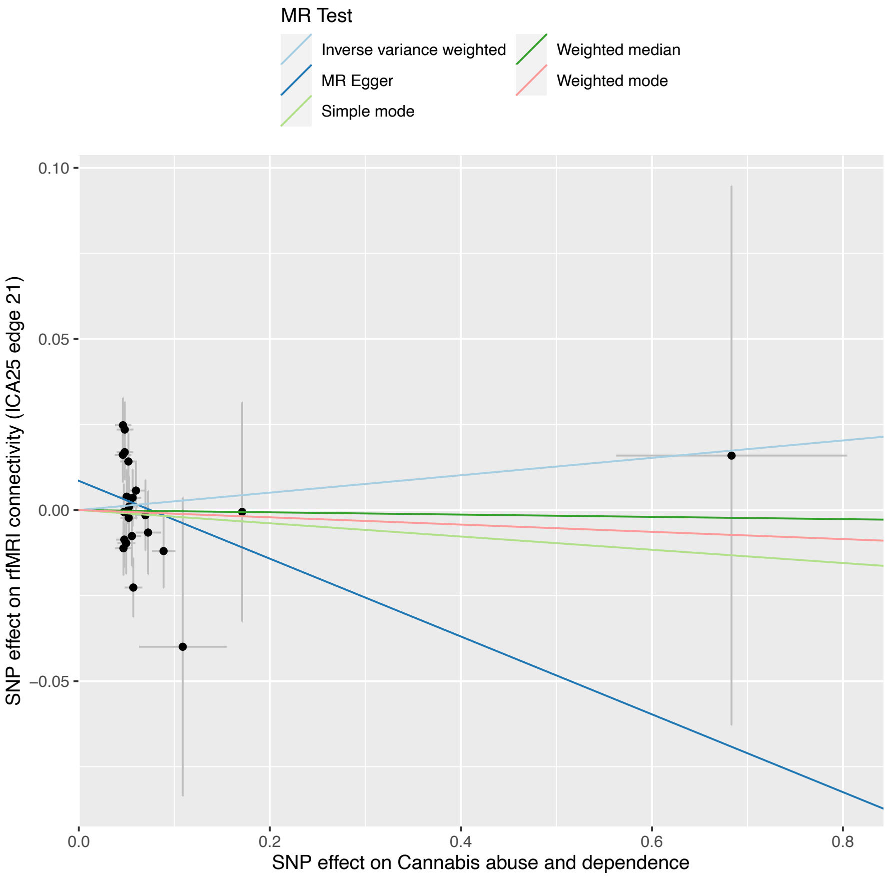
MR Test
Inverse variance weighted
MR Egger
Simple mode
Weighted median
Weighted mode
0.10
0.05
0.00
−0.05
0.0
0.2
0.4
0.6
0.8
SNP effect on rfMRI connectivity (ICA25 edge 21)
SNP effect on Cannabis abuse and dependence

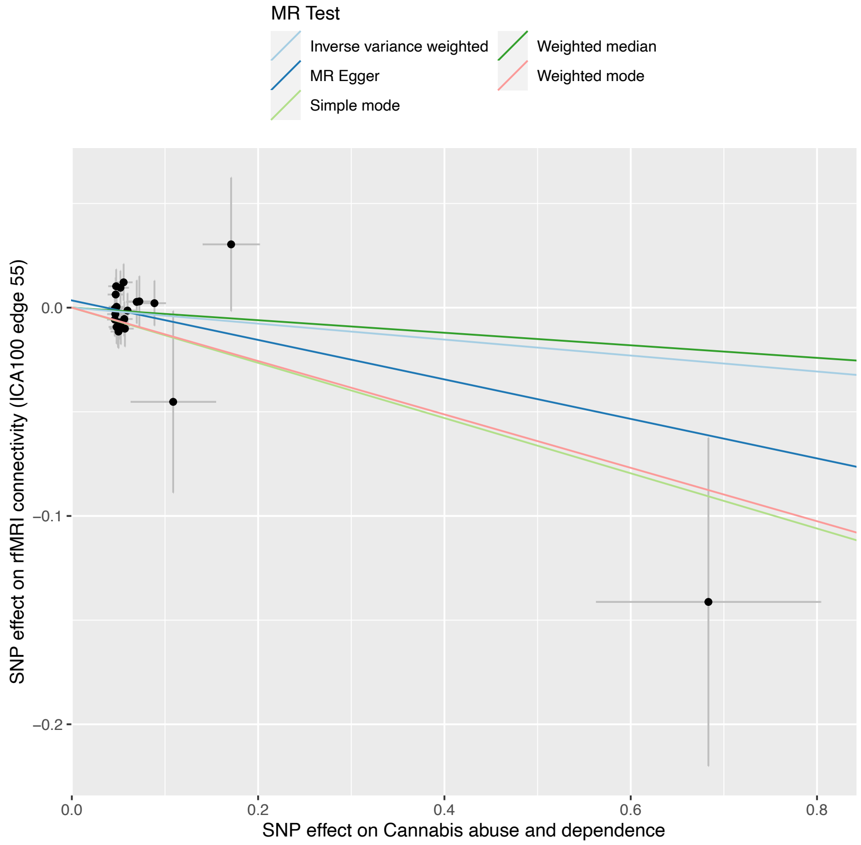


**Lifetime Cannabis use and brain IDPs**

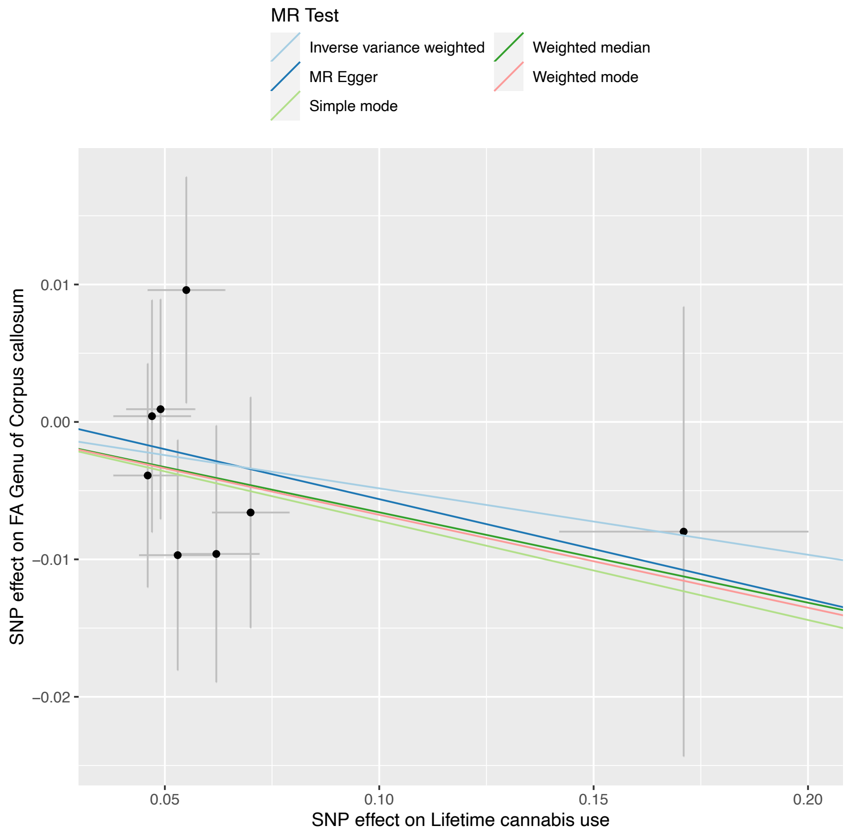

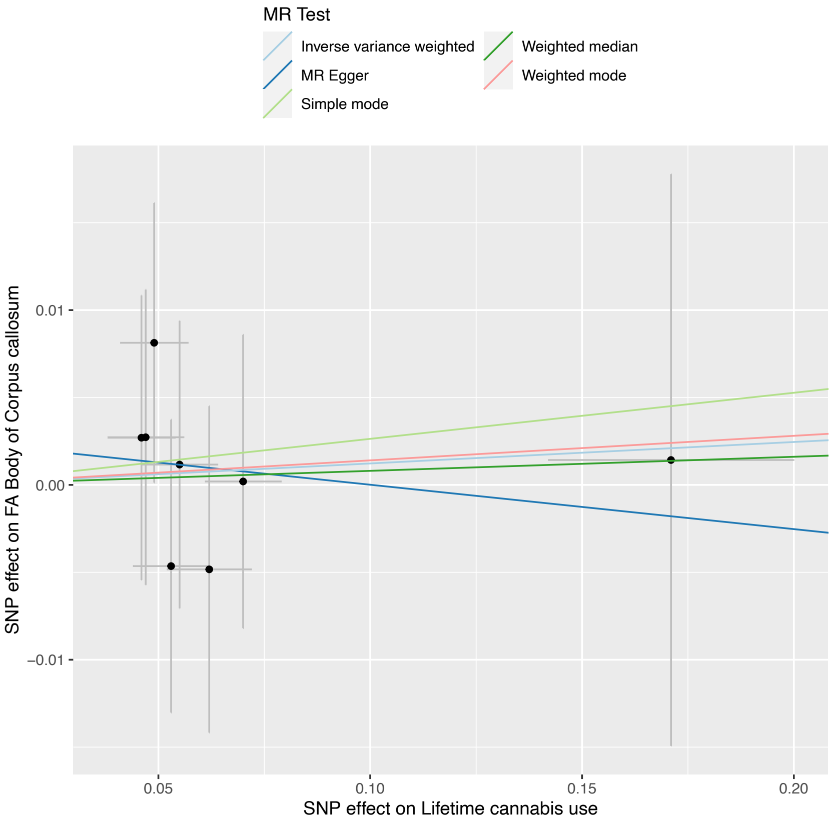
MR Test
Inverse variance weighted
MR Egger
Simple mode
Weighted median
Weighted mode
SNP effect on FA Body of Corpus callosum
0.01
0.00
−0.01
0.05
0.10
0.15
0.20
SNP effect on Lifetime cannabis use

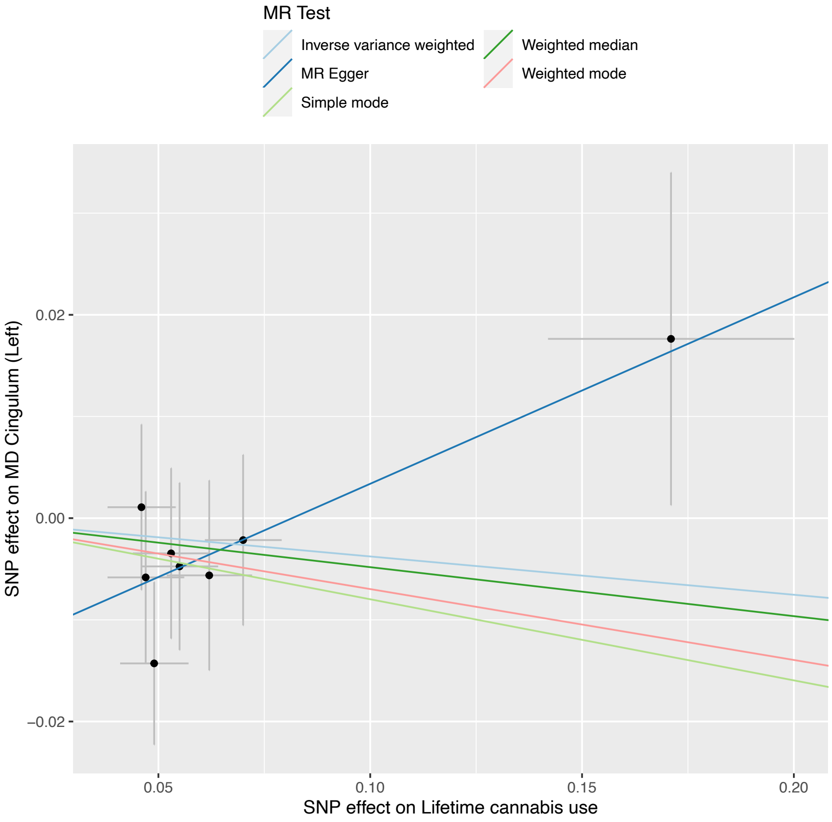
MR Test
Inverse variance weighted
MR Egger
Simple mode
Weighted median
Weighted mode
SNP effect on MD Cingulum (Left)
0.02
0.00
−0.02
0.05
0.10
0.15
0.20
SNP effect on Lifetime cannabis use

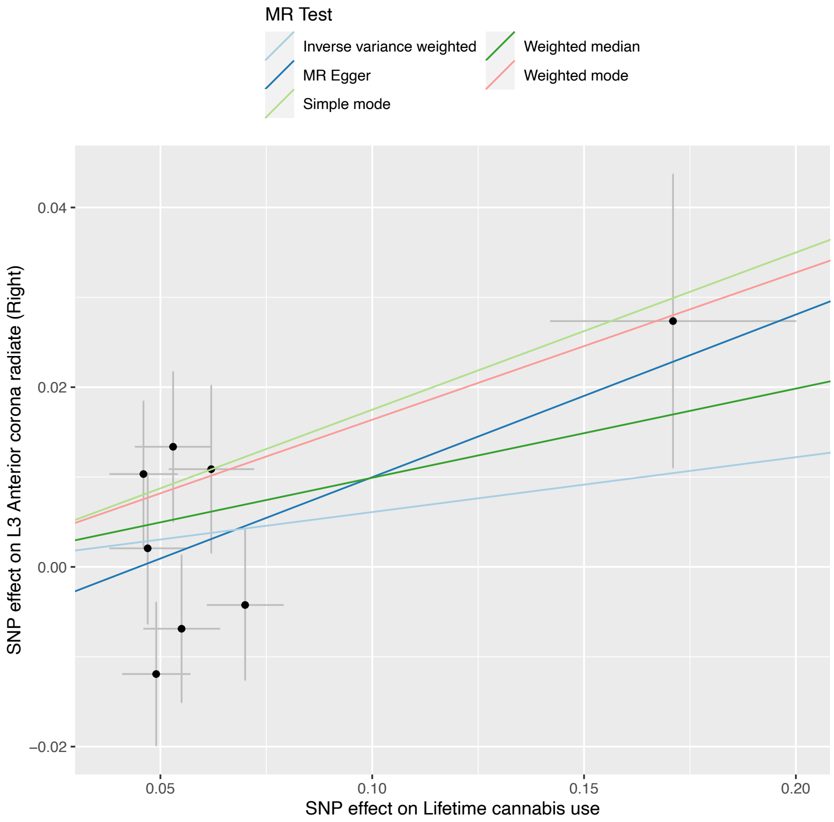
MR Test
Inverse variance weighted
MR Egger
Simple mode
Weighted median
Weighted mode
0.04
0.02
0.00
−0.02
SNP effect on L3 Anterior corona radiate (Right)
0.05
0.10
0.15
0.20
SNP effect on Lifetime cannabis use

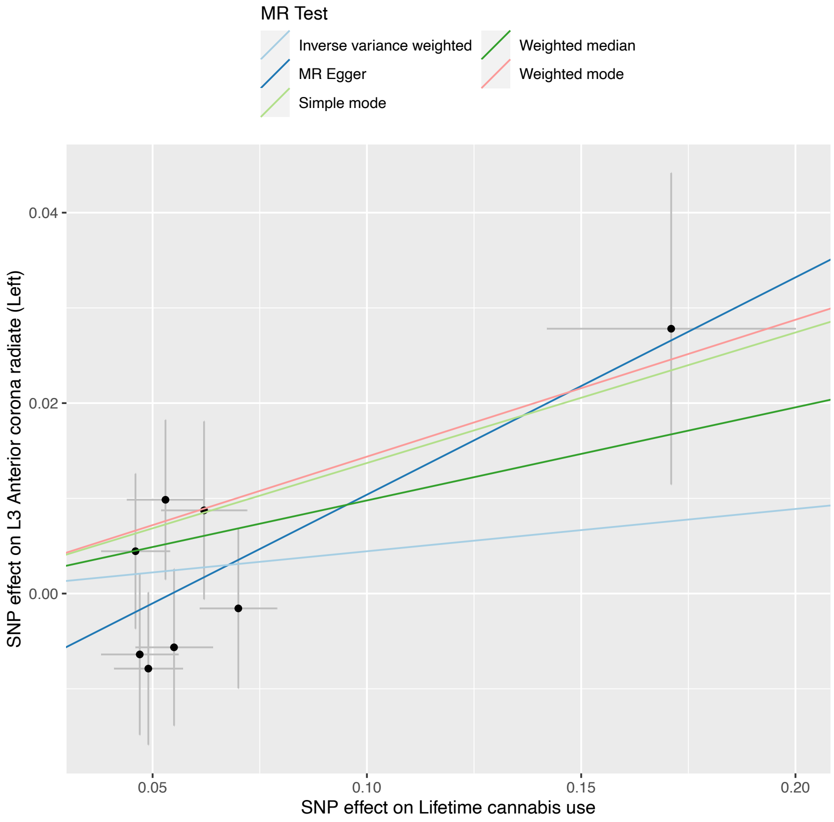
MR Test
Inverse variance weighted
MR Egger
Simple mode
Weighted median
Weighted mode
0.04
0.02
0.00
SNP effect on L3 Anterior corona radiate (Left)
0.05
0.10
0.15
0.20
SNP effect on Lifetime cannabis use

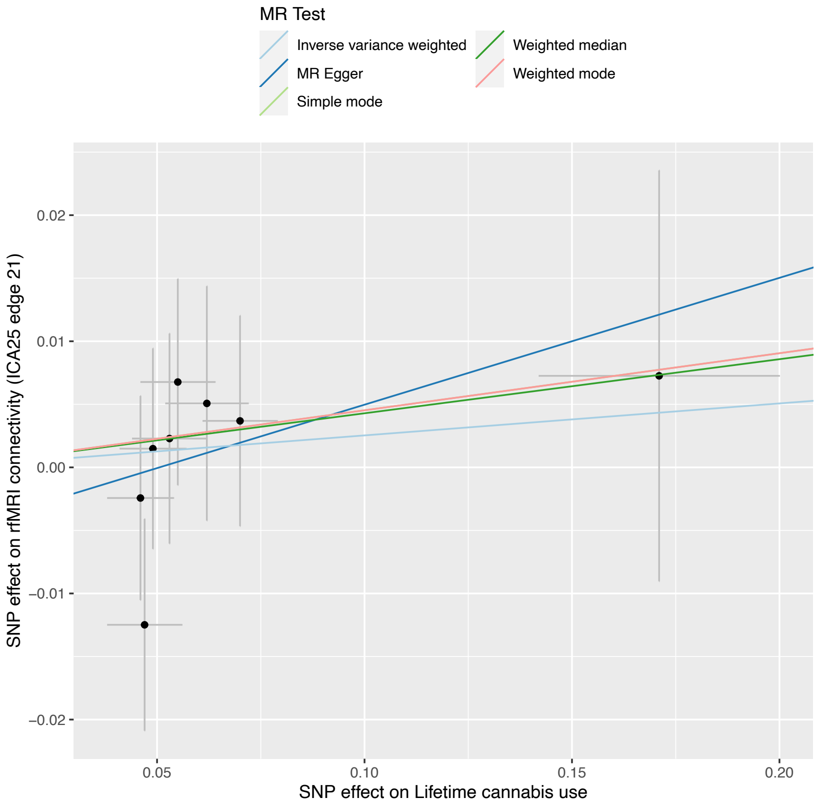
MR Test
Inverse variance weighted
MR Egger
Simple mode
Weighted median
Weighted mode
SNP effect on rfMRI connectivity (ICA25 edge 21)
0.02
0.01
0.00
−0.01
−0.02
0.05
0.10
0.15
0.20
SNP effect on Lifetime cannabis use

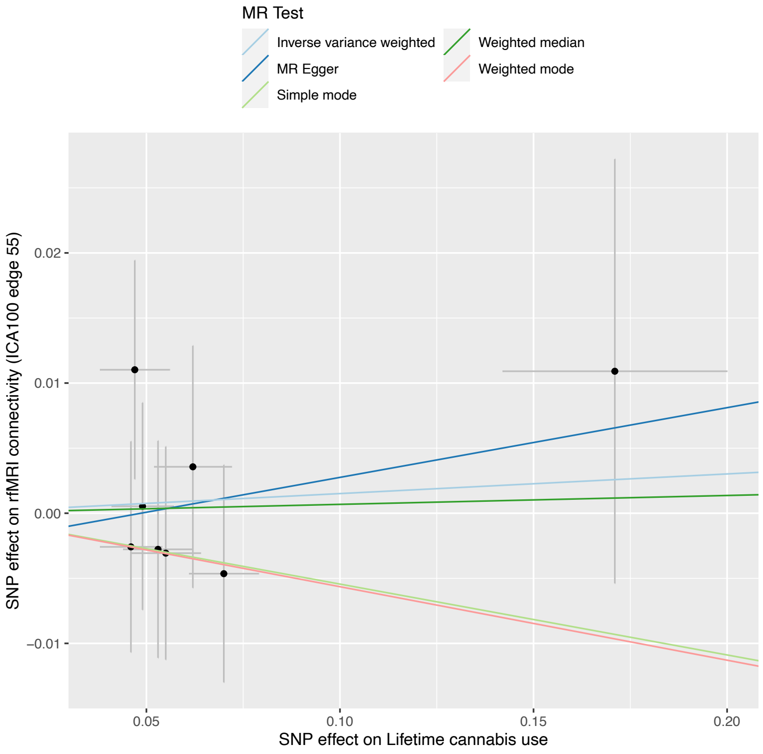
MR Test
Inverse variance weighted
MR Egger
Simple mode
Weighted median
Weighted mode
SNP effect on rfMRI connectivity (ICA100 edge 55)
0.02
0.01
0.00
−0.01
0.05
0.10
0.15
0.20
SNP effect on Lifetime cannabis use

## SFigure 3b: Reverse two-sample linear MR plot for the causal effect of brain measures on Cannabis dependence or abuse

### Brain measures and Lifetime Cannabis use

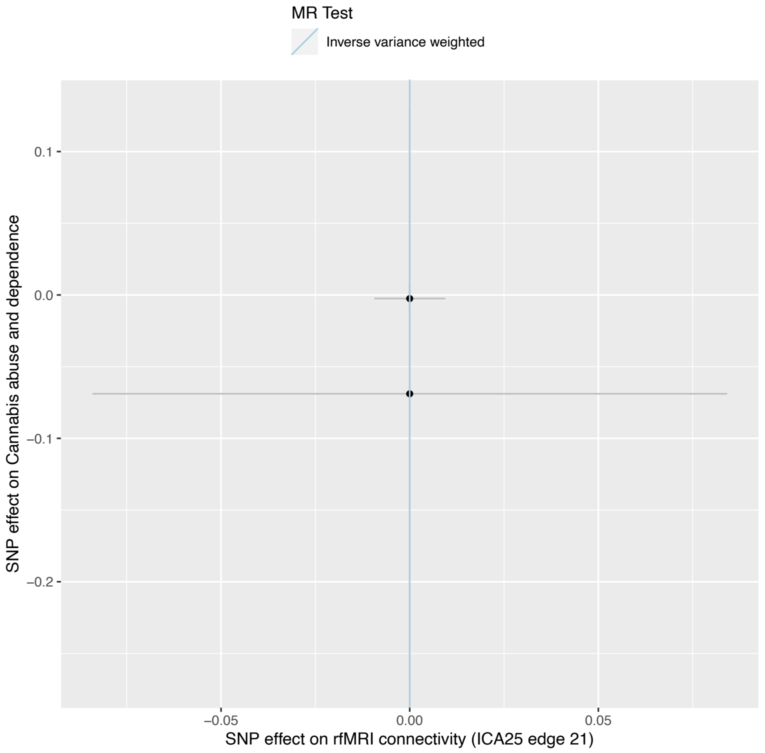


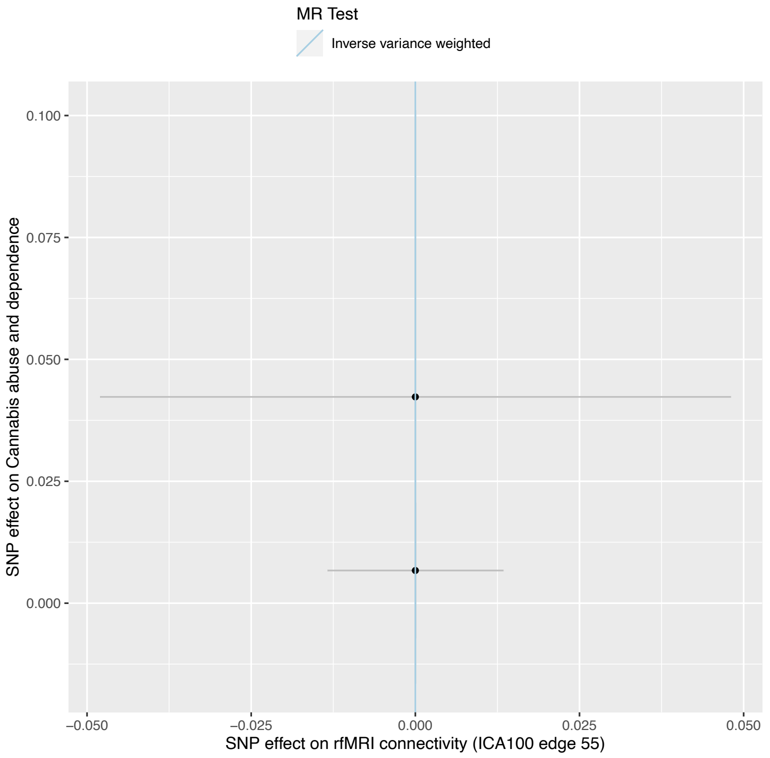

Supplement: online supplemental file 1 [file bmjment-27-1-s001.pdf]
