# Supplementary material for: Association between cannabis use and brain structure and function: an observational and Mendelian randomisation study
Source: BMJ Ment Health. 2024 Oct 30;27(1):e301065. doi: 10.1136/bmjment-2024-301065 (PMC11529520; doi:10.1136/bmjment-2024-301065)
Supplement: online supplemental file 2 [file bmjment-27-1-s002.pdf]

Supplementary methods

MRI acquisition and data processing

The UK Biobank team generated the brain imaging data which was accessible to researchers. The brain imaging data were preprocessed by a fully automated processing pipeline primarily centered around FSL software [1]. The imaging modalities within the UK Biobank comprised T1-weighted, T2-weighted flair, diffusion MRI (dMRI), susceptibility-weighted imaging (SWI), task functional MRI timeseries data (tfMRI), and resting-state functional MRI timeseries data (rsfMRI) [2].

T1-weighted MPRAGE and T2-weighted FLAIR volumes were obtained at 1×1×1mm (208×256×256 field of view [FOV] matrix) and 1.05×1×1mm (192×256×256 FOV matrix) respectively. Linear and non-linear registration to MNI152 "nonlinear 6th generation" standard-space was conducted using FMRIB's Linear Registration Tool (FLIRT) and FMRIB's Nonlinear Image Registration Tool (FNIRT) respectively. Additionally, brain extraction was performed using the Brain Extraction Tool (BET), followed by defacing and segmentation into tissue types using FMRIB's Automated Segmentation Tool (FAST). Grey matter was extracted from FAST and the estimation of the grey matter was performed for a total of 139 regions of interest (ROIs) defined by the HarvardOxford cortical and subcortical atlases and the Diedrichsen cerebellar atlas. Subcortical volumes were generated by using FMRIB's Integrated Registration and Segmentation Tool (FIRST) and estimation was done using the population priors on shape and intensity variation across subjects. Image segmentation was performed to identify white matter hyperintensities (WMH). Additionally, periventricular WMH (pWMH) and deep WMH (dWMH), were defined based on subsets of total WM hyperintensities.

DMRI were obtained at 2×2×2mm (104×104×72 FOV matrix) using a spin-echo echo-planar sequence with two b-values (b = 1000 and 2000 s/mm2). 50 diffusion encoding directions were acquired for each diffusion-weighted shell and the tensor fitting was performed using the b = 1000 s/mm2. The diffusion tensor and NODDI models were applied to pre-processed data, generating nine microstructural maps: fractional anisotropy (FA), mean diffusivity (MD), axial diffusivity (L1), radial diffusivities (L2, L3) and mode of anisotropy (MO) from DTI, and intracellular volume fraction (ICVF), isotropic volume fraction (ISOVF), and orientation dispersion (OD) from NODDI. Tract-Based Spatial Statistics (TBSS) processing was performed using these DTI maps and TBSS-derived measures were computed by averaging the skeletonized images of each DTI map within a predefined set of 48 standard-space tract masks defined by the JHU White Matter Atlas (ICBM-DTI-81) [3].

SWI was obtained at 0.8×0.8×3mm (256×288×48 FOV matrix). T2* and Quantitative susceptibility mapping (QSM) were used to generate the IDPs. First T1-weighted structural brain scan was used to derive subject-specific masks for 14 subcortical regions that correspond to the left and right of the 7 subcortical structure ROIs. Subsequently, image-derived phenotypes (IDPs) were calculated based on the median T2* and χ values for each of these regions.

TfMRI were obtained at 2.4×2.4×2.4mm (88×88×64 FOV matrix) involving Hariri faces/shapes "emotion" task with either angry or fearful faces. Participants were presented with blocks of trials where they were required to determine which of the two faces displayed at the bottom of the screen correspond to the face shown at the top, or which of the two shapes presented at the bottom match the shape displayed at the top. Preprocessing and modeling of task-induced activation were conducted using FEAT (FMRI Expert Analysis Tool), while time-series statistical analysis utilized FILM with local autocorrelation correction. The three contrasts of interest were: 1 (Shapes), 2 (Faces), and 5 (Faces-Shapes), with the last contrast was with respect to amygdala activation. Group-average activation maps were generated from analysis across all subjects and Regions of Interest (ROIs) were defined. Task fMRI IDPs represent summary measures of activation, including the median and 90th percentile for both the percent signal change and the z-statistic. These measures are computed from regions selected from the group-level activation map [4].

RsfMRI were also obtained as per tfMRI at 2.4×2.4×2.4mm (88×88×64 FOV matrix). On a preprocessed sample of 4162 participants, grouped average independent component analysis (ICA) was carried out using MELODIC [4, 5]. ICA was performed with dimensionality set to 25 and 100, which resulted in 21 and 55 components, respectively, after discarding the noise components. These 21×21 and 55×55 partial correlation matrices were used as measurements of functional connections. The ICA maps were mapped on each participant's rsfMRI timeseries data in order to acquire one representative node timeseries per ICA component for each subject. These network 'nodes' are a measure of within-network functional connectivity. Subject-specific network-matrices ('edges') were also extracted from the node timeseries that provide a measure of functional connectivity between the nodes [6].

Mendelian Randomization

Mendelian Randomization (MR) is based on three fundamental assumptions. A genetic variant is considered an instrumental variable for a specific exposure when it fulfils the following criteria:

1. It is associated with the exposure
2. It is not associated with the outcome due to confounding pathways

3. It does not affect the outcome except potentially via the exposure

Robust Methods MR

We performed two-sample MR analyses by using the TwoSampleMR in an R package [7]. For the SNPs significantly associated with cannabis dependence or abuse and lifetime cannabis use, five different MR methods were applied: inverse-variance weighted (IVW), MR Egger, weighted median, weighted mode, and simple mode.

- The IVW method was utilized because the instruments used in the study consisted of multiple SNPs. To ensure valid results, the IVW method requires that all instruments are associated with the exposure variable, but not directly associated with the outcome variable or any confounding factors affecting the relationship between the exposure and outcome.
- MR Egger tests for horizontal pleiotropy, which assumes that the pleiotropy effects are independent of the strength of the instrument used [8].
- The estimate obtained from the weighted median gives an estimate of the causal effect when at least half of the genetic variants used are valid instrumental variables.
- Weighted mode assumes that the validity of the instruments is based on the largest number of instruments with consistent MR estimates.
- Last, the simple mode approach uses the mode of the instrumental variable estimate to estimate the causal effect. In addition, we performed a reverse analysis to test for reverse causation.

References

1. Jenkinson, M., Beckmann, C. F., Behrens, T. E., Woolrich, M. W., & Smith, S. M. (2012). FSL. *NeuroImage*, *62*(2), 782–790. https://doi.org/10.1016/j.neuroimage.2011.09.015
2. Miller, K. L., Alfaro-Almagro, F., Bangerter, N. K., Thomas, D. L., Yacoub, E., Xu, J., Bartsch, A. J., Jbabdi, S., Sotiropoulos, S. N., Andersson, J. L., Griffanti, L., Douaud, G., Okell, T. W., Weale, P., Dragonu, I., Garratt, S., Hudson, S., Collins, R., Jenkinson, M., Matthews, P. M., … Smith, S. M. (2016). Multimodal population brain imaging in the UK Biobank prospective epidemiological study. *Nature neuroscience*, *19*(11), 1523–1536. https://doi.org/10.1038/nn.4393
3. (2006). MRI Atlas of Human White Matter. *AJNR: American Journal of Neuroradiology*, *27*(6), 1384-1385. https://www.ncbi.nlm.nih.gov/pmc/articles/PMC8133945/
4. Alfaro-Almagro, F. et al. Image processing and quality control for the first 10,000 brain imaging datasets from UK Biobank. *Neuroimage* **166**, 400–424 (2018).
5. Beckmann, C. F., & Smith, S. M. (2004). Probabilistic independent component analysis for functional magnetic resonance imaging. *IEEE*

transactions on medical imaging, *23*(2), 137–152. https://doi.org/10.1109/TMI.2003.822821

6. Smith, S. M., Vidaurre, D., Beckmann, C. F., Glasser, M. F., Jenkinson, M., Miller, K. L., Nichols, T. E., Robinson, E. C., Salimi-Khorshidi, G., Woolrich, M. W., Barch, D. M., Uğurbil, K., & Van Essen, D. C. (2013). Functional connectomics from resting-state fMRI. *Trends in cognitive sciences*, *17*(12), 666–682. https://doi.org/10.1016/j.tics.2013.09.016
7. Hemani, G., Zheng, J., Elsworth, B., Wade, K. H., Haberland, V., Baird, D., Laurin, C., Burgess, S., Bowden, J., Langdon, R., Tan, V. Y., Yarmolinsky, J., Shihab, H. A., Timpson, N. J., Evans, D. M., Relton, C., Martin, R. M., Smith, G. D., Gaunt, T. R., . . . Haycock, P. C. (2018). The MR-Base platform supports systematic causal inference across the human phenome. *ELife*, *7*. https://doi.org/10.7554/eLife.34408
8. Burgess, S., & Thompson, S. G. (2017). Interpreting findings from Mendelian randomization using the MR-Egger method. European Journal of Epidemiology, 32(5), 377-389. https://doi.org/10.1007/s10654-017-0255-x
