# Supplementary material for: Association between cannabis use and brain structure and function: an observational and Mendelian randomisation study
Source: BMJ Ment Health. 2024 Oct 30;27(1):e301065. doi: 10.1136/bmjment-2024-301065 (PMC11529520; doi:10.1136/bmjment-2024-301065)
Supplement: online supplemental file 3 [file bmjment-27-1-s003.pdf]

Supplementary tables

Table of Contents

STable 1: Individual image-derived phenotypes in UK Biobank (n= 3,921) 2

STable 2a: SNPs identified in GWAS associated with cannabis use 96

STable 2b: SNPs identified in GWAS associated with brain IDPs 97

STable 3: Brain imaging confounds in UK Biobank (n = 613)...98

STable 4a: Demographic characteristics (missing vs non missing cannabis data).................113

STable 4b: Demographic characteristics 114

STable 4c: Demographic characteristics (male and female)..115

STable 5: Association between cannabis use and hippocampus volume 116

STable 6: Associations between cannabis use and individual image-derived phenotypes.....117

STable 7: ID, location, and network of resting-state functional connectivity association with cannabis use...119

STable 8: Associations between cannabis use and brain measures surviving Bonferroni correction 121

STable 9: Associations between cannabis use and brain measures in males surviving False Discovery Rate 122

STable 10: Associations between cannabis use and brain measures in females surviving False Discovery Rate 123

STable 11a: Two-sample linear MR estimates for the causal effect of cannabis use on brain IDPs...124

STable 11b: Two-sample linear Reverse MR estimates for the causal effect of brain IDPs on cannabis use..126

STable 1: Individual image-derived phenotypes in UK Biobank (n= 3,921)

1)	IDP_T1_SIENAX_peripheral_grey_normalised_volume
2)	IDP_T1_SIENAX_peripheral_grey_unnormalised_volume
3)	IDP_T1_SIENAX_CSF_normalised_volume
4)	IDP_T1_SIENAX_CSF_unnormalised_volume
5)	IDP_T1_SIENAX_grey_normalised_volume
6)	IDP_T1_SIENAX_grey_unnormalised_volume
7)	IDP_T1_SIENAX_white_normalised_volume
8)	IDP_T1_SIENAX_white_unnormalised_volume
9)	IDP_T1_SIENAX_brain-normalised_volume
10)	IDP_T1_SIENAX_brain-unnormalised_volume
11)	IDP_T1_FIRST_left_thalamus_volume
12)	IDP_T1_FIRST_right_thalamus_volume
13)	IDP_T1_FIRST_left_caudate_volume
14)	IDP_T1_FIRST_right_caudate_volume
15)	IDP_T1_FIRST_left_putamen_volume
16)	IDP_T1_FIRST_right_putamen_volume
17)	IDP_T1_FIRST_left_pallidum_volume
18)	IDP_T1_FIRST_right_pallidum_volume
19)	IDP_T1_FIRST_left_hippocampus_volume
20)	IDP_T1_FIRST_right_hippocampus_volume
21)	IDP_T1_FIRST_left_amygdala_volume
22)	IDP_T1_FIRST_right_amygdala_volume
23)	IDP_T1_FIRST_left_accumbens_volume
24)	IDP_T1_FIRST_right_accumbens_volume
25)	IDP_T1_FIRST_brain_stem+4th_ventricle_volume
26)	IDP_T1_FAST_ROIs_L_frontal_pole
27)	IDP_T1_FAST_ROIs_R_frontal_pole
28)	IDP_T1_FAST_ROIs_L_insular_cortex
29)	IDP_T1_FAST_ROIs_R_insular_cortex
30)	IDP_T1_FAST_ROIs_L_sup_front_gyrus
31)	IDP_T1_FAST_ROIs_R_sup_front_gyrus
32)	IDP_T1_FAST_ROIs_L_mid_front_gyrus
33)	IDP_T1_FAST_ROIs_R_mid_front_gyrus
34)	IDP_T1_FAST_ROIs_L_inf_front_gyrus_parstri
35)	IDP_T1_FAST_ROIs_R_inf_front_gyrus_parstri
36)	IDP_T1_FAST_ROIs_L_inf_front_gyrus_parsop
37)	IDP_T1_FAST_ROIs_R_inf_front_gyrus_parsop
38)	IDP_T1_FAST_ROIs_L_precentral_gyrus
39)	IDP_T1_FAST_ROIs_R_precentral_gyrus
40)	IDP_T1_FAST_ROIs_L_temporal_pole

41)	IDP_T1_FAST_ROIs_R_temporal_pole
42)	IDP_T1_FAST_ROIs_L_sup_temp_gyrus_ant
43)	IDP_T1_FAST_ROIs_R_sup_temp_gyrus_ant
44)	IDP_T1_FAST_ROIs_L_sup_temp_gyrus_post
45)	IDP_T1_FAST_ROIs_R_sup_temp_gyrus_post
46)	IDP_T1_FAST_ROIs_L_mid_temp_gyrus_ant
47)	IDP_T1_FAST_ROIs_R_mid_temp_gyrus_ant
48)	IDP_T1_FAST_ROIs_L_mid_temp_gyrus_post
49)	IDP_T1_FAST_ROIs_R_mid_temp_gyrus_post
50)	IDP_T1_FAST_ROIs_L_mid_temp_gyrus_tempocc
51)	IDP_T1_FAST_ROIs_R_mid_temp_gyrus_tempocc
52)	IDP_T1_FAST_ROIs_L_inf_temp_gyrus_ant
53)	IDP_T1_FAST_ROIs_R_inf_temp_gyrus_ant
54)	IDP_T1_FAST_ROIs_L_inf_temp_gyrus_post
55)	IDP_T1_FAST_ROIs_R_inf_temp_gyrus_post
56)	IDP_T1_FAST_ROIs_L_inf_temp_gyrus_tempocc
57)	IDP_T1_FAST_ROIs_R_inf_temp_gyrus_tempocc
58)	IDP_T1_FAST_ROIs_L_postcent_gyrus
59)	IDP_T1_FAST_ROIs_R_postcent_gyrus
60)	IDP_T1_FAST_ROIs_L_sup_parietal_lobule
61)	IDP_T1_FAST_ROIs_R_sup_parietal_lobule
62)	IDP_T1_FAST_ROIs_L_supramarg_gyrus_ant
63)	IDP_T1_FAST_ROIs_R_supramarg_gyrus_ant
64)	IDP_T1_FAST_ROIs_L_supramarg_gyrus_post
65)	IDP_T1_FAST_ROIs_R_supramarg_gyrus_post
66)	IDP_T1_FAST_ROIs_L_angular_gyrus
67)	IDP_T1_FAST_ROIs_R_angular_gyrus
68)	IDP_T1_FAST_ROIs_L_latocc_cortex_sup
69)	IDP_T1_FAST_ROIs_R_latocc_cortex_sup
70)	IDP_T1_FAST_ROIs_L_latocc_cortex_inf
71)	IDP_T1_FAST_ROIs_R_latocc_cortex_inf
72)	IDP_T1_FAST_ROIs_L_intracalc_cortex
73)	IDP_T1_FAST_ROIs_R_intracalc_cortex
74)	IDP_T1_FAST_ROIs_L_front_med_cortex
75)	IDP_T1_FAST_ROIs_R_front_med_cortex
76)	IDP_T1_FAST_ROIs_L_juxtapos_lobule_cortex
77)	IDP_T1_FAST_ROIs_R_juxtapos_lobule_cortex
78)	IDP_T1_FAST_ROIs_L_subcallosal_cortex
79)	IDP_T1_FAST_ROIs_R_subcallosal_cortex
80)	IDP_T1_FAST_ROIs_L_paracing_gyrus
81)	IDP_T1_FAST_ROIs_R_paracing_gyrus
82)	IDP_T1_FAST_ROIs_L_cing_gyrus_ant

83)	IDP_T1_FAST_ROIs_R_cing_gyrus_ant
84)	IDP_T1_FAST_ROIs_L_cing_gyrus_post
85)	IDP_T1_FAST_ROIs_R_cing_gyrus_post
86)	IDP_T1_FAST_ROIs_L_precun_cortex
87)	IDP_T1_FAST_ROIs_R_precun_cortex
88)	IDP_T1_FAST_ROIs_L_cuneal_cortex
89)	IDP_T1_FAST_ROIs_R_cuneal_cortex
90)	IDP_T1_FAST_ROIs_L_front_orb_cortex
91)	IDP_T1_FAST_ROIs_R_front_orb_cortex
92)	IDP_T1_FAST_ROIs_L_parahipp_gyrus_ant
93)	IDP_T1_FAST_ROIs_R_parahipp_gyrus_ant
94)	IDP_T1_FAST_ROIs_L_parahipp_gyrus_post
95)	IDP_T1_FAST_ROIs_R_parahipp_gyrus_post
96)	IDP_T1_FAST_ROIs_L_lingual_gyrus
97)	IDP_T1_FAST_ROIs_R_lingual_gyrus
98)	IDP_T1_FAST_ROIs_L_temp_fusif_cortex_ant
99)	IDP_T1_FAST_ROIs_R_temp_fusif_cortex_ant
100)	IDP_T1_FAST_ROIs_L_temp_fusif_cortex_post
101)	IDP_T1_FAST_ROIs_R_temp_fusif_cortex_post
102)	IDP_T1_FAST_ROIs_L_temp_occ_fusif_cortex
103)	IDP_T1_FAST_ROIs_R_temp_occ_fusif_cortex
104)	IDP_T1_FAST_ROIs_L_occ_fusif_gyrus
105)	IDP_T1_FAST_ROIs_R_occ_fusif_gyrus
106)	IDP_T1_FAST_ROIs_L_front_operc_cortex
107)	IDP_T1_FAST_ROIs_R_front_operc_cortex
108)	IDP_T1_FAST_ROIs_L_cent_operc_cortex
109)	IDP_T1_FAST_ROIs_R_cent_operc_cortex
110)	IDP_T1_FAST_ROIs_L_parietal_operc_cortex
111)	IDP_T1_FAST_ROIs_R_parietal_operc_cortex
112)	IDP_T1_FAST_ROIs_L_planum_polare
113)	IDP_T1_FAST_ROIs_R_planum_polare
114)	IDP_T1_FAST_ROIs_L_heschl_gyrus
115)	IDP_T1_FAST_ROIs_R_heschl_gyrus
116)	IDP_T1_FAST_ROIs_L_planum_temporale
117)	IDP_T1_FAST_ROIs_R_planum_temporale
118)	IDP_T1_FAST_ROIs_L_supracalc_cortex
119)	IDP_T1_FAST_ROIs_R_supracalc_cortex
120)	IDP_T1_FAST_ROIs_L_occ_pole
121)	IDP_T1_FAST_ROIs_R_occ_pole
122)	IDP_T1_FAST_ROIs_L_thalamus
123)	IDP_T1_FAST_ROIs_R_thalamus
124)	IDP_T1_FAST_ROIs_L_caudate

125)	IDP_T1_FAST_ROIs_R_caudate
126)	IDP_T1_FAST_ROIs_L_putamen
127)	IDP_T1_FAST_ROIs_R_putamen
128)	IDP_T1_FAST_ROIs_L_pallidum
129)	IDP_T1_FAST_ROIs_R_pallidum
130)	IDP_T1_FAST_ROIs_L_hippocampus
131)	IDP_T1_FAST_ROIs_R_hippocampus
132)	IDP_T1_FAST_ROIs_L_amygdala
133)	IDP_T1_FAST_ROIs_R_amygdala
134)	IDP_T1_FAST_ROIs_L_ventral_striatum
135)	IDP_T1_FAST_ROIs_R_ventral_striatum
136)	IDP_T1_FAST_ROIs_brain_stem
137)	IDP_T1_FAST_ROIs_L_cerebellum_I-IV
138)	IDP_T1_FAST_ROIs_R_cerebellum_I-IV
139)	IDP_T1_FAST_ROIs_L_cerebellum_V
140)	IDP_T1_FAST_ROIs_R_cerebellum_V
141)	IDP_T1_FAST_ROIs_L_cerebellum_VI
142)	IDP_T1_FAST_ROIs_V_cerebellum_VI
143)	IDP_T1_FAST_ROIs_R_cerebellum_VI
144)	IDP_T1_FAST_ROIs_L_cerebellum_crus_I
145)	IDP_T1_FAST_ROIs_V_cerebellum_crus_I
146)	IDP_T1_FAST_ROIs_R_cerebellum_crus_I
147)	IDP_T1_FAST_ROIs_L_cerebellum_crus_II
148)	IDP_T1_FAST_ROIs_V_cerebellum_crus_II
149)	IDP_T1_FAST_ROIs_R_cerebellum_crus_II
150)	IDP_T1_FAST_ROIs_L_cerebellum_VIIb
151)	IDP_T1_FAST_ROIs_V_cerebellum_VIIb
152)	IDP_T1_FAST_ROIs_R_cerebellum_VIIb
153)	IDP_T1_FAST_ROIs_L_cerebellum_VIIIa
154)	IDP_T1_FAST_ROIs_V_cerebellum_VIIIa
155)	IDP_T1_FAST_ROIs_R_cerebellum_VIIIa
156)	IDP_T1_FAST_ROIs_L_cerebellum_VIIIb
157)	IDP_T1_FAST_ROIs_V_cerebellum_VIIIb
158)	IDP_T1_FAST_ROIs_R_cerebellum_VIIIb
159)	IDP_T1_FAST_ROIs_L_cerebellum_IX
160)	IDP_T1_FAST_ROIs_V_cerebellum_IX
161)	IDP_T1_FAST_ROIs_R_cerebellum_IX
162)	IDP_T1_FAST_ROIs_L_cerebellum_X
163)	IDP_T1_FAST_ROIs_V_cerebellum_X
164)	IDP_T1_FAST_ROIs_R_cerebellum_X
165)	aseg_global_volume_BrainSeg
166)	aseg_global_volume_BrainSegNotVent

167)	aseg_global_volume_BrainSegNotVentSurf
168)	aseg_global_volume_SubCortGray
169)	aseg_global_volume_TotalGray
170)	aseg_global_volume_SupraTentorial
171)	aseg_global_volume_SupraTentorialNotVent
172)	aseg_global_volume_EstimatedTotalIntraCranial
173)	aseg_global_volume_VentricleChoroid
174)	aseg_global_volume_3rd-Ventricle
175)	aseg_global_volume_4th-Ventricle
176)	aseg_global_volume_5th-Ventricle
177)	aseg_global_volume_Brain-Stem
178)	aseg_global_volume_CSF
179)	aseg_global_volume_WM-hypointensities
180)	aseg_global_volume_non-WM-hypointensities
181)	aseg_global_volume_Optic-Chiasm
182)	aseg_global_volume_CC-Posterior
183)	aseg_global_volume_CC-Mid-Posterior
184)	aseg_global_volume_CC-Central
185)	aseg_global_volume_CC-Mid-Anterior
186)	aseg_global_volume_CC-Anterior
187)	aseg_global_volume-ratio_BrainSegVol-to-eTIV
188)	aseg_global_volume-ratio_MaskVol-to-eTIV
189)	aseg_lh_volume_Cortex
190)	aseg_lh_volume_CerebralWhiteMatter
191)	aseg_lh_volume_Lateral-Ventricle
192)	aseg_lh_volume_Inf-Lat-Vent
193)	aseg_lh_volume_Cerebellum-White-Matter
194)	aseg_lh_volume_Cerebellum-Cortex
195)	aseg_lh_volume_Thalamus-Proper
196)	aseg_lh_volume_Caudate
197)	aseg_lh_volume_Putamen
198)	aseg_lh_volume_Pallidum
199)	aseg_lh_volume_Hippocampus
200)	aseg_lh_volume_Amygdala
201)	aseg_lh_volume_Accumbens-area
202)	aseg_lh_volume_VentralDC
203)	aseg_lh_volume_vessel
204)	aseg_lh_volume_choroid-plexus
205)	aseg_lh_number_HolesBeforeFixing
206)	aseg_rh_volume_Cortex
207)	aseg_rh_volume_CerebralWhiteMatter
208)	aseg_rh_volume_Lateral-Ventricle

209)	aseg_rh_volume_Inf-Lat-Vent
210)	aseg_rh_volume_Cerebellum-White-Matter
211)	aseg_rh_volume_Cerebellum-Cortex
212)	aseg_rh_volume_Thalamus-Proper
213)	aseg_rh_volume_Caudate
214)	aseg_rh_volume_Putamen
215)	aseg_rh_volume_Pallidum
216)	aseg_rh_volume_Hippocampus
217)	aseg_rh_volume_Amygdala
218)	aseg_rh_volume_Accumbens-area
219)	aseg_rh_volume_VentralDC
220)	aseg_rh_volume_vessel
221)	aseg_rh_volume_choroid-plexus
222)	aseg_rh_number_HolesBeforeFixing
223)	AmygNuclei_lh_volume_Lateral-nucleus
224)	AmygNuclei_lh_volume_Basal-nucleus
225)	AmygNuclei_lh_volume_Accessory-Basal-nucleus
226)	AmygNuclei_lh_volume_Anterior-amygdaloid-area-AAA
227)	AmygNuclei_lh_volume_Central-nucleus
228)	AmygNuclei_lh_volume_Medial-nucleus
229)	AmygNuclei_lh_volume_Cortical-nucleus
230)	AmygNuclei_lh_volume_Corticoamygdaloid-transitio
231)	AmygNuclei_lh_volume_Paralaminar-nucleus
232)	AmygNuclei_lh_volume_Whole-amygdala
233)	AmygNuclei_rh_volume_Lateral-nucleus
234)	AmygNuclei_rh_volume_Basal-nucleus
235)	AmygNuclei_rh_volume_Accessory-Basal-nucleus
236)	AmygNuclei_rh_volume_Anterior-amygdaloid-area-AAA
237)	AmygNuclei_rh_volume_Central-nucleus
238)	AmygNuclei_rh_volume_Medial-nucleus
239)	AmygNuclei_rh_volume_Cortical-nucleus
240)	AmygNuclei_rh_volume_Corticoamygdaloid-transitio
241)	AmygNuclei_rh_volume_Paralaminar-nucleus
242)	AmygNuclei_rh_volume_Whole-amygdala
243)	HippSubfield_lh_volume_Hippocampal-tail
244)	HippSubfield_lh_volume_subiculum-body
245)	HippSubfield_lh_volume_CA1-body
246)	HippSubfield_lh_volume_subiculum-head
247)	HippSubfield_lh_volume_hippocampal-fissure
248)	HippSubfield_lh_volume_presubiculum-head
249)	HippSubfield_lh_volume_CA1-head
250)	HippSubfield_lh_volume_presubiculum-body

251)	HippSubfield_lh_volume_parasubiculum
252)	HippSubfield_lh_volume_molecular-layer-HP-head
253)	HippSubfield_lh_volume_molecular-layer-HP-body
254)	HippSubfield_lh_volume_GC-ML-DG-head
255)	HippSubfield_lh_volume_CA3-body
256)	HippSubfield_lh_volume_GC-ML-DG-body
257)	HippSubfield_lh_volume_CA4-head
258)	HippSubfield_lh_volume_CA4-body
259)	HippSubfield_lh_volume_fimbria
260)	HippSubfield_lh_volume_CA3-head
261)	HippSubfield_lh_volume_HATA
262)	HippSubfield_lh_volume_Whole-hippocampal-body
263)	HippSubfield_lh_volume_Whole-hippocampal-head
264)	HippSubfield_lh_volume_Whole-hippocampus
265)	HippSubfield_rh_volume_Hippocampal-tail
266)	HippSubfield_rh_volume_subiculum-body
267)	HippSubfield_rh_volume_CA1-body
268)	HippSubfield_rh_volume_subiculum-head
269)	HippSubfield_rh_volume_hippocampal-fissure
270)	HippSubfield_rh_volume_presubiculum-head
271)	HippSubfield_rh_volume_CA1-head
272)	HippSubfield_rh_volume_presubiculum-body
273)	HippSubfield_rh_volume_parasubiculum
274)	HippSubfield_rh_volume_molecular-layer-HP-head
275)	HippSubfield_rh_volume_molecular-layer-HP-body
276)	HippSubfield_rh_volume_GC-ML-DG-head
277)	HippSubfield_rh_volume_CA3-body
278)	HippSubfield_rh_volume_GC-ML-DG-body
279)	HippSubfield_rh_volume_CA4-head
280)	HippSubfield_rh_volume_CA4-body
281)	HippSubfield_rh_volume_fimbria
282)	HippSubfield_rh_volume_CA3-head
283)	HippSubfield_rh_volume_HATA
284)	HippSubfield_rh_volume_Whole-hippocampal-body
285)	HippSubfield_rh_volume_Whole-hippocampal-head
286)	HippSubfield_rh_volume_Whole-hippocampus
287)	ThalamNuclei_lh_volume_MGN
288)	ThalamNuclei_lh_volume_LGN
289)	ThalamNuclei_lh_volume_PuI
290)	ThalamNuclei_lh_volume_PuM
291)	ThalamNuclei_lh_volume_L-Sg
292)	ThalamNuclei_lh_volume_VPL

293)	ThalamNuclei_lh_volume_CM
294)	ThalamNuclei_lh_volume_VLa
295)	ThalamNuclei_lh_volume_PuA
296)	ThalamNuclei_lh_volume_MDm
297)	ThalamNuclei_lh_volume_Pf
298)	ThalamNuclei_lh_volume_VAmc
299)	ThalamNuclei_lh_volume_MDl
300)	ThalamNuclei_lh_volume_CeM
301)	ThalamNuclei_lh_volume_VA
302)	ThalamNuclei_lh_volume_MV(Re)
303)	ThalamNuclei_lh_volume_VM
304)	ThalamNuclei_lh_volume_CL
305)	ThalamNuclei_lh_volume_PuL
306)	ThalamNuclei_lh_volume_Pt
307)	ThalamNuclei_lh_volume_AV
308)	ThalamNuclei_lh_volume_Pc
309)	ThalamNuclei_lh_volume_VLp
310)	ThalamNuclei_lh_volume_LP
311)	ThalamNuclei_rh_volume_LGN
312)	ThalamNuclei_rh_volume_MGN
313)	ThalamNuclei_rh_volume_PuI
314)	ThalamNuclei_rh_volume_PuM
315)	ThalamNuclei_rh_volume_L-Sg
316)	ThalamNuclei_rh_volume_VPL
317)	ThalamNuclei_rh_volume_CM
318)	ThalamNuclei_rh_volume_VLa
319)	ThalamNuclei_rh_volume_PuA
320)	ThalamNuclei_rh_volume_MDm
321)	ThalamNuclei_rh_volume_Pf
322)	ThalamNuclei_rh_volume_VAmc
323)	ThalamNuclei_rh_volume_MDl
324)	ThalamNuclei_rh_volume_VA
325)	ThalamNuclei_rh_volume_MV(Re)
326)	ThalamNuclei_rh_volume_CeM
327)	ThalamNuclei_rh_volume_VM
328)	ThalamNuclei_rh_volume_PuL
329)	ThalamNuclei_rh_volume_CL
330)	ThalamNuclei_rh_volume_VLp
331)	ThalamNuclei_rh_volume_Pc
332)	ThalamNuclei_rh_volume_Pt
333)	ThalamNuclei_rh_volume_AV
334)	ThalamNuclei_rh_volume_LP

335)	ThalamNuclei_lh_volume_LD
336)	ThalamNuclei_rh_volume_LD
337)	ThalamNuclei_lh_volume_Whole-thalamus
338)	ThalamNuclei_rh_volume_Whole-thalamus
339)	Brainstem_global_volume_Medulla
340)	Brainstem_global_volume_Pons
341)	Brainstem_global_volume_SCP
342)	Brainstem_global_volume_Midbrain
343)	Brainstem_global_volume_Whole-brainstem
344)	aparc-Desikan_lh_volume_bankssts
345)	aparc-Desikan_lh_volume_caudalanteriorcingulate
346)	aparc-Desikan_lh_volume_caudalmiddlefrontal
347)	aparc-Desikan_lh_volume_cuneus
348)	aparc-Desikan_lh_volume_entorhinal
349)	aparc-Desikan_lh_volume_fusiform
350)	aparc-Desikan_lh_volume_inferiorparietal
351)	aparc-Desikan_lh_volume_inferiortemporal
352)	aparc-Desikan_lh_volume_isthmuscingulate
353)	aparc-Desikan_lh_volume_lateraloccipital
354)	aparc-Desikan_lh_volume_lateralorbitofrontal
355)	aparc-Desikan_lh_volume_lingual
356)	aparc-Desikan_lh_volume_medialorbitofrontal
357)	aparc-Desikan_lh_volume_middletemporal
358)	aparc-Desikan_lh_volume_parahippocampal
359)	aparc-Desikan_lh_volume_paracentral
360)	aparc-Desikan_lh_volume_parsopercularis
361)	aparc-Desikan_lh_volume_parsorbitalis
362)	aparc-Desikan_lh_volume_parstriangularis
363)	aparc-Desikan_lh_volume_pericalcarine
364)	aparc-Desikan_lh_volume_postcentral
365)	aparc-Desikan_lh_volume_posteriorcingulate
366)	aparc-Desikan_lh_volume_precentral
367)	aparc-Desikan_lh_volume_precuneus
368)	aparc-Desikan_lh_volume_rostralanteriorcingulate
369)	aparc-Desikan_lh_volume_rostralmiddlefrontal
370)	aparc-Desikan_lh_volume_superiorfrontal
371)	aparc-Desikan_lh_volume_superiorparietal
372)	aparc-Desikan_lh_volume_superiortemporal
373)	aparc-Desikan_lh_volume_supramarginal
374)	aparc-Desikan_lh_volume_frontalpole
375)	aparc-Desikan_lh_volume_transversetemporal
376)	aparc-Desikan_lh_volume_insula

377)	aparc-Desikan_rh_volume_bankssts
378)	aparc-Desikan_rh_volume_caudalanteriorcingulate
379)	aparc-Desikan_rh_volume_caudalmiddlefrontal
380)	aparc-Desikan_rh_volume_cuneus
381)	aparc-Desikan_rh_volume_entorhinal
382)	aparc-Desikan_rh_volume_fusiform
383)	aparc-Desikan_rh_volume_inferiorparietal
384)	aparc-Desikan_rh_volume_inferiortemporal
385)	aparc-Desikan_rh_volume_isthmuscingulate
386)	aparc-Desikan_rh_volume_lateraloccipital
387)	aparc-Desikan_rh_volume_lateralorbitofrontal
388)	aparc-Desikan_rh_volume_lingual
389)	aparc-Desikan_rh_volume_medialorbitofrontal
390)	aparc-Desikan_rh_volume_middletemporal
391)	aparc-Desikan_rh_volume_parahippocampal
392)	aparc-Desikan_rh_volume_paracentral
393)	aparc-Desikan_rh_volume_parsopercularis
394)	aparc-Desikan_rh_volume_parsorbitalis
395)	aparc-Desikan_rh_volume_parstriangularis
396)	aparc-Desikan_rh_volume_pericalcarine
397)	aparc-Desikan_rh_volume_postcentral
398)	aparc-Desikan_rh_volume_posteriorcingulate
399)	aparc-Desikan_rh_volume_precentral
400)	aparc-Desikan_rh_volume_precuneus
401)	aparc-Desikan_rh_volume_rostralanteriorcingulate
402)	aparc-Desikan_rh_volume_rostralmiddlefrontal
403)	aparc-Desikan_rh_volume_superiorfrontal
404)	aparc-Desikan_rh_volume_superiorparietal
405)	aparc-Desikan_rh_volume_superiortemporal
406)	aparc-Desikan_rh_volume_supramarginal
407)	aparc-Desikan_rh_volume_frontalpole
408)	aparc-Desikan_rh_volume_transversetemporal
409)	aparc-Desikan_rh_volume_insula
410)	BA-exvivo_lh_volume_BA1
411)	BA-exvivo_lh_volume_BA2
412)	BA-exvivo_lh_volume_BA3a
413)	BA-exvivo_lh_volume_BA3b
414)	BA-exvivo_lh_volume_BA4a
415)	BA-exvivo_lh_volume_BA4p
416)	BA-exvivo_lh_volume_BA6
417)	BA-exvivo_lh_volume_BA44
418)	BA-exvivo_lh_volume_BA45

419)	BA-exvivo_lh_volume_V1
420)	BA-exvivo_lh_volume_V2
421)	BA-exvivo_lh_volume_MT
422)	BA-exvivo_lh_volume_perirhinal
423)	BA-exvivo_lh_volume_entorhinal
424)	BA-exvivo_rh_volume_BA1
425)	BA-exvivo_rh_volume_BA2
426)	BA-exvivo_rh_volume_BA3a
427)	BA-exvivo_rh_volume_BA3b
428)	BA-exvivo_rh_volume_BA4a
429)	BA-exvivo_rh_volume_BA4p
430)	BA-exvivo_rh_volume_BA6
431)	BA-exvivo_rh_volume_BA44
432)	BA-exvivo_rh_volume_BA45
433)	BA-exvivo_rh_volume_V1
434)	BA-exvivo_rh_volume_V2
435)	BA-exvivo_rh_volume_MT
436)	BA-exvivo_rh_volume_perirhinal
437)	BA-exvivo_rh_volume_entorhinal
438)	aparc-DKTatlas_lh_volume_caudalanteriorcingulate
439)	aparc-DKTatlas_lh_volume_caudalmiddlefrontal
440)	aparc-DKTatlas_lh_volume_cuneus
441)	aparc-DKTatlas_lh_volume_entorhinal
442)	aparc-DKTatlas_lh_volume_fusiform
443)	aparc-DKTatlas_lh_volume_inferiorparietal
444)	aparc-DKTatlas_lh_volume_inferiortemporal
445)	aparc-DKTatlas_lh_volume_isthmuscingulate
446)	aparc-DKTatlas_lh_volume_lateraloccipital
447)	aparc-DKTatlas_lh_volume_lateralorbitofrontal
448)	aparc-DKTatlas_lh_volume_lingual
449)	aparc-DKTatlas_lh_volume_medialorbitofrontal
450)	aparc-DKTatlas_lh_volume_middletemporal
451)	aparc-DKTatlas_lh_volume_parahippocampal
452)	aparc-DKTatlas_lh_volume_paracentral
453)	aparc-DKTatlas_lh_volume_parsopercularis
454)	aparc-DKTatlas_lh_volume_parsorbitalis
455)	aparc-DKTatlas_lh_volume_parstriangularis
456)	aparc-DKTatlas_lh_volume_pericalcarine
457)	aparc-DKTatlas_lh_volume_postcentral
458)	aparc-DKTatlas_lh_volume_posteriorcingulate
459)	aparc-DKTatlas_lh_volume_precentral
460)	aparc-DKTatlas_lh_volume_precuneus

461)	aparc-DKTatlas_lh_volume_rostralanteriorcingulate
462)	aparc-DKTatlas_lh_volume_rostralmiddlefrontal
463)	aparc-DKTatlas_lh_volume_superiorfrontal
464)	aparc-DKTatlas_lh_volume_superiorparietal
465)	aparc-DKTatlas_lh_volume_superiortemporal
466)	aparc-DKTatlas_lh_volume_supramarginal
467)	aparc-DKTatlas_lh_volume_transversetemporal
468)	aparc-DKTatlas_lh_volume_insula
469)	aparc-DKTatlas_rh_volume_caudalanteriorcingulate
470)	aparc-DKTatlas_rh_volume_caudalmiddlefrontal
471)	aparc-DKTatlas_rh_volume_cuneus
472)	aparc-DKTatlas_rh_volume_entorhinal
473)	aparc-DKTatlas_rh_volume_fusiform
474)	aparc-DKTatlas_rh_volume_inferiorparietal
475)	aparc-DKTatlas_rh_volume_inferiortemporal
476)	aparc-DKTatlas_rh_volume_isthmuscingulate
477)	aparc-DKTatlas_rh_volume_lateraloccipital
478)	aparc-DKTatlas_rh_volume_lateralorbitofrontal
479)	aparc-DKTatlas_rh_volume_lingual
480)	aparc-DKTatlas_rh_volume_medialorbitofrontal
481)	aparc-DKTatlas_rh_volume_middletemporal
482)	aparc-DKTatlas_rh_volume_parahippocampal
483)	aparc-DKTatlas_rh_volume_paracentral
484)	aparc-DKTatlas_rh_volume_parsopercularis
485)	aparc-DKTatlas_rh_volume_parsorbitalis
486)	aparc-DKTatlas_rh_volume_parstriangularis
487)	aparc-DKTatlas_rh_volume_pericalcarine
488)	aparc-DKTatlas_rh_volume_postcentral
489)	aparc-DKTatlas_rh_volume_posteriorcingulate
490)	aparc-DKTatlas_rh_volume_precentral
491)	aparc-DKTatlas_rh_volume_precuneus
492)	aparc-DKTatlas_rh_volume_rostralanteriorcingulate
493)	aparc-DKTatlas_rh_volume_rostralmiddlefrontal
494)	aparc-DKTatlas_rh_volume_superiorfrontal
495)	aparc-DKTatlas_rh_volume_superiorparietal
496)	aparc-DKTatlas_rh_volume_superiortemporal
497)	aparc-DKTatlas_rh_volume_supramarginal
498)	aparc-DKTatlas_rh_volume_transversetemporal
499)	aparc-DKTatlas_rh_volume_insula
500)	aparc-a2009s_lh_volume_G+S-frontomargin
501)	aparc-a2009s_lh_volume_G+S-occipital-inf
502)	aparc-a2009s_lh_volume_G+S-paracentral

503)	aparc-a2009s_lh_volume_G+S-subcentral
504)	aparc-a2009s_lh_volume_G+S-transv-frontopol
505)	aparc-a2009s_lh_volume_G+S-cingul-Ant
506)	aparc-a2009s_lh_volume_G+S-cingul-Mid-Ant
507)	aparc-a2009s_lh_volume_G+S-cingul-Mid-Post
508)	aparc-a2009s_lh_volume_G-cingul-Post-dorsal
509)	aparc-a2009s_lh_volume_G-cingul-Post-ventral
510)	aparc-a2009s_lh_volume_G-cuneus
511)	aparc-a2009s_lh_volume_G-front-inf-Opercular
512)	aparc-a2009s_lh_volume_G-front-inf-Orbital
513)	aparc-a2009s_lh_volume_G-front-inf-Triangul
514)	aparc-a2009s_lh_volume_G-front-middle
515)	aparc-a2009s_lh_volume_G-front-sup
516)	aparc-a2009s_lh_volume_G-Ins-lg+S-cent-ins
517)	aparc-a2009s_lh_volume_G-insular-short
518)	aparc-a2009s_lh_volume_G-occipital-middle
519)	aparc-a2009s_lh_volume_G-occipital-sup
520)	aparc-a2009s_lh_volume_G-oc-temp-lat-fusifor
521)	aparc-a2009s_lh_volume_G-oc-temp-med-Lingual
522)	aparc-a2009s_lh_volume_G-oc-temp-med-Parahip
523)	aparc-a2009s_lh_volume_G-orbital
524)	aparc-a2009s_lh_volume_G-pariet-inf-Angular
525)	aparc-a2009s_lh_volume_G-pariet-inf-Supramar
526)	aparc-a2009s_lh_volume_G-parietal-sup
527)	aparc-a2009s_lh_volume_G-postcentral
528)	aparc-a2009s_lh_volume_G-precentral
529)	aparc-a2009s_lh_volume_G-precuneus
530)	aparc-a2009s_lh_volume_G-rectus
531)	aparc-a2009s_lh_volume_G-subcallosal
532)	aparc-a2009s_lh_volume_G-temp-sup-G-T-transv
533)	aparc-a2009s_lh_volume_G-temp-sup-Lateral
534)	aparc-a2009s_lh_volume_G-temp-sup-Plan-polar
535)	aparc-a2009s_lh_volume_G-temp-sup-Plan-tempo
536)	aparc-a2009s_lh_volume_G-temporal-inf
537)	aparc-a2009s_lh_volume_G-temporal-middle
538)	aparc-a2009s_lh_volume_Lat-Fis-ant-Horizont
539)	aparc-a2009s_lh_volume_Lat-Fis-ant-Vertical
540)	aparc-a2009s_lh_volume_Lat-Fis-post
541)	aparc-a2009s_lh_volume_Pole-occipital
542)	aparc-a2009s_lh_volume_Pole-temporal
543)	aparc-a2009s_lh_volume_S-calcarine
544)	aparc-a2009s_lh_volume_S-central

545)	aparc-a2009s_lh_volume_S-cingul-Marginalis
546)	aparc-a2009s_lh_volume_S-circular-insula-ant
547)	aparc-a2009s_lh_volume_S-circular-insula-inf
548)	aparc-a2009s_lh_volume_S-circular-insula-sup
549)	aparc-a2009s_lh_volume_S-collat-transv-ant
550)	aparc-a2009s_lh_volume_S-collat-transv-post
551)	aparc-a2009s_lh_volume_S-front-inf
552)	aparc-a2009s_lh_volume_S-front-middle
553)	aparc-a2009s_lh_volume_S-front-sup
554)	aparc-a2009s_lh_volume_S-interm-prim-Jensen
555)	aparc-a2009s_lh_volume_S-intrapariet+P-trans
556)	aparc-a2009s_lh_volume_S-oc-middle+Lunatus
557)	aparc-a2009s_lh_volume_S-oc-sup+transversal
558)	aparc-a2009s_lh_volume_S-occipital-ant
559)	aparc-a2009s_lh_volume_S-oc-temp-lat
560)	aparc-a2009s_lh_volume_S-oc-temp-med+Lingual
561)	aparc-a2009s_lh_volume_S-orbital-lateral
562)	aparc-a2009s_lh_volume_S-orbital-med-olfact
563)	aparc-a2009s_lh_volume_S-orbital-H-Shaped
564)	aparc-a2009s_lh_volume_S-parieto-occipital
565)	aparc-a2009s_lh_volume_S-pericallosal
566)	aparc-a2009s_lh_volume_S-postcentral
567)	aparc-a2009s_lh_volume_S-precentral-inf-part
568)	aparc-a2009s_lh_volume_S-precentral-sup-part
569)	aparc-a2009s_lh_volume_S-suborbital
570)	aparc-a2009s_lh_volume_S-subparietal
571)	aparc-a2009s_lh_volume_S-temporal-inf
572)	aparc-a2009s_lh_volume_S-temporal-sup
573)	aparc-a2009s_lh_volume_S-temporal-transverse
574)	aparc-a2009s_rh_volume_G+S-frontomargin
575)	aparc-a2009s_rh_volume_G+S-occipital-inf
576)	aparc-a2009s_rh_volume_G+S-paracentral
577)	aparc-a2009s_rh_volume_G+S-subcentral
578)	aparc-a2009s_rh_volume_G+S-transv-frontopol
579)	aparc-a2009s_rh_volume_G+S-cingul-Ant
580)	aparc-a2009s_rh_volume_G+S-cingul-Mid-Ant
581)	aparc-a2009s_rh_volume_G+S-cingul-Mid-Post
582)	aparc-a2009s_rh_volume_G-cingul-Post-dorsal
583)	aparc-a2009s_rh_volume_G-cingul-Post-ventral
584)	aparc-a2009s_rh_volume_G-cuneus
585)	aparc-a2009s_rh_volume_G-front-inf-Opercular
586)	aparc-a2009s_rh_volume_G-front-inf-Orbital

587)	aparc-a2009s_rh_volume_G-front-inf-Triangul
588)	aparc-a2009s_rh_volume_G-front-middle
589)	aparc-a2009s_rh_volume_G-front-sup
590)	aparc-a2009s_rh_volume_G-Ins-lg+S-cent-ins
591)	aparc-a2009s_rh_volume_G-insular-short
592)	aparc-a2009s_rh_volume_G-occipital-middle
593)	aparc-a2009s_rh_volume_G-occipital-sup
594)	aparc-a2009s_rh_volume_G-oc-temp-lat-fusifor
595)	aparc-a2009s_rh_volume_G-oc-temp-med-Lingual
596)	aparc-a2009s_rh_volume_G-oc-temp-med-Parahip
597)	aparc-a2009s_rh_volume_G-orbital
598)	aparc-a2009s_rh_volume_G-pariet-inf-Angular
599)	aparc-a2009s_rh_volume_G-pariet-inf-Supramar
600)	aparc-a2009s_rh_volume_G-parietal-sup
601)	aparc-a2009s_rh_volume_G-postcentral
602)	aparc-a2009s_rh_volume_G-precentral
603)	aparc-a2009s_rh_volume_G-precuneus
604)	aparc-a2009s_rh_volume_G-rectus
605)	aparc-a2009s_rh_volume_G-subcallosal
606)	aparc-a2009s_rh_volume_G-temp-sup-G-T-transv
607)	aparc-a2009s_rh_volume_G-temp-sup-Lateral
608)	aparc-a2009s_rh_volume_G-temp-sup-Plan-polar
609)	aparc-a2009s_rh_volume_G-temp-sup-Plan-tempo
610)	aparc-a2009s_rh_volume_G-temporal-inf
611)	aparc-a2009s_rh_volume_G-temporal-middle
612)	aparc-a2009s_rh_volume_Lat-Fis-ant-Horizont
613)	aparc-a2009s_rh_volume_Lat-Fis-ant-Vertical
614)	aparc-a2009s_rh_volume_Lat-Fis-post
615)	aparc-a2009s_rh_volume_Pole-occipital
616)	aparc-a2009s_rh_volume_Pole-temporal
617)	aparc-a2009s_rh_volume_S-calcarine
618)	aparc-a2009s_rh_volume_S-central
619)	aparc-a2009s_rh_volume_S-cingul-Marginalis
620)	aparc-a2009s_rh_volume_S-circular-insula-ant
621)	aparc-a2009s_rh_volume_S-circular-insula-inf
622)	aparc-a2009s_rh_volume_S-circular-insula-sup
623)	aparc-a2009s_rh_volume_S-collat-transv-ant
624)	aparc-a2009s_rh_volume_S-collat-transv-post
625)	aparc-a2009s_rh_volume_S-front-inf
626)	aparc-a2009s_rh_volume_S-front-middle
627)	aparc-a2009s_rh_volume_S-front-sup
628)	aparc-a2009s_rh_volume_S-interm-prim-Jensen

629)	aparc-a2009s_rh_volume_S-intrapariet+P-trans
630)	aparc-a2009s_rh_volume_S-oc-middle+Lunatus
631)	aparc-a2009s_rh_volume_S-oc-sup+transversal
632)	aparc-a2009s_rh_volume_S-occipital-ant
633)	aparc-a2009s_rh_volume_S-oc-temp-lat
634)	aparc-a2009s_rh_volume_S-oc-temp-med+Lingual
635)	aparc-a2009s_rh_volume_S-orbital-lateral
636)	aparc-a2009s_rh_volume_S-orbital-med-olfact
637)	aparc-a2009s_rh_volume_S-orbital-H-Shaped
638)	aparc-a2009s_rh_volume_S-parieto-occipital
639)	aparc-a2009s_rh_volume_S-pericallosal
640)	aparc-a2009s_rh_volume_S-postcentral
641)	aparc-a2009s_rh_volume_S-precentral-inf-part
642)	aparc-a2009s_rh_volume_S-precentral-sup-part
643)	aparc-a2009s_rh_volume_S-suborbital
644)	aparc-a2009s_rh_volume_S-subparietal
645)	aparc-a2009s_rh_volume_S-temporal-inf
646)	aparc-a2009s_rh_volume_S-temporal-sup
647)	aparc-a2009s_rh_volume_S-temporal-transverse
648)	aparc-Desikan_lh_area_TotalSurface
649)	aparc-Desikan_lh_area_bankssts
650)	aparc-Desikan_lh_area_caudalanteriorcingulate
651)	aparc-Desikan_lh_area_caudalmiddlefrontal
652)	aparc-Desikan_lh_area_cuneus
653)	aparc-Desikan_lh_area_entorhinal
654)	aparc-Desikan_lh_area_fusiform
655)	aparc-Desikan_lh_area_inferiorparietal
656)	aparc-Desikan_lh_area_inferiortemporal
657)	aparc-Desikan_lh_area_isthmuscingulate
658)	aparc-Desikan_lh_area_lateraloccipital
659)	aparc-Desikan_lh_area_lateralorbitofrontal
660)	aparc-Desikan_lh_area_lingual
661)	aparc-Desikan_lh_area_medialorbitofrontal
662)	aparc-Desikan_lh_area_middletemporal
663)	aparc-Desikan_lh_area_parahippocampal
664)	aparc-Desikan_lh_area_paracentral
665)	aparc-Desikan_lh_area_parsopercularis
666)	aparc-Desikan_lh_area_parsorbitalis
667)	aparc-Desikan_lh_area_parstriangularis
668)	aparc-Desikan_lh_area_pericalcarine
669)	aparc-Desikan_lh_area_postcentral
670)	aparc-Desikan_lh_area_posteriorcingulate

671)	aparc-Desikan_lh_area_precentral
672)	aparc-Desikan_lh_area_precuneus
673)	aparc-Desikan_lh_area_rostralanteriorcingulate
674)	aparc-Desikan_lh_area_rostralmiddlefrontal
675)	aparc-Desikan_lh_area_superiorfrontal
676)	aparc-Desikan_lh_area_superiorparietal
677)	aparc-Desikan_lh_area_superiortemporal
678)	aparc-Desikan_lh_area_supramarginal
679)	aparc-Desikan_lh_area_frontalpole
680)	aparc-Desikan_lh_area_transversetemporal
681)	aparc-Desikan_lh_area_insula
682)	aparc-Desikan_rh_area_TotalSurface
683)	aparc-Desikan_rh_area_bankssts
684)	aparc-Desikan_rh_area_caudalanteriorcingulate
685)	aparc-Desikan_rh_area_caudalmiddlefrontal
686)	aparc-Desikan_rh_area_cuneus
687)	aparc-Desikan_rh_area_entorhinal
688)	aparc-Desikan_rh_area_fusiform
689)	aparc-Desikan_rh_area_inferiorparietal
690)	aparc-Desikan_rh_area_inferiortemporal
691)	aparc-Desikan_rh_area_isthmuscingulate
692)	aparc-Desikan_rh_area_lateraloccipital
693)	aparc-Desikan_rh_area_lateralorbitofrontal
694)	aparc-Desikan_rh_area_lingual
695)	aparc-Desikan_rh_area_medialorbitofrontal
696)	aparc-Desikan_rh_area_middletemporal
697)	aparc-Desikan_rh_area_parahippocampal
698)	aparc-Desikan_rh_area_paracentral
699)	aparc-Desikan_rh_area_parsopercularis
700)	aparc-Desikan_rh_area_parsorbitalis
701)	aparc-Desikan_rh_area_parstriangularis
702)	aparc-Desikan_rh_area_pericalcarine
703)	aparc-Desikan_rh_area_postcentral
704)	aparc-Desikan_rh_area_posteriorcingulate
705)	aparc-Desikan_rh_area_precentral
706)	aparc-Desikan_rh_area_precuneus
707)	aparc-Desikan_rh_area_rostralanteriorcingulate
708)	aparc-Desikan_rh_area_rostralmiddlefrontal
709)	aparc-Desikan_rh_area_superiorfrontal
710)	aparc-Desikan_rh_area_superiorparietal
711)	aparc-Desikan_rh_area_superiortemporal
712)	aparc-Desikan_rh_area_supramarginal

713)	aparc-Desikan_rh_area_frontalpole
714)	aparc-Desikan_rh_area_transversetemporal
715)	aparc-Desikan_rh_area_insula
716)	aparc-pial_lh_area_TotalSurface
717)	aparc-pial_lh_area_bankssts
718)	aparc-pial_lh_area_caudalanteriorcingulate
719)	aparc-pial_lh_area_caudalmiddlefrontal
720)	aparc-pial_lh_area_cuneus
721)	aparc-pial_lh_area_entorhinal
722)	aparc-pial_lh_area_fusiform
723)	aparc-pial_lh_area_inferiorparietal
724)	aparc-pial_lh_area_inferiortemporal
725)	aparc-pial_lh_area_isthmuscingulate
726)	aparc-pial_lh_area_lateraloccipital
727)	aparc-pial_lh_area_lateralorbitofrontal
728)	aparc-pial_lh_area_lingual
729)	aparc-pial_lh_area_medialorbitofrontal
730)	aparc-pial_lh_area_middletemporal
731)	aparc-pial_lh_area_parahippocampal
732)	aparc-pial_lh_area_paracentral
733)	aparc-pial_lh_area_parsopercularis
734)	aparc-pial_lh_area_parsorbitalis
735)	aparc-pial_lh_area_parstriangularis
736)	aparc-pial_lh_area_pericalcarine
737)	aparc-pial_lh_area_postcentral
738)	aparc-pial_lh_area_posteriorcingulate
739)	aparc-pial_lh_area_precentral
740)	aparc-pial_lh_area_precuneus
741)	aparc-pial_lh_area_rostralanteriorcingulate
742)	aparc-pial_lh_area_rostralmiddlefrontal
743)	aparc-pial_lh_area_superiorfrontal
744)	aparc-pial_lh_area_superiorparietal
745)	aparc-pial_lh_area_superiortemporal
746)	aparc-pial_lh_area_supramarginal
747)	aparc-pial_lh_area_frontalpole
748)	aparc-pial_lh_area_transversetemporal
749)	aparc-pial_rh_area_TotalSurface
750)	aparc-pial_rh_area_bankssts
751)	aparc-pial_rh_area_caudalanteriorcingulate
752)	aparc-pial_rh_area_caudalmiddlefrontal
753)	aparc-pial_rh_area_cuneus
754)	aparc-pial_rh_area_entorhinal

755)	aparc-pial_rh_area_fusiform
756)	aparc-pial_rh_area_inferiorparietal
757)	aparc-pial_rh_area_inferiortemporal
758)	aparc-pial_rh_area_isthmuscingulate
759)	aparc-pial_rh_area_lateraloccipital
760)	aparc-pial_rh_area_lateralorbitofrontal
761)	aparc-pial_rh_area_lingual
762)	aparc-pial_rh_area_medialorbitofrontal
763)	aparc-pial_rh_area_middletemporal
764)	aparc-pial_rh_area_parahippocampal
765)	aparc-pial_rh_area_paracentral
766)	aparc-pial_rh_area_parsopercularis
767)	aparc-pial_rh_area_parsorbitalis
768)	aparc-pial_rh_area_parstriangularis
769)	aparc-pial_rh_area_pericalcarine
770)	aparc-pial_rh_area_postcentral
771)	aparc-pial_rh_area_posteriorcingulate
772)	aparc-pial_rh_area_precentral
773)	aparc-pial_rh_area_precuneus
774)	aparc-pial_rh_area_rostralanteriorcingulate
775)	aparc-pial_rh_area_rostralmiddlefrontal
776)	aparc-pial_rh_area_superiorfrontal
777)	aparc-pial_rh_area_superiorparietal
778)	aparc-pial_rh_area_superiortemporal
779)	aparc-pial_rh_area_supramarginal
780)	aparc-pial_rh_area_frontalpole
781)	aparc-pial_rh_area_transversetemporal
782)	BA-exvivo_lh_area_BA1
783)	BA-exvivo_lh_area_BA2
784)	BA-exvivo_lh_area_BA3a
785)	BA-exvivo_lh_area_BA3b
786)	BA-exvivo_lh_area_BA4a
787)	BA-exvivo_lh_area_BA4p
788)	BA-exvivo_lh_area_BA6
789)	BA-exvivo_lh_area_BA44
790)	BA-exvivo_lh_area_BA45
791)	BA-exvivo_lh_area_V1
792)	BA-exvivo_lh_area_V2
793)	BA-exvivo_lh_area_MT
794)	BA-exvivo_lh_area_perirhinal
795)	BA-exvivo_lh_area_entorhinal
796)	BA-exvivo_rh_area_BA1

797)	BA-exvivo_rh_area_BA2
798)	BA-exvivo_rh_area_BA3a
799)	BA-exvivo_rh_area_BA3b
800)	BA-exvivo_rh_area_BA4a
801)	BA-exvivo_rh_area_BA4p
802)	BA-exvivo_rh_area_BA6
803)	BA-exvivo_rh_area_BA44
804)	BA-exvivo_rh_area_BA45
805)	BA-exvivo_rh_area_V1
806)	BA-exvivo_rh_area_V2
807)	BA-exvivo_rh_area_MT
808)	BA-exvivo_rh_area_perirhinal
809)	BA-exvivo_rh_area_entorhinal
810)	aparc-DKTatlas_lh_area_caudalanteriorcingulate
811)	aparc-DKTatlas_lh_area_caudalmiddlefrontal
812)	aparc-DKTatlas_lh_area_cuneus
813)	aparc-DKTatlas_lh_area_entorhinal
814)	aparc-DKTatlas_lh_area_fusiform
815)	aparc-DKTatlas_lh_area_inferiorparietal
816)	aparc-DKTatlas_lh_area_inferiortemporal
817)	aparc-DKTatlas_lh_area_isthmuscingulate
818)	aparc-DKTatlas_lh_area_lateraloccipital
819)	aparc-DKTatlas_lh_area_lateralorbitofrontal
820)	aparc-DKTatlas_lh_area_lingual
821)	aparc-DKTatlas_lh_area_medialorbitofrontal
822)	aparc-DKTatlas_lh_area_middletemporal
823)	aparc-DKTatlas_lh_area_parahippocampal
824)	aparc-DKTatlas_lh_area_paracentral
825)	aparc-DKTatlas_lh_area_parsopercularis
826)	aparc-DKTatlas_lh_area_parsorbitalis
827)	aparc-DKTatlas_lh_area_parstriangularis
828)	aparc-DKTatlas_lh_area_pericalcarine
829)	aparc-DKTatlas_lh_area_postcentral
830)	aparc-DKTatlas_lh_area_posteriorcingulate
831)	aparc-DKTatlas_lh_area_precentral
832)	aparc-DKTatlas_lh_area_precuneus
833)	aparc-DKTatlas_lh_area_rostralanteriorcingulate
834)	aparc-DKTatlas_lh_area_rostralmiddlefrontal
835)	aparc-DKTatlas_lh_area_superiorfrontal
836)	aparc-DKTatlas_lh_area_superiorparietal
837)	aparc-DKTatlas_lh_area_superiortemporal
838)	aparc-DKTatlas_lh_area_supramarginal

839)	aparc-DKTatlas_lh_area_transversetemporal
840)	aparc-DKTatlas_lh_area_insula
841)	aparc-DKTatlas_rh_area_caudalanteriorcingulate
842)	aparc-DKTatlas_rh_area_caudalmiddlefrontal
843)	aparc-DKTatlas_rh_area_cuneus
844)	aparc-DKTatlas_rh_area_entorhinal
845)	aparc-DKTatlas_rh_area_fusiform
846)	aparc-DKTatlas_rh_area_inferiorparietal
847)	aparc-DKTatlas_rh_area_inferiortemporal
848)	aparc-DKTatlas_rh_area_isthmuscingulate
849)	aparc-DKTatlas_rh_area_lateraloccipital
850)	aparc-DKTatlas_rh_area_lateralorbitofrontal
851)	aparc-DKTatlas_rh_area_lingual
852)	aparc-DKTatlas_rh_area_medialorbitofrontal
853)	aparc-DKTatlas_rh_area_middletemporal
854)	aparc-DKTatlas_rh_area_parahippocampal
855)	aparc-DKTatlas_rh_area_paracentral
856)	aparc-DKTatlas_rh_area_parsopercularis
857)	aparc-DKTatlas_rh_area_parsorbitalis
858)	aparc-DKTatlas_rh_area_parstriangularis
859)	aparc-DKTatlas_rh_area_pericalcarine
860)	aparc-DKTatlas_rh_area_postcentral
861)	aparc-DKTatlas_rh_area_posteriorcingulate
862)	aparc-DKTatlas_rh_area_precentral
863)	aparc-DKTatlas_rh_area_precuneus
864)	aparc-DKTatlas_rh_area_rostralanteriorcingulate
865)	aparc-DKTatlas_rh_area_rostralmiddlefrontal
866)	aparc-DKTatlas_rh_area_superiorfrontal
867)	aparc-DKTatlas_rh_area_superiorparietal
868)	aparc-DKTatlas_rh_area_superiortemporal
869)	aparc-DKTatlas_rh_area_supramarginal
870)	aparc-DKTatlas_rh_area_transversetemporal
871)	aparc-DKTatlas_rh_area_insula
872)	aparc-a2009s_lh_area_G+S-frontomargin
873)	aparc-a2009s_lh_area_G+S-occipital-inf
874)	aparc-a2009s_lh_area_G+S-paracentral
875)	aparc-a2009s_lh_area_G+S-subcentral
876)	aparc-a2009s_lh_area_G+S-transv-frontopol
877)	aparc-a2009s_lh_area_G+S-cingul-Ant
878)	aparc-a2009s_lh_area_G+S-cingul-Mid-Ant
879)	aparc-a2009s_lh_area_G+S-cingul-Mid-Post
880)	aparc-a2009s_lh_area_G-cingul-Post-dorsal

881)	aparc-a2009s_lh_area_G-cingul-Post-ventral
882)	aparc-a2009s_lh_area_G-cuneus
883)	aparc-a2009s_lh_area_G-front-inf-Opercular
884)	aparc-a2009s_lh_area_G-front-inf-Orbital
885)	aparc-a2009s_lh_area_G-front-inf-Triangul
886)	aparc-a2009s_lh_area_G-front-middle
887)	aparc-a2009s_lh_area_G-front-sup
888)	aparc-a2009s_lh_area_G-Ins-lg+S-cent-ins
889)	aparc-a2009s_lh_area_G-insular-short
890)	aparc-a2009s_lh_area_G-occipital-middle
891)	aparc-a2009s_lh_area_G-occipital-sup
892)	aparc-a2009s_lh_area_G-oc-temp-lat-fusifor
893)	aparc-a2009s_lh_area_G-oc-temp-med-Lingual
894)	aparc-a2009s_lh_area_G-oc-temp-med-Parahip
895)	aparc-a2009s_lh_area_G-orbital
896)	aparc-a2009s_lh_area_G-pariet-inf-Angular
897)	aparc-a2009s_lh_area_G-pariet-inf-Supramar
898)	aparc-a2009s_lh_area_G-parietal-sup
899)	aparc-a2009s_lh_area_G-postcentral
900)	aparc-a2009s_lh_area_G-precentral
901)	aparc-a2009s_lh_area_G-precuneus
902)	aparc-a2009s_lh_area_G-rectus
903)	aparc-a2009s_lh_area_G-subcallosal
904)	aparc-a2009s_lh_area_G-temp-sup-G-T-transv
905)	aparc-a2009s_lh_area_G-temp-sup-Lateral
906)	aparc-a2009s_lh_area_G-temp-sup-Plan-polar
907)	aparc-a2009s_lh_area_G-temp-sup-Plan-tempo
908)	aparc-a2009s_lh_area_G-temporal-inf
909)	aparc-a2009s_lh_area_G-temporal-middle
910)	aparc-a2009s_lh_area_Lat-Fis-ant-Horizont
911)	aparc-a2009s_lh_area_Lat-Fis-ant-Vertical
912)	aparc-a2009s_lh_area_Lat-Fis-post
913)	aparc-a2009s_lh_area_Pole-occipital
914)	aparc-a2009s_lh_area_Pole-temporal
915)	aparc-a2009s_lh_area_S-calcarine
916)	aparc-a2009s_lh_area_S-central
917)	aparc-a2009s_lh_area_S-cingul-Marginalis
918)	aparc-a2009s_lh_area_S-circular-insula-ant
919)	aparc-a2009s_lh_area_S-circular-insula-inf
920)	aparc-a2009s_lh_area_S-circular-insula-sup
921)	aparc-a2009s_lh_area_S-collat-transv-ant
922)	aparc-a2009s_lh_area_S-collat-transv-post

923)	aparc-a2009s_lh_area_S-front-inf
924)	aparc-a2009s_lh_area_S-front-middle
925)	aparc-a2009s_lh_area_S-front-sup
926)	aparc-a2009s_lh_area_S-interm-prim-Jensen
927)	aparc-a2009s_lh_area_S-intrapariet+P-trans
928)	aparc-a2009s_lh_area_S-oc-middle+Lunatus
929)	aparc-a2009s_lh_area_S-oc-sup+transversal
930)	aparc-a2009s_lh_area_S-occipital-ant
931)	aparc-a2009s_lh_area_S-oc-temp-lat
932)	aparc-a2009s_lh_area_S-oc-temp-med+Lingual
933)	aparc-a2009s_lh_area_S-orbital-lateral
934)	aparc-a2009s_lh_area_S-orbital-med-olfact
935)	aparc-a2009s_lh_area_S-orbital-H-Shaped
936)	aparc-a2009s_lh_area_S-parieto-occipital
937)	aparc-a2009s_lh_area_S-pericallosal
938)	aparc-a2009s_lh_area_S-postcentral
939)	aparc-a2009s_lh_area_S-precentral-inf-part
940)	aparc-a2009s_lh_area_S-precentral-sup-part
941)	aparc-a2009s_lh_area_S-suborbital
942)	aparc-a2009s_lh_area_S-subparietal
943)	aparc-a2009s_lh_area_S-temporal-inf
944)	aparc-a2009s_lh_area_S-temporal-sup
945)	aparc-a2009s_lh_area_S-temporal-transverse
946)	aparc-a2009s_rh_area_G+S-frontomargin
947)	aparc-a2009s_rh_area_G+S-occipital-inf
948)	aparc-a2009s_rh_area_G+S-paracentral
949)	aparc-a2009s_rh_area_G+S-subcentral
950)	aparc-a2009s_rh_area_G+S-transv-frontopol
951)	aparc-a2009s_rh_area_G+S-cingul-Ant
952)	aparc-a2009s_rh_area_G+S-cingul-Mid-Ant
953)	aparc-a2009s_rh_area_G+S-cingul-Mid-Post
954)	aparc-a2009s_rh_area_G-cingul-Post-dorsal
955)	aparc-a2009s_rh_area_G-cingul-Post-ventral
956)	aparc-a2009s_rh_area_G-cuneus
957)	aparc-a2009s_rh_area_G-front-inf-Opercular
958)	aparc-a2009s_rh_area_G-front-inf-Orbital
959)	aparc-a2009s_rh_area_G-front-inf-Triangul
960)	aparc-a2009s_rh_area_G-front-middle
961)	aparc-a2009s_rh_area_G-front-sup
962)	aparc-a2009s_rh_area_G-Ins-lg+S-cent-ins
963)	aparc-a2009s_rh_area_G-insular-short
964)	aparc-a2009s_rh_area_G-occipital-middle

965)	aparc-a2009s_rh_area_G-occipital-sup
966)	aparc-a2009s_rh_area_G-oc-temp-lat-fusifor
967)	aparc-a2009s_rh_area_G-oc-temp-med-Lingual
968)	aparc-a2009s_rh_area_G-oc-temp-med-Parahip
969)	aparc-a2009s_rh_area_G-orbital
970)	aparc-a2009s_rh_area_G-pariet-inf-Angular
971)	aparc-a2009s_rh_area_G-pariet-inf-Supramar
972)	aparc-a2009s_rh_area_G-parietal-sup
973)	aparc-a2009s_rh_area_G-postcentral
974)	aparc-a2009s_rh_area_G-precentral
975)	aparc-a2009s_rh_area_G-precuneus
976)	aparc-a2009s_rh_area_G-rectus
977)	aparc-a2009s_rh_area_G-subcallosal
978)	aparc-a2009s_rh_area_G-temp-sup-G-T-transv
979)	aparc-a2009s_rh_area_G-temp-sup-Lateral
980)	aparc-a2009s_rh_area_G-temp-sup-Plan-polar
981)	aparc-a2009s_rh_area_G-temp-sup-Plan-tempo
982)	aparc-a2009s_rh_area_G-temporal-inf
983)	aparc-a2009s_rh_area_G-temporal-middle
984)	aparc-a2009s_rh_area_Lat-Fis-ant-Horizont
985)	aparc-a2009s_rh_area_Lat-Fis-ant-Vertical
986)	aparc-a2009s_rh_area_Lat-Fis-post
987)	aparc-a2009s_rh_area_Pole-occipital
988)	aparc-a2009s_rh_area_Pole-temporal
989)	aparc-a2009s_rh_area_S-calcarine
990)	aparc-a2009s_rh_area_S-central
991)	aparc-a2009s_rh_area_S-cingul-Marginalis
992)	aparc-a2009s_rh_area_S-circular-insula-ant
993)	aparc-a2009s_rh_area_S-circular-insula-inf
994)	aparc-a2009s_rh_area_S-circular-insula-sup
995)	aparc-a2009s_rh_area_S-collat-transv-ant
996)	aparc-a2009s_rh_area_S-collat-transv-post
997)	aparc-a2009s_rh_area_S-front-inf
998)	aparc-a2009s_rh_area_S-front-middle
999)	aparc-a2009s_rh_area_S-front-sup
1000)	aparc-a2009s_rh_area_S-interm-prim-Jensen
1001)	aparc-a2009s_rh_area_S-intrapariet+P-trans
1002)	aparc-a2009s_rh_area_S-oc-middle+Lunatus
1003)	aparc-a2009s_rh_area_S-oc-sup+transversal
1004)	aparc-a2009s_rh_area_S-occipital-ant
1005)	aparc-a2009s_rh_area_S-oc-temp-lat
1006)	aparc-a2009s_rh_area_S-oc-temp-med+Lingual

1007)	aparc-a2009s_rh_area_S-orbital-lateral
1008)	aparc-a2009s_rh_area_S-orbital-med-olfact
1009)	aparc-a2009s_rh_area_S-orbital-H-Shaped
1010)	aparc-a2009s_rh_area_S-parieto-occipital
1011)	aparc-a2009s_rh_area_S-pericallosal
1012)	aparc-a2009s_rh_area_S-postcentral
1013)	aparc-a2009s_rh_area_S-precentral-inf-part
1014)	aparc-a2009s_rh_area_S-precentral-sup-part
1015)	aparc-a2009s_rh_area_S-suborbital
1016)	aparc-a2009s_rh_area_S-subparietal
1017)	aparc-a2009s_rh_area_S-temporal-inf
1018)	aparc-a2009s_rh_area_S-temporal-sup
1019)	aparc-a2009s_rh_area_S-temporal-transverse
1020)	aparc-Desikan_lh_thickness_GlobalMeanThickness
1021)	aparc-Desikan_lh_thickness_bankssts
1022)	aparc-Desikan_lh_thickness_caudalanteriorcingulate
1023)	aparc-Desikan_lh_thickness_caudalmiddlefrontal
1024)	aparc-Desikan_lh_thickness_cuneus
1025)	aparc-Desikan_lh_thickness_entorhinal
1026)	aparc-Desikan_lh_thickness_fusiform
1027)	aparc-Desikan_lh_thickness_inferiorparietal
1028)	aparc-Desikan_lh_thickness_inferiortemporal
1029)	aparc-Desikan_lh_thickness_isthmuscingulate
1030)	aparc-Desikan_lh_thickness_lateraloccipital
1031)	aparc-Desikan_lh_thickness_lateralorbitofrontal
1032)	aparc-Desikan_lh_thickness_lingual
1033)	aparc-Desikan_lh_thickness_medialorbitofrontal
1034)	aparc-Desikan_lh_thickness_middletemporal
1035)	aparc-Desikan_lh_thickness_parahippocampal
1036)	aparc-Desikan_lh_thickness_paracentral
1037)	aparc-Desikan_lh_thickness_parsopercularis
1038)	aparc-Desikan_lh_thickness_parsorbitalis
1039)	aparc-Desikan_lh_thickness_parstriangularis
1040)	aparc-Desikan_lh_thickness_pericalcarine
1041)	aparc-Desikan_lh_thickness_postcentral
1042)	aparc-Desikan_lh_thickness_posteriorcingulate
1043)	aparc-Desikan_lh_thickness_precentral
1044)	aparc-Desikan_lh_thickness_precuneus
1045)	aparc-Desikan_lh_thickness_rostralanteriorcingulate
1046)	aparc-Desikan_lh_thickness_rostralmiddlefrontal
1047)	aparc-Desikan_lh_thickness_superiorfrontal
1048)	aparc-Desikan_lh_thickness_superiorparietal

1049)	aparc-Desikan_lh_thickness_superiortemporal
1050)	aparc-Desikan_lh_thickness_supramarginal
1051)	aparc-Desikan_lh_thickness_frontalpole
1052)	aparc-Desikan_lh_thickness_transversetemporal
1053)	aparc-Desikan_lh_thickness_insula
1054)	aparc-Desikan_rh_thickness_GlobalMeanThickness
1055)	aparc-Desikan_rh_thickness_bankssts
1056)	aparc-Desikan_rh_thickness_caudalanteriorcingulate
1057)	aparc-Desikan_rh_thickness_caudalmiddlefrontal
1058)	aparc-Desikan_rh_thickness_cuneus
1059)	aparc-Desikan_rh_thickness_entorhinal
1060)	aparc-Desikan_rh_thickness_fusiform
1061)	aparc-Desikan_rh_thickness_inferiorparietal
1062)	aparc-Desikan_rh_thickness_inferiortemporal
1063)	aparc-Desikan_rh_thickness_isthmuscingulate
1064)	aparc-Desikan_rh_thickness_lateraloccipital
1065)	aparc-Desikan_rh_thickness_lateralorbitofrontal
1066)	aparc-Desikan_rh_thickness_lingual
1067)	aparc-Desikan_rh_thickness_medialorbitofrontal
1068)	aparc-Desikan_rh_thickness_middletemporal
1069)	aparc-Desikan_rh_thickness_parahippocampal
1070)	aparc-Desikan_rh_thickness_paracentral
1071)	aparc-Desikan_rh_thickness_parsopercularis
1072)	aparc-Desikan_rh_thickness_parsorbitalis
1073)	aparc-Desikan_rh_thickness_parstriangularis
1074)	aparc-Desikan_rh_thickness_pericalcarine
1075)	aparc-Desikan_rh_thickness_postcentral
1076)	aparc-Desikan_rh_thickness_posteriorcingulate
1077)	aparc-Desikan_rh_thickness_precentral
1078)	aparc-Desikan_rh_thickness_precuneus
1079)	aparc-Desikan_rh_thickness_rostralanteriorcingulate
1080)	aparc-Desikan_rh_thickness_rostralmiddlefrontal
1081)	aparc-Desikan_rh_thickness_superiorfrontal
1082)	aparc-Desikan_rh_thickness_superiorparietal
1083)	aparc-Desikan_rh_thickness_superiortemporal
1084)	aparc-Desikan_rh_thickness_supramarginal
1085)	aparc-Desikan_rh_thickness_frontalpole
1086)	aparc-Desikan_rh_thickness_transversetemporal
1087)	aparc-Desikan_rh_thickness_insula
1088)	BA-exvivo_lh_thickness_BA1
1089)	BA-exvivo_lh_thickness_BA2
1090)	BA-exvivo_lh_thickness_BA3a

1091)	BA-exvivo_lh_thickness_BA3b
1092)	BA-exvivo_lh_thickness_BA4a
1093)	BA-exvivo_lh_thickness_BA4p
1094)	BA-exvivo_lh_thickness_BA6
1095)	BA-exvivo_lh_thickness_BA44
1096)	BA-exvivo_lh_thickness_BA45
1097)	BA-exvivo_lh_thickness_V1
1098)	BA-exvivo_lh_thickness_V2
1099)	BA-exvivo_lh_thickness_MT
1100)	BA-exvivo_lh_thickness_perirhinal
1101)	BA-exvivo_lh_thickness_entorhinal
1102)	BA-exvivo_rh_thickness_BA1
1103)	BA-exvivo_rh_thickness_BA2
1104)	BA-exvivo_rh_thickness_BA3a
1105)	BA-exvivo_rh_thickness_BA3b
1106)	BA-exvivo_rh_thickness_BA4a
1107)	BA-exvivo_rh_thickness_BA4p
1108)	BA-exvivo_rh_thickness_BA6
1109)	BA-exvivo_rh_thickness_BA44
1110)	BA-exvivo_rh_thickness_BA45
1111)	BA-exvivo_rh_thickness_V1
1112)	BA-exvivo_rh_thickness_V2
1113)	BA-exvivo_rh_thickness_MT
1114)	BA-exvivo_rh_thickness_perirhinal
1115)	BA-exvivo_rh_thickness_entorhinal
1116)	aparc-DKTatlas_lh_thickness_caudalanteriorcingulate
1117)	aparc-DKTatlas_lh_thickness_caudalmiddlefrontal
1118)	aparc-DKTatlas_lh_thickness_cuneus
1119)	aparc-DKTatlas_lh_thickness_entorhinal
1120)	aparc-DKTatlas_lh_thickness_fusiform
1121)	aparc-DKTatlas_lh_thickness_inferiorparietal
1122)	aparc-DKTatlas_lh_thickness_inferiortemporal
1123)	aparc-DKTatlas_lh_thickness_isthmuscingulate
1124)	aparc-DKTatlas_lh_thickness_lateraloccipital
1125)	aparc-DKTatlas_lh_thickness_lateralorbitofrontal
1126)	aparc-DKTatlas_lh_thickness_lingual
1127)	aparc-DKTatlas_lh_thickness_medialorbitofrontal
1128)	aparc-DKTatlas_lh_thickness_middletemporal
1129)	aparc-DKTatlas_lh_thickness_parahippocampal
1130)	aparc-DKTatlas_lh_thickness_paracentral
1131)	aparc-DKTatlas_lh_thickness_parsopercularis
1132)	aparc-DKTatlas_lh_thickness_parsorbitalis

1133)	aparc-DKTatlas_lh_thickness_parstriangularis
1134)	aparc-DKTatlas_lh_thickness_pericalcarine
1135)	aparc-DKTatlas_lh_thickness_postcentral
1136)	aparc-DKTatlas_lh_thickness_posteriorcingulate
1137)	aparc-DKTatlas_lh_thickness_precentral
1138)	aparc-DKTatlas_lh_thickness_precuneus
1139)	aparc-DKTatlas_lh_thickness_rostralanteriorcingulate
1140)	aparc-DKTatlas_lh_thickness_rostralmiddlefrontal
1141)	aparc-DKTatlas_lh_thickness_superiorfrontal
1142)	aparc-DKTatlas_lh_thickness_superiorparietal
1143)	aparc-DKTatlas_lh_thickness_superiortemporal
1144)	aparc-DKTatlas_lh_thickness_supramarginal
1145)	aparc-DKTatlas_lh_thickness_transversetemporal
1146)	aparc-DKTatlas_lh_thickness_insula
1147)	aparc-DKTatlas_rh_thickness_caudalanteriorcingulate
1148)	aparc-DKTatlas_rh_thickness_caudalmiddlefrontal
1149)	aparc-DKTatlas_rh_thickness_cuneus
1150)	aparc-DKTatlas_rh_thickness_entorhinal
1151)	aparc-DKTatlas_rh_thickness_fusiform
1152)	aparc-DKTatlas_rh_thickness_inferiorparietal
1153)	aparc-DKTatlas_rh_thickness_inferiortemporal
1154)	aparc-DKTatlas_rh_thickness_isthmuscingulate
1155)	aparc-DKTatlas_rh_thickness_lateraloccipital
1156)	aparc-DKTatlas_rh_thickness_lateralorbitofrontal
1157)	aparc-DKTatlas_rh_thickness_lingual
1158)	aparc-DKTatlas_rh_thickness_medialorbitofrontal
1159)	aparc-DKTatlas_rh_thickness_middletemporal
1160)	aparc-DKTatlas_rh_thickness_parahippocampal
1161)	aparc-DKTatlas_rh_thickness_paracentral
1162)	aparc-DKTatlas_rh_thickness_parsopercularis
1163)	aparc-DKTatlas_rh_thickness_parsorbitalis
1164)	aparc-DKTatlas_rh_thickness_parstriangularis
1165)	aparc-DKTatlas_rh_thickness_pericalcarine
1166)	aparc-DKTatlas_rh_thickness_postcentral
1167)	aparc-DKTatlas_rh_thickness_posteriorcingulate
1168)	aparc-DKTatlas_rh_thickness_precentral
1169)	aparc-DKTatlas_rh_thickness_precuneus
1170)	aparc-DKTatlas_rh_thickness_rostralanteriorcingulate
1171)	aparc-DKTatlas_rh_thickness_rostralmiddlefrontal
1172)	aparc-DKTatlas_rh_thickness_superiorfrontal
1173)	aparc-DKTatlas_rh_thickness_superiorparietal
1174)	aparc-DKTatlas_rh_thickness_superiortemporal

1175)	aparc-DKTatlas_rh_thickness_supramarginal
1176)	aparc-DKTatlas_rh_thickness_transversetemporal
1177)	aparc-DKTatlas_rh_thickness_insula
1178)	aparc-a2009s_lh_thickness_G+S-frontomargin
1179)	aparc-a2009s_lh_thickness_G+S-occipital-inf
1180)	aparc-a2009s_lh_thickness_G+S-paracentral
1181)	aparc-a2009s_lh_thickness_G+S-subcentral
1182)	aparc-a2009s_lh_thickness_G+S-transv-frontopol
1183)	aparc-a2009s_lh_thickness_G+S-cingul-Ant
1184)	aparc-a2009s_lh_thickness_G+S-cingul-Mid-Ant
1185)	aparc-a2009s_lh_thickness_G+S-cingul-Mid-Post
1186)	aparc-a2009s_lh_thickness_G-cingul-Post-dorsal
1187)	aparc-a2009s_lh_thickness_G-cingul-Post-ventral
1188)	aparc-a2009s_lh_thickness_G-cuneus
1189)	aparc-a2009s_lh_thickness_G-front-inf-Opercular
1190)	aparc-a2009s_lh_thickness_G-front-inf-Orbital
1191)	aparc-a2009s_lh_thickness_G-front-inf-Triangul
1192)	aparc-a2009s_lh_thickness_G-front-middle
1193)	aparc-a2009s_lh_thickness_G-front-sup
1194)	aparc-a2009s_lh_thickness_G-Ins-lg+S-cent-ins
1195)	aparc-a2009s_lh_thickness_G-insular-short
1196)	aparc-a2009s_lh_thickness_G-occipital-middle
1197)	aparc-a2009s_lh_thickness_G-occipital-sup
1198)	aparc-a2009s_lh_thickness_G-oc-temp-lat-fusifor
1199)	aparc-a2009s_lh_thickness_G-oc-temp-med-Lingual
1200)	aparc-a2009s_lh_thickness_G-oc-temp-med-Parahip
1201)	aparc-a2009s_lh_thickness_G-orbital
1202)	aparc-a2009s_lh_thickness_G-pariet-inf-Angular
1203)	aparc-a2009s_lh_thickness_G-pariet-inf-Supramar
1204)	aparc-a2009s_lh_thickness_G-parietal-sup
1205)	aparc-a2009s_lh_thickness_G-postcentral
1206)	aparc-a2009s_lh_thickness_G-precentral
1207)	aparc-a2009s_lh_thickness_G-precuneus
1208)	aparc-a2009s_lh_thickness_G-rectus
1209)	aparc-a2009s_lh_thickness_G-subcallosal
1210)	aparc-a2009s_lh_thickness_G-temp-sup-G-T-transv
1211)	aparc-a2009s_lh_thickness_G-temp-sup-Lateral
1212)	aparc-a2009s_lh_thickness_G-temp-sup-Plan-polar
1213)	aparc-a2009s_lh_thickness_G-temp-sup-Plan-tempo
1214)	aparc-a2009s_lh_thickness_G-temporal-inf
1215)	aparc-a2009s_lh_thickness_G-temporal-middle
1216)	aparc-a2009s_lh_thickness_Lat-Fis-ant-Horizont

1217)	aparc-a2009s_lh_thickness_Lat-Fis-ant-Vertical
1218)	aparc-a2009s_lh_thickness_Lat-Fis-post
1219)	aparc-a2009s_lh_thickness_Pole-occipital
1220)	aparc-a2009s_lh_thickness_Pole-temporal
1221)	aparc-a2009s_lh_thickness_S-calcarine
1222)	aparc-a2009s_lh_thickness_S-central
1223)	aparc-a2009s_lh_thickness_S-cingul-Marginalis
1224)	aparc-a2009s_lh_thickness_S-circular-insula-ant
1225)	aparc-a2009s_lh_thickness_S-circular-insula-inf
1226)	aparc-a2009s_lh_thickness_S-circular-insula-sup
1227)	aparc-a2009s_lh_thickness_S-collat-transv-ant
1228)	aparc-a2009s_lh_thickness_S-collat-transv-post
1229)	aparc-a2009s_lh_thickness_S-front-inf
1230)	aparc-a2009s_lh_thickness_S-front-middle
1231)	aparc-a2009s_lh_thickness_S-front-sup
1232)	aparc-a2009s_lh_thickness_S-interm-prim-Jensen
1233)	aparc-a2009s_lh_thickness_S-intrapariet+P-trans
1234)	aparc-a2009s_lh_thickness_S-oc-middle+Lunatus
1235)	aparc-a2009s_lh_thickness_S-oc-sup+transversal
1236)	aparc-a2009s_lh_thickness_S-occipital-ant
1237)	aparc-a2009s_lh_thickness_S-oc-temp-lat
1238)	aparc-a2009s_lh_thickness_S-oc-temp-med+Lingual
1239)	aparc-a2009s_lh_thickness_S-orbital-lateral
1240)	aparc-a2009s_lh_thickness_S-orbital-med-olfact
1241)	aparc-a2009s_lh_thickness_S-orbital-H-Shaped
1242)	aparc-a2009s_lh_thickness_S-parieto-occipital
1243)	aparc-a2009s_lh_thickness_S-pericallosal
1244)	aparc-a2009s_lh_thickness_S-postcentral
1245)	aparc-a2009s_lh_thickness_S-precentral-inf-part
1246)	aparc-a2009s_lh_thickness_S-precentral-sup-part
1247)	aparc-a2009s_lh_thickness_S-suborbital
1248)	aparc-a2009s_lh_thickness_S-subparietal
1249)	aparc-a2009s_lh_thickness_S-temporal-inf
1250)	aparc-a2009s_lh_thickness_S-temporal-sup
1251)	aparc-a2009s_lh_thickness_S-temporal-transverse
1252)	aparc-a2009s_rh_thickness_G+S-frontomargin
1253)	aparc-a2009s_rh_thickness_G+S-occipital-inf
1254)	aparc-a2009s_rh_thickness_G+S-paracentral
1255)	aparc-a2009s_rh_thickness_G+S-subcentral
1256)	aparc-a2009s_rh_thickness_G+S-transv-frontopol
1257)	aparc-a2009s_rh_thickness_G+S-cingul-Ant
1258)	aparc-a2009s_rh_thickness_G+S-cingul-Mid-Ant

1259)	aparc-a2009s_rh_thickness_G+S-cingul-Mid-Post
1260)	aparc-a2009s_rh_thickness_G-cingul-Post-dorsal
1261)	aparc-a2009s_rh_thickness_G-cingul-Post-ventral
1262)	aparc-a2009s_rh_thickness_G-cuneus
1263)	aparc-a2009s_rh_thickness_G-front-inf-Opercular
1264)	aparc-a2009s_rh_thickness_G-front-inf-Orbital
1265)	aparc-a2009s_rh_thickness_G-front-inf-Triangul
1266)	aparc-a2009s_rh_thickness_G-front-middle
1267)	aparc-a2009s_rh_thickness_G-front-sup
1268)	aparc-a2009s_rh_thickness_G-Ins-lg+S-cent-ins
1269)	aparc-a2009s_rh_thickness_G-insular-short
1270)	aparc-a2009s_rh_thickness_G-occipital-middle
1271)	aparc-a2009s_rh_thickness_G-occipital-sup
1272)	aparc-a2009s_rh_thickness_G-oc-temp-lat-fusifor
1273)	aparc-a2009s_rh_thickness_G-oc-temp-med-Lingual
1274)	aparc-a2009s_rh_thickness_G-oc-temp-med-Parahip
1275)	aparc-a2009s_rh_thickness_G-orbital
1276)	aparc-a2009s_rh_thickness_G-pariet-inf-Angular
1277)	aparc-a2009s_rh_thickness_G-pariet-inf-Supramar
1278)	aparc-a2009s_rh_thickness_G-parietal-sup
1279)	aparc-a2009s_rh_thickness_G-postcentral
1280)	aparc-a2009s_rh_thickness_G-precentral
1281)	aparc-a2009s_rh_thickness_G-precuneus
1282)	aparc-a2009s_rh_thickness_G-rectus
1283)	aparc-a2009s_rh_thickness_G-subcallosal
1284)	aparc-a2009s_rh_thickness_G-temp-sup-G-T-transv
1285)	aparc-a2009s_rh_thickness_G-temp-sup-Lateral
1286)	aparc-a2009s_rh_thickness_G-temp-sup-Plan-polar
1287)	aparc-a2009s_rh_thickness_G-temp-sup-Plan-tempo
1288)	aparc-a2009s_rh_thickness_G-temporal-inf
1289)	aparc-a2009s_rh_thickness_G-temporal-middle
1290)	aparc-a2009s_rh_thickness_Lat-Fis-ant-Horizont
1291)	aparc-a2009s_rh_thickness_Lat-Fis-ant-Vertical
1292)	aparc-a2009s_rh_thickness_Lat-Fis-post
1293)	aparc-a2009s_rh_thickness_Pole-occipital
1294)	aparc-a2009s_rh_thickness_Pole-temporal
1295)	aparc-a2009s_rh_thickness_S-calcarine
1296)	aparc-a2009s_rh_thickness_S-central
1297)	aparc-a2009s_rh_thickness_S-cingul-Marginalis
1298)	aparc-a2009s_rh_thickness_S-circular-insula-ant
1299)	aparc-a2009s_rh_thickness_S-circular-insula-inf
1300)	aparc-a2009s_rh_thickness_S-circular-insula-sup

1301)	aparc-a2009s_rh_thickness_S-collat-transv-ant
1302)	aparc-a2009s_rh_thickness_S-collat-transv-post
1303)	aparc-a2009s_rh_thickness_S-front-inf
1304)	aparc-a2009s_rh_thickness_S-front-middle
1305)	aparc-a2009s_rh_thickness_S-front-sup
1306)	aparc-a2009s_rh_thickness_S-interm-prim-Jensen
1307)	aparc-a2009s_rh_thickness_S-intrapariet+P-trans
1308)	aparc-a2009s_rh_thickness_S-oc-middle+Lunatus
1309)	aparc-a2009s_rh_thickness_S-oc-sup+transversal
1310)	aparc-a2009s_rh_thickness_S-occipital-ant
1311)	aparc-a2009s_rh_thickness_S-oc-temp-lat
1312)	aparc-a2009s_rh_thickness_S-oc-temp-med+Lingual
1313)	aparc-a2009s_rh_thickness_S-orbital-lateral
1314)	aparc-a2009s_rh_thickness_S-orbital-med-olfact
1315)	aparc-a2009s_rh_thickness_S-orbital-H-Shaped
1316)	aparc-a2009s_rh_thickness_S-parieto-occipital
1317)	aparc-a2009s_rh_thickness_S-pericallosal
1318)	aparc-a2009s_rh_thickness_S-postcentral
1319)	aparc-a2009s_rh_thickness_S-precentral-inf-part
1320)	aparc-a2009s_rh_thickness_S-precentral-sup-part
1321)	aparc-a2009s_rh_thickness_S-suborbital
1322)	aparc-a2009s_rh_thickness_S-subparietal
1323)	aparc-a2009s_rh_thickness_S-temporal-inf
1324)	aparc-a2009s_rh_thickness_S-temporal-sup
1325)	aparc-a2009s_rh_thickness_S-temporal-transverse
1326)	aseg_global_intensity_3rd-Ventricle
1327)	aseg_global_intensity_4th-Ventricle
1328)	aseg_global_intensity_5th-Ventricle
1329)	aseg_global_intensity_Brain-Stem
1330)	aseg_global_intensity_CSF
1331)	aseg_global_intensity_WM-hypointensities
1332)	aseg_global_intensity_non-WM-hypointensities
1333)	aseg_global_intensity_Optic-Chiasm
1334)	aseg_global_intensity_CC-Posterior
1335)	aseg_global_intensity_CC-Mid-Posterior
1336)	aseg_global_intensity_CC-Central
1337)	aseg_global_intensity_CC-Mid-Anterior
1338)	aseg_global_intensity_CC-Anterior
1339)	aseg_lh_intensity_Lateral-Ventricle
1340)	aseg_lh_intensity_Inf-Lat-Vent
1341)	aseg_lh_intensity_Cerebellum-White-Matter
1342)	aseg_lh_intensity_Cerebellum-Cortex

1343)	aseg_lh_intensity_Thalamus-Proper
1344)	aseg_lh_intensity_Caudate
1345)	aseg_lh_intensity_Putamen
1346)	aseg_lh_intensity_Pallidum
1347)	aseg_lh_intensity_Hippocampus
1348)	aseg_lh_intensity_Amygdala
1349)	aseg_lh_intensity_Accumbens-area
1350)	aseg_lh_intensity_VentralDC
1351)	aseg_lh_intensity_vessel
1352)	aseg_lh_intensity_choroid-plexus
1353)	aseg_rh_intensity_Lateral-Ventricle
1354)	aseg_rh_intensity_Inf-Lat-Vent
1355)	aseg_rh_intensity_Cerebellum-White-Matter
1356)	aseg_rh_intensity_Cerebellum-Cortex
1357)	aseg_rh_intensity_Thalamus-Proper
1358)	aseg_rh_intensity_Caudate
1359)	aseg_rh_intensity_Putamen
1360)	aseg_rh_intensity_Pallidum
1361)	aseg_rh_intensity_Hippocampus
1362)	aseg_rh_intensity_Amygdala
1363)	aseg_rh_intensity_Accumbens-area
1364)	aseg_rh_intensity_VentralDC
1365)	aseg_rh_intensity_vessel
1366)	aseg_rh_intensity_choroid-plexus
1367)	wg_lh_intensity-contrast_unknown
1368)	wg_lh_intensity-contrast_bankssts
1369)	wg_lh_intensity-contrast_caudalanteriorcingulate
1370)	wg_lh_intensity-contrast_caudalmiddlefrontal
1371)	wg_lh_intensity-contrast_cuneus
1372)	wg_lh_intensity-contrast_entorhinal
1373)	wg_lh_intensity-contrast_fusiform
1374)	wg_lh_intensity-contrast_inferiorparietal
1375)	wg_lh_intensity-contrast_inferiortemporal
1376)	wg_lh_intensity-contrast_isthmuscingulate
1377)	wg_lh_intensity-contrast_lateraloccipital
1378)	wg_lh_intensity-contrast_lateralorbitofrontal
1379)	wg_lh_intensity-contrast_lingual
1380)	wg_lh_intensity-contrast_medialorbitofrontal
1381)	wg_lh_intensity-contrast_middletemporal
1382)	wg_lh_intensity-contrast_parahippocampal
1383)	wg_lh_intensity-contrast_paracentral
1384)	wg_lh_intensity-contrast_parsopercularis

1385)	wg_lh_intensity-contrast_parsorbitalis
1386)	wg_lh_intensity-contrast_parstriangularis
1387)	wg_lh_intensity-contrast_pericalcarine
1388)	wg_lh_intensity-contrast_postcentral
1389)	wg_lh_intensity-contrast_posteriorcingulate
1390)	wg_lh_intensity-contrast_precentral
1391)	wg_lh_intensity-contrast_precuneus
1392)	wg_lh_intensity-contrast_rostralanteriorcingulate
1393)	wg_lh_intensity-contrast_rostralmiddlefrontal
1394)	wg_lh_intensity-contrast_superiorfrontal
1395)	wg_lh_intensity-contrast_superiorparietal
1396)	wg_lh_intensity-contrast_superiortemporal
1397)	wg_lh_intensity-contrast_supramarginal
1398)	wg_lh_intensity-contrast_frontalpole
1399)	wg_lh_intensity-contrast_temporalpole
1400)	wg_lh_intensity-contrast_transversetemporal
1401)	wg_lh_intensity-contrast_insula
1402)	wg_rh_intensity-contrast_unknown
1403)	wg_rh_intensity-contrast_bankssts
1404)	wg_rh_intensity-contrast_caudalanteriorcingulate
1405)	wg_rh_intensity-contrast_caudalmiddlefrontal
1406)	wg_rh_intensity-contrast_cuneus
1407)	wg_rh_intensity-contrast_entorhinal
1408)	wg_rh_intensity-contrast_fusiform
1409)	wg_rh_intensity-contrast_inferiorparietal
1410)	wg_rh_intensity-contrast_inferiortemporal
1411)	wg_rh_intensity-contrast_isthmuscingulate
1412)	wg_rh_intensity-contrast_lateraloccipital
1413)	wg_rh_intensity-contrast_lateralorbitofrontal
1414)	wg_rh_intensity-contrast_lingual
1415)	wg_rh_intensity-contrast_medialorbitofrontal
1416)	wg_rh_intensity-contrast_middletemporal
1417)	wg_rh_intensity-contrast_parahippocampal
1418)	wg_rh_intensity-contrast_paracentral
1419)	wg_rh_intensity-contrast_parsopercularis
1420)	wg_rh_intensity-contrast_parsorbitalis
1421)	wg_rh_intensity-contrast_parstriangularis
1422)	wg_rh_intensity-contrast_pericalcarine
1423)	wg_rh_intensity-contrast_postcentral
1424)	wg_rh_intensity-contrast_posteriorcingulate
1425)	wg_rh_intensity-contrast_precentral
1426)	wg_rh_intensity-contrast_precuneus

1427)	wg_rh_intensity-contrast_rostralanteriorcingulate
1428)	wg_rh_intensity-contrast_rostralmiddlefrontal
1429)	wg_rh_intensity-contrast_superiorfrontal
1430)	wg_rh_intensity-contrast_superiorparietal
1431)	wg_rh_intensity-contrast_superiortemporal
1432)	wg_rh_intensity-contrast_supramarginal
1433)	wg_rh_intensity-contrast_frontalpole
1434)	wg_rh_intensity-contrast_temporalpole
1435)	wg_rh_intensity-contrast_transversetemporal
1436)	wg_rh_intensity-contrast_insula
1437)	IDP_T2_FLAIR_BIANCA_WMH_volume
1438)	IDP_T2_FLAIR_BIANCA_periventWMH_volume
1439)	IDP_T2_FLAIR_BIANCA_deepWMH_volume
1440)	IDP_SWI_T2star_left_thalamus
1441)	IDP_SWI_T2star_right_thalamus
1442)	IDP_SWI_T2star_left_caudate
1443)	IDP_SWI_T2star_right_caudate
1444)	IDP_SWI_T2star_left_putamen
1445)	IDP_SWI_T2star_right_putamen
1446)	IDP_SWI_T2star_left_pallidum
1447)	IDP_SWI_T2star_right_pallidum
1448)	IDP_SWI_T2star_left_hippocampus
1449)	IDP_SWI_T2star_right_hippocampus
1450)	IDP_SWI_T2star_left_amygdala
1451)	IDP_SWI_T2star_right_amygdala
1452)	IDP_SWI_T2star_left_accumbens
1453)	IDP_SWI_T2star_right_accumbens
1454)	IDP_dMRI_TBSS_FA_Middle_cerebellar_peduncle
1455)	IDP_dMRI_TBSS_FA_Pontine_crossing_tract
1456)	IDP_dMRI_TBSS_FA_Genu_of_corpus_callosum
1457)	IDP_dMRI_TBSS_FA_Body_of_corpus_callosum
1458)	IDP_dMRI_TBSS_FA_Splenium_of_corpus_callosum
1459)	IDP_dMRI_TBSS_FA_Fornix
1460)	IDP_dMRI_TBSS_FA_Corticospinal_tract_R
1461)	IDP_dMRI_TBSS_FA_Corticospinal_tract_L
1462)	IDP_dMRI_TBSS_FA_Medial_lemniscus_R
1463)	IDP_dMRI_TBSS_FA_Medial_lemniscus_L
1464)	IDP_dMRI_TBSS_FA_Inferior_cerebellar_peduncle_R
1465)	IDP_dMRI_TBSS_FA_Inferior_cerebellar_peduncle_L
1466)	IDP_dMRI_TBSS_FA_Superior_cerebellar_peduncle_R
1467)	IDP_dMRI_TBSS_FA_Superior_cerebellar_peduncle_L
1468)	IDP_dMRI_TBSS_FA_Cerebral_peduncle_R

1469)	IDP_dMRI_TBSS_FA_Cerebral_peduncle_L
1470)	IDP_dMRI_TBSS_FA_Anterior_limb_of_internal_capsule_R
1471)	IDP_dMRI_TBSS_FA_Anterior_limb_of_internal_capsule_L
1472)	IDP_dMRI_TBSS_FA_Posterior_limb_of_internal_capsule_R
1473)	IDP_dMRI_TBSS_FA_Posterior_limb_of_internal_capsule_L
1474)	IDP_dMRI_TBSS_FA_Retrolenticular_part_of_internal_capsule_R
1475)	IDP_dMRI_TBSS_FA_Retrolenticular_part_of_internal_capsule_L
1476)	IDP_dMRI_TBSS_FA_Anterior_corona_radiata_R
1477)	IDP_dMRI_TBSS_FA_Anterior_corona_radiata_L
1478)	IDP_dMRI_TBSS_FA_Superior_corona_radiata_R
1479)	IDP_dMRI_TBSS_FA_Superior_corona_radiata_L
1480)	IDP_dMRI_TBSS_FA_Posterior_corona_radiata_R
1481)	IDP_dMRI_TBSS_FA_Posterior_corona_radiata_L
1482)	IDP_dMRI_TBSS_FA_Posterior_thalamic_radiation_R
1483)	IDP_dMRI_TBSS_FA_Posterior_thalamic_radiation_L
1484)	IDP_dMRI_TBSS_FA_Sagittal_stratum_R
1485)	IDP_dMRI_TBSS_FA_Sagittal_stratum_L
1486)	IDP_dMRI_TBSS_FA_External_capsule_R
1487)	IDP_dMRI_TBSS_FA_External_capsule_L
1488)	IDP_dMRI_TBSS_FA_Cingulum_cingulate_gyrus_R
1489)	IDP_dMRI_TBSS_FA_Cingulum_cingulate_gyrus_L
1490)	IDP_dMRI_TBSS_FA_Cingulum_hippocampus_R
1491)	IDP_dMRI_TBSS_FA_Cingulum_hippocampus_L
1492)	IDP_dMRI_TBSS_FA_Fornix_cres+Stria_terminalis_R
1493)	IDP_dMRI_TBSS_FA_Fornix_cres+Stria_terminalis_L
1494)	IDP_dMRI_TBSS_FA_Superior_longitudinal_fasciculus_R
1495)	IDP_dMRI_TBSS_FA_Superior_longitudinal_fasciculus_L
1496)	IDP_dMRI_TBSS_FA_Superior_fronto-occipital_fasciculus_R
1497)	IDP_dMRI_TBSS_FA_Superior_fronto-occipital_fasciculus_L
1498)	IDP_dMRI_TBSS_FA_Uncinate_fasciculus_R
1499)	IDP_dMRI_TBSS_FA_Uncinate_fasciculus_L
1500)	IDP_dMRI_TBSS_FA_Tapetum_R
1501)	IDP_dMRI_TBSS_FA_Tapetum_L
1502)	IDP_dMRI_ProbtrackX_FA_ar_l
1503)	IDP_dMRI_ProbtrackX_FA_ar_r
1504)	IDP_dMRI_ProbtrackX_FA_atr_l
1505)	IDP_dMRI_ProbtrackX_FA_atr_r
1506)	IDP_dMRI_ProbtrackX_FA_cgc_l
1507)	IDP_dMRI_ProbtrackX_FA_cgc_r
1508)	IDP_dMRI_ProbtrackX_FA_cgh_l
1509)	IDP_dMRI_ProbtrackX_FA_cgh_r
1510)	IDP_dMRI_ProbtrackX_FA_cst_l

1511)	IDP_dMRI_ProbtrackX_FA_cst_r
1512)	IDP_dMRI_ProbtrackX_FA_fma
1513)	IDP_dMRI_ProbtrackX_FA_fmi
1514)	IDP_dMRI_ProbtrackX_FA_ifo_l
1515)	IDP_dMRI_ProbtrackX_FA_ifo_r
1516)	IDP_dMRI_ProbtrackX_FA_ilf_l
1517)	IDP_dMRI_ProbtrackX_FA_ilf_r
1518)	IDP_dMRI_ProbtrackX_FA_mcp
1519)	IDP_dMRI_ProbtrackX_FA_ml_l
1520)	IDP_dMRI_ProbtrackX_FA_ml_r
1521)	IDP_dMRI_ProbtrackX_FA_ptr_l
1522)	IDP_dMRI_ProbtrackX_FA_ptr_r
1523)	IDP_dMRI_ProbtrackX_FA_slf_l
1524)	IDP_dMRI_ProbtrackX_FA_slf_r
1525)	IDP_dMRI_ProbtrackX_FA_str_l
1526)	IDP_dMRI_ProbtrackX_FA_str_r
1527)	IDP_dMRI_ProbtrackX_FA_unc_l
1528)	IDP_dMRI_ProbtrackX_FA_unc_r
1529)	IDP_dMRI_TBSS_MO_Middle_cerebellar_peduncle
1530)	IDP_dMRI_TBSS_MO_Pontine_crossing_tract
1531)	IDP_dMRI_TBSS_MO_Genu_of_corpus_callosum
1532)	IDP_dMRI_TBSS_MO_Body_of_corpus_callosum
1533)	IDP_dMRI_TBSS_MO_Splenium_of_corpus_callosum
1534)	IDP_dMRI_TBSS_MO_Fornix
1535)	IDP_dMRI_TBSS_MO_Corticospinal_tract_R
1536)	IDP_dMRI_TBSS_MO_Corticospinal_tract_L
1537)	IDP_dMRI_TBSS_MO_Medial_lemniscus_R
1538)	IDP_dMRI_TBSS_MO_Medial_lemniscus_L
1539)	IDP_dMRI_TBSS_MO_Inferior_cerebellar_peduncle_R
1540)	IDP_dMRI_TBSS_MO_Inferior_cerebellar_peduncle_L
1541)	IDP_dMRI_TBSS_MO_Superior_cerebellar_peduncle_R
1542)	IDP_dMRI_TBSS_MO_Superior_cerebellar_peduncle_L
1543)	IDP_dMRI_TBSS_MO_Cerebral_peduncle_R
1544)	IDP_dMRI_TBSS_MO_Cerebral_peduncle_L
1545)	IDP_dMRI_TBSS_MO_Anterior_limb_of_internal_capsule_R
1546)	IDP_dMRI_TBSS_MO_Anterior_limb_of_internal_capsule_L
1547)	IDP_dMRI_TBSS_MO_Posterior_limb_of_internal_capsule_R
1548)	IDP_dMRI_TBSS_MO_Posterior_limb_of_internal_capsule_L
1549)	IDP_dMRI_TBSS_MO_Retrolenticular_part_of_internal_capsule_R
1550)	IDP_dMRI_TBSS_MO_Retrolenticular_part_of_internal_capsule_L
1551)	IDP_dMRI_TBSS_MO_Anterior_corona_radiata_R
1552)	IDP_dMRI_TBSS_MO_Anterior_corona_radiata_L

1553)	IDP_dMRI_TBSS_MO_Superior_corona_radiata_R
1554)	IDP_dMRI_TBSS_MO_Superior_corona_radiata_L
1555)	IDP_dMRI_TBSS_MO_Posterior_corona_radiata_R
1556)	IDP_dMRI_TBSS_MO_Posterior_corona_radiata_L
1557)	IDP_dMRI_TBSS_MO_Posterior_thalamic_radiation_R
1558)	IDP_dMRI_TBSS_MO_Posterior_thalamic_radiation_L
1559)	IDP_dMRI_TBSS_MO_Sagittal_stratum_R
1560)	IDP_dMRI_TBSS_MO_Sagittal_stratum_L
1561)	IDP_dMRI_TBSS_MO_External_capsule_R
1562)	IDP_dMRI_TBSS_MO_External_capsule_L
1563)	IDP_dMRI_TBSS_MO_Cingulum_cingulate_gyrus_R
1564)	IDP_dMRI_TBSS_MO_Cingulum_cingulate_gyrus_L
1565)	IDP_dMRI_TBSS_MO_Cingulum_hippocampus_R
1566)	IDP_dMRI_TBSS_MO_Cingulum_hippocampus_L
1567)	IDP_dMRI_TBSS_MO_Fornix_cres+Stria_terminalis_R
1568)	IDP_dMRI_TBSS_MO_Fornix_cres+Stria_terminalis_L
1569)	IDP_dMRI_TBSS_MO_Superior_longitudinal_fasciculus_R
1570)	IDP_dMRI_TBSS_MO_Superior_longitudinal_fasciculus_L
1571)	IDP_dMRI_TBSS_MO_Superior_fronto-occipital_fasciculus_R
1572)	IDP_dMRI_TBSS_MO_Superior_fronto-occipital_fasciculus_L
1573)	IDP_dMRI_TBSS_MO_Uncinate_fasciculus_R
1574)	IDP_dMRI_TBSS_MO_Uncinate_fasciculus_L
1575)	IDP_dMRI_TBSS_MO_Tapetum_R
1576)	IDP_dMRI_TBSS_MO_Tapetum_L
1577)	IDP_dMRI_ProbtrackX_MO_ar_l
1578)	IDP_dMRI_ProbtrackX_MO_ar_r
1579)	IDP_dMRI_ProbtrackX_MO_atr_l
1580)	IDP_dMRI_ProbtrackX_MO_atr_r
1581)	IDP_dMRI_ProbtrackX_MO_cgc_l
1582)	IDP_dMRI_ProbtrackX_MO_cgc_r
1583)	IDP_dMRI_ProbtrackX_MO_cgh_l
1584)	IDP_dMRI_ProbtrackX_MO_cgh_r
1585)	IDP_dMRI_ProbtrackX_MO_cst_l
1586)	IDP_dMRI_ProbtrackX_MO_cst_r
1587)	IDP_dMRI_ProbtrackX_MO_fma
1588)	IDP_dMRI_ProbtrackX_MO_fmi
1589)	IDP_dMRI_ProbtrackX_MO_ifo_l
1590)	IDP_dMRI_ProbtrackX_MO_ifo_r
1591)	IDP_dMRI_ProbtrackX_MO_ilf_l
1592)	IDP_dMRI_ProbtrackX_MO_ilf_r
1593)	IDP_dMRI_ProbtrackX_MO_mcp
1594)	IDP_dMRI_ProbtrackX_MO_ml_l

1595)	IDP_dMRI_ProbtrackX_MO_ml_r
1596)	IDP_dMRI_ProbtrackX_MO_ptr_l
1597)	IDP_dMRI_ProbtrackX_MO_ptr_r
1598)	IDP_dMRI_ProbtrackX_MO_slf_l
1599)	IDP_dMRI_ProbtrackX_MO_slf_r
1600)	IDP_dMRI_ProbtrackX_MO_str_l
1601)	IDP_dMRI_ProbtrackX_MO_str_r
1602)	IDP_dMRI_ProbtrackX_MO_unc_l
1603)	IDP_dMRI_ProbtrackX_MO_unc_r
1604)	IDP_dMRI_TBSS_MD_Middle_cerebellar_peduncle
1605)	IDP_dMRI_TBSS_MD_Pontine_crossing_tract
1606)	IDP_dMRI_TBSS_MD_Genu_of_corpus_callosum
1607)	IDP_dMRI_TBSS_MD_Body_of_corpus_callosum
1608)	IDP_dMRI_TBSS_MD_Splenium_of_corpus_callosum
1609)	IDP_dMRI_TBSS_MD_Fornix
1610)	IDP_dMRI_TBSS_MD_Corticospinal_tract_R
1611)	IDP_dMRI_TBSS_MD_Corticospinal_tract_L
1612)	IDP_dMRI_TBSS_MD_Medial_lemniscus_R
1613)	IDP_dMRI_TBSS_MD_Medial_lemniscus_L
1614)	IDP_dMRI_TBSS_MD_Inferior_cerebellar_peduncle_R
1615)	IDP_dMRI_TBSS_MD_Inferior_cerebellar_peduncle_L
1616)	IDP_dMRI_TBSS_MD_Superior_cerebellar_peduncle_R
1617)	IDP_dMRI_TBSS_MD_Superior_cerebellar_peduncle_L
1618)	IDP_dMRI_TBSS_MD_Cerebral_peduncle_R
1619)	IDP_dMRI_TBSS_MD_Cerebral_peduncle_L
1620)	IDP_dMRI_TBSS_MD_Anterior_limb_of_internal_capsule_R
1621)	IDP_dMRI_TBSS_MD_Anterior_limb_of_internal_capsule_L
1622)	IDP_dMRI_TBSS_MD_Posterior_limb_of_internal_capsule_R
1623)	IDP_dMRI_TBSS_MD_Posterior_limb_of_internal_capsule_L
1624)	IDP_dMRI_TBSS_MD_Retrolenticular_part_of_internal_capsule_R
1625)	IDP_dMRI_TBSS_MD_Retrolenticular_part_of_internal_capsule_L
1626)	IDP_dMRI_TBSS_MD_Anterior_corona_radiata_R
1627)	IDP_dMRI_TBSS_MD_Anterior_corona_radiata_L
1628)	IDP_dMRI_TBSS_MD_Superior_corona_radiata_R
1629)	IDP_dMRI_TBSS_MD_Superior_corona_radiata_L
1630)	IDP_dMRI_TBSS_MD_Posterior_corona_radiata_R
1631)	IDP_dMRI_TBSS_MD_Posterior_corona_radiata_L
1632)	IDP_dMRI_TBSS_MD_Posterior_thalamic_radiation_R
1633)	IDP_dMRI_TBSS_MD_Posterior_thalamic_radiation_L
1634)	IDP_dMRI_TBSS_MD_Sagittal_stratum_R
1635)	IDP_dMRI_TBSS_MD_Sagittal_stratum_L
1636)	IDP_dMRI_TBSS_MD_External_capsule_R

1637)	IDP_dMRI_TBSS_MD_External_capsule_L
1638)	IDP_dMRI_TBSS_MD_Cingulum_cingulate_gyrus_R
1639)	IDP_dMRI_TBSS_MD_Cingulum_cingulate_gyrus_L
1640)	IDP_dMRI_TBSS_MD_Cingulum_hippocampus_R
1641)	IDP_dMRI_TBSS_MD_Cingulum_hippocampus_L
1642)	IDP_dMRI_TBSS_MD_Fornix_cres+Stria_terminalis_R
1643)	IDP_dMRI_TBSS_MD_Fornix_cres+Stria_terminalis_L
1644)	IDP_dMRI_TBSS_MD_Superior_longitudinal_fasciculus_R
1645)	IDP_dMRI_TBSS_MD_Superior_longitudinal_fasciculus_L
1646)	IDP_dMRI_TBSS_MD_Superior_fronto-occipital_fasciculus_R
1647)	IDP_dMRI_TBSS_MD_Superior_fronto-occipital_fasciculus_L
1648)	IDP_dMRI_TBSS_MD_Uncinate_fasciculus_R
1649)	IDP_dMRI_TBSS_MD_Uncinate_fasciculus_L
1650)	IDP_dMRI_TBSS_MD_Tapetum_R
1651)	IDP_dMRI_TBSS_MD_Tapetum_L
1652)	IDP_dMRI_TBSS_L1_Middle_cerebellar_peduncle
1653)	IDP_dMRI_TBSS_L1_Pontine_crossing_tract
1654)	IDP_dMRI_TBSS_L1_Genu_of_corpus_callosum
1655)	IDP_dMRI_TBSS_L1_Body_of_corpus_callosum
1656)	IDP_dMRI_TBSS_L1_Splenium_of_corpus_callosum
1657)	IDP_dMRI_TBSS_L1_Fornix
1658)	IDP_dMRI_TBSS_L1_Corticospinal_tract_R
1659)	IDP_dMRI_TBSS_L1_Corticospinal_tract_L
1660)	IDP_dMRI_TBSS_L1_Medial_lemniscus_R
1661)	IDP_dMRI_TBSS_L1_Medial_lemniscus_L
1662)	IDP_dMRI_TBSS_L1_Inferior_cerebellar_peduncle_R
1663)	IDP_dMRI_TBSS_L1_Inferior_cerebellar_peduncle_L
1664)	IDP_dMRI_TBSS_L1_Superior_cerebellar_peduncle_R
1665)	IDP_dMRI_TBSS_L1_Superior_cerebellar_peduncle_L
1666)	IDP_dMRI_TBSS_L1_Cerebral_peduncle_R
1667)	IDP_dMRI_TBSS_L1_Cerebral_peduncle_L
1668)	IDP_dMRI_TBSS_L1_Anterior_limb_of_internal_capsule_R
1669)	IDP_dMRI_TBSS_L1_Anterior_limb_of_internal_capsule_L
1670)	IDP_dMRI_TBSS_L1_Posterior_limb_of_internal_capsule_R
1671)	IDP_dMRI_TBSS_L1_Posterior_limb_of_internal_capsule_L
1672)	IDP_dMRI_TBSS_L1_Retrolenticular_part_of_internal_capsule_R
1673)	IDP_dMRI_TBSS_L1_Retrolenticular_part_of_internal_capsule_L
1674)	IDP_dMRI_TBSS_L1_Anterior_corona_radiata_R
1675)	IDP_dMRI_TBSS_L1_Anterior_corona_radiata_L
1676)	IDP_dMRI_TBSS_L1_Superior_corona_radiata_R
1677)	IDP_dMRI_TBSS_L1_Superior_corona_radiata_L
1678)	IDP_dMRI_TBSS_L1_Posterior_corona_radiata_R

1679)	IDP_dMRI_TBSS_L1_Posterior_corona_radiata_L
1680)	IDP_dMRI_TBSS_L1_Posterior_thalamic_radiation_R
1681)	IDP_dMRI_TBSS_L1_Posterior_thalamic_radiation_L
1682)	IDP_dMRI_TBSS_L1_Sagittal_stratum_R
1683)	IDP_dMRI_TBSS_L1_Sagittal_stratum_L
1684)	IDP_dMRI_TBSS_L1_External_capsule_R
1685)	IDP_dMRI_TBSS_L1_External_capsule_L
1686)	IDP_dMRI_TBSS_L1_Cingulum_cingulate_gyrus_R
1687)	IDP_dMRI_TBSS_L1_Cingulum_cingulate_gyrus_L
1688)	IDP_dMRI_TBSS_L1_Cingulum_hippocampus_R
1689)	IDP_dMRI_TBSS_L1_Cingulum_hippocampus_L
1690)	IDP_dMRI_TBSS_L1_Fornix_cres+Stria_terminalis_R
1691)	IDP_dMRI_TBSS_L1_Fornix_cres+Stria_terminalis_L
1692)	IDP_dMRI_TBSS_L1_Superior_longitudinal_fasciculus_R
1693)	IDP_dMRI_TBSS_L1_Superior_longitudinal_fasciculus_L
1694)	IDP_dMRI_TBSS_L1_Superior_fronto-occipital_fasciculus_R
1695)	IDP_dMRI_TBSS_L1_Superior_fronto-occipital_fasciculus_L
1696)	IDP_dMRI_TBSS_L1_Uncinate_fasciculus_R
1697)	IDP_dMRI_TBSS_L1_Uncinate_fasciculus_L
1698)	IDP_dMRI_TBSS_L1_Tapetum_R
1699)	IDP_dMRI_TBSS_L1_Tapetum_L
1700)	IDP_dMRI_TBSS_L2_Middle_cerebellar_peduncle
1701)	IDP_dMRI_TBSS_L2_Pontine_crossing_tract
1702)	IDP_dMRI_TBSS_L2_Genu_of_corpus_callosum
1703)	IDP_dMRI_TBSS_L2_Body_of_corpus_callosum
1704)	IDP_dMRI_TBSS_L2_Splenium_of_corpus_callosum
1705)	IDP_dMRI_TBSS_L2_Fornix
1706)	IDP_dMRI_TBSS_L2_Corticospinal_tract_R
1707)	IDP_dMRI_TBSS_L2_Corticospinal_tract_L
1708)	IDP_dMRI_TBSS_L2_Medial_lemniscus_R
1709)	IDP_dMRI_TBSS_L2_Medial_lemniscus_L
1710)	IDP_dMRI_TBSS_L2_Inferior_cerebellar_peduncle_R
1711)	IDP_dMRI_TBSS_L2_Inferior_cerebellar_peduncle_L
1712)	IDP_dMRI_TBSS_L2_Superior_cerebellar_peduncle_R
1713)	IDP_dMRI_TBSS_L2_Superior_cerebellar_peduncle_L
1714)	IDP_dMRI_TBSS_L2_Cerebral_peduncle_R
1715)	IDP_dMRI_TBSS_L2_Cerebral_peduncle_L
1716)	IDP_dMRI_TBSS_L2_Anterior_limb_of_internal_capsule_R
1717)	IDP_dMRI_TBSS_L2_Anterior_limb_of_internal_capsule_L
1718)	IDP_dMRI_TBSS_L2_Posterior_limb_of_internal_capsule_R
1719)	IDP_dMRI_TBSS_L2_Posterior_limb_of_internal_capsule_L
1720)	IDP_dMRI_TBSS_L2_Retrolenticular_part_of_internal_capsule_R

1721)	IDP_dMRI_TBSS_L2_Retrolenticular_part_of_internal_capsule_L
1722)	IDP_dMRI_TBSS_L2_Anterior_corona_radiata_R
1723)	IDP_dMRI_TBSS_L2_Anterior_corona_radiata_L
1724)	IDP_dMRI_TBSS_L2_Superior_corona_radiata_R
1725)	IDP_dMRI_TBSS_L2_Superior_corona_radiata_L
1726)	IDP_dMRI_TBSS_L2_Posterior_corona_radiata_R
1727)	IDP_dMRI_TBSS_L2_Posterior_corona_radiata_L
1728)	IDP_dMRI_TBSS_L2_Posterior_thalamic_radiation_R
1729)	IDP_dMRI_TBSS_L2_Posterior_thalamic_radiation_L
1730)	IDP_dMRI_TBSS_L2_Sagittal_stratum_R
1731)	IDP_dMRI_TBSS_L2_Sagittal_stratum_L
1732)	IDP_dMRI_TBSS_L2_External_capsule_R
1733)	IDP_dMRI_TBSS_L2_External_capsule_L
1734)	IDP_dMRI_TBSS_L2_Cingulum_cingulate_gyrus_R
1735)	IDP_dMRI_TBSS_L2_Cingulum_cingulate_gyrus_L
1736)	IDP_dMRI_TBSS_L2_Cingulum_hippocampus_R
1737)	IDP_dMRI_TBSS_L2_Cingulum_hippocampus_L
1738)	IDP_dMRI_TBSS_L2_Fornix_cres+Stria_terminalis_R
1739)	IDP_dMRI_TBSS_L2_Fornix_cres+Stria_terminalis_L
1740)	IDP_dMRI_TBSS_L2_Superior_longitudinal_fasciculus_R
1741)	IDP_dMRI_TBSS_L2_Superior_longitudinal_fasciculus_L
1742)	IDP_dMRI_TBSS_L2_Superior_fronto-occipital_fasciculus_R
1743)	IDP_dMRI_TBSS_L2_Superior_fronto-occipital_fasciculus_L
1744)	IDP_dMRI_TBSS_L2_Uncinate_fasciculus_R
1745)	IDP_dMRI_TBSS_L2_Uncinate_fasciculus_L
1746)	IDP_dMRI_TBSS_L2_Tapetum_R
1747)	IDP_dMRI_TBSS_L2_Tapetum_L
1748)	IDP_dMRI_TBSS_L3_Middle_cerebellar_peduncle
1749)	IDP_dMRI_TBSS_L3_Pontine_crossing_tract
1750)	IDP_dMRI_TBSS_L3_Genu_of_corpus_callosum
1751)	IDP_dMRI_TBSS_L3_Body_of_corpus_callosum
1752)	IDP_dMRI_TBSS_L3_Splenium_of_corpus_callosum
1753)	IDP_dMRI_TBSS_L3_Fornix
1754)	IDP_dMRI_TBSS_L3_Corticospinal_tract_R
1755)	IDP_dMRI_TBSS_L3_Corticospinal_tract_L
1756)	IDP_dMRI_TBSS_L3_Medial_lemniscus_R
1757)	IDP_dMRI_TBSS_L3_Medial_lemniscus_L
1758)	IDP_dMRI_TBSS_L3_Inferior_cerebellar_peduncle_R
1759)	IDP_dMRI_TBSS_L3_Inferior_cerebellar_peduncle_L
1760)	IDP_dMRI_TBSS_L3_Superior_cerebellar_peduncle_R
1761)	IDP_dMRI_TBSS_L3_Superior_cerebellar_peduncle_L
1762)	IDP_dMRI_TBSS_L3_Cerebral_peduncle_R

1763)	IDP_dMRI_TBSS_L3_Cerebral_peduncle_L
1764)	IDP_dMRI_TBSS_L3_Anterior_limb_of_internal_capsule_R
1765)	IDP_dMRI_TBSS_L3_Anterior_limb_of_internal_capsule_L
1766)	IDP_dMRI_TBSS_L3_Posterior_limb_of_internal_capsule_R
1767)	IDP_dMRI_TBSS_L3_Posterior_limb_of_internal_capsule_L
1768)	IDP_dMRI_TBSS_L3_Retrolenticular_part_of_internal_capsule_R
1769)	IDP_dMRI_TBSS_L3_Retrolenticular_part_of_internal_capsule_L
1770)	IDP_dMRI_TBSS_L3_Anterior_corona_radiata_R
1771)	IDP_dMRI_TBSS_L3_Anterior_corona_radiata_L
1772)	IDP_dMRI_TBSS_L3_Superior_corona_radiata_R
1773)	IDP_dMRI_TBSS_L3_Superior_corona_radiata_L
1774)	IDP_dMRI_TBSS_L3_Posterior_corona_radiata_R
1775)	IDP_dMRI_TBSS_L3_Posterior_corona_radiata_L
1776)	IDP_dMRI_TBSS_L3_Posterior_thalamic_radiation_R
1777)	IDP_dMRI_TBSS_L3_Posterior_thalamic_radiation_L
1778)	IDP_dMRI_TBSS_L3_Sagittal_stratum_R
1779)	IDP_dMRI_TBSS_L3_Sagittal_stratum_L
1780)	IDP_dMRI_TBSS_L3_External_capsule_R
1781)	IDP_dMRI_TBSS_L3_External_capsule_L
1782)	IDP_dMRI_TBSS_L3_Cingulum_cingulate_gyrus_R
1783)	IDP_dMRI_TBSS_L3_Cingulum_cingulate_gyrus_L
1784)	IDP_dMRI_TBSS_L3_Cingulum_hippocampus_R
1785)	IDP_dMRI_TBSS_L3_Cingulum_hippocampus_L
1786)	IDP_dMRI_TBSS_L3_Fornix_cres+Stria_terminalis_R
1787)	IDP_dMRI_TBSS_L3_Fornix_cres+Stria_terminalis_L
1788)	IDP_dMRI_TBSS_L3_Superior_longitudinal_fasciculus_R
1789)	IDP_dMRI_TBSS_L3_Superior_longitudinal_fasciculus_L
1790)	IDP_dMRI_TBSS_L3_Superior_fronto-occipital_fasciculus_R
1791)	IDP_dMRI_TBSS_L3_Superior_fronto-occipital_fasciculus_L
1792)	IDP_dMRI_TBSS_L3_Uncinate_fasciculus_R
1793)	IDP_dMRI_TBSS_L3_Uncinate_fasciculus_L
1794)	IDP_dMRI_TBSS_L3_Tapetum_R
1795)	IDP_dMRI_TBSS_L3_Tapetum_L
1796)	IDP_dMRI_ProbtrackX_MD_ar_l
1797)	IDP_dMRI_ProbtrackX_MD_ar_r
1798)	IDP_dMRI_ProbtrackX_MD_atr_l
1799)	IDP_dMRI_ProbtrackX_MD_atr_r
1800)	IDP_dMRI_ProbtrackX_MD_cgc_l
1801)	IDP_dMRI_ProbtrackX_MD_cgc_r
1802)	IDP_dMRI_ProbtrackX_MD_cgh_l
1803)	IDP_dMRI_ProbtrackX_MD_cgh_r
1804)	IDP_dMRI_ProbtrackX_MD_cst_l

1805)	IDP_dMRI_ProbtrackX_MD_cst_r
1806)	IDP_dMRI_ProbtrackX_MD_fma
1807)	IDP_dMRI_ProbtrackX_MD_fmi
1808)	IDP_dMRI_ProbtrackX_MD_ifo_l
1809)	IDP_dMRI_ProbtrackX_MD_ifo_r
1810)	IDP_dMRI_ProbtrackX_MD_ilf_l
1811)	IDP_dMRI_ProbtrackX_MD_ilf_r
1812)	IDP_dMRI_ProbtrackX_MD_mcp
1813)	IDP_dMRI_ProbtrackX_MD_ml_l
1814)	IDP_dMRI_ProbtrackX_MD_ml_r
1815)	IDP_dMRI_ProbtrackX_MD_ptr_l
1816)	IDP_dMRI_ProbtrackX_MD_ptr_r
1817)	IDP_dMRI_ProbtrackX_MD_slf_l
1818)	IDP_dMRI_ProbtrackX_MD_slf_r
1819)	IDP_dMRI_ProbtrackX_MD_str_l
1820)	IDP_dMRI_ProbtrackX_MD_str_r
1821)	IDP_dMRI_ProbtrackX_MD_unc_l
1822)	IDP_dMRI_ProbtrackX_MD_unc_r
1823)	IDP_dMRI_ProbtrackX_L1_ar_l
1824)	IDP_dMRI_ProbtrackX_L1_ar_r
1825)	IDP_dMRI_ProbtrackX_L1_atr_l
1826)	IDP_dMRI_ProbtrackX_L1_atr_r
1827)	IDP_dMRI_ProbtrackX_L1_cgc_l
1828)	IDP_dMRI_ProbtrackX_L1_cgc_r
1829)	IDP_dMRI_ProbtrackX_L1_cgh_l
1830)	IDP_dMRI_ProbtrackX_L1_cgh_r
1831)	IDP_dMRI_ProbtrackX_L1_cst_l
1832)	IDP_dMRI_ProbtrackX_L1_cst_r
1833)	IDP_dMRI_ProbtrackX_L1_fma
1834)	IDP_dMRI_ProbtrackX_L1_fmi
1835)	IDP_dMRI_ProbtrackX_L1_ifo_l
1836)	IDP_dMRI_ProbtrackX_L1_ifo_r
1837)	IDP_dMRI_ProbtrackX_L1_ilf_l
1838)	IDP_dMRI_ProbtrackX_L1_ilf_r
1839)	IDP_dMRI_ProbtrackX_L1_mcp
1840)	IDP_dMRI_ProbtrackX_L1_ml_l
1841)	IDP_dMRI_ProbtrackX_L1_ml_r
1842)	IDP_dMRI_ProbtrackX_L1_ptr_l
1843)	IDP_dMRI_ProbtrackX_L1_ptr_r
1844)	IDP_dMRI_ProbtrackX_L1_slf_l
1845)	IDP_dMRI_ProbtrackX_L1_slf_r
1846)	IDP_dMRI_ProbtrackX_L1_str_l

1847)	IDP_dMRI_ProbtrackX_L1_str_r
1848)	IDP_dMRI_ProbtrackX_L1_unc_l
1849)	IDP_dMRI_ProbtrackX_L1_unc_r
1850)	IDP_dMRI_ProbtrackX_L2_ar_l
1851)	IDP_dMRI_ProbtrackX_L2_ar_r
1852)	IDP_dMRI_ProbtrackX_L2_atr_l
1853)	IDP_dMRI_ProbtrackX_L2_atr_r
1854)	IDP_dMRI_ProbtrackX_L2_cgc_l
1855)	IDP_dMRI_ProbtrackX_L2_cgc_r
1856)	IDP_dMRI_ProbtrackX_L2_cgh_l
1857)	IDP_dMRI_ProbtrackX_L2_cgh_r
1858)	IDP_dMRI_ProbtrackX_L2_cst_l
1859)	IDP_dMRI_ProbtrackX_L2_cst_r
1860)	IDP_dMRI_ProbtrackX_L2_fma
1861)	IDP_dMRI_ProbtrackX_L2_fmi
1862)	IDP_dMRI_ProbtrackX_L2_ifo_l
1863)	IDP_dMRI_ProbtrackX_L2_ifo_r
1864)	IDP_dMRI_ProbtrackX_L2_ilf_l
1865)	IDP_dMRI_ProbtrackX_L2_ilf_r
1866)	IDP_dMRI_ProbtrackX_L2_mcp
1867)	IDP_dMRI_ProbtrackX_L2_ml_l
1868)	IDP_dMRI_ProbtrackX_L2_ml_r
1869)	IDP_dMRI_ProbtrackX_L2_ptr_l
1870)	IDP_dMRI_ProbtrackX_L2_ptr_r
1871)	IDP_dMRI_ProbtrackX_L2_slf_l
1872)	IDP_dMRI_ProbtrackX_L2_slf_r
1873)	IDP_dMRI_ProbtrackX_L2_str_l
1874)	IDP_dMRI_ProbtrackX_L2_str_r
1875)	IDP_dMRI_ProbtrackX_L2_unc_l
1876)	IDP_dMRI_ProbtrackX_L2_unc_r
1877)	IDP_dMRI_ProbtrackX_L3_ar_l
1878)	IDP_dMRI_ProbtrackX_L3_ar_r
1879)	IDP_dMRI_ProbtrackX_L3_atr_l
1880)	IDP_dMRI_ProbtrackX_L3_atr_r
1881)	IDP_dMRI_ProbtrackX_L3_cgc_l
1882)	IDP_dMRI_ProbtrackX_L3_cgc_r
1883)	IDP_dMRI_ProbtrackX_L3_cgh_l
1884)	IDP_dMRI_ProbtrackX_L3_cgh_r
1885)	IDP_dMRI_ProbtrackX_L3_cst_l
1886)	IDP_dMRI_ProbtrackX_L3_cst_r
1887)	IDP_dMRI_ProbtrackX_L3_fma
1888)	IDP_dMRI_ProbtrackX_L3_fmi

1889)	IDP_dMRI_ProbtrackX_L3_ifo_l
1890)	IDP_dMRI_ProbtrackX_L3_ifo_r
1891)	IDP_dMRI_ProbtrackX_L3_ilf_l
1892)	IDP_dMRI_ProbtrackX_L3_ilf_r
1893)	IDP_dMRI_ProbtrackX_L3_mcp
1894)	IDP_dMRI_ProbtrackX_L3_ml_l
1895)	IDP_dMRI_ProbtrackX_L3_ml_r
1896)	IDP_dMRI_ProbtrackX_L3_ptr_l
1897)	IDP_dMRI_ProbtrackX_L3_ptr_r
1898)	IDP_dMRI_ProbtrackX_L3_slf_l
1899)	IDP_dMRI_ProbtrackX_L3_slf_r
1900)	IDP_dMRI_ProbtrackX_L3_str_l
1901)	IDP_dMRI_ProbtrackX_L3_str_r
1902)	IDP_dMRI_ProbtrackX_L3_unc_l
1903)	IDP_dMRI_ProbtrackX_L3_unc_r
1904)	IDP_dMRI_TBSS_ICVF_Middle_cerebellar_peduncle
1905)	IDP_dMRI_TBSS_ICVF_Pontine_crossing_tract
1906)	IDP_dMRI_TBSS_ICVF_Genu_of_corpus_callosum
1907)	IDP_dMRI_TBSS_ICVF_Body_of_corpus_callosum
1908)	IDP_dMRI_TBSS_ICVF_Splenium_of_corpus_callosum
1909)	IDP_dMRI_TBSS_ICVF_Fornix
1910)	IDP_dMRI_TBSS_ICVF_Corticospinal_tract_R
1911)	IDP_dMRI_TBSS_ICVF_Corticospinal_tract_L
1912)	IDP_dMRI_TBSS_ICVF_Medial_lemniscus_R
1913)	IDP_dMRI_TBSS_ICVF_Medial_lemniscus_L
1914)	IDP_dMRI_TBSS_ICVF_Inferior_cerebellar_peduncle_R
1915)	IDP_dMRI_TBSS_ICVF_Inferior_cerebellar_peduncle_L
1916)	IDP_dMRI_TBSS_ICVF_Superior_cerebellar_peduncle_R
1917)	IDP_dMRI_TBSS_ICVF_Superior_cerebellar_peduncle_L
1918)	IDP_dMRI_TBSS_ICVF_Cerebral_peduncle_R
1919)	IDP_dMRI_TBSS_ICVF_Cerebral_peduncle_L
1920)	IDP_dMRI_TBSS_ICVF_Anterior_limb_of_internal_capsule_R
1921)	IDP_dMRI_TBSS_ICVF_Anterior_limb_of_internal_capsule_L
1922)	IDP_dMRI_TBSS_ICVF_Posterior_limb_of_internal_capsule_R
1923)	IDP_dMRI_TBSS_ICVF_Posterior_limb_of_internal_capsule_L
1924)	IDP_dMRI_TBSS_ICVF_Retrolenticular_part_of_internal_capsule_R
1925)	IDP_dMRI_TBSS_ICVF_Retrolenticular_part_of_internal_capsule_L
1926)	IDP_dMRI_TBSS_ICVF_Anterior_corona_radiata_R
1927)	IDP_dMRI_TBSS_ICVF_Anterior_corona_radiata_L
1928)	IDP_dMRI_TBSS_ICVF_Superior_corona_radiata_R
1929)	IDP_dMRI_TBSS_ICVF_Superior_corona_radiata_L
1930)	IDP_dMRI_TBSS_ICVF_Posterior_corona_radiata_R

1931)	IDP_dMRI_TBSS_ICVF_Posterior_corona_radiata_L
1932)	IDP_dMRI_TBSS_ICVF_Posterior_thalamic_radiation_R
1933)	IDP_dMRI_TBSS_ICVF_Posterior_thalamic_radiation_L
1934)	IDP_dMRI_TBSS_ICVF_Sagittal_stratum_R
1935)	IDP_dMRI_TBSS_ICVF_Sagittal_stratum_L
1936)	IDP_dMRI_TBSS_ICVF_External_capsule_R
1937)	IDP_dMRI_TBSS_ICVF_External_capsule_L
1938)	IDP_dMRI_TBSS_ICVF_Cingulum_cingulate_gyrus_R
1939)	IDP_dMRI_TBSS_ICVF_Cingulum_cingulate_gyrus_L
1940)	IDP_dMRI_TBSS_ICVF_Cingulum_hippocampus_R
1941)	IDP_dMRI_TBSS_ICVF_Cingulum_hippocampus_L
1942)	IDP_dMRI_TBSS_ICVF_Fornix_cres+Stria_terminalis_R
1943)	IDP_dMRI_TBSS_ICVF_Fornix_cres+Stria_terminalis_L
1944)	IDP_dMRI_TBSS_ICVF_Superior_longitudinal_fasciculus_R
1945)	IDP_dMRI_TBSS_ICVF_Superior_longitudinal_fasciculus_L
1946)	IDP_dMRI_TBSS_ICVF_Superior_fronto-occipital_fasciculus_R
1947)	IDP_dMRI_TBSS_ICVF_Superior_fronto-occipital_fasciculus_L
1948)	IDP_dMRI_TBSS_ICVF_Uncinate_fasciculus_R
1949)	IDP_dMRI_TBSS_ICVF_Uncinate_fasciculus_L
1950)	IDP_dMRI_TBSS_ICVF_Tapetum_R
1951)	IDP_dMRI_TBSS_ICVF_Tapetum_L
1952)	IDP_dMRI_ProbtrackX_ICVF_ar_l
1953)	IDP_dMRI_ProbtrackX_ICVF_ar_r
1954)	IDP_dMRI_ProbtrackX_ICVF_atr_l
1955)	IDP_dMRI_ProbtrackX_ICVF_atr_r
1956)	IDP_dMRI_ProbtrackX_ICVF_cgc_l
1957)	IDP_dMRI_ProbtrackX_ICVF_cgc_r
1958)	IDP_dMRI_ProbtrackX_ICVF_cgh_l
1959)	IDP_dMRI_ProbtrackX_ICVF_cgh_r
1960)	IDP_dMRI_ProbtrackX_ICVF_cst_l
1961)	IDP_dMRI_ProbtrackX_ICVF_cst_r
1962)	IDP_dMRI_ProbtrackX_ICVF_fma
1963)	IDP_dMRI_ProbtrackX_ICVF_fmi
1964)	IDP_dMRI_ProbtrackX_ICVF_ifo_l
1965)	IDP_dMRI_ProbtrackX_ICVF_ifo_r
1966)	IDP_dMRI_ProbtrackX_ICVF_ilf_l
1967)	IDP_dMRI_ProbtrackX_ICVF_ilf_r
1968)	IDP_dMRI_ProbtrackX_ICVF_mcp
1969)	IDP_dMRI_ProbtrackX_ICVF_ml_l
1970)	IDP_dMRI_ProbtrackX_ICVF_ml_r
1971)	IDP_dMRI_ProbtrackX_ICVF_ptr_l
1972)	IDP_dMRI_ProbtrackX_ICVF_ptr_r

1973)	IDP_dMRI_ProbtrackX_ICVF_slf_l
1974)	IDP_dMRI_ProbtrackX_ICVF_slf_r
1975)	IDP_dMRI_ProbtrackX_ICVF_str_l
1976)	IDP_dMRI_ProbtrackX_ICVF_str_r
1977)	IDP_dMRI_ProbtrackX_ICVF_unc_l
1978)	IDP_dMRI_ProbtrackX_ICVF_unc_r
1979)	IDP_dMRI_TBSS_OD_Middle_cerebellar_peduncle
1980)	IDP_dMRI_TBSS_OD_Pontine_crossing_tract
1981)	IDP_dMRI_TBSS_OD_Genu_of_corpus_callosum
1982)	IDP_dMRI_TBSS_OD_Body_of_corpus_callosum
1983)	IDP_dMRI_TBSS_OD_Splenium_of_corpus_callosum
1984)	IDP_dMRI_TBSS_OD_Fornix
1985)	IDP_dMRI_TBSS_OD_Corticospinal_tract_R
1986)	IDP_dMRI_TBSS_OD_Corticospinal_tract_L
1987)	IDP_dMRI_TBSS_OD_Medial_lemniscus_R
1988)	IDP_dMRI_TBSS_OD_Medial_lemniscus_L
1989)	IDP_dMRI_TBSS_OD_Inferior_cerebellar_peduncle_R
1990)	IDP_dMRI_TBSS_OD_Inferior_cerebellar_peduncle_L
1991)	IDP_dMRI_TBSS_OD_Superior_cerebellar_peduncle_R
1992)	IDP_dMRI_TBSS_OD_Superior_cerebellar_peduncle_L
1993)	IDP_dMRI_TBSS_OD_Cerebral_peduncle_R
1994)	IDP_dMRI_TBSS_OD_Cerebral_peduncle_L
1995)	IDP_dMRI_TBSS_OD_Anterior_limb_of_internal_capsule_R
1996)	IDP_dMRI_TBSS_OD_Anterior_limb_of_internal_capsule_L
1997)	IDP_dMRI_TBSS_OD_Posterior_limb_of_internal_capsule_R
1998)	IDP_dMRI_TBSS_OD_Posterior_limb_of_internal_capsule_L
1999)	IDP_dMRI_TBSS_OD_Retrolenticular_part_of_internal_capsule_R
2000)	IDP_dMRI_TBSS_OD_Retrolenticular_part_of_internal_capsule_L
2001)	IDP_dMRI_TBSS_OD_Anterior_corona_radiata_R
2002)	IDP_dMRI_TBSS_OD_Anterior_corona_radiata_L
2003)	IDP_dMRI_TBSS_OD_Superior_corona_radiata_R
2004)	IDP_dMRI_TBSS_OD_Superior_corona_radiata_L
2005)	IDP_dMRI_TBSS_OD_Posterior_corona_radiata_R
2006)	IDP_dMRI_TBSS_OD_Posterior_corona_radiata_L
2007)	IDP_dMRI_TBSS_OD_Posterior_thalamic_radiation_R
2008)	IDP_dMRI_TBSS_OD_Posterior_thalamic_radiation_L
2009)	IDP_dMRI_TBSS_OD_Sagittal_stratum_R
2010)	IDP_dMRI_TBSS_OD_Sagittal_stratum_L
2011)	IDP_dMRI_TBSS_OD_External_capsule_R
2012)	IDP_dMRI_TBSS_OD_External_capsule_L
2013)	IDP_dMRI_TBSS_OD_Cingulum_cingulate_gyrus_R
2014)	IDP_dMRI_TBSS_OD_Cingulum_cingulate_gyrus_L

2015)	IDP_dMRI_TBSS_OD_Cingulum_hippocampus_R
2016)	IDP_dMRI_TBSS_OD_Cingulum_hippocampus_L
2017)	IDP_dMRI_TBSS_OD_Fornix_cres+Stria_terminalis_R
2018)	IDP_dMRI_TBSS_OD_Fornix_cres+Stria_terminalis_L
2019)	IDP_dMRI_TBSS_OD_Superior_longitudinal_fasciculus_R
2020)	IDP_dMRI_TBSS_OD_Superior_longitudinal_fasciculus_L
2021)	IDP_dMRI_TBSS_OD_Superior_fronto-occipital_fasciculus_R
2022)	IDP_dMRI_TBSS_OD_Superior_fronto-occipital_fasciculus_L
2023)	IDP_dMRI_TBSS_OD_Uncinate_fasciculus_R
2024)	IDP_dMRI_TBSS_OD_Uncinate_fasciculus_L
2025)	IDP_dMRI_TBSS_OD_Tapetum_R
2026)	IDP_dMRI_TBSS_OD_Tapetum_L
2027)	IDP_dMRI_ProbtrackX_OD_ar_l
2028)	IDP_dMRI_ProbtrackX_OD_ar_r
2029)	IDP_dMRI_ProbtrackX_OD_atr_l
2030)	IDP_dMRI_ProbtrackX_OD_atr_r
2031)	IDP_dMRI_ProbtrackX_OD_cgc_l
2032)	IDP_dMRI_ProbtrackX_OD_cgc_r
2033)	IDP_dMRI_ProbtrackX_OD_cgh_l
2034)	IDP_dMRI_ProbtrackX_OD_cgh_r
2035)	IDP_dMRI_ProbtrackX_OD_cst_l
2036)	IDP_dMRI_ProbtrackX_OD_cst_r
2037)	IDP_dMRI_ProbtrackX_OD_fma
2038)	IDP_dMRI_ProbtrackX_OD_fmi
2039)	IDP_dMRI_ProbtrackX_OD_ifo_l
2040)	IDP_dMRI_ProbtrackX_OD_ifo_r
2041)	IDP_dMRI_ProbtrackX_OD_ilf_l
2042)	IDP_dMRI_ProbtrackX_OD_ilf_r
2043)	IDP_dMRI_ProbtrackX_OD_mcp
2044)	IDP_dMRI_ProbtrackX_OD_ml_l
2045)	IDP_dMRI_ProbtrackX_OD_ml_r
2046)	IDP_dMRI_ProbtrackX_OD_ptr_l
2047)	IDP_dMRI_ProbtrackX_OD_ptr_r
2048)	IDP_dMRI_ProbtrackX_OD_slf_l
2049)	IDP_dMRI_ProbtrackX_OD_slf_r
2050)	IDP_dMRI_ProbtrackX_OD_str_l
2051)	IDP_dMRI_ProbtrackX_OD_str_r
2052)	IDP_dMRI_ProbtrackX_OD_unc_l
2053)	IDP_dMRI_ProbtrackX_OD_unc_r
2054)	IDP_dMRI_TBSS_ISOVF_Middle_cerebellar_peduncle
2055)	IDP_dMRI_TBSS_ISOVF_Pontine_crossing_tract
2056)	IDP_dMRI_TBSS_ISOVF_Genu_of_corpus_callosum

2057)	IDP_dMRI_TBSS_ISOVF_Body_of_corpus_callosum
2058)	IDP_dMRI_TBSS_ISOVF_Splenium_of_corpus_callosum
2059)	IDP_dMRI_TBSS_ISOVF_Fornix
2060)	IDP_dMRI_TBSS_ISOVF_Corticospinal_tract_R
2061)	IDP_dMRI_TBSS_ISOVF_Corticospinal_tract_L
2062)	IDP_dMRI_TBSS_ISOVF_Medial_lemniscus_R
2063)	IDP_dMRI_TBSS_ISOVF_Medial_lemniscus_L
2064)	IDP_dMRI_TBSS_ISOVF_Inferior_cerebellar_peduncle_R
2065)	IDP_dMRI_TBSS_ISOVF_Inferior_cerebellar_peduncle_L
2066)	IDP_dMRI_TBSS_ISOVF_Superior_cerebellar_peduncle_R
2067)	IDP_dMRI_TBSS_ISOVF_Superior_cerebellar_peduncle_L
2068)	IDP_dMRI_TBSS_ISOVF_Cerebral_peduncle_R
2069)	IDP_dMRI_TBSS_ISOVF_Cerebral_peduncle_L
2070)	IDP_dMRI_TBSS_ISOVF_Anterior_limb_of_internal_capsule_R
2071)	IDP_dMRI_TBSS_ISOVF_Anterior_limb_of_internal_capsule_L
2072)	IDP_dMRI_TBSS_ISOVF_Posterior_limb_of_internal_capsule_R
2073)	IDP_dMRI_TBSS_ISOVF_Posterior_limb_of_internal_capsule_L
2074)	IDP_dMRI_TBSS_ISOVF_Retrolenticular_part_of_internal_capsule_R
2075)	IDP_dMRI_TBSS_ISOVF_Retrolenticular_part_of_internal_capsule_L
2076)	IDP_dMRI_TBSS_ISOVF_Anterior_corona_radiata_R
2077)	IDP_dMRI_TBSS_ISOVF_Anterior_corona_radiata_L
2078)	IDP_dMRI_TBSS_ISOVF_Superior_corona_radiata_R
2079)	IDP_dMRI_TBSS_ISOVF_Superior_corona_radiata_L
2080)	IDP_dMRI_TBSS_ISOVF_Posterior_corona_radiata_R
2081)	IDP_dMRI_TBSS_ISOVF_Posterior_corona_radiata_L
2082)	IDP_dMRI_TBSS_ISOVF_Posterior_thalamic_radiation_R
2083)	IDP_dMRI_TBSS_ISOVF_Posterior_thalamic_radiation_L
2084)	IDP_dMRI_TBSS_ISOVF_Sagittal_stratum_R
2085)	IDP_dMRI_TBSS_ISOVF_Sagittal_stratum_L
2086)	IDP_dMRI_TBSS_ISOVF_External_capsule_R
2087)	IDP_dMRI_TBSS_ISOVF_External_capsule_L
2088)	IDP_dMRI_TBSS_ISOVF_Cingulum_cingulate_gyrus_R
2089)	IDP_dMRI_TBSS_ISOVF_Cingulum_cingulate_gyrus_L
2090)	IDP_dMRI_TBSS_ISOVF_Cingulum_hippocampus_R
2091)	IDP_dMRI_TBSS_ISOVF_Cingulum_hippocampus_L
2092)	IDP_dMRI_TBSS_ISOVF_Fornix_cres+Stria_terminalis_R
2093)	IDP_dMRI_TBSS_ISOVF_Fornix_cres+Stria_terminalis_L
2094)	IDP_dMRI_TBSS_ISOVF_Superior_longitudinal_fasciculus_R
2095)	IDP_dMRI_TBSS_ISOVF_Superior_longitudinal_fasciculus_L
2096)	IDP_dMRI_TBSS_ISOVF_Superior_fronto-occipital_fasciculus_R
2097)	IDP_dMRI_TBSS_ISOVF_Superior_fronto-occipital_fasciculus_L
2098)	IDP_dMRI_TBSS_ISOVF_Uncinate_fasciculus_R

2099)	IDP_dMRI_TBSS_ISOVF_Uncinate_fasciculus_L
2100)	IDP_dMRI_TBSS_ISOVF_Tapetum_R
2101)	IDP_dMRI_TBSS_ISOVF_Tapetum_L
2102)	IDP_dMRI_ProbtrackX_ISOVF_ar_l
2103)	IDP_dMRI_ProbtrackX_ISOVF_ar_r
2104)	IDP_dMRI_ProbtrackX_ISOVF_atr_l
2105)	IDP_dMRI_ProbtrackX_ISOVF_atr_r
2106)	IDP_dMRI_ProbtrackX_ISOVF_cgc_l
2107)	IDP_dMRI_ProbtrackX_ISOVF_cgc_r
2108)	IDP_dMRI_ProbtrackX_ISOVF_cgh_l
2109)	IDP_dMRI_ProbtrackX_ISOVF_cgh_r
2110)	IDP_dMRI_ProbtrackX_ISOVF_cst_l
2111)	IDP_dMRI_ProbtrackX_ISOVF_cst_r
2112)	IDP_dMRI_ProbtrackX_ISOVF_fma
2113)	IDP_dMRI_ProbtrackX_ISOVF_fmi
2114)	IDP_dMRI_ProbtrackX_ISOVF_ifo_l
2115)	IDP_dMRI_ProbtrackX_ISOVF_ifo_r
2116)	IDP_dMRI_ProbtrackX_ISOVF_ilf_l
2117)	IDP_dMRI_ProbtrackX_ISOVF_ilf_r
2118)	IDP_dMRI_ProbtrackX_ISOVF_mcp
2119)	IDP_dMRI_ProbtrackX_ISOVF_ml_l
2120)	IDP_dMRI_ProbtrackX_ISOVF_ml_r
2121)	IDP_dMRI_ProbtrackX_ISOVF_ptr_l
2122)	IDP_dMRI_ProbtrackX_ISOVF_ptr_r
2123)	IDP_dMRI_ProbtrackX_ISOVF_slf_l
2124)	IDP_dMRI_ProbtrackX_ISOVF_slf_r
2125)	IDP_dMRI_ProbtrackX_ISOVF_str_l
2126)	IDP_dMRI_ProbtrackX_ISOVF_str_r
2127)	IDP_dMRI_ProbtrackX_ISOVF_unc_l
2128)	IDP_dMRI_ProbtrackX_ISOVF_unc_r
2129)	IDP_tfMRI_median_BOLD_shapes
2130)	IDP_tfMRI_90th-percentile_BOLD_shapes
2131)	IDP_tfMRI_median_zstat_shapes
2132)	IDP_tfMRI_90th-percentile_zstat_shapes
2133)	IDP_tfMRI_median_BOLD_faces
2134)	IDP_tfMRI_90th-percentile_BOLD_faces
2135)	IDP_tfMRI_median_zstat_faces
2136)	IDP_tfMRI_90th-percentile_zstat_faces
2137)	IDP_tfMRI_median_BOLD_faces-shapes
2138)	IDP_tfMRI_90th-percentile_BOLD_faces-shapes
2139)	IDP_tfMRI_median_zstat_faces-shapes
2140)	IDP_tfMRI_90th-percentile_zstat_faces-shapes

2141)	IDP_tfMRI_median_BOLD_faces-shapes_amygdala
2142)	IDP_tfMRI_90th-percentile_BOLD_faces-shapes_amygdala
2143)	IDP_tfMRI_median_zstat_faces-shapes_amygdala
2144)	IDP_tfMRI_90th-percentile_zstat_faces-shapes_amygdala
2145)	rfMRI amplitudes (ICA25 node 1)
2146)	rfMRI amplitudes (ICA25 node 2)
2147)	rfMRI amplitudes (ICA25 node 3)
2148)	rfMRI amplitudes (ICA25 node 4)
2149)	rfMRI amplitudes (ICA25 node 5)
2150)	rfMRI amplitudes (ICA25 node 6)
2151)	rfMRI amplitudes (ICA25 node 7)
2152)	rfMRI amplitudes (ICA25 node 8)
2153)	rfMRI amplitudes (ICA25 node 9)
2154)	rfMRI amplitudes (ICA25 node 10)
2155)	rfMRI amplitudes (ICA25 node 11)
2156)	rfMRI amplitudes (ICA25 node 12)
2157)	rfMRI amplitudes (ICA25 node 13)
2158)	rfMRI amplitudes (ICA25 node 14)
2159)	rfMRI amplitudes (ICA25 node 15)
2160)	rfMRI amplitudes (ICA25 node 16)
2161)	rfMRI amplitudes (ICA25 node 17)
2162)	rfMRI amplitudes (ICA25 node 18)
2163)	rfMRI amplitudes (ICA25 node 19)
2164)	rfMRI amplitudes (ICA25 node 20)
2165)	rfMRI amplitudes (ICA25 node 21)
2166)	rfMRI amplitudes (ICA100 node 1)
2167)	rfMRI amplitudes (ICA100 node 2)
2168)	rfMRI amplitudes (ICA100 node 3)
2169)	rfMRI amplitudes (ICA100 node 4)
2170)	rfMRI amplitudes (ICA100 node 5)
2171)	rfMRI amplitudes (ICA100 node 6)
2172)	rfMRI amplitudes (ICA100 node 7)
2173)	rfMRI amplitudes (ICA100 node 8)
2174)	rfMRI amplitudes (ICA100 node 9)
2175)	rfMRI amplitudes (ICA100 node 10)
2176)	rfMRI amplitudes (ICA100 node 11)
2177)	rfMRI amplitudes (ICA100 node 12)
2178)	rfMRI amplitudes (ICA100 node 13)
2179)	rfMRI amplitudes (ICA100 node 14)
2180)	rfMRI amplitudes (ICA100 node 15)
2181)	rfMRI amplitudes (ICA100 node 16)
2182)	rfMRI amplitudes (ICA100 node 17)

2183)	rfMRI amplitudes (ICA100 node 18)
2184)	rfMRI amplitudes (ICA100 node 19)
2185)	rfMRI amplitudes (ICA100 node 20)
2186)	rfMRI amplitudes (ICA100 node 21)
2187)	rfMRI amplitudes (ICA100 node 22)
2188)	rfMRI amplitudes (ICA100 node 23)
2189)	rfMRI amplitudes (ICA100 node 24)
2190)	rfMRI amplitudes (ICA100 node 25)
2191)	rfMRI amplitudes (ICA100 node 26)
2192)	rfMRI amplitudes (ICA100 node 27)
2193)	rfMRI amplitudes (ICA100 node 28)
2194)	rfMRI amplitudes (ICA100 node 29)
2195)	rfMRI amplitudes (ICA100 node 30)
2196)	rfMRI amplitudes (ICA100 node 31)
2197)	rfMRI amplitudes (ICA100 node 32)
2198)	rfMRI amplitudes (ICA100 node 33)
2199)	rfMRI amplitudes (ICA100 node 34)
2200)	rfMRI amplitudes (ICA100 node 35)
2201)	rfMRI amplitudes (ICA100 node 36)
2202)	rfMRI amplitudes (ICA100 node 37)
2203)	rfMRI amplitudes (ICA100 node 38)
2204)	rfMRI amplitudes (ICA100 node 39)
2205)	rfMRI amplitudes (ICA100 node 40)
2206)	rfMRI amplitudes (ICA100 node 41)
2207)	rfMRI amplitudes (ICA100 node 42)
2208)	rfMRI amplitudes (ICA100 node 43)
2209)	rfMRI amplitudes (ICA100 node 44)
2210)	rfMRI amplitudes (ICA100 node 45)
2211)	rfMRI amplitudes (ICA100 node 46)
2212)	rfMRI amplitudes (ICA100 node 47)
2213)	rfMRI amplitudes (ICA100 node 48)
2214)	rfMRI amplitudes (ICA100 node 49)
2215)	rfMRI amplitudes (ICA100 node 50)
2216)	rfMRI amplitudes (ICA100 node 51)
2217)	rfMRI amplitudes (ICA100 node 52)
2218)	rfMRI amplitudes (ICA100 node 53)
2219)	rfMRI amplitudes (ICA100 node 54)
2220)	rfMRI amplitudes (ICA100 node 55)
2221)	rfMRI connectivity (ICA25 edge 1)
2222)	rfMRI connectivity (ICA25 edge 2)
2223)	rfMRI connectivity (ICA25 edge 3)
2224)	rfMRI connectivity (ICA25 edge 4)

2225)	rfMRI connectivity (ICA25 edge 5)
2226)	rfMRI connectivity (ICA25 edge 6)
2227)	rfMRI connectivity (ICA25 edge 7)
2228)	rfMRI connectivity (ICA25 edge 8)
2229)	rfMRI connectivity (ICA25 edge 9)
2230)	rfMRI connectivity (ICA25 edge 10)
2231)	rfMRI connectivity (ICA25 edge 11)
2232)	rfMRI connectivity (ICA25 edge 12)
2233)	rfMRI connectivity (ICA25 edge 13)
2234)	rfMRI connectivity (ICA25 edge 14)
2235)	rfMRI connectivity (ICA25 edge 15)
2236)	rfMRI connectivity (ICA25 edge 16)
2237)	rfMRI connectivity (ICA25 edge 17)
2238)	rfMRI connectivity (ICA25 edge 18)
2239)	rfMRI connectivity (ICA25 edge 19)
2240)	rfMRI connectivity (ICA25 edge 20)
2241)	rfMRI connectivity (ICA25 edge 21)
2242)	rfMRI connectivity (ICA25 edge 22)
2243)	rfMRI connectivity (ICA25 edge 23)
2244)	rfMRI connectivity (ICA25 edge 24)
2245)	rfMRI connectivity (ICA25 edge 25)
2246)	rfMRI connectivity (ICA25 edge 26)
2247)	rfMRI connectivity (ICA25 edge 27)
2248)	rfMRI connectivity (ICA25 edge 28)
2249)	rfMRI connectivity (ICA25 edge 29)
2250)	rfMRI connectivity (ICA25 edge 30)
2251)	rfMRI connectivity (ICA25 edge 31)
2252)	rfMRI connectivity (ICA25 edge 32)
2253)	rfMRI connectivity (ICA25 edge 33)
2254)	rfMRI connectivity (ICA25 edge 34)
2255)	rfMRI connectivity (ICA25 edge 35)
2256)	rfMRI connectivity (ICA25 edge 36)
2257)	rfMRI connectivity (ICA25 edge 37)
2258)	rfMRI connectivity (ICA25 edge 38)
2259)	rfMRI connectivity (ICA25 edge 39)
2260)	rfMRI connectivity (ICA25 edge 40)
2261)	rfMRI connectivity (ICA25 edge 41)
2262)	rfMRI connectivity (ICA25 edge 42)
2263)	rfMRI connectivity (ICA25 edge 43)
2264)	rfMRI connectivity (ICA25 edge 44)
2265)	rfMRI connectivity (ICA25 edge 45)
2266)	rfMRI connectivity (ICA25 edge 46)

2267)	rfMRI connectivity (ICA25 edge 47)
2268)	rfMRI connectivity (ICA25 edge 48)
2269)	rfMRI connectivity (ICA25 edge 49)
2270)	rfMRI connectivity (ICA25 edge 50)
2271)	rfMRI connectivity (ICA25 edge 51)
2272)	rfMRI connectivity (ICA25 edge 52)
2273)	rfMRI connectivity (ICA25 edge 53)
2274)	rfMRI connectivity (ICA25 edge 54)
2275)	rfMRI connectivity (ICA25 edge 55)
2276)	rfMRI connectivity (ICA25 edge 56)
2277)	rfMRI connectivity (ICA25 edge 57)
2278)	rfMRI connectivity (ICA25 edge 58)
2279)	rfMRI connectivity (ICA25 edge 59)
2280)	rfMRI connectivity (ICA25 edge 60)
2281)	rfMRI connectivity (ICA25 edge 61)
2282)	rfMRI connectivity (ICA25 edge 62)
2283)	rfMRI connectivity (ICA25 edge 63)
2284)	rfMRI connectivity (ICA25 edge 64)
2285)	rfMRI connectivity (ICA25 edge 65)
2286)	rfMRI connectivity (ICA25 edge 66)
2287)	rfMRI connectivity (ICA25 edge 67)
2288)	rfMRI connectivity (ICA25 edge 68)
2289)	rfMRI connectivity (ICA25 edge 69)
2290)	rfMRI connectivity (ICA25 edge 70)
2291)	rfMRI connectivity (ICA25 edge 71)
2292)	rfMRI connectivity (ICA25 edge 72)
2293)	rfMRI connectivity (ICA25 edge 73)
2294)	rfMRI connectivity (ICA25 edge 74)
2295)	rfMRI connectivity (ICA25 edge 75)
2296)	rfMRI connectivity (ICA25 edge 76)
2297)	rfMRI connectivity (ICA25 edge 77)
2298)	rfMRI connectivity (ICA25 edge 78)
2299)	rfMRI connectivity (ICA25 edge 79)
2300)	rfMRI connectivity (ICA25 edge 80)
2301)	rfMRI connectivity (ICA25 edge 81)
2302)	rfMRI connectivity (ICA25 edge 82)
2303)	rfMRI connectivity (ICA25 edge 83)
2304)	rfMRI connectivity (ICA25 edge 84)
2305)	rfMRI connectivity (ICA25 edge 85)
2306)	rfMRI connectivity (ICA25 edge 86)
2307)	rfMRI connectivity (ICA25 edge 87)
2308)	rfMRI connectivity (ICA25 edge 88)

2309)	rfMRI connectivity (ICA25 edge 89)
2310)	rfMRI connectivity (ICA25 edge 90)
2311)	rfMRI connectivity (ICA25 edge 91)
2312)	rfMRI connectivity (ICA25 edge 92)
2313)	rfMRI connectivity (ICA25 edge 93)
2314)	rfMRI connectivity (ICA25 edge 94)
2315)	rfMRI connectivity (ICA25 edge 95)
2316)	rfMRI connectivity (ICA25 edge 96)
2317)	rfMRI connectivity (ICA25 edge 97)
2318)	rfMRI connectivity (ICA25 edge 98)
2319)	rfMRI connectivity (ICA25 edge 99)
2320)	rfMRI connectivity (ICA25 edge 100)
2321)	rfMRI connectivity (ICA25 edge 101)
2322)	rfMRI connectivity (ICA25 edge 102)
2323)	rfMRI connectivity (ICA25 edge 103)
2324)	rfMRI connectivity (ICA25 edge 104)
2325)	rfMRI connectivity (ICA25 edge 105)
2326)	rfMRI connectivity (ICA25 edge 106)
2327)	rfMRI connectivity (ICA25 edge 107)
2328)	rfMRI connectivity (ICA25 edge 108)
2329)	rfMRI connectivity (ICA25 edge 109)
2330)	rfMRI connectivity (ICA25 edge 110)
2331)	rfMRI connectivity (ICA25 edge 111)
2332)	rfMRI connectivity (ICA25 edge 112)
2333)	rfMRI connectivity (ICA25 edge 113)
2334)	rfMRI connectivity (ICA25 edge 114)
2335)	rfMRI connectivity (ICA25 edge 115)
2336)	rfMRI connectivity (ICA25 edge 116)
2337)	rfMRI connectivity (ICA25 edge 117)
2338)	rfMRI connectivity (ICA25 edge 118)
2339)	rfMRI connectivity (ICA25 edge 119)
2340)	rfMRI connectivity (ICA25 edge 120)
2341)	rfMRI connectivity (ICA25 edge 121)
2342)	rfMRI connectivity (ICA25 edge 122)
2343)	rfMRI connectivity (ICA25 edge 123)
2344)	rfMRI connectivity (ICA25 edge 124)
2345)	rfMRI connectivity (ICA25 edge 125)
2346)	rfMRI connectivity (ICA25 edge 126)
2347)	rfMRI connectivity (ICA25 edge 127)
2348)	rfMRI connectivity (ICA25 edge 128)
2349)	rfMRI connectivity (ICA25 edge 129)
2350)	rfMRI connectivity (ICA25 edge 130)

2351)	rfMRI connectivity (ICA25 edge 131)
2352)	rfMRI connectivity (ICA25 edge 132)
2353)	rfMRI connectivity (ICA25 edge 133)
2354)	rfMRI connectivity (ICA25 edge 134)
2355)	rfMRI connectivity (ICA25 edge 135)
2356)	rfMRI connectivity (ICA25 edge 136)
2357)	rfMRI connectivity (ICA25 edge 137)
2358)	rfMRI connectivity (ICA25 edge 138)
2359)	rfMRI connectivity (ICA25 edge 139)
2360)	rfMRI connectivity (ICA25 edge 140)
2361)	rfMRI connectivity (ICA25 edge 141)
2362)	rfMRI connectivity (ICA25 edge 142)
2363)	rfMRI connectivity (ICA25 edge 143)
2364)	rfMRI connectivity (ICA25 edge 144)
2365)	rfMRI connectivity (ICA25 edge 145)
2366)	rfMRI connectivity (ICA25 edge 146)
2367)	rfMRI connectivity (ICA25 edge 147)
2368)	rfMRI connectivity (ICA25 edge 148)
2369)	rfMRI connectivity (ICA25 edge 149)
2370)	rfMRI connectivity (ICA25 edge 150)
2371)	rfMRI connectivity (ICA25 edge 151)
2372)	rfMRI connectivity (ICA25 edge 152)
2373)	rfMRI connectivity (ICA25 edge 153)
2374)	rfMRI connectivity (ICA25 edge 154)
2375)	rfMRI connectivity (ICA25 edge 155)
2376)	rfMRI connectivity (ICA25 edge 156)
2377)	rfMRI connectivity (ICA25 edge 157)
2378)	rfMRI connectivity (ICA25 edge 158)
2379)	rfMRI connectivity (ICA25 edge 159)
2380)	rfMRI connectivity (ICA25 edge 160)
2381)	rfMRI connectivity (ICA25 edge 161)
2382)	rfMRI connectivity (ICA25 edge 162)
2383)	rfMRI connectivity (ICA25 edge 163)
2384)	rfMRI connectivity (ICA25 edge 164)
2385)	rfMRI connectivity (ICA25 edge 165)
2386)	rfMRI connectivity (ICA25 edge 166)
2387)	rfMRI connectivity (ICA25 edge 167)
2388)	rfMRI connectivity (ICA25 edge 168)
2389)	rfMRI connectivity (ICA25 edge 169)
2390)	rfMRI connectivity (ICA25 edge 170)
2391)	rfMRI connectivity (ICA25 edge 171)
2392)	rfMRI connectivity (ICA25 edge 172)

2393)	rfMRI connectivity (ICA25 edge 173)
2394)	rfMRI connectivity (ICA25 edge 174)
2395)	rfMRI connectivity (ICA25 edge 175)
2396)	rfMRI connectivity (ICA25 edge 176)
2397)	rfMRI connectivity (ICA25 edge 177)
2398)	rfMRI connectivity (ICA25 edge 178)
2399)	rfMRI connectivity (ICA25 edge 179)
2400)	rfMRI connectivity (ICA25 edge 180)
2401)	rfMRI connectivity (ICA25 edge 181)
2402)	rfMRI connectivity (ICA25 edge 182)
2403)	rfMRI connectivity (ICA25 edge 183)
2404)	rfMRI connectivity (ICA25 edge 184)
2405)	rfMRI connectivity (ICA25 edge 185)
2406)	rfMRI connectivity (ICA25 edge 186)
2407)	rfMRI connectivity (ICA25 edge 187)
2408)	rfMRI connectivity (ICA25 edge 188)
2409)	rfMRI connectivity (ICA25 edge 189)
2410)	rfMRI connectivity (ICA25 edge 190)
2411)	rfMRI connectivity (ICA25 edge 191)
2412)	rfMRI connectivity (ICA25 edge 192)
2413)	rfMRI connectivity (ICA25 edge 193)
2414)	rfMRI connectivity (ICA25 edge 194)
2415)	rfMRI connectivity (ICA25 edge 195)
2416)	rfMRI connectivity (ICA25 edge 196)
2417)	rfMRI connectivity (ICA25 edge 197)
2418)	rfMRI connectivity (ICA25 edge 198)
2419)	rfMRI connectivity (ICA25 edge 199)
2420)	rfMRI connectivity (ICA25 edge 200)
2421)	rfMRI connectivity (ICA25 edge 201)
2422)	rfMRI connectivity (ICA25 edge 202)
2423)	rfMRI connectivity (ICA25 edge 203)
2424)	rfMRI connectivity (ICA25 edge 204)
2425)	rfMRI connectivity (ICA25 edge 205)
2426)	rfMRI connectivity (ICA25 edge 206)
2427)	rfMRI connectivity (ICA25 edge 207)
2428)	rfMRI connectivity (ICA25 edge 208)
2429)	rfMRI connectivity (ICA25 edge 209)
2430)	rfMRI connectivity (ICA25 edge 210)
2431)	rfMRI connectivity (ICA100 edge 1)
2432)	rfMRI connectivity (ICA100 edge 2)
2433)	rfMRI connectivity (ICA100 edge 3)
2434)	rfMRI connectivity (ICA100 edge 4)

2435)	rfMRI connectivity (ICA100 edge 5)
2436)	rfMRI connectivity (ICA100 edge 6)
2437)	rfMRI connectivity (ICA100 edge 7)
2438)	rfMRI connectivity (ICA100 edge 8)
2439)	rfMRI connectivity (ICA100 edge 9)
2440)	rfMRI connectivity (ICA100 edge 10)
2441)	rfMRI connectivity (ICA100 edge 11)
2442)	rfMRI connectivity (ICA100 edge 12)
2443)	rfMRI connectivity (ICA100 edge 13)
2444)	rfMRI connectivity (ICA100 edge 14)
2445)	rfMRI connectivity (ICA100 edge 15)
2446)	rfMRI connectivity (ICA100 edge 16)
2447)	rfMRI connectivity (ICA100 edge 17)
2448)	rfMRI connectivity (ICA100 edge 18)
2449)	rfMRI connectivity (ICA100 edge 19)
2450)	rfMRI connectivity (ICA100 edge 20)
2451)	rfMRI connectivity (ICA100 edge 21)
2452)	rfMRI connectivity (ICA100 edge 22)
2453)	rfMRI connectivity (ICA100 edge 23)
2454)	rfMRI connectivity (ICA100 edge 24)
2455)	rfMRI connectivity (ICA100 edge 25)
2456)	rfMRI connectivity (ICA100 edge 26)
2457)	rfMRI connectivity (ICA100 edge 27)
2458)	rfMRI connectivity (ICA100 edge 28)
2459)	rfMRI connectivity (ICA100 edge 29)
2460)	rfMRI connectivity (ICA100 edge 30)
2461)	rfMRI connectivity (ICA100 edge 31)
2462)	rfMRI connectivity (ICA100 edge 32)
2463)	rfMRI connectivity (ICA100 edge 33)
2464)	rfMRI connectivity (ICA100 edge 34)
2465)	rfMRI connectivity (ICA100 edge 35)
2466)	rfMRI connectivity (ICA100 edge 36)
2467)	rfMRI connectivity (ICA100 edge 37)
2468)	rfMRI connectivity (ICA100 edge 38)
2469)	rfMRI connectivity (ICA100 edge 39)
2470)	rfMRI connectivity (ICA100 edge 40)
2471)	rfMRI connectivity (ICA100 edge 41)
2472)	rfMRI connectivity (ICA100 edge 42)
2473)	rfMRI connectivity (ICA100 edge 43)
2474)	rfMRI connectivity (ICA100 edge 44)
2475)	rfMRI connectivity (ICA100 edge 45)
2476)	rfMRI connectivity (ICA100 edge 46)

2477)	rfMRI connectivity (ICA100 edge 47)
2478)	rfMRI connectivity (ICA100 edge 48)
2479)	rfMRI connectivity (ICA100 edge 49)
2480)	rfMRI connectivity (ICA100 edge 50)
2481)	rfMRI connectivity (ICA100 edge 51)
2482)	rfMRI connectivity (ICA100 edge 52)
2483)	rfMRI connectivity (ICA100 edge 53)
2484)	rfMRI connectivity (ICA100 edge 54)
2485)	rfMRI connectivity (ICA100 edge 55)
2486)	rfMRI connectivity (ICA100 edge 56)
2487)	rfMRI connectivity (ICA100 edge 57)
2488)	rfMRI connectivity (ICA100 edge 58)
2489)	rfMRI connectivity (ICA100 edge 59)
2490)	rfMRI connectivity (ICA100 edge 60)
2491)	rfMRI connectivity (ICA100 edge 61)
2492)	rfMRI connectivity (ICA100 edge 62)
2493)	rfMRI connectivity (ICA100 edge 63)
2494)	rfMRI connectivity (ICA100 edge 64)
2495)	rfMRI connectivity (ICA100 edge 65)
2496)	rfMRI connectivity (ICA100 edge 66)
2497)	rfMRI connectivity (ICA100 edge 67)
2498)	rfMRI connectivity (ICA100 edge 68)
2499)	rfMRI connectivity (ICA100 edge 69)
2500)	rfMRI connectivity (ICA100 edge 70)
2501)	rfMRI connectivity (ICA100 edge 71)
2502)	rfMRI connectivity (ICA100 edge 72)
2503)	rfMRI connectivity (ICA100 edge 73)
2504)	rfMRI connectivity (ICA100 edge 74)
2505)	rfMRI connectivity (ICA100 edge 75)
2506)	rfMRI connectivity (ICA100 edge 76)
2507)	rfMRI connectivity (ICA100 edge 77)
2508)	rfMRI connectivity (ICA100 edge 78)
2509)	rfMRI connectivity (ICA100 edge 79)
2510)	rfMRI connectivity (ICA100 edge 80)
2511)	rfMRI connectivity (ICA100 edge 81)
2512)	rfMRI connectivity (ICA100 edge 82)
2513)	rfMRI connectivity (ICA100 edge 83)
2514)	rfMRI connectivity (ICA100 edge 84)
2515)	rfMRI connectivity (ICA100 edge 85)
2516)	rfMRI connectivity (ICA100 edge 86)
2517)	rfMRI connectivity (ICA100 edge 87)
2518)	rfMRI connectivity (ICA100 edge 88)

2519)	rfMRI connectivity (ICA100 edge 89)
2520)	rfMRI connectivity (ICA100 edge 90)
2521)	rfMRI connectivity (ICA100 edge 91)
2522)	rfMRI connectivity (ICA100 edge 92)
2523)	rfMRI connectivity (ICA100 edge 93)
2524)	rfMRI connectivity (ICA100 edge 94)
2525)	rfMRI connectivity (ICA100 edge 95)
2526)	rfMRI connectivity (ICA100 edge 96)
2527)	rfMRI connectivity (ICA100 edge 97)
2528)	rfMRI connectivity (ICA100 edge 98)
2529)	rfMRI connectivity (ICA100 edge 99)
2530)	rfMRI connectivity (ICA100 edge 100)
2531)	rfMRI connectivity (ICA100 edge 101)
2532)	rfMRI connectivity (ICA100 edge 102)
2533)	rfMRI connectivity (ICA100 edge 103)
2534)	rfMRI connectivity (ICA100 edge 104)
2535)	rfMRI connectivity (ICA100 edge 105)
2536)	rfMRI connectivity (ICA100 edge 106)
2537)	rfMRI connectivity (ICA100 edge 107)
2538)	rfMRI connectivity (ICA100 edge 108)
2539)	rfMRI connectivity (ICA100 edge 109)
2540)	rfMRI connectivity (ICA100 edge 110)
2541)	rfMRI connectivity (ICA100 edge 111)
2542)	rfMRI connectivity (ICA100 edge 112)
2543)	rfMRI connectivity (ICA100 edge 113)
2544)	rfMRI connectivity (ICA100 edge 114)
2545)	rfMRI connectivity (ICA100 edge 115)
2546)	rfMRI connectivity (ICA100 edge 116)
2547)	rfMRI connectivity (ICA100 edge 117)
2548)	rfMRI connectivity (ICA100 edge 118)
2549)	rfMRI connectivity (ICA100 edge 119)
2550)	rfMRI connectivity (ICA100 edge 120)
2551)	rfMRI connectivity (ICA100 edge 121)
2552)	rfMRI connectivity (ICA100 edge 122)
2553)	rfMRI connectivity (ICA100 edge 123)
2554)	rfMRI connectivity (ICA100 edge 124)
2555)	rfMRI connectivity (ICA100 edge 125)
2556)	rfMRI connectivity (ICA100 edge 126)
2557)	rfMRI connectivity (ICA100 edge 127)
2558)	rfMRI connectivity (ICA100 edge 128)
2559)	rfMRI connectivity (ICA100 edge 129)
2560)	rfMRI connectivity (ICA100 edge 130)

2561)	rfMRI connectivity (ICA100 edge 131)
2562)	rfMRI connectivity (ICA100 edge 132)
2563)	rfMRI connectivity (ICA100 edge 133)
2564)	rfMRI connectivity (ICA100 edge 134)
2565)	rfMRI connectivity (ICA100 edge 135)
2566)	rfMRI connectivity (ICA100 edge 136)
2567)	rfMRI connectivity (ICA100 edge 137)
2568)	rfMRI connectivity (ICA100 edge 138)
2569)	rfMRI connectivity (ICA100 edge 139)
2570)	rfMRI connectivity (ICA100 edge 140)
2571)	rfMRI connectivity (ICA100 edge 141)
2572)	rfMRI connectivity (ICA100 edge 142)
2573)	rfMRI connectivity (ICA100 edge 143)
2574)	rfMRI connectivity (ICA100 edge 144)
2575)	rfMRI connectivity (ICA100 edge 145)
2576)	rfMRI connectivity (ICA100 edge 146)
2577)	rfMRI connectivity (ICA100 edge 147)
2578)	rfMRI connectivity (ICA100 edge 148)
2579)	rfMRI connectivity (ICA100 edge 149)
2580)	rfMRI connectivity (ICA100 edge 150)
2581)	rfMRI connectivity (ICA100 edge 151)
2582)	rfMRI connectivity (ICA100 edge 152)
2583)	rfMRI connectivity (ICA100 edge 153)
2584)	rfMRI connectivity (ICA100 edge 154)
2585)	rfMRI connectivity (ICA100 edge 155)
2586)	rfMRI connectivity (ICA100 edge 156)
2587)	rfMRI connectivity (ICA100 edge 157)
2588)	rfMRI connectivity (ICA100 edge 158)
2589)	rfMRI connectivity (ICA100 edge 159)
2590)	rfMRI connectivity (ICA100 edge 160)
2591)	rfMRI connectivity (ICA100 edge 161)
2592)	rfMRI connectivity (ICA100 edge 162)
2593)	rfMRI connectivity (ICA100 edge 163)
2594)	rfMRI connectivity (ICA100 edge 164)
2595)	rfMRI connectivity (ICA100 edge 165)
2596)	rfMRI connectivity (ICA100 edge 166)
2597)	rfMRI connectivity (ICA100 edge 167)
2598)	rfMRI connectivity (ICA100 edge 168)
2599)	rfMRI connectivity (ICA100 edge 169)
2600)	rfMRI connectivity (ICA100 edge 170)
2601)	rfMRI connectivity (ICA100 edge 171)
2602)	rfMRI connectivity (ICA100 edge 172)

2603)	rfMRI connectivity (ICA100 edge 173)
2604)	rfMRI connectivity (ICA100 edge 174)
2605)	rfMRI connectivity (ICA100 edge 175)
2606)	rfMRI connectivity (ICA100 edge 176)
2607)	rfMRI connectivity (ICA100 edge 177)
2608)	rfMRI connectivity (ICA100 edge 178)
2609)	rfMRI connectivity (ICA100 edge 179)
2610)	rfMRI connectivity (ICA100 edge 180)
2611)	rfMRI connectivity (ICA100 edge 181)
2612)	rfMRI connectivity (ICA100 edge 182)
2613)	rfMRI connectivity (ICA100 edge 183)
2614)	rfMRI connectivity (ICA100 edge 184)
2615)	rfMRI connectivity (ICA100 edge 185)
2616)	rfMRI connectivity (ICA100 edge 186)
2617)	rfMRI connectivity (ICA100 edge 187)
2618)	rfMRI connectivity (ICA100 edge 188)
2619)	rfMRI connectivity (ICA100 edge 189)
2620)	rfMRI connectivity (ICA100 edge 190)
2621)	rfMRI connectivity (ICA100 edge 191)
2622)	rfMRI connectivity (ICA100 edge 192)
2623)	rfMRI connectivity (ICA100 edge 193)
2624)	rfMRI connectivity (ICA100 edge 194)
2625)	rfMRI connectivity (ICA100 edge 195)
2626)	rfMRI connectivity (ICA100 edge 196)
2627)	rfMRI connectivity (ICA100 edge 197)
2628)	rfMRI connectivity (ICA100 edge 198)
2629)	rfMRI connectivity (ICA100 edge 199)
2630)	rfMRI connectivity (ICA100 edge 200)
2631)	rfMRI connectivity (ICA100 edge 201)
2632)	rfMRI connectivity (ICA100 edge 202)
2633)	rfMRI connectivity (ICA100 edge 203)
2634)	rfMRI connectivity (ICA100 edge 204)
2635)	rfMRI connectivity (ICA100 edge 205)
2636)	rfMRI connectivity (ICA100 edge 206)
2637)	rfMRI connectivity (ICA100 edge 207)
2638)	rfMRI connectivity (ICA100 edge 208)
2639)	rfMRI connectivity (ICA100 edge 209)
2640)	rfMRI connectivity (ICA100 edge 210)
2641)	rfMRI connectivity (ICA100 edge 211)
2642)	rfMRI connectivity (ICA100 edge 212)
2643)	rfMRI connectivity (ICA100 edge 213)
2644)	rfMRI connectivity (ICA100 edge 214)

2645)	rfMRI connectivity (ICA100 edge 215)
2646)	rfMRI connectivity (ICA100 edge 216)
2647)	rfMRI connectivity (ICA100 edge 217)
2648)	rfMRI connectivity (ICA100 edge 218)
2649)	rfMRI connectivity (ICA100 edge 219)
2650)	rfMRI connectivity (ICA100 edge 220)
2651)	rfMRI connectivity (ICA100 edge 221)
2652)	rfMRI connectivity (ICA100 edge 222)
2653)	rfMRI connectivity (ICA100 edge 223)
2654)	rfMRI connectivity (ICA100 edge 224)
2655)	rfMRI connectivity (ICA100 edge 225)
2656)	rfMRI connectivity (ICA100 edge 226)
2657)	rfMRI connectivity (ICA100 edge 227)
2658)	rfMRI connectivity (ICA100 edge 228)
2659)	rfMRI connectivity (ICA100 edge 229)
2660)	rfMRI connectivity (ICA100 edge 230)
2661)	rfMRI connectivity (ICA100 edge 231)
2662)	rfMRI connectivity (ICA100 edge 232)
2663)	rfMRI connectivity (ICA100 edge 233)
2664)	rfMRI connectivity (ICA100 edge 234)
2665)	rfMRI connectivity (ICA100 edge 235)
2666)	rfMRI connectivity (ICA100 edge 236)
2667)	rfMRI connectivity (ICA100 edge 237)
2668)	rfMRI connectivity (ICA100 edge 238)
2669)	rfMRI connectivity (ICA100 edge 239)
2670)	rfMRI connectivity (ICA100 edge 240)
2671)	rfMRI connectivity (ICA100 edge 241)
2672)	rfMRI connectivity (ICA100 edge 242)
2673)	rfMRI connectivity (ICA100 edge 243)
2674)	rfMRI connectivity (ICA100 edge 244)
2675)	rfMRI connectivity (ICA100 edge 245)
2676)	rfMRI connectivity (ICA100 edge 246)
2677)	rfMRI connectivity (ICA100 edge 247)
2678)	rfMRI connectivity (ICA100 edge 248)
2679)	rfMRI connectivity (ICA100 edge 249)
2680)	rfMRI connectivity (ICA100 edge 250)
2681)	rfMRI connectivity (ICA100 edge 251)
2682)	rfMRI connectivity (ICA100 edge 252)
2683)	rfMRI connectivity (ICA100 edge 253)
2684)	rfMRI connectivity (ICA100 edge 254)
2685)	rfMRI connectivity (ICA100 edge 255)
2686)	rfMRI connectivity (ICA100 edge 256)

2687)	rfMRI connectivity (ICA100 edge 257)
2688)	rfMRI connectivity (ICA100 edge 258)
2689)	rfMRI connectivity (ICA100 edge 259)
2690)	rfMRI connectivity (ICA100 edge 260)
2691)	rfMRI connectivity (ICA100 edge 261)
2692)	rfMRI connectivity (ICA100 edge 262)
2693)	rfMRI connectivity (ICA100 edge 263)
2694)	rfMRI connectivity (ICA100 edge 264)
2695)	rfMRI connectivity (ICA100 edge 265)
2696)	rfMRI connectivity (ICA100 edge 266)
2697)	rfMRI connectivity (ICA100 edge 267)
2698)	rfMRI connectivity (ICA100 edge 268)
2699)	rfMRI connectivity (ICA100 edge 269)
2700)	rfMRI connectivity (ICA100 edge 270)
2701)	rfMRI connectivity (ICA100 edge 271)
2702)	rfMRI connectivity (ICA100 edge 272)
2703)	rfMRI connectivity (ICA100 edge 273)
2704)	rfMRI connectivity (ICA100 edge 274)
2705)	rfMRI connectivity (ICA100 edge 275)
2706)	rfMRI connectivity (ICA100 edge 276)
2707)	rfMRI connectivity (ICA100 edge 277)
2708)	rfMRI connectivity (ICA100 edge 278)
2709)	rfMRI connectivity (ICA100 edge 279)
2710)	rfMRI connectivity (ICA100 edge 280)
2711)	rfMRI connectivity (ICA100 edge 281)
2712)	rfMRI connectivity (ICA100 edge 282)
2713)	rfMRI connectivity (ICA100 edge 283)
2714)	rfMRI connectivity (ICA100 edge 284)
2715)	rfMRI connectivity (ICA100 edge 285)
2716)	rfMRI connectivity (ICA100 edge 286)
2717)	rfMRI connectivity (ICA100 edge 287)
2718)	rfMRI connectivity (ICA100 edge 288)
2719)	rfMRI connectivity (ICA100 edge 289)
2720)	rfMRI connectivity (ICA100 edge 290)
2721)	rfMRI connectivity (ICA100 edge 291)
2722)	rfMRI connectivity (ICA100 edge 292)
2723)	rfMRI connectivity (ICA100 edge 293)
2724)	rfMRI connectivity (ICA100 edge 294)
2725)	rfMRI connectivity (ICA100 edge 295)
2726)	rfMRI connectivity (ICA100 edge 296)
2727)	rfMRI connectivity (ICA100 edge 297)
2728)	rfMRI connectivity (ICA100 edge 298)

2729)	rfMRI connectivity (ICA100 edge 299)
2730)	rfMRI connectivity (ICA100 edge 300)
2731)	rfMRI connectivity (ICA100 edge 301)
2732)	rfMRI connectivity (ICA100 edge 302)
2733)	rfMRI connectivity (ICA100 edge 303)
2734)	rfMRI connectivity (ICA100 edge 304)
2735)	rfMRI connectivity (ICA100 edge 305)
2736)	rfMRI connectivity (ICA100 edge 306)
2737)	rfMRI connectivity (ICA100 edge 307)
2738)	rfMRI connectivity (ICA100 edge 308)
2739)	rfMRI connectivity (ICA100 edge 309)
2740)	rfMRI connectivity (ICA100 edge 310)
2741)	rfMRI connectivity (ICA100 edge 311)
2742)	rfMRI connectivity (ICA100 edge 312)
2743)	rfMRI connectivity (ICA100 edge 313)
2744)	rfMRI connectivity (ICA100 edge 314)
2745)	rfMRI connectivity (ICA100 edge 315)
2746)	rfMRI connectivity (ICA100 edge 316)
2747)	rfMRI connectivity (ICA100 edge 317)
2748)	rfMRI connectivity (ICA100 edge 318)
2749)	rfMRI connectivity (ICA100 edge 319)
2750)	rfMRI connectivity (ICA100 edge 320)
2751)	rfMRI connectivity (ICA100 edge 321)
2752)	rfMRI connectivity (ICA100 edge 322)
2753)	rfMRI connectivity (ICA100 edge 323)
2754)	rfMRI connectivity (ICA100 edge 324)
2755)	rfMRI connectivity (ICA100 edge 325)
2756)	rfMRI connectivity (ICA100 edge 326)
2757)	rfMRI connectivity (ICA100 edge 327)
2758)	rfMRI connectivity (ICA100 edge 328)
2759)	rfMRI connectivity (ICA100 edge 329)
2760)	rfMRI connectivity (ICA100 edge 330)
2761)	rfMRI connectivity (ICA100 edge 331)
2762)	rfMRI connectivity (ICA100 edge 332)
2763)	rfMRI connectivity (ICA100 edge 333)
2764)	rfMRI connectivity (ICA100 edge 334)
2765)	rfMRI connectivity (ICA100 edge 335)
2766)	rfMRI connectivity (ICA100 edge 336)
2767)	rfMRI connectivity (ICA100 edge 337)
2768)	rfMRI connectivity (ICA100 edge 338)
2769)	rfMRI connectivity (ICA100 edge 339)
2770)	rfMRI connectivity (ICA100 edge 340)

2771)	rfMRI connectivity (ICA100 edge 341)
2772)	rfMRI connectivity (ICA100 edge 342)
2773)	rfMRI connectivity (ICA100 edge 343)
2774)	rfMRI connectivity (ICA100 edge 344)
2775)	rfMRI connectivity (ICA100 edge 345)
2776)	rfMRI connectivity (ICA100 edge 346)
2777)	rfMRI connectivity (ICA100 edge 347)
2778)	rfMRI connectivity (ICA100 edge 348)
2779)	rfMRI connectivity (ICA100 edge 349)
2780)	rfMRI connectivity (ICA100 edge 350)
2781)	rfMRI connectivity (ICA100 edge 351)
2782)	rfMRI connectivity (ICA100 edge 352)
2783)	rfMRI connectivity (ICA100 edge 353)
2784)	rfMRI connectivity (ICA100 edge 354)
2785)	rfMRI connectivity (ICA100 edge 355)
2786)	rfMRI connectivity (ICA100 edge 356)
2787)	rfMRI connectivity (ICA100 edge 357)
2788)	rfMRI connectivity (ICA100 edge 358)
2789)	rfMRI connectivity (ICA100 edge 359)
2790)	rfMRI connectivity (ICA100 edge 360)
2791)	rfMRI connectivity (ICA100 edge 361)
2792)	rfMRI connectivity (ICA100 edge 362)
2793)	rfMRI connectivity (ICA100 edge 363)
2794)	rfMRI connectivity (ICA100 edge 364)
2795)	rfMRI connectivity (ICA100 edge 365)
2796)	rfMRI connectivity (ICA100 edge 366)
2797)	rfMRI connectivity (ICA100 edge 367)
2798)	rfMRI connectivity (ICA100 edge 368)
2799)	rfMRI connectivity (ICA100 edge 369)
2800)	rfMRI connectivity (ICA100 edge 370)
2801)	rfMRI connectivity (ICA100 edge 371)
2802)	rfMRI connectivity (ICA100 edge 372)
2803)	rfMRI connectivity (ICA100 edge 373)
2804)	rfMRI connectivity (ICA100 edge 374)
2805)	rfMRI connectivity (ICA100 edge 375)
2806)	rfMRI connectivity (ICA100 edge 376)
2807)	rfMRI connectivity (ICA100 edge 377)
2808)	rfMRI connectivity (ICA100 edge 378)
2809)	rfMRI connectivity (ICA100 edge 379)
2810)	rfMRI connectivity (ICA100 edge 380)
2811)	rfMRI connectivity (ICA100 edge 381)
2812)	rfMRI connectivity (ICA100 edge 382)

2813)	rfMRI connectivity (ICA100 edge 383)
2814)	rfMRI connectivity (ICA100 edge 384)
2815)	rfMRI connectivity (ICA100 edge 385)
2816)	rfMRI connectivity (ICA100 edge 386)
2817)	rfMRI connectivity (ICA100 edge 387)
2818)	rfMRI connectivity (ICA100 edge 388)
2819)	rfMRI connectivity (ICA100 edge 389)
2820)	rfMRI connectivity (ICA100 edge 390)
2821)	rfMRI connectivity (ICA100 edge 391)
2822)	rfMRI connectivity (ICA100 edge 392)
2823)	rfMRI connectivity (ICA100 edge 393)
2824)	rfMRI connectivity (ICA100 edge 394)
2825)	rfMRI connectivity (ICA100 edge 395)
2826)	rfMRI connectivity (ICA100 edge 396)
2827)	rfMRI connectivity (ICA100 edge 397)
2828)	rfMRI connectivity (ICA100 edge 398)
2829)	rfMRI connectivity (ICA100 edge 399)
2830)	rfMRI connectivity (ICA100 edge 400)
2831)	rfMRI connectivity (ICA100 edge 401)
2832)	rfMRI connectivity (ICA100 edge 402)
2833)	rfMRI connectivity (ICA100 edge 403)
2834)	rfMRI connectivity (ICA100 edge 404)
2835)	rfMRI connectivity (ICA100 edge 405)
2836)	rfMRI connectivity (ICA100 edge 406)
2837)	rfMRI connectivity (ICA100 edge 407)
2838)	rfMRI connectivity (ICA100 edge 408)
2839)	rfMRI connectivity (ICA100 edge 409)
2840)	rfMRI connectivity (ICA100 edge 410)
2841)	rfMRI connectivity (ICA100 edge 411)
2842)	rfMRI connectivity (ICA100 edge 412)
2843)	rfMRI connectivity (ICA100 edge 413)
2844)	rfMRI connectivity (ICA100 edge 414)
2845)	rfMRI connectivity (ICA100 edge 415)
2846)	rfMRI connectivity (ICA100 edge 416)
2847)	rfMRI connectivity (ICA100 edge 417)
2848)	rfMRI connectivity (ICA100 edge 418)
2849)	rfMRI connectivity (ICA100 edge 419)
2850)	rfMRI connectivity (ICA100 edge 420)
2851)	rfMRI connectivity (ICA100 edge 421)
2852)	rfMRI connectivity (ICA100 edge 422)
2853)	rfMRI connectivity (ICA100 edge 423)
2854)	rfMRI connectivity (ICA100 edge 424)

2855)	rfMRI connectivity (ICA100 edge 425)
2856)	rfMRI connectivity (ICA100 edge 426)
2857)	rfMRI connectivity (ICA100 edge 427)
2858)	rfMRI connectivity (ICA100 edge 428)
2859)	rfMRI connectivity (ICA100 edge 429)
2860)	rfMRI connectivity (ICA100 edge 430)
2861)	rfMRI connectivity (ICA100 edge 431)
2862)	rfMRI connectivity (ICA100 edge 432)
2863)	rfMRI connectivity (ICA100 edge 433)
2864)	rfMRI connectivity (ICA100 edge 434)
2865)	rfMRI connectivity (ICA100 edge 435)
2866)	rfMRI connectivity (ICA100 edge 436)
2867)	rfMRI connectivity (ICA100 edge 437)
2868)	rfMRI connectivity (ICA100 edge 438)
2869)	rfMRI connectivity (ICA100 edge 439)
2870)	rfMRI connectivity (ICA100 edge 440)
2871)	rfMRI connectivity (ICA100 edge 441)
2872)	rfMRI connectivity (ICA100 edge 442)
2873)	rfMRI connectivity (ICA100 edge 443)
2874)	rfMRI connectivity (ICA100 edge 444)
2875)	rfMRI connectivity (ICA100 edge 445)
2876)	rfMRI connectivity (ICA100 edge 446)
2877)	rfMRI connectivity (ICA100 edge 447)
2878)	rfMRI connectivity (ICA100 edge 448)
2879)	rfMRI connectivity (ICA100 edge 449)
2880)	rfMRI connectivity (ICA100 edge 450)
2881)	rfMRI connectivity (ICA100 edge 451)
2882)	rfMRI connectivity (ICA100 edge 452)
2883)	rfMRI connectivity (ICA100 edge 453)
2884)	rfMRI connectivity (ICA100 edge 454)
2885)	rfMRI connectivity (ICA100 edge 455)
2886)	rfMRI connectivity (ICA100 edge 456)
2887)	rfMRI connectivity (ICA100 edge 457)
2888)	rfMRI connectivity (ICA100 edge 458)
2889)	rfMRI connectivity (ICA100 edge 459)
2890)	rfMRI connectivity (ICA100 edge 460)
2891)	rfMRI connectivity (ICA100 edge 461)
2892)	rfMRI connectivity (ICA100 edge 462)
2893)	rfMRI connectivity (ICA100 edge 463)
2894)	rfMRI connectivity (ICA100 edge 464)
2895)	rfMRI connectivity (ICA100 edge 465)
2896)	rfMRI connectivity (ICA100 edge 466)

2897)	rfMRI connectivity (ICA100 edge 467)
2898)	rfMRI connectivity (ICA100 edge 468)
2899)	rfMRI connectivity (ICA100 edge 469)
2900)	rfMRI connectivity (ICA100 edge 470)
2901)	rfMRI connectivity (ICA100 edge 471)
2902)	rfMRI connectivity (ICA100 edge 472)
2903)	rfMRI connectivity (ICA100 edge 473)
2904)	rfMRI connectivity (ICA100 edge 474)
2905)	rfMRI connectivity (ICA100 edge 475)
2906)	rfMRI connectivity (ICA100 edge 476)
2907)	rfMRI connectivity (ICA100 edge 477)
2908)	rfMRI connectivity (ICA100 edge 478)
2909)	rfMRI connectivity (ICA100 edge 479)
2910)	rfMRI connectivity (ICA100 edge 480)
2911)	rfMRI connectivity (ICA100 edge 481)
2912)	rfMRI connectivity (ICA100 edge 482)
2913)	rfMRI connectivity (ICA100 edge 483)
2914)	rfMRI connectivity (ICA100 edge 484)
2915)	rfMRI connectivity (ICA100 edge 485)
2916)	rfMRI connectivity (ICA100 edge 486)
2917)	rfMRI connectivity (ICA100 edge 487)
2918)	rfMRI connectivity (ICA100 edge 488)
2919)	rfMRI connectivity (ICA100 edge 489)
2920)	rfMRI connectivity (ICA100 edge 490)
2921)	rfMRI connectivity (ICA100 edge 491)
2922)	rfMRI connectivity (ICA100 edge 492)
2923)	rfMRI connectivity (ICA100 edge 493)
2924)	rfMRI connectivity (ICA100 edge 494)
2925)	rfMRI connectivity (ICA100 edge 495)
2926)	rfMRI connectivity (ICA100 edge 496)
2927)	rfMRI connectivity (ICA100 edge 497)
2928)	rfMRI connectivity (ICA100 edge 498)
2929)	rfMRI connectivity (ICA100 edge 499)
2930)	rfMRI connectivity (ICA100 edge 500)
2931)	rfMRI connectivity (ICA100 edge 501)
2932)	rfMRI connectivity (ICA100 edge 502)
2933)	rfMRI connectivity (ICA100 edge 503)
2934)	rfMRI connectivity (ICA100 edge 504)
2935)	rfMRI connectivity (ICA100 edge 505)
2936)	rfMRI connectivity (ICA100 edge 506)
2937)	rfMRI connectivity (ICA100 edge 507)
2938)	rfMRI connectivity (ICA100 edge 508)

2939)	rfMRI connectivity (ICA100 edge 509)
2940)	rfMRI connectivity (ICA100 edge 510)
2941)	rfMRI connectivity (ICA100 edge 511)
2942)	rfMRI connectivity (ICA100 edge 512)
2943)	rfMRI connectivity (ICA100 edge 513)
2944)	rfMRI connectivity (ICA100 edge 514)
2945)	rfMRI connectivity (ICA100 edge 515)
2946)	rfMRI connectivity (ICA100 edge 516)
2947)	rfMRI connectivity (ICA100 edge 517)
2948)	rfMRI connectivity (ICA100 edge 518)
2949)	rfMRI connectivity (ICA100 edge 519)
2950)	rfMRI connectivity (ICA100 edge 520)
2951)	rfMRI connectivity (ICA100 edge 521)
2952)	rfMRI connectivity (ICA100 edge 522)
2953)	rfMRI connectivity (ICA100 edge 523)
2954)	rfMRI connectivity (ICA100 edge 524)
2955)	rfMRI connectivity (ICA100 edge 525)
2956)	rfMRI connectivity (ICA100 edge 526)
2957)	rfMRI connectivity (ICA100 edge 527)
2958)	rfMRI connectivity (ICA100 edge 528)
2959)	rfMRI connectivity (ICA100 edge 529)
2960)	rfMRI connectivity (ICA100 edge 530)
2961)	rfMRI connectivity (ICA100 edge 531)
2962)	rfMRI connectivity (ICA100 edge 532)
2963)	rfMRI connectivity (ICA100 edge 533)
2964)	rfMRI connectivity (ICA100 edge 534)
2965)	rfMRI connectivity (ICA100 edge 535)
2966)	rfMRI connectivity (ICA100 edge 536)
2967)	rfMRI connectivity (ICA100 edge 537)
2968)	rfMRI connectivity (ICA100 edge 538)
2969)	rfMRI connectivity (ICA100 edge 539)
2970)	rfMRI connectivity (ICA100 edge 540)
2971)	rfMRI connectivity (ICA100 edge 541)
2972)	rfMRI connectivity (ICA100 edge 542)
2973)	rfMRI connectivity (ICA100 edge 543)
2974)	rfMRI connectivity (ICA100 edge 544)
2975)	rfMRI connectivity (ICA100 edge 545)
2976)	rfMRI connectivity (ICA100 edge 546)
2977)	rfMRI connectivity (ICA100 edge 547)
2978)	rfMRI connectivity (ICA100 edge 548)
2979)	rfMRI connectivity (ICA100 edge 549)
2980)	rfMRI connectivity (ICA100 edge 550)

2981)	rfMRI connectivity (ICA100 edge 551)
2982)	rfMRI connectivity (ICA100 edge 552)
2983)	rfMRI connectivity (ICA100 edge 553)
2984)	rfMRI connectivity (ICA100 edge 554)
2985)	rfMRI connectivity (ICA100 edge 555)
2986)	rfMRI connectivity (ICA100 edge 556)
2987)	rfMRI connectivity (ICA100 edge 557)
2988)	rfMRI connectivity (ICA100 edge 558)
2989)	rfMRI connectivity (ICA100 edge 559)
2990)	rfMRI connectivity (ICA100 edge 560)
2991)	rfMRI connectivity (ICA100 edge 561)
2992)	rfMRI connectivity (ICA100 edge 562)
2993)	rfMRI connectivity (ICA100 edge 563)
2994)	rfMRI connectivity (ICA100 edge 564)
2995)	rfMRI connectivity (ICA100 edge 565)
2996)	rfMRI connectivity (ICA100 edge 566)
2997)	rfMRI connectivity (ICA100 edge 567)
2998)	rfMRI connectivity (ICA100 edge 568)
2999)	rfMRI connectivity (ICA100 edge 569)
3000)	rfMRI connectivity (ICA100 edge 570)
3001)	rfMRI connectivity (ICA100 edge 571)
3002)	rfMRI connectivity (ICA100 edge 572)
3003)	rfMRI connectivity (ICA100 edge 573)
3004)	rfMRI connectivity (ICA100 edge 574)
3005)	rfMRI connectivity (ICA100 edge 575)
3006)	rfMRI connectivity (ICA100 edge 576)
3007)	rfMRI connectivity (ICA100 edge 577)
3008)	rfMRI connectivity (ICA100 edge 578)
3009)	rfMRI connectivity (ICA100 edge 579)
3010)	rfMRI connectivity (ICA100 edge 580)
3011)	rfMRI connectivity (ICA100 edge 581)
3012)	rfMRI connectivity (ICA100 edge 582)
3013)	rfMRI connectivity (ICA100 edge 583)
3014)	rfMRI connectivity (ICA100 edge 584)
3015)	rfMRI connectivity (ICA100 edge 585)
3016)	rfMRI connectivity (ICA100 edge 586)
3017)	rfMRI connectivity (ICA100 edge 587)
3018)	rfMRI connectivity (ICA100 edge 588)
3019)	rfMRI connectivity (ICA100 edge 589)
3020)	rfMRI connectivity (ICA100 edge 590)
3021)	rfMRI connectivity (ICA100 edge 591)
3022)	rfMRI connectivity (ICA100 edge 592)

3023)	rfMRI connectivity (ICA100 edge 593)
3024)	rfMRI connectivity (ICA100 edge 594)
3025)	rfMRI connectivity (ICA100 edge 595)
3026)	rfMRI connectivity (ICA100 edge 596)
3027)	rfMRI connectivity (ICA100 edge 597)
3028)	rfMRI connectivity (ICA100 edge 598)
3029)	rfMRI connectivity (ICA100 edge 599)
3030)	rfMRI connectivity (ICA100 edge 600)
3031)	rfMRI connectivity (ICA100 edge 601)
3032)	rfMRI connectivity (ICA100 edge 602)
3033)	rfMRI connectivity (ICA100 edge 603)
3034)	rfMRI connectivity (ICA100 edge 604)
3035)	rfMRI connectivity (ICA100 edge 605)
3036)	rfMRI connectivity (ICA100 edge 606)
3037)	rfMRI connectivity (ICA100 edge 607)
3038)	rfMRI connectivity (ICA100 edge 608)
3039)	rfMRI connectivity (ICA100 edge 609)
3040)	rfMRI connectivity (ICA100 edge 610)
3041)	rfMRI connectivity (ICA100 edge 611)
3042)	rfMRI connectivity (ICA100 edge 612)
3043)	rfMRI connectivity (ICA100 edge 613)
3044)	rfMRI connectivity (ICA100 edge 614)
3045)	rfMRI connectivity (ICA100 edge 615)
3046)	rfMRI connectivity (ICA100 edge 616)
3047)	rfMRI connectivity (ICA100 edge 617)
3048)	rfMRI connectivity (ICA100 edge 618)
3049)	rfMRI connectivity (ICA100 edge 619)
3050)	rfMRI connectivity (ICA100 edge 620)
3051)	rfMRI connectivity (ICA100 edge 621)
3052)	rfMRI connectivity (ICA100 edge 622)
3053)	rfMRI connectivity (ICA100 edge 623)
3054)	rfMRI connectivity (ICA100 edge 624)
3055)	rfMRI connectivity (ICA100 edge 625)
3056)	rfMRI connectivity (ICA100 edge 626)
3057)	rfMRI connectivity (ICA100 edge 627)
3058)	rfMRI connectivity (ICA100 edge 628)
3059)	rfMRI connectivity (ICA100 edge 629)
3060)	rfMRI connectivity (ICA100 edge 630)
3061)	rfMRI connectivity (ICA100 edge 631)
3062)	rfMRI connectivity (ICA100 edge 632)
3063)	rfMRI connectivity (ICA100 edge 633)
3064)	rfMRI connectivity (ICA100 edge 634)

3065)	rfMRI connectivity (ICA100 edge 635)
3066)	rfMRI connectivity (ICA100 edge 636)
3067)	rfMRI connectivity (ICA100 edge 637)
3068)	rfMRI connectivity (ICA100 edge 638)
3069)	rfMRI connectivity (ICA100 edge 639)
3070)	rfMRI connectivity (ICA100 edge 640)
3071)	rfMRI connectivity (ICA100 edge 641)
3072)	rfMRI connectivity (ICA100 edge 642)
3073)	rfMRI connectivity (ICA100 edge 643)
3074)	rfMRI connectivity (ICA100 edge 644)
3075)	rfMRI connectivity (ICA100 edge 645)
3076)	rfMRI connectivity (ICA100 edge 646)
3077)	rfMRI connectivity (ICA100 edge 647)
3078)	rfMRI connectivity (ICA100 edge 648)
3079)	rfMRI connectivity (ICA100 edge 649)
3080)	rfMRI connectivity (ICA100 edge 650)
3081)	rfMRI connectivity (ICA100 edge 651)
3082)	rfMRI connectivity (ICA100 edge 652)
3083)	rfMRI connectivity (ICA100 edge 653)
3084)	rfMRI connectivity (ICA100 edge 654)
3085)	rfMRI connectivity (ICA100 edge 655)
3086)	rfMRI connectivity (ICA100 edge 656)
3087)	rfMRI connectivity (ICA100 edge 657)
3088)	rfMRI connectivity (ICA100 edge 658)
3089)	rfMRI connectivity (ICA100 edge 659)
3090)	rfMRI connectivity (ICA100 edge 660)
3091)	rfMRI connectivity (ICA100 edge 661)
3092)	rfMRI connectivity (ICA100 edge 662)
3093)	rfMRI connectivity (ICA100 edge 663)
3094)	rfMRI connectivity (ICA100 edge 664)
3095)	rfMRI connectivity (ICA100 edge 665)
3096)	rfMRI connectivity (ICA100 edge 666)
3097)	rfMRI connectivity (ICA100 edge 667)
3098)	rfMRI connectivity (ICA100 edge 668)
3099)	rfMRI connectivity (ICA100 edge 669)
3100)	rfMRI connectivity (ICA100 edge 670)
3101)	rfMRI connectivity (ICA100 edge 671)
3102)	rfMRI connectivity (ICA100 edge 672)
3103)	rfMRI connectivity (ICA100 edge 673)
3104)	rfMRI connectivity (ICA100 edge 674)
3105)	rfMRI connectivity (ICA100 edge 675)
3106)	rfMRI connectivity (ICA100 edge 676)

3107)	rfMRI connectivity (ICA100 edge 677)
3108)	rfMRI connectivity (ICA100 edge 678)
3109)	rfMRI connectivity (ICA100 edge 679)
3110)	rfMRI connectivity (ICA100 edge 680)
3111)	rfMRI connectivity (ICA100 edge 681)
3112)	rfMRI connectivity (ICA100 edge 682)
3113)	rfMRI connectivity (ICA100 edge 683)
3114)	rfMRI connectivity (ICA100 edge 684)
3115)	rfMRI connectivity (ICA100 edge 685)
3116)	rfMRI connectivity (ICA100 edge 686)
3117)	rfMRI connectivity (ICA100 edge 687)
3118)	rfMRI connectivity (ICA100 edge 688)
3119)	rfMRI connectivity (ICA100 edge 689)
3120)	rfMRI connectivity (ICA100 edge 690)
3121)	rfMRI connectivity (ICA100 edge 691)
3122)	rfMRI connectivity (ICA100 edge 692)
3123)	rfMRI connectivity (ICA100 edge 693)
3124)	rfMRI connectivity (ICA100 edge 694)
3125)	rfMRI connectivity (ICA100 edge 695)
3126)	rfMRI connectivity (ICA100 edge 696)
3127)	rfMRI connectivity (ICA100 edge 697)
3128)	rfMRI connectivity (ICA100 edge 698)
3129)	rfMRI connectivity (ICA100 edge 699)
3130)	rfMRI connectivity (ICA100 edge 700)
3131)	rfMRI connectivity (ICA100 edge 701)
3132)	rfMRI connectivity (ICA100 edge 702)
3133)	rfMRI connectivity (ICA100 edge 703)
3134)	rfMRI connectivity (ICA100 edge 704)
3135)	rfMRI connectivity (ICA100 edge 705)
3136)	rfMRI connectivity (ICA100 edge 706)
3137)	rfMRI connectivity (ICA100 edge 707)
3138)	rfMRI connectivity (ICA100 edge 708)
3139)	rfMRI connectivity (ICA100 edge 709)
3140)	rfMRI connectivity (ICA100 edge 710)
3141)	rfMRI connectivity (ICA100 edge 711)
3142)	rfMRI connectivity (ICA100 edge 712)
3143)	rfMRI connectivity (ICA100 edge 713)
3144)	rfMRI connectivity (ICA100 edge 714)
3145)	rfMRI connectivity (ICA100 edge 715)
3146)	rfMRI connectivity (ICA100 edge 716)
3147)	rfMRI connectivity (ICA100 edge 717)
3148)	rfMRI connectivity (ICA100 edge 718)

3149)	rfMRI connectivity (ICA100 edge 719)
3150)	rfMRI connectivity (ICA100 edge 720)
3151)	rfMRI connectivity (ICA100 edge 721)
3152)	rfMRI connectivity (ICA100 edge 722)
3153)	rfMRI connectivity (ICA100 edge 723)
3154)	rfMRI connectivity (ICA100 edge 724)
3155)	rfMRI connectivity (ICA100 edge 725)
3156)	rfMRI connectivity (ICA100 edge 726)
3157)	rfMRI connectivity (ICA100 edge 727)
3158)	rfMRI connectivity (ICA100 edge 728)
3159)	rfMRI connectivity (ICA100 edge 729)
3160)	rfMRI connectivity (ICA100 edge 730)
3161)	rfMRI connectivity (ICA100 edge 731)
3162)	rfMRI connectivity (ICA100 edge 732)
3163)	rfMRI connectivity (ICA100 edge 733)
3164)	rfMRI connectivity (ICA100 edge 734)
3165)	rfMRI connectivity (ICA100 edge 735)
3166)	rfMRI connectivity (ICA100 edge 736)
3167)	rfMRI connectivity (ICA100 edge 737)
3168)	rfMRI connectivity (ICA100 edge 738)
3169)	rfMRI connectivity (ICA100 edge 739)
3170)	rfMRI connectivity (ICA100 edge 740)
3171)	rfMRI connectivity (ICA100 edge 741)
3172)	rfMRI connectivity (ICA100 edge 742)
3173)	rfMRI connectivity (ICA100 edge 743)
3174)	rfMRI connectivity (ICA100 edge 744)
3175)	rfMRI connectivity (ICA100 edge 745)
3176)	rfMRI connectivity (ICA100 edge 746)
3177)	rfMRI connectivity (ICA100 edge 747)
3178)	rfMRI connectivity (ICA100 edge 748)
3179)	rfMRI connectivity (ICA100 edge 749)
3180)	rfMRI connectivity (ICA100 edge 750)
3181)	rfMRI connectivity (ICA100 edge 751)
3182)	rfMRI connectivity (ICA100 edge 752)
3183)	rfMRI connectivity (ICA100 edge 753)
3184)	rfMRI connectivity (ICA100 edge 754)
3185)	rfMRI connectivity (ICA100 edge 755)
3186)	rfMRI connectivity (ICA100 edge 756)
3187)	rfMRI connectivity (ICA100 edge 757)
3188)	rfMRI connectivity (ICA100 edge 758)
3189)	rfMRI connectivity (ICA100 edge 759)
3190)	rfMRI connectivity (ICA100 edge 760)

3191)	rfMRI connectivity (ICA100 edge 761)
3192)	rfMRI connectivity (ICA100 edge 762)
3193)	rfMRI connectivity (ICA100 edge 763)
3194)	rfMRI connectivity (ICA100 edge 764)
3195)	rfMRI connectivity (ICA100 edge 765)
3196)	rfMRI connectivity (ICA100 edge 766)
3197)	rfMRI connectivity (ICA100 edge 767)
3198)	rfMRI connectivity (ICA100 edge 768)
3199)	rfMRI connectivity (ICA100 edge 769)
3200)	rfMRI connectivity (ICA100 edge 770)
3201)	rfMRI connectivity (ICA100 edge 771)
3202)	rfMRI connectivity (ICA100 edge 772)
3203)	rfMRI connectivity (ICA100 edge 773)
3204)	rfMRI connectivity (ICA100 edge 774)
3205)	rfMRI connectivity (ICA100 edge 775)
3206)	rfMRI connectivity (ICA100 edge 776)
3207)	rfMRI connectivity (ICA100 edge 777)
3208)	rfMRI connectivity (ICA100 edge 778)
3209)	rfMRI connectivity (ICA100 edge 779)
3210)	rfMRI connectivity (ICA100 edge 780)
3211)	rfMRI connectivity (ICA100 edge 781)
3212)	rfMRI connectivity (ICA100 edge 782)
3213)	rfMRI connectivity (ICA100 edge 783)
3214)	rfMRI connectivity (ICA100 edge 784)
3215)	rfMRI connectivity (ICA100 edge 785)
3216)	rfMRI connectivity (ICA100 edge 786)
3217)	rfMRI connectivity (ICA100 edge 787)
3218)	rfMRI connectivity (ICA100 edge 788)
3219)	rfMRI connectivity (ICA100 edge 789)
3220)	rfMRI connectivity (ICA100 edge 790)
3221)	rfMRI connectivity (ICA100 edge 791)
3222)	rfMRI connectivity (ICA100 edge 792)
3223)	rfMRI connectivity (ICA100 edge 793)
3224)	rfMRI connectivity (ICA100 edge 794)
3225)	rfMRI connectivity (ICA100 edge 795)
3226)	rfMRI connectivity (ICA100 edge 796)
3227)	rfMRI connectivity (ICA100 edge 797)
3228)	rfMRI connectivity (ICA100 edge 798)
3229)	rfMRI connectivity (ICA100 edge 799)
3230)	rfMRI connectivity (ICA100 edge 800)
3231)	rfMRI connectivity (ICA100 edge 801)
3232)	rfMRI connectivity (ICA100 edge 802)

3233)	rfMRI connectivity (ICA100 edge 803)
3234)	rfMRI connectivity (ICA100 edge 804)
3235)	rfMRI connectivity (ICA100 edge 805)
3236)	rfMRI connectivity (ICA100 edge 806)
3237)	rfMRI connectivity (ICA100 edge 807)
3238)	rfMRI connectivity (ICA100 edge 808)
3239)	rfMRI connectivity (ICA100 edge 809)
3240)	rfMRI connectivity (ICA100 edge 810)
3241)	rfMRI connectivity (ICA100 edge 811)
3242)	rfMRI connectivity (ICA100 edge 812)
3243)	rfMRI connectivity (ICA100 edge 813)
3244)	rfMRI connectivity (ICA100 edge 814)
3245)	rfMRI connectivity (ICA100 edge 815)
3246)	rfMRI connectivity (ICA100 edge 816)
3247)	rfMRI connectivity (ICA100 edge 817)
3248)	rfMRI connectivity (ICA100 edge 818)
3249)	rfMRI connectivity (ICA100 edge 819)
3250)	rfMRI connectivity (ICA100 edge 820)
3251)	rfMRI connectivity (ICA100 edge 821)
3252)	rfMRI connectivity (ICA100 edge 822)
3253)	rfMRI connectivity (ICA100 edge 823)
3254)	rfMRI connectivity (ICA100 edge 824)
3255)	rfMRI connectivity (ICA100 edge 825)
3256)	rfMRI connectivity (ICA100 edge 826)
3257)	rfMRI connectivity (ICA100 edge 827)
3258)	rfMRI connectivity (ICA100 edge 828)
3259)	rfMRI connectivity (ICA100 edge 829)
3260)	rfMRI connectivity (ICA100 edge 830)
3261)	rfMRI connectivity (ICA100 edge 831)
3262)	rfMRI connectivity (ICA100 edge 832)
3263)	rfMRI connectivity (ICA100 edge 833)
3264)	rfMRI connectivity (ICA100 edge 834)
3265)	rfMRI connectivity (ICA100 edge 835)
3266)	rfMRI connectivity (ICA100 edge 836)
3267)	rfMRI connectivity (ICA100 edge 837)
3268)	rfMRI connectivity (ICA100 edge 838)
3269)	rfMRI connectivity (ICA100 edge 839)
3270)	rfMRI connectivity (ICA100 edge 840)
3271)	rfMRI connectivity (ICA100 edge 841)
3272)	rfMRI connectivity (ICA100 edge 842)
3273)	rfMRI connectivity (ICA100 edge 843)
3274)	rfMRI connectivity (ICA100 edge 844)

3275)	rfMRI connectivity (ICA100 edge 845)
3276)	rfMRI connectivity (ICA100 edge 846)
3277)	rfMRI connectivity (ICA100 edge 847)
3278)	rfMRI connectivity (ICA100 edge 848)
3279)	rfMRI connectivity (ICA100 edge 849)
3280)	rfMRI connectivity (ICA100 edge 850)
3281)	rfMRI connectivity (ICA100 edge 851)
3282)	rfMRI connectivity (ICA100 edge 852)
3283)	rfMRI connectivity (ICA100 edge 853)
3284)	rfMRI connectivity (ICA100 edge 854)
3285)	rfMRI connectivity (ICA100 edge 855)
3286)	rfMRI connectivity (ICA100 edge 856)
3287)	rfMRI connectivity (ICA100 edge 857)
3288)	rfMRI connectivity (ICA100 edge 858)
3289)	rfMRI connectivity (ICA100 edge 859)
3290)	rfMRI connectivity (ICA100 edge 860)
3291)	rfMRI connectivity (ICA100 edge 861)
3292)	rfMRI connectivity (ICA100 edge 862)
3293)	rfMRI connectivity (ICA100 edge 863)
3294)	rfMRI connectivity (ICA100 edge 864)
3295)	rfMRI connectivity (ICA100 edge 865)
3296)	rfMRI connectivity (ICA100 edge 866)
3297)	rfMRI connectivity (ICA100 edge 867)
3298)	rfMRI connectivity (ICA100 edge 868)
3299)	rfMRI connectivity (ICA100 edge 869)
3300)	rfMRI connectivity (ICA100 edge 870)
3301)	rfMRI connectivity (ICA100 edge 871)
3302)	rfMRI connectivity (ICA100 edge 872)
3303)	rfMRI connectivity (ICA100 edge 873)
3304)	rfMRI connectivity (ICA100 edge 874)
3305)	rfMRI connectivity (ICA100 edge 875)
3306)	rfMRI connectivity (ICA100 edge 876)
3307)	rfMRI connectivity (ICA100 edge 877)
3308)	rfMRI connectivity (ICA100 edge 878)
3309)	rfMRI connectivity (ICA100 edge 879)
3310)	rfMRI connectivity (ICA100 edge 880)
3311)	rfMRI connectivity (ICA100 edge 881)
3312)	rfMRI connectivity (ICA100 edge 882)
3313)	rfMRI connectivity (ICA100 edge 883)
3314)	rfMRI connectivity (ICA100 edge 884)
3315)	rfMRI connectivity (ICA100 edge 885)
3316)	rfMRI connectivity (ICA100 edge 886)

3317)	rfMRI connectivity (ICA100 edge 887)
3318)	rfMRI connectivity (ICA100 edge 888)
3319)	rfMRI connectivity (ICA100 edge 889)
3320)	rfMRI connectivity (ICA100 edge 890)
3321)	rfMRI connectivity (ICA100 edge 891)
3322)	rfMRI connectivity (ICA100 edge 892)
3323)	rfMRI connectivity (ICA100 edge 893)
3324)	rfMRI connectivity (ICA100 edge 894)
3325)	rfMRI connectivity (ICA100 edge 895)
3326)	rfMRI connectivity (ICA100 edge 896)
3327)	rfMRI connectivity (ICA100 edge 897)
3328)	rfMRI connectivity (ICA100 edge 898)
3329)	rfMRI connectivity (ICA100 edge 899)
3330)	rfMRI connectivity (ICA100 edge 900)
3331)	rfMRI connectivity (ICA100 edge 901)
3332)	rfMRI connectivity (ICA100 edge 902)
3333)	rfMRI connectivity (ICA100 edge 903)
3334)	rfMRI connectivity (ICA100 edge 904)
3335)	rfMRI connectivity (ICA100 edge 905)
3336)	rfMRI connectivity (ICA100 edge 906)
3337)	rfMRI connectivity (ICA100 edge 907)
3338)	rfMRI connectivity (ICA100 edge 908)
3339)	rfMRI connectivity (ICA100 edge 909)
3340)	rfMRI connectivity (ICA100 edge 910)
3341)	rfMRI connectivity (ICA100 edge 911)
3342)	rfMRI connectivity (ICA100 edge 912)
3343)	rfMRI connectivity (ICA100 edge 913)
3344)	rfMRI connectivity (ICA100 edge 914)
3345)	rfMRI connectivity (ICA100 edge 915)
3346)	rfMRI connectivity (ICA100 edge 916)
3347)	rfMRI connectivity (ICA100 edge 917)
3348)	rfMRI connectivity (ICA100 edge 918)
3349)	rfMRI connectivity (ICA100 edge 919)
3350)	rfMRI connectivity (ICA100 edge 920)
3351)	rfMRI connectivity (ICA100 edge 921)
3352)	rfMRI connectivity (ICA100 edge 922)
3353)	rfMRI connectivity (ICA100 edge 923)
3354)	rfMRI connectivity (ICA100 edge 924)
3355)	rfMRI connectivity (ICA100 edge 925)
3356)	rfMRI connectivity (ICA100 edge 926)
3357)	rfMRI connectivity (ICA100 edge 927)
3358)	rfMRI connectivity (ICA100 edge 928)

3359)	rfMRI connectivity (ICA100 edge 929)
3360)	rfMRI connectivity (ICA100 edge 930)
3361)	rfMRI connectivity (ICA100 edge 931)
3362)	rfMRI connectivity (ICA100 edge 932)
3363)	rfMRI connectivity (ICA100 edge 933)
3364)	rfMRI connectivity (ICA100 edge 934)
3365)	rfMRI connectivity (ICA100 edge 935)
3366)	rfMRI connectivity (ICA100 edge 936)
3367)	rfMRI connectivity (ICA100 edge 937)
3368)	rfMRI connectivity (ICA100 edge 938)
3369)	rfMRI connectivity (ICA100 edge 939)
3370)	rfMRI connectivity (ICA100 edge 940)
3371)	rfMRI connectivity (ICA100 edge 941)
3372)	rfMRI connectivity (ICA100 edge 942)
3373)	rfMRI connectivity (ICA100 edge 943)
3374)	rfMRI connectivity (ICA100 edge 944)
3375)	rfMRI connectivity (ICA100 edge 945)
3376)	rfMRI connectivity (ICA100 edge 946)
3377)	rfMRI connectivity (ICA100 edge 947)
3378)	rfMRI connectivity (ICA100 edge 948)
3379)	rfMRI connectivity (ICA100 edge 949)
3380)	rfMRI connectivity (ICA100 edge 950)
3381)	rfMRI connectivity (ICA100 edge 951)
3382)	rfMRI connectivity (ICA100 edge 952)
3383)	rfMRI connectivity (ICA100 edge 953)
3384)	rfMRI connectivity (ICA100 edge 954)
3385)	rfMRI connectivity (ICA100 edge 955)
3386)	rfMRI connectivity (ICA100 edge 956)
3387)	rfMRI connectivity (ICA100 edge 957)
3388)	rfMRI connectivity (ICA100 edge 958)
3389)	rfMRI connectivity (ICA100 edge 959)
3390)	rfMRI connectivity (ICA100 edge 960)
3391)	rfMRI connectivity (ICA100 edge 961)
3392)	rfMRI connectivity (ICA100 edge 962)
3393)	rfMRI connectivity (ICA100 edge 963)
3394)	rfMRI connectivity (ICA100 edge 964)
3395)	rfMRI connectivity (ICA100 edge 965)
3396)	rfMRI connectivity (ICA100 edge 966)
3397)	rfMRI connectivity (ICA100 edge 967)
3398)	rfMRI connectivity (ICA100 edge 968)
3399)	rfMRI connectivity (ICA100 edge 969)
3400)	rfMRI connectivity (ICA100 edge 970)

3401)	rfMRI connectivity (ICA100 edge 971)
3402)	rfMRI connectivity (ICA100 edge 972)
3403)	rfMRI connectivity (ICA100 edge 973)
3404)	rfMRI connectivity (ICA100 edge 974)
3405)	rfMRI connectivity (ICA100 edge 975)
3406)	rfMRI connectivity (ICA100 edge 976)
3407)	rfMRI connectivity (ICA100 edge 977)
3408)	rfMRI connectivity (ICA100 edge 978)
3409)	rfMRI connectivity (ICA100 edge 979)
3410)	rfMRI connectivity (ICA100 edge 980)
3411)	rfMRI connectivity (ICA100 edge 981)
3412)	rfMRI connectivity (ICA100 edge 982)
3413)	rfMRI connectivity (ICA100 edge 983)
3414)	rfMRI connectivity (ICA100 edge 984)
3415)	rfMRI connectivity (ICA100 edge 985)
3416)	rfMRI connectivity (ICA100 edge 986)
3417)	rfMRI connectivity (ICA100 edge 987)
3418)	rfMRI connectivity (ICA100 edge 988)
3419)	rfMRI connectivity (ICA100 edge 989)
3420)	rfMRI connectivity (ICA100 edge 990)
3421)	rfMRI connectivity (ICA100 edge 991)
3422)	rfMRI connectivity (ICA100 edge 992)
3423)	rfMRI connectivity (ICA100 edge 993)
3424)	rfMRI connectivity (ICA100 edge 994)
3425)	rfMRI connectivity (ICA100 edge 995)
3426)	rfMRI connectivity (ICA100 edge 996)
3427)	rfMRI connectivity (ICA100 edge 997)
3428)	rfMRI connectivity (ICA100 edge 998)
3429)	rfMRI connectivity (ICA100 edge 999)
3430)	rfMRI connectivity (ICA100 edge 1000)
3431)	rfMRI connectivity (ICA100 edge 1001)
3432)	rfMRI connectivity (ICA100 edge 1002)
3433)	rfMRI connectivity (ICA100 edge 1003)
3434)	rfMRI connectivity (ICA100 edge 1004)
3435)	rfMRI connectivity (ICA100 edge 1005)
3436)	rfMRI connectivity (ICA100 edge 1006)
3437)	rfMRI connectivity (ICA100 edge 1007)
3438)	rfMRI connectivity (ICA100 edge 1008)
3439)	rfMRI connectivity (ICA100 edge 1009)
3440)	rfMRI connectivity (ICA100 edge 1010)
3441)	rfMRI connectivity (ICA100 edge 1011)
3442)	rfMRI connectivity (ICA100 edge 1012)

3443)	rfMRI connectivity (ICA100 edge 1013)
3444)	rfMRI connectivity (ICA100 edge 1014)
3445)	rfMRI connectivity (ICA100 edge 1015)
3446)	rfMRI connectivity (ICA100 edge 1016)
3447)	rfMRI connectivity (ICA100 edge 1017)
3448)	rfMRI connectivity (ICA100 edge 1018)
3449)	rfMRI connectivity (ICA100 edge 1019)
3450)	rfMRI connectivity (ICA100 edge 1020)
3451)	rfMRI connectivity (ICA100 edge 1021)
3452)	rfMRI connectivity (ICA100 edge 1022)
3453)	rfMRI connectivity (ICA100 edge 1023)
3454)	rfMRI connectivity (ICA100 edge 1024)
3455)	rfMRI connectivity (ICA100 edge 1025)
3456)	rfMRI connectivity (ICA100 edge 1026)
3457)	rfMRI connectivity (ICA100 edge 1027)
3458)	rfMRI connectivity (ICA100 edge 1028)
3459)	rfMRI connectivity (ICA100 edge 1029)
3460)	rfMRI connectivity (ICA100 edge 1030)
3461)	rfMRI connectivity (ICA100 edge 1031)
3462)	rfMRI connectivity (ICA100 edge 1032)
3463)	rfMRI connectivity (ICA100 edge 1033)
3464)	rfMRI connectivity (ICA100 edge 1034)
3465)	rfMRI connectivity (ICA100 edge 1035)
3466)	rfMRI connectivity (ICA100 edge 1036)
3467)	rfMRI connectivity (ICA100 edge 1037)
3468)	rfMRI connectivity (ICA100 edge 1038)
3469)	rfMRI connectivity (ICA100 edge 1039)
3470)	rfMRI connectivity (ICA100 edge 1040)
3471)	rfMRI connectivity (ICA100 edge 1041)
3472)	rfMRI connectivity (ICA100 edge 1042)
3473)	rfMRI connectivity (ICA100 edge 1043)
3474)	rfMRI connectivity (ICA100 edge 1044)
3475)	rfMRI connectivity (ICA100 edge 1045)
3476)	rfMRI connectivity (ICA100 edge 1046)
3477)	rfMRI connectivity (ICA100 edge 1047)
3478)	rfMRI connectivity (ICA100 edge 1048)
3479)	rfMRI connectivity (ICA100 edge 1049)
3480)	rfMRI connectivity (ICA100 edge 1050)
3481)	rfMRI connectivity (ICA100 edge 1051)
3482)	rfMRI connectivity (ICA100 edge 1052)
3483)	rfMRI connectivity (ICA100 edge 1053)
3484)	rfMRI connectivity (ICA100 edge 1054)

3485)	rfMRI connectivity (ICA100 edge 1055)
3486)	rfMRI connectivity (ICA100 edge 1056)
3487)	rfMRI connectivity (ICA100 edge 1057)
3488)	rfMRI connectivity (ICA100 edge 1058)
3489)	rfMRI connectivity (ICA100 edge 1059)
3490)	rfMRI connectivity (ICA100 edge 1060)
3491)	rfMRI connectivity (ICA100 edge 1061)
3492)	rfMRI connectivity (ICA100 edge 1062)
3493)	rfMRI connectivity (ICA100 edge 1063)
3494)	rfMRI connectivity (ICA100 edge 1064)
3495)	rfMRI connectivity (ICA100 edge 1065)
3496)	rfMRI connectivity (ICA100 edge 1066)
3497)	rfMRI connectivity (ICA100 edge 1067)
3498)	rfMRI connectivity (ICA100 edge 1068)
3499)	rfMRI connectivity (ICA100 edge 1069)
3500)	rfMRI connectivity (ICA100 edge 1070)
3501)	rfMRI connectivity (ICA100 edge 1071)
3502)	rfMRI connectivity (ICA100 edge 1072)
3503)	rfMRI connectivity (ICA100 edge 1073)
3504)	rfMRI connectivity (ICA100 edge 1074)
3505)	rfMRI connectivity (ICA100 edge 1075)
3506)	rfMRI connectivity (ICA100 edge 1076)
3507)	rfMRI connectivity (ICA100 edge 1077)
3508)	rfMRI connectivity (ICA100 edge 1078)
3509)	rfMRI connectivity (ICA100 edge 1079)
3510)	rfMRI connectivity (ICA100 edge 1080)
3511)	rfMRI connectivity (ICA100 edge 1081)
3512)	rfMRI connectivity (ICA100 edge 1082)
3513)	rfMRI connectivity (ICA100 edge 1083)
3514)	rfMRI connectivity (ICA100 edge 1084)
3515)	rfMRI connectivity (ICA100 edge 1085)
3516)	rfMRI connectivity (ICA100 edge 1086)
3517)	rfMRI connectivity (ICA100 edge 1087)
3518)	rfMRI connectivity (ICA100 edge 1088)
3519)	rfMRI connectivity (ICA100 edge 1089)
3520)	rfMRI connectivity (ICA100 edge 1090)
3521)	rfMRI connectivity (ICA100 edge 1091)
3522)	rfMRI connectivity (ICA100 edge 1092)
3523)	rfMRI connectivity (ICA100 edge 1093)
3524)	rfMRI connectivity (ICA100 edge 1094)
3525)	rfMRI connectivity (ICA100 edge 1095)
3526)	rfMRI connectivity (ICA100 edge 1096)

3527)	rfMRI connectivity (ICA100 edge 1097)
3528)	rfMRI connectivity (ICA100 edge 1098)
3529)	rfMRI connectivity (ICA100 edge 1099)
3530)	rfMRI connectivity (ICA100 edge 1100)
3531)	rfMRI connectivity (ICA100 edge 1101)
3532)	rfMRI connectivity (ICA100 edge 1102)
3533)	rfMRI connectivity (ICA100 edge 1103)
3534)	rfMRI connectivity (ICA100 edge 1104)
3535)	rfMRI connectivity (ICA100 edge 1105)
3536)	rfMRI connectivity (ICA100 edge 1106)
3537)	rfMRI connectivity (ICA100 edge 1107)
3538)	rfMRI connectivity (ICA100 edge 1108)
3539)	rfMRI connectivity (ICA100 edge 1109)
3540)	rfMRI connectivity (ICA100 edge 1110)
3541)	rfMRI connectivity (ICA100 edge 1111)
3542)	rfMRI connectivity (ICA100 edge 1112)
3543)	rfMRI connectivity (ICA100 edge 1113)
3544)	rfMRI connectivity (ICA100 edge 1114)
3545)	rfMRI connectivity (ICA100 edge 1115)
3546)	rfMRI connectivity (ICA100 edge 1116)
3547)	rfMRI connectivity (ICA100 edge 1117)
3548)	rfMRI connectivity (ICA100 edge 1118)
3549)	rfMRI connectivity (ICA100 edge 1119)
3550)	rfMRI connectivity (ICA100 edge 1120)
3551)	rfMRI connectivity (ICA100 edge 1121)
3552)	rfMRI connectivity (ICA100 edge 1122)
3553)	rfMRI connectivity (ICA100 edge 1123)
3554)	rfMRI connectivity (ICA100 edge 1124)
3555)	rfMRI connectivity (ICA100 edge 1125)
3556)	rfMRI connectivity (ICA100 edge 1126)
3557)	rfMRI connectivity (ICA100 edge 1127)
3558)	rfMRI connectivity (ICA100 edge 1128)
3559)	rfMRI connectivity (ICA100 edge 1129)
3560)	rfMRI connectivity (ICA100 edge 1130)
3561)	rfMRI connectivity (ICA100 edge 1131)
3562)	rfMRI connectivity (ICA100 edge 1132)
3563)	rfMRI connectivity (ICA100 edge 1133)
3564)	rfMRI connectivity (ICA100 edge 1134)
3565)	rfMRI connectivity (ICA100 edge 1135)
3566)	rfMRI connectivity (ICA100 edge 1136)
3567)	rfMRI connectivity (ICA100 edge 1137)
3568)	rfMRI connectivity (ICA100 edge 1138)

3569)	rfMRI connectivity (ICA100 edge 1139)
3570)	rfMRI connectivity (ICA100 edge 1140)
3571)	rfMRI connectivity (ICA100 edge 1141)
3572)	rfMRI connectivity (ICA100 edge 1142)
3573)	rfMRI connectivity (ICA100 edge 1143)
3574)	rfMRI connectivity (ICA100 edge 1144)
3575)	rfMRI connectivity (ICA100 edge 1145)
3576)	rfMRI connectivity (ICA100 edge 1146)
3577)	rfMRI connectivity (ICA100 edge 1147)
3578)	rfMRI connectivity (ICA100 edge 1148)
3579)	rfMRI connectivity (ICA100 edge 1149)
3580)	rfMRI connectivity (ICA100 edge 1150)
3581)	rfMRI connectivity (ICA100 edge 1151)
3582)	rfMRI connectivity (ICA100 edge 1152)
3583)	rfMRI connectivity (ICA100 edge 1153)
3584)	rfMRI connectivity (ICA100 edge 1154)
3585)	rfMRI connectivity (ICA100 edge 1155)
3586)	rfMRI connectivity (ICA100 edge 1156)
3587)	rfMRI connectivity (ICA100 edge 1157)
3588)	rfMRI connectivity (ICA100 edge 1158)
3589)	rfMRI connectivity (ICA100 edge 1159)
3590)	rfMRI connectivity (ICA100 edge 1160)
3591)	rfMRI connectivity (ICA100 edge 1161)
3592)	rfMRI connectivity (ICA100 edge 1162)
3593)	rfMRI connectivity (ICA100 edge 1163)
3594)	rfMRI connectivity (ICA100 edge 1164)
3595)	rfMRI connectivity (ICA100 edge 1165)
3596)	rfMRI connectivity (ICA100 edge 1166)
3597)	rfMRI connectivity (ICA100 edge 1167)
3598)	rfMRI connectivity (ICA100 edge 1168)
3599)	rfMRI connectivity (ICA100 edge 1169)
3600)	rfMRI connectivity (ICA100 edge 1170)
3601)	rfMRI connectivity (ICA100 edge 1171)
3602)	rfMRI connectivity (ICA100 edge 1172)
3603)	rfMRI connectivity (ICA100 edge 1173)
3604)	rfMRI connectivity (ICA100 edge 1174)
3605)	rfMRI connectivity (ICA100 edge 1175)
3606)	rfMRI connectivity (ICA100 edge 1176)
3607)	rfMRI connectivity (ICA100 edge 1177)
3608)	rfMRI connectivity (ICA100 edge 1178)
3609)	rfMRI connectivity (ICA100 edge 1179)
3610)	rfMRI connectivity (ICA100 edge 1180)

3611)	rfMRI connectivity (ICA100 edge 1181)
3612)	rfMRI connectivity (ICA100 edge 1182)
3613)	rfMRI connectivity (ICA100 edge 1183)
3614)	rfMRI connectivity (ICA100 edge 1184)
3615)	rfMRI connectivity (ICA100 edge 1185)
3616)	rfMRI connectivity (ICA100 edge 1186)
3617)	rfMRI connectivity (ICA100 edge 1187)
3618)	rfMRI connectivity (ICA100 edge 1188)
3619)	rfMRI connectivity (ICA100 edge 1189)
3620)	rfMRI connectivity (ICA100 edge 1190)
3621)	rfMRI connectivity (ICA100 edge 1191)
3622)	rfMRI connectivity (ICA100 edge 1192)
3623)	rfMRI connectivity (ICA100 edge 1193)
3624)	rfMRI connectivity (ICA100 edge 1194)
3625)	rfMRI connectivity (ICA100 edge 1195)
3626)	rfMRI connectivity (ICA100 edge 1196)
3627)	rfMRI connectivity (ICA100 edge 1197)
3628)	rfMRI connectivity (ICA100 edge 1198)
3629)	rfMRI connectivity (ICA100 edge 1199)
3630)	rfMRI connectivity (ICA100 edge 1200)
3631)	rfMRI connectivity (ICA100 edge 1201)
3632)	rfMRI connectivity (ICA100 edge 1202)
3633)	rfMRI connectivity (ICA100 edge 1203)
3634)	rfMRI connectivity (ICA100 edge 1204)
3635)	rfMRI connectivity (ICA100 edge 1205)
3636)	rfMRI connectivity (ICA100 edge 1206)
3637)	rfMRI connectivity (ICA100 edge 1207)
3638)	rfMRI connectivity (ICA100 edge 1208)
3639)	rfMRI connectivity (ICA100 edge 1209)
3640)	rfMRI connectivity (ICA100 edge 1210)
3641)	rfMRI connectivity (ICA100 edge 1211)
3642)	rfMRI connectivity (ICA100 edge 1212)
3643)	rfMRI connectivity (ICA100 edge 1213)
3644)	rfMRI connectivity (ICA100 edge 1214)
3645)	rfMRI connectivity (ICA100 edge 1215)
3646)	rfMRI connectivity (ICA100 edge 1216)
3647)	rfMRI connectivity (ICA100 edge 1217)
3648)	rfMRI connectivity (ICA100 edge 1218)
3649)	rfMRI connectivity (ICA100 edge 1219)
3650)	rfMRI connectivity (ICA100 edge 1220)
3651)	rfMRI connectivity (ICA100 edge 1221)
3652)	rfMRI connectivity (ICA100 edge 1222)

3653)	rfMRI connectivity (ICA100 edge 1223)
3654)	rfMRI connectivity (ICA100 edge 1224)
3655)	rfMRI connectivity (ICA100 edge 1225)
3656)	rfMRI connectivity (ICA100 edge 1226)
3657)	rfMRI connectivity (ICA100 edge 1227)
3658)	rfMRI connectivity (ICA100 edge 1228)
3659)	rfMRI connectivity (ICA100 edge 1229)
3660)	rfMRI connectivity (ICA100 edge 1230)
3661)	rfMRI connectivity (ICA100 edge 1231)
3662)	rfMRI connectivity (ICA100 edge 1232)
3663)	rfMRI connectivity (ICA100 edge 1233)
3664)	rfMRI connectivity (ICA100 edge 1234)
3665)	rfMRI connectivity (ICA100 edge 1235)
3666)	rfMRI connectivity (ICA100 edge 1236)
3667)	rfMRI connectivity (ICA100 edge 1237)
3668)	rfMRI connectivity (ICA100 edge 1238)
3669)	rfMRI connectivity (ICA100 edge 1239)
3670)	rfMRI connectivity (ICA100 edge 1240)
3671)	rfMRI connectivity (ICA100 edge 1241)
3672)	rfMRI connectivity (ICA100 edge 1242)
3673)	rfMRI connectivity (ICA100 edge 1243)
3674)	rfMRI connectivity (ICA100 edge 1244)
3675)	rfMRI connectivity (ICA100 edge 1245)
3676)	rfMRI connectivity (ICA100 edge 1246)
3677)	rfMRI connectivity (ICA100 edge 1247)
3678)	rfMRI connectivity (ICA100 edge 1248)
3679)	rfMRI connectivity (ICA100 edge 1249)
3680)	rfMRI connectivity (ICA100 edge 1250)
3681)	rfMRI connectivity (ICA100 edge 1251)
3682)	rfMRI connectivity (ICA100 edge 1252)
3683)	rfMRI connectivity (ICA100 edge 1253)
3684)	rfMRI connectivity (ICA100 edge 1254)
3685)	rfMRI connectivity (ICA100 edge 1255)
3686)	rfMRI connectivity (ICA100 edge 1256)
3687)	rfMRI connectivity (ICA100 edge 1257)
3688)	rfMRI connectivity (ICA100 edge 1258)
3689)	rfMRI connectivity (ICA100 edge 1259)
3690)	rfMRI connectivity (ICA100 edge 1260)
3691)	rfMRI connectivity (ICA100 edge 1261)
3692)	rfMRI connectivity (ICA100 edge 1262)
3693)	rfMRI connectivity (ICA100 edge 1263)
3694)	rfMRI connectivity (ICA100 edge 1264)

3695)	rfMRI connectivity (ICA100 edge 1265)
3696)	rfMRI connectivity (ICA100 edge 1266)
3697)	rfMRI connectivity (ICA100 edge 1267)
3698)	rfMRI connectivity (ICA100 edge 1268)
3699)	rfMRI connectivity (ICA100 edge 1269)
3700)	rfMRI connectivity (ICA100 edge 1270)
3701)	rfMRI connectivity (ICA100 edge 1271)
3702)	rfMRI connectivity (ICA100 edge 1272)
3703)	rfMRI connectivity (ICA100 edge 1273)
3704)	rfMRI connectivity (ICA100 edge 1274)
3705)	rfMRI connectivity (ICA100 edge 1275)
3706)	rfMRI connectivity (ICA100 edge 1276)
3707)	rfMRI connectivity (ICA100 edge 1277)
3708)	rfMRI connectivity (ICA100 edge 1278)
3709)	rfMRI connectivity (ICA100 edge 1279)
3710)	rfMRI connectivity (ICA100 edge 1280)
3711)	rfMRI connectivity (ICA100 edge 1281)
3712)	rfMRI connectivity (ICA100 edge 1282)
3713)	rfMRI connectivity (ICA100 edge 1283)
3714)	rfMRI connectivity (ICA100 edge 1284)
3715)	rfMRI connectivity (ICA100 edge 1285)
3716)	rfMRI connectivity (ICA100 edge 1286)
3717)	rfMRI connectivity (ICA100 edge 1287)
3718)	rfMRI connectivity (ICA100 edge 1288)
3719)	rfMRI connectivity (ICA100 edge 1289)
3720)	rfMRI connectivity (ICA100 edge 1290)
3721)	rfMRI connectivity (ICA100 edge 1291)
3722)	rfMRI connectivity (ICA100 edge 1292)
3723)	rfMRI connectivity (ICA100 edge 1293)
3724)	rfMRI connectivity (ICA100 edge 1294)
3725)	rfMRI connectivity (ICA100 edge 1295)
3726)	rfMRI connectivity (ICA100 edge 1296)
3727)	rfMRI connectivity (ICA100 edge 1297)
3728)	rfMRI connectivity (ICA100 edge 1298)
3729)	rfMRI connectivity (ICA100 edge 1299)
3730)	rfMRI connectivity (ICA100 edge 1300)
3731)	rfMRI connectivity (ICA100 edge 1301)
3732)	rfMRI connectivity (ICA100 edge 1302)
3733)	rfMRI connectivity (ICA100 edge 1303)
3734)	rfMRI connectivity (ICA100 edge 1304)
3735)	rfMRI connectivity (ICA100 edge 1305)
3736)	rfMRI connectivity (ICA100 edge 1306)

3737)	rfMRI connectivity (ICA100 edge 1307)
3738)	rfMRI connectivity (ICA100 edge 1308)
3739)	rfMRI connectivity (ICA100 edge 1309)
3740)	rfMRI connectivity (ICA100 edge 1310)
3741)	rfMRI connectivity (ICA100 edge 1311)
3742)	rfMRI connectivity (ICA100 edge 1312)
3743)	rfMRI connectivity (ICA100 edge 1313)
3744)	rfMRI connectivity (ICA100 edge 1314)
3745)	rfMRI connectivity (ICA100 edge 1315)
3746)	rfMRI connectivity (ICA100 edge 1316)
3747)	rfMRI connectivity (ICA100 edge 1317)
3748)	rfMRI connectivity (ICA100 edge 1318)
3749)	rfMRI connectivity (ICA100 edge 1319)
3750)	rfMRI connectivity (ICA100 edge 1320)
3751)	rfMRI connectivity (ICA100 edge 1321)
3752)	rfMRI connectivity (ICA100 edge 1322)
3753)	rfMRI connectivity (ICA100 edge 1323)
3754)	rfMRI connectivity (ICA100 edge 1324)
3755)	rfMRI connectivity (ICA100 edge 1325)
3756)	rfMRI connectivity (ICA100 edge 1326)
3757)	rfMRI connectivity (ICA100 edge 1327)
3758)	rfMRI connectivity (ICA100 edge 1328)
3759)	rfMRI connectivity (ICA100 edge 1329)
3760)	rfMRI connectivity (ICA100 edge 1330)
3761)	rfMRI connectivity (ICA100 edge 1331)
3762)	rfMRI connectivity (ICA100 edge 1332)
3763)	rfMRI connectivity (ICA100 edge 1333)
3764)	rfMRI connectivity (ICA100 edge 1334)
3765)	rfMRI connectivity (ICA100 edge 1335)
3766)	rfMRI connectivity (ICA100 edge 1336)
3767)	rfMRI connectivity (ICA100 edge 1337)
3768)	rfMRI connectivity (ICA100 edge 1338)
3769)	rfMRI connectivity (ICA100 edge 1339)
3770)	rfMRI connectivity (ICA100 edge 1340)
3771)	rfMRI connectivity (ICA100 edge 1341)
3772)	rfMRI connectivity (ICA100 edge 1342)
3773)	rfMRI connectivity (ICA100 edge 1343)
3774)	rfMRI connectivity (ICA100 edge 1344)
3775)	rfMRI connectivity (ICA100 edge 1345)
3776)	rfMRI connectivity (ICA100 edge 1346)
3777)	rfMRI connectivity (ICA100 edge 1347)
3778)	rfMRI connectivity (ICA100 edge 1348)

3779)	rfMRI connectivity (ICA100 edge 1349)
3780)	rfMRI connectivity (ICA100 edge 1350)
3781)	rfMRI connectivity (ICA100 edge 1351)
3782)	rfMRI connectivity (ICA100 edge 1352)
3783)	rfMRI connectivity (ICA100 edge 1353)
3784)	rfMRI connectivity (ICA100 edge 1354)
3785)	rfMRI connectivity (ICA100 edge 1355)
3786)	rfMRI connectivity (ICA100 edge 1356)
3787)	rfMRI connectivity (ICA100 edge 1357)
3788)	rfMRI connectivity (ICA100 edge 1358)
3789)	rfMRI connectivity (ICA100 edge 1359)
3790)	rfMRI connectivity (ICA100 edge 1360)
3791)	rfMRI connectivity (ICA100 edge 1361)
3792)	rfMRI connectivity (ICA100 edge 1362)
3793)	rfMRI connectivity (ICA100 edge 1363)
3794)	rfMRI connectivity (ICA100 edge 1364)
3795)	rfMRI connectivity (ICA100 edge 1365)
3796)	rfMRI connectivity (ICA100 edge 1366)
3797)	rfMRI connectivity (ICA100 edge 1367)
3798)	rfMRI connectivity (ICA100 edge 1368)
3799)	rfMRI connectivity (ICA100 edge 1369)
3800)	rfMRI connectivity (ICA100 edge 1370)
3801)	rfMRI connectivity (ICA100 edge 1371)
3802)	rfMRI connectivity (ICA100 edge 1372)
3803)	rfMRI connectivity (ICA100 edge 1373)
3804)	rfMRI connectivity (ICA100 edge 1374)
3805)	rfMRI connectivity (ICA100 edge 1375)
3806)	rfMRI connectivity (ICA100 edge 1376)
3807)	rfMRI connectivity (ICA100 edge 1377)
3808)	rfMRI connectivity (ICA100 edge 1378)
3809)	rfMRI connectivity (ICA100 edge 1379)
3810)	rfMRI connectivity (ICA100 edge 1380)
3811)	rfMRI connectivity (ICA100 edge 1381)
3812)	rfMRI connectivity (ICA100 edge 1382)
3813)	rfMRI connectivity (ICA100 edge 1383)
3814)	rfMRI connectivity (ICA100 edge 1384)
3815)	rfMRI connectivity (ICA100 edge 1385)
3816)	rfMRI connectivity (ICA100 edge 1386)
3817)	rfMRI connectivity (ICA100 edge 1387)
3818)	rfMRI connectivity (ICA100 edge 1388)
3819)	rfMRI connectivity (ICA100 edge 1389)
3820)	rfMRI connectivity (ICA100 edge 1390)

3821)	rfMRI connectivity (ICA100 edge 1391)
3822)	rfMRI connectivity (ICA100 edge 1392)
3823)	rfMRI connectivity (ICA100 edge 1393)
3824)	rfMRI connectivity (ICA100 edge 1394)
3825)	rfMRI connectivity (ICA100 edge 1395)
3826)	rfMRI connectivity (ICA100 edge 1396)
3827)	rfMRI connectivity (ICA100 edge 1397)
3828)	rfMRI connectivity (ICA100 edge 1398)
3829)	rfMRI connectivity (ICA100 edge 1399)
3830)	rfMRI connectivity (ICA100 edge 1400)
3831)	rfMRI connectivity (ICA100 edge 1401)
3832)	rfMRI connectivity (ICA100 edge 1402)
3833)	rfMRI connectivity (ICA100 edge 1403)
3834)	rfMRI connectivity (ICA100 edge 1404)
3835)	rfMRI connectivity (ICA100 edge 1405)
3836)	rfMRI connectivity (ICA100 edge 1406)
3837)	rfMRI connectivity (ICA100 edge 1407)
3838)	rfMRI connectivity (ICA100 edge 1408)
3839)	rfMRI connectivity (ICA100 edge 1409)
3840)	rfMRI connectivity (ICA100 edge 1410)
3841)	rfMRI connectivity (ICA100 edge 1411)
3842)	rfMRI connectivity (ICA100 edge 1412)
3843)	rfMRI connectivity (ICA100 edge 1413)
3844)	rfMRI connectivity (ICA100 edge 1414)
3845)	rfMRI connectivity (ICA100 edge 1415)
3846)	rfMRI connectivity (ICA100 edge 1416)
3847)	rfMRI connectivity (ICA100 edge 1417)
3848)	rfMRI connectivity (ICA100 edge 1418)
3849)	rfMRI connectivity (ICA100 edge 1419)
3850)	rfMRI connectivity (ICA100 edge 1420)
3851)	rfMRI connectivity (ICA100 edge 1421)
3852)	rfMRI connectivity (ICA100 edge 1422)
3853)	rfMRI connectivity (ICA100 edge 1423)
3854)	rfMRI connectivity (ICA100 edge 1424)
3855)	rfMRI connectivity (ICA100 edge 1425)
3856)	rfMRI connectivity (ICA100 edge 1426)
3857)	rfMRI connectivity (ICA100 edge 1427)
3858)	rfMRI connectivity (ICA100 edge 1428)
3859)	rfMRI connectivity (ICA100 edge 1429)
3860)	rfMRI connectivity (ICA100 edge 1430)
3861)	rfMRI connectivity (ICA100 edge 1431)
3862)	rfMRI connectivity (ICA100 edge 1432)

3863)	rfMRI connectivity (ICA100 edge 1433)
3864)	rfMRI connectivity (ICA100 edge 1434)
3865)	rfMRI connectivity (ICA100 edge 1435)
3866)	rfMRI connectivity (ICA100 edge 1436)
3867)	rfMRI connectivity (ICA100 edge 1437)
3868)	rfMRI connectivity (ICA100 edge 1438)
3869)	rfMRI connectivity (ICA100 edge 1439)
3870)	rfMRI connectivity (ICA100 edge 1440)
3871)	rfMRI connectivity (ICA100 edge 1441)
3872)	rfMRI connectivity (ICA100 edge 1442)
3873)	rfMRI connectivity (ICA100 edge 1443)
3874)	rfMRI connectivity (ICA100 edge 1444)
3875)	rfMRI connectivity (ICA100 edge 1445)
3876)	rfMRI connectivity (ICA100 edge 1446)
3877)	rfMRI connectivity (ICA100 edge 1447)
3878)	rfMRI connectivity (ICA100 edge 1448)
3879)	rfMRI connectivity (ICA100 edge 1449)
3880)	rfMRI connectivity (ICA100 edge 1450)
3881)	rfMRI connectivity (ICA100 edge 1451)
3882)	rfMRI connectivity (ICA100 edge 1452)
3883)	rfMRI connectivity (ICA100 edge 1453)
3884)	rfMRI connectivity (ICA100 edge 1454)
3885)	rfMRI connectivity (ICA100 edge 1455)
3886)	rfMRI connectivity (ICA100 edge 1456)
3887)	rfMRI connectivity (ICA100 edge 1457)
3888)	rfMRI connectivity (ICA100 edge 1458)
3889)	rfMRI connectivity (ICA100 edge 1459)
3890)	rfMRI connectivity (ICA100 edge 1460)
3891)	rfMRI connectivity (ICA100 edge 1461)
3892)	rfMRI connectivity (ICA100 edge 1462)
3893)	rfMRI connectivity (ICA100 edge 1463)
3894)	rfMRI connectivity (ICA100 edge 1464)
3895)	rfMRI connectivity (ICA100 edge 1465)
3896)	rfMRI connectivity (ICA100 edge 1466)
3897)	rfMRI connectivity (ICA100 edge 1467)
3898)	rfMRI connectivity (ICA100 edge 1468)
3899)	rfMRI connectivity (ICA100 edge 1469)
3900)	rfMRI connectivity (ICA100 edge 1470)
3901)	rfMRI connectivity (ICA100 edge 1471)
3902)	rfMRI connectivity (ICA100 edge 1472)
3903)	rfMRI connectivity (ICA100 edge 1473)
3904)	rfMRI connectivity (ICA100 edge 1474)

3905)	rfMRI connectivity (ICA100 edge 1475)
3906)	rfMRI connectivity (ICA100 edge 1476)
3907)	rfMRI connectivity (ICA100 edge 1477)
3908)	rfMRI connectivity (ICA100 edge 1478)
3909)	rfMRI connectivity (ICA100 edge 1479)
3910)	rfMRI connectivity (ICA100 edge 1480)
3911)	rfMRI connectivity (ICA100 edge 1481)
3912)	rfMRI connectivity (ICA100 edge 1482)
3913)	rfMRI connectivity (ICA100 edge 1483)
3914)	rfMRI connectivity (ICA100 edge 1484)
3915)	rfMRI connectivity (ICA100 edge 1485)
3916)	rfMRI connectivity ICA-features 1
3917)	rfMRI connectivity ICA-features 2
3918)	rfMRI connectivity ICA-features 3
3919)	rfMRI connectivity ICA-features 4
3920)	rfMRI connectivity ICA-features 5
3921)	rfMRI connectivity ICA-features 6

STable 2a: SNPs identified in GWAS associated with cannabis use

GWAS	SNP	Chr	BP	A1	A2	β	SE	*P*-value	*F* stats
Cannabis dependence or abuse (Levey et al 2023)	rs10835372	11	28643913	C	T	0.047	0.009	3.028e-08	27.28
	rs10986600	9	127928735	C	T	0.057	0.009	2.172e-10	34.67
	rs11711407	3	50225029	G	A	0.046	0.008	2.954e-08	29.06
	rs1526480	1	91209986	C	T	-0.052	0.008	5.906e-10	40.25
	rs159365	5	60500273	G	A	0.046	0.008	3.330e-08	29.06
	rs17007864	3	70876858	C	T	0.052	0.009	1.053e-09	30.22
	rs62461183	7	77716309	C	T	0.07	0.011	5.863e-10	43.64
	rs73247642*	4	47114175	C	T	-0.109	0.046	1.699e-02	6.50
	rs11608109*	11	113303448	C	G	-0.048	0.008	1.550e-08	29.25
	rs1637570	10	118619529	A	G	-0.05	0.009	4.786e-08	27.78
	rs2014920	11	113466565	T	G	0.053	0.009	8.004e-09	31.89
	rs2189010	7	114119430	A	G	0.048	0.009	1.280e-08	29.78
	rs3774800	3	49334768	A	G	-0.06	0.009	1.718e-12	40.00
	rs545943750	8	16059558	A	AT	-0.683	0.121	1.449e-08	38.94
	rs56070621	5	30825684	A	T	0.048	0.008	1.151e-08	29.25
	rs56372821	8	27436500	A	G	-0.089	0.012	7.272e-14	52.64
	rs62051488	16	72652784	A	C	-0.073	0.013	2.976e-08	39.33
	rs6690119	1	73580964	T	C	0.047	0.009	4.995e-08	27.28
	rs726610	3	85551403	T	C	-0.056	0.009	4.288e-11	35.11
	rs7519259	1	66434743	A	G	0.05	0.008	1.830e-09	35.00
	rs80030908	13	55159898	A	G	0.171	0.031	2.127e-08	184.27
	rs9344740	6	88619412	T	G	-0.056	0.009	8.344e-10	35.11
Lifetime cannabis use (Pasman et al 2018)	rs2875907	3	85518580	A	G	0.07	0.009	9.38E-17	68.89
	rs1448602	3	85780454	A	G	-0.062	0.01	6.55E-11	38.44
	rs7651996	3	85057349	T	G	0.049	0.008	2.37E-09	29.06
	rs10085617	7	3634711	A	T	0.046	0.008	2.93E-08	29.06
	rs9773390	8	81565692	T	C	-0.171	0.029	5.66E-09	104.22
	rs9919557	11	112877408	T	C	-0.055	0.009	9.94E-11	30.11
	rs10499	16	28915527	A	G	0.053	0.009	1.13E-09	31.89
	rs17761723	17	2107090	T	C	0.047	0.009	3.24E-08	27.28

Significance threshold was set at $p<5E-08$
*Proxy SNPs
Abbreviations: Chromosome (Chr), location in base pairs (BP), effect allele (A1), allele 2 (A2), Frequency of allele 1 (Freq A1), effect size beta (β), standard error of beta (SE)

STable 2b: SNPs identified in GWAS associated with brain IDPs

GWAS	SNP	Chr	BP	A1	A2	β	SE	*P*-value
FA Genu of Corpus callosum	rs72776055	5	85744869	G	T	3.33e-10	0.08	1.44e-9
rfMRI connectivity (ICA25 edge 21)	rs559370521	5	172852051	T	C	-7.55e-9	0.084	3.11e-8
	rs7113557	11	48317492	C	T	-5.86e-11	0.009	2.18e-9
rfMRI connectivity (ICA100 edge 55)	rs147270121	5	153248944	C	T	-3.43e-9	0.048	2.48e-8
	rs28649975	8	92275269	C	T	-1.21e-9	0.013	3.12e-8

Significance threshold was set at p<5E-08

Abbreviations: Chromosome (Chr), location in base pairs (BP), effect allele (A1), allele 2 (A2), Frequency of allele 1, effect size beta (β), standard error of beta (SE)

STable 3: Brain imaging confounds in UK Biobank (n = 613)

1)	Age_Site_1
2)	Age_Site_2
3)	Age_Site_3
4)	Sex_1_Site_1
5)	Sex_1_Site_2
6)	Sex_1_Site_3
7)	AgeSex_Site_1
8)	AgeSex_Site_2
9)	AgeSex_Site_3
10)	HeadSize_Site_1
11)	HeadSize_Site_2
12)	HeadSize_Site_3
13)	Site_1_vs_2
14)	Site_1_vs_3
15)	Batch_1_Site_1
16)	Batch_2_Site_1
17)	Batch_3_Site_1
18)	Batch_4_Site_1
19)	Batch_5_Site_1
20)	Batch_6_Site_1
21)	Batch_1_Site_2
22)	Batch_2_Site_2
23)	Batch_3_Site_2
24)	Batch_4_Site_2
25)	Batch_1_Site_3
26)	Batch_2_Site_3
27)	Batch_3_Site_3
28)	CMRR_1_Site_1
29)	CMRR_2_Site_1
30)	CMRR_3_Site_1
31)	CMRR_4_Site_1
32)	CMRR_5_Site_1
33)	CMRR_6_Site_1
34)	CMRR_7_Site_1
35)	CMRR_1_Site_2
36)	Protocol_1_Site_1
37)	Protocol_2_Site_1
38)	Protocol_3_Site_1
39)	Protocol_4_Site_1
40)	Protocol_5_Site_1

41)	Protocol_1_Site_3
42)	Service_Pack_1_Site_1
43)	ScanEvents_1_Site_1
44)	ScanEvents_2_Site_1
45)	ScanEvents_3_Site_1
46)	ScanEvents_4_Site_1
47)	ScanEvents_5_Site_1
48)	ScanEvents_6_Site_1
49)	ScanEvents_7_Site_1
50)	ScanEvents_8_Site_1
51)	ScanEvents_9_Site_1
52)	ScanEvents_10_Site_1
53)	ScanEvents_11_Site_1
54)	ScanEvents_12_Site_1
55)	ScanEvents_13_Site_1
56)	ScanEvents_14_Site_1
57)	ScanEvents_1_Site_2
58)	ScanEvents_2_Site_2
59)	ScanEvents_3_Site_2
60)	ScanEvents_4_Site_2
61)	ScanEvents_5_Site_2
62)	ScanEvents_6_Site_2
63)	ScanEvents_7_Site_2
64)	ScanEvents_1_Site_3
65)	ScanEvents_2_Site_3
66)	ScanEvents_3_Site_3
67)	ScanEvents_4_Site_3
68)	ScanEvents_5_Site_3
69)	Flipped_SWI_1_Site_1
70)	Flipped_SWI_1_Site_2
71)	Flipped_SWI_1_Site_3
72)	T1_and_T2_for_FreeSurfer_1_Site_1
73)	T1_and_T2_for_FreeSurfer_1_Site_2
74)	T1_and_T2_for_FreeSurfer_1_Site_3
75)	New_Eddy_1_Site_1
76)	New_Eddy_1_Site_2
77)	New_Eddy_1_Site_3
78)	SCALING_T1_1_Site_1
79)	SCALING_T2_FLAIR_1_Site_1
80)	SCALING_T2_FLAIR_2_Site_1
81)	SCALING_SWI_1_Site_1
82)	SCALING_SWI_1_Site_2

83)	SCALING_dMRI_1_Site_1
84)	SCALING_dMRI_1_Site_2
85)	SCALING_rfMRI_1_Site_1
86)	SCALING_tfMRI_1_Site_1
87)	TE_rfMRI_Site_1
88)	TE_tfMRI_Site_1
89)	TE_rfMRI_Site_2
90)	TE_tfMRI_Site_2
91)	TE_rfMRI_Site_3
92)	TE_tfMRI_Site_3
93)	StructHeadMotion_Site_1
94)	StructHeadMotion_Site_2
95)	StructHeadMotion_Site_3
96)	S_dirty_mean_Site_1
97)	S_dirty_median_Site_1
98)	S_dirty_p90_Site_1
99)	D_dirty_mean_Site_1
100)	D_dirty_median_Site_1
101)	D_dirty_p90_Site_1
102)	SD_dirty_mean_Site_1
103)	SD_dirty_median_Site_1
104)	SD_dirty_p90_Site_1
105)	S_clean_mean_Site_1
106)	S_clean_median_Site_1
107)	S_clean_p90_Site_1
108)	D_clean_mean_Site_1
109)	D_clean_median_Site_1
110)	D_clean_p90_Site_1
111)	SD_clean_mean_Site_1
112)	SD_clean_median_Site_1
113)	SD_clean_p90_Site_1
114)	S_dirty_mean_Site_2
115)	S_dirty_median_Site_2
116)	S_dirty_p90_Site_2
117)	D_dirty_mean_Site_2
118)	D_dirty_median_Site_2
119)	D_dirty_p90_Site_2
120)	SD_dirty_mean_Site_2
121)	SD_dirty_median_Site_2
122)	SD_dirty_p90_Site_2
123)	S_clean_mean_Site_2
124)	S_clean_median_Site_2

125)	S_clean_p90_Site_2
126)	D_clean_mean_Site_2
127)	D_clean_median_Site_2
128)	D_clean_p90_Site_2
129)	SD_clean_mean_Site_2
130)	SD_clean_median_Site_2
131)	SD_clean_p90_Site_2
132)	S_dirty_mean_Site_3
133)	S_dirty_median_Site_3
134)	S_dirty_p90_Site_3
135)	D_dirty_mean_Site_3
136)	D_dirty_median_Site_3
137)	D_dirty_p90_Site_3
138)	SD_dirty_mean_Site_3
139)	SD_dirty_median_Site_3
140)	SD_dirty_p90_Site_3
141)	S_clean_mean_Site_3
142)	S_clean_median_Site_3
143)	S_clean_p90_Site_3
144)	D_clean_mean_Site_3
145)	D_clean_median_Site_3
146)	D_clean_p90_Site_3
147)	SD_clean_mean_Site_3
148)	SD_clean_median_Site_3
149)	SD_clean_p90_Site_3
150)	HeadMotion_mean_rfMRI_abs_Site_1
151)	HeadMotion_median_rfMRI_abs_Site_1
152)	HeadMotion_p90_rfMRI_abs_Site_1
153)	HeadMotion_mean_rfMRI_rel_Site_1
154)	HeadMotion_median_rfMRI_rel_Site_1
155)	HeadMotion_p90_rfMRI_rel_Site_1
156)	HeadMotion_mean_tfMRI_abs_Site_1
157)	HeadMotion_median_tfMRI_abs_Site_1
158)	HeadMotion_p90_tfMRI_abs_Site_1
159)	HeadMotion_mean_tfMRI_rel_Site_1
160)	HeadMotion_median_tfMRI_rel_Site_1
161)	HeadMotion_p90_tfMRI_rel_Site_1
162)	HeadMotion_mean_dMRI_abs_Site_1
163)	HeadMotion_median_dMRI_abs_Site_1
164)	HeadMotion_p90_dMRI_abs_Site_1
165)	HeadMotion_mean_dMRI_rel_Site_1
166)	HeadMotion_median_dMRI_rel_Site_1

167)	HeadMotion_p90_dMRI_rel_Site_1
168)	HeadMotion_p90_dMRI_outliers_Site_1
169)	HeadMotion_mean_rfMRI_abs_Site_2
170)	HeadMotion_median_rfMRI_abs_Site_2
171)	HeadMotion_p90_rfMRI_abs_Site_2
172)	HeadMotion_mean_rfMRI_rel_Site_2
173)	HeadMotion_median_rfMRI_rel_Site_2
174)	HeadMotion_p90_rfMRI_rel_Site_2
175)	HeadMotion_mean_tfMRI_abs_Site_2
176)	HeadMotion_median_tfMRI_abs_Site_2
177)	HeadMotion_p90_tfMRI_abs_Site_2
178)	HeadMotion_mean_tfMRI_rel_Site_2
179)	HeadMotion_median_tfMRI_rel_Site_2
180)	HeadMotion_p90_tfMRI_rel_Site_2
181)	HeadMotion_mean_dMRI_abs_Site_2
182)	HeadMotion_median_dMRI_abs_Site_2
183)	HeadMotion_p90_dMRI_abs_Site_2
184)	HeadMotion_mean_dMRI_rel_Site_2
185)	HeadMotion_median_dMRI_rel_Site_2
186)	HeadMotion_p90_dMRI_rel_Site_2
187)	HeadMotion_p90_dMRI_outliers_Site_2
188)	HeadMotion_mean_rfMRI_abs_Site_3
189)	HeadMotion_median_rfMRI_abs_Site_3
190)	HeadMotion_p90_rfMRI_abs_Site_3
191)	HeadMotion_mean_rfMRI_rel_Site_3
192)	HeadMotion_median_rfMRI_rel_Site_3
193)	HeadMotion_p90_rfMRI_rel_Site_3
194)	HeadMotion_mean_tfMRI_abs_Site_3
195)	HeadMotion_median_tfMRI_abs_Site_3
196)	HeadMotion_p90_tfMRI_abs_Site_3
197)	HeadMotion_mean_tfMRI_rel_Site_3
198)	HeadMotion_median_tfMRI_rel_Site_3
199)	HeadMotion_p90_tfMRI_rel_Site_3
200)	HeadMotion_mean_dMRI_abs_Site_3
201)	HeadMotion_median_dMRI_abs_Site_3
202)	HeadMotion_p90_dMRI_abs_Site_3
203)	HeadMotion_mean_dMRI_rel_Site_3
204)	HeadMotion_median_dMRI_rel_Site_3
205)	HeadMotion_p90_dMRI_rel_Site_3
206)	HeadMotion_p90_dMRI_outliers_Site_3
207)	HeadMotionST_mean_inplane_time_Site_1
208)	HeadMotionST_median_inplane_time_Site_1

209)	HeadMotionST_p90_inplane_time_Site_1
210)	HeadMotionST_mean_Z_time_Site_1
211)	HeadMotionST_median_Z_time_Site_1
212)	HeadMotionST_p90_Z_time_Site_1
213)	HeadMotionST_median_inplane_space_Site_1
214)	HeadMotionST_p90_inplane_space_Site_1
215)	HeadMotionST_median_Z_space_Site_1
216)	HeadMotionST_p90_Z_space_Site_1
217)	HeadMotionST_mean_inplane_time_Site_2
218)	HeadMotionST_median_inplane_time_Site_2
219)	HeadMotionST_p90_inplane_time_Site_2
220)	HeadMotionST_mean_Z_time_Site_2
221)	HeadMotionST_median_Z_time_Site_2
222)	HeadMotionST_p90_Z_time_Site_2
223)	HeadMotionST_median_inplane_space_Site_2
224)	HeadMotionST_p90_inplane_space_Site_2
225)	HeadMotionST_median_Z_space_Site_2
226)	HeadMotionST_p90_Z_space_Site_2
227)	HeadMotionST_mean_inplane_time_Site_3
228)	HeadMotionST_median_inplane_time_Site_3
229)	HeadMotionST_p90_inplane_time_Site_3
230)	HeadMotionST_mean_Z_time_Site_3
231)	HeadMotionST_median_Z_time_Site_3
232)	HeadMotionST_p90_Z_time_Site_3
233)	HeadMotionST_median_inplane_space_Site_3
234)	HeadMotionST_p90_inplane_space_Site_3
235)	HeadMotionST_median_Z_space_Site_3
236)	HeadMotionST_p90_Z_space_Site_3
237)	TablePos_COG_X_Site_1
238)	TablePos_COG_Y_Site_1
239)	TablePos_COG_Z_Site_1
240)	TablePos_Table_Site_1
241)	TablePos_COG_X_Site_2
242)	TablePos_COG_Y_Site_2
243)	TablePos_COG_Z_Site_2
244)	TablePos_Table_Site_2
245)	TablePos_COG_X_Site_3
246)	TablePos_COG_Y_Site_3
247)	TablePos_COG_Z_Site_3
248)	TablePos_Table_Site_3
249)	Eddy_QC_X_Site_1
250)	Eddy_QC_Y_Site_1

251)	Eddy_QC_Z_Site_1
252)	YTranslation_Site_1
253)	Eddy_QC_X_Site_2
254)	Eddy_QC_Y_Site_2
255)	Eddy_QC_Z_Site_2
256)	YTranslation_Site_2
257)	Eddy_QC_X_Site_3
258)	Eddy_QC_Y_Site_3
259)	Eddy_QC_Z_Site_3
260)	YTranslation_Site_3
261)	Age_Site_1_squared
262)	Age_Site_2_squared
263)	Age_Site_3_squared
264)	Age_Site_1_inormal
265)	Age_Site_2_inormal
266)	Age_Site_3_inormal
267)	Age_Site_1_squared_inormal
268)	Age_Site_2_squared_inormal
269)	Age_Site_3_squared_inormal
270)	AgeSex_Site_1_squared
271)	AgeSex_Site_2_squared
272)	AgeSex_Site_3_squared
273)	AgeSex_Site_1_inormal
274)	AgeSex_Site_2_inormal
275)	AgeSex_Site_3_inormal
276)	AgeSex_Site_1_squared_inormal
277)	AgeSex_Site_2_squared_inormal
278)	AgeSex_Site_3_squared_inormal
279)	HeadSize_Site_1_squared
280)	HeadSize_Site_3_squared
281)	HeadSize_Site_1_inormal
282)	HeadSize_Site_3_inormal
283)	HeadSize_Site_3_squared_inormal
284)	StructHeadMotion_Site_1_squared
285)	StructHeadMotion_Site_2_squared
286)	StructHeadMotion_Site_3_squared
287)	StructHeadMotion_Site_1_inormal
288)	StructHeadMotion_Site_2_inormal
289)	StructHeadMotion_Site_3_inormal
290)	StructHeadMotion_Site_1_squared_inormal
291)	S_dirty_p90_Site_1_squared
292)	D_dirty_median_Site_1_squared

293)	SD_dirty_mean_Site_1_squared
294)	SD_dirty_p90_Site_1_squared
295)	S_clean_mean_Site_1_squared
296)	S_clean_median_Site_1_squared
297)	S_clean_p90_Site_1_squared
298)	D_clean_mean_Site_1_squared
299)	D_clean_median_Site_1_squared
300)	D_clean_p90_Site_1_squared
301)	SD_clean_mean_Site_1_squared
302)	SD_clean_median_Site_1_squared
303)	SD_clean_p90_Site_1_squared
304)	S_dirty_p90_Site_2_squared
305)	SD_dirty_p90_Site_2_squared
306)	S_clean_mean_Site_2_squared
307)	S_clean_median_Site_2_squared
308)	S_clean_p90_Site_2_squared
309)	D_clean_mean_Site_2_squared
310)	D_clean_median_Site_2_squared
311)	SD_clean_mean_Site_2_squared
312)	SD_clean_median_Site_2_squared
313)	SD_clean_p90_Site_2_squared
314)	S_dirty_p90_Site_3_squared
315)	SD_dirty_p90_Site_3_squared
316)	S_clean_mean_Site_3_squared
317)	S_clean_median_Site_3_squared
318)	S_clean_p90_Site_3_squared
319)	D_clean_mean_Site_3_squared
320)	D_clean_median_Site_3_squared
321)	SD_clean_mean_Site_3_squared
322)	SD_clean_median_Site_3_squared
323)	SD_clean_p90_Site_3_squared
324)	S_dirty_p90_Site_1_inormal
325)	S_clean_mean_Site_1_inormal
326)	S_clean_median_Site_1_inormal
327)	S_clean_p90_Site_1_inormal
328)	D_clean_mean_Site_1_inormal
329)	D_clean_median_Site_1_inormal
330)	D_clean_p90_Site_1_inormal
331)	SD_clean_mean_Site_1_inormal
332)	SD_clean_median_Site_1_inormal
333)	SD_clean_p90_Site_1_inormal
334)	S_dirty_p90_Site_2_inormal

335)	S_clean_mean_Site_2_inormal
336)	S_clean_median_Site_2_inormal
337)	S_clean_p90_Site_2_inormal
338)	D_clean_mean_Site_2_inormal
339)	D_clean_median_Site_2_inormal
340)	D_clean_p90_Site_2_inormal
341)	SD_clean_mean_Site_2_inormal
342)	SD_clean_median_Site_2_inormal
343)	SD_clean_p90_Site_2_inormal
344)	S_dirty_p90_Site_3_inormal
345)	S_clean_mean_Site_3_inormal
346)	S_clean_median_Site_3_inormal
347)	S_clean_p90_Site_3_inormal
348)	D_clean_mean_Site_3_inormal
349)	D_clean_median_Site_3_inormal
350)	D_clean_p90_Site_3_inormal
351)	SD_clean_mean_Site_3_inormal
352)	SD_clean_median_Site_3_inormal
353)	SD_clean_p90_Site_3_inormal
354)	S_clean_mean_Site_1_squared_inormal
355)	S_clean_median_Site_1_squared_inormal
356)	S_clean_p90_Site_1_squared_inormal
357)	D_clean_mean_Site_1_squared_inormal
358)	D_clean_median_Site_1_squared_inormal
359)	SD_clean_mean_Site_1_squared_inormal
360)	SD_clean_median_Site_1_squared_inormal
361)	SD_clean_p90_Site_1_squared_inormal
362)	S_dirty_p90_Site_2_squared_inormal
363)	S_clean_mean_Site_2_squared_inormal
364)	S_clean_p90_Site_2_squared_inormal
365)	D_clean_mean_Site_2_squared_inormal
366)	D_clean_median_Site_2_squared_inormal
367)	SD_clean_mean_Site_2_squared_inormal
368)	SD_clean_median_Site_2_squared_inormal
369)	SD_clean_p90_Site_2_squared_inormal
370)	S_clean_mean_Site_3_squared_inormal
371)	S_clean_median_Site_3_squared_inormal
372)	S_clean_p90_Site_3_squared_inormal
373)	D_clean_mean_Site_3_squared_inormal
374)	D_clean_median_Site_3_squared_inormal
375)	SD_clean_mean_Site_3_squared_inormal
376)	SD_clean_median_Site_3_squared_inormal

377)	SD_clean_p90_Site_3_squared_inormal
378)	TablePos_COG_Z_Site_1_squared
379)	TablePos_Table_Site_1_squared
380)	TablePos_COG_Z_Site_2_squared
381)	TablePos_COG_Z_Site_3_squared
382)	TablePos_COG_Z_Site_1_inormal
383)	TablePos_COG_Z_Site_2_inormal
384)	TablePos_COG_Z_Site_3_inormal
385)	TablePos_COG_Z_Site_1_squared_inormal
386)	TablePos_COG_Z_Site_2_squared_inormal
387)	TablePos_COG_Z_Site_3_squared_inormal
388)	Eddy_QC_Y_Site_1_squared
389)	Eddy_QC_Y_Site_2_squared
390)	Eddy_QC_Y_Site_3_squared
391)	Eddy_QC_Y_Site_1_inormal
392)	Eddy_QC_Y_Site_2_inormal
393)	Eddy_QC_Y_Site_3_inormal
394)	Eddy_QC_Y_Site_1_squared_inormal
395)	Age_Site_1__x__TablePos_Table_Site_1
396)	Age_Site_2__x__HeadMotion_p90_tfMRI_abs_Site_2
397)	Sex_1_Site_1__x__TablePos_COG_Z_Site_1
398)	Sex_1_Site_1__x__TablePos_Table_Site_1
399)	Sex_1_Site_2__x__TablePos_COG_Z_Site_2
400)	Sex_1_Site_3__x__TablePos_COG_Z_Site_3
401)	HeadSize_Site_1__x__TablePos_COG_Z_Site_1
402)	HeadSize_Site_1__x__TablePos_Table_Site_1
403)	HeadSize_Site_3__x__TablePos_COG_Z_Site_3
404)	HeadSize_Site_3__x__HeadSize_Site_3_squared
405)	Batch_1_Site_1__x__SCALING_tfMRI_1_Site_1
406)	Batch_2_Site_1__x__SCALING_tfMRI_1_Site_1
407)	Batch_3_Site_1__x__SCALING_tfMRI_1_Site_1
408)	Batch_1_Site_2__x__SCALING_SWI_1_Site_2
409)	Batch_2_Site_2__x__SCALING_SWI_1_Site_2
410)	Batch_3_Site_2__x__SCALING_SWI_1_Site_2
411)	Batch_4_Site_2__x__SCALING_SWI_1_Site_2
412)	Batch_4_Site_2__x__D_clean_p90_Site_2_inormal
413)	Batch_1_Site_3__x__Batch_2_Site_3
414)	Batch_1_Site_3__x__Batch_3_Site_3
415)	Batch_1_Site_3__x__ScanEvents_1_Site_3
416)	Batch_1_Site_3__x__ScanEvents_2_Site_3
417)	Batch_1_Site_3__x__ScanEvents_3_Site_3
418)	Batch_1_Site_3__x__ScanEvents_4_Site_3

419)	Batch_1_Site_3__x__ScanEvents_5_Site_3
420)	Batch_1_Site_3__x__TE_rfMRI_Site_3
421)	Batch_1_Site_3__x__Eddy_QC_Y_Site_3
422)	Batch_2_Site_3__x__Batch_3_Site_3
423)	Batch_2_Site_3__x__ScanEvents_1_Site_3
424)	Batch_2_Site_3__x__ScanEvents_2_Site_3
425)	Batch_2_Site_3__x__ScanEvents_3_Site_3
426)	Batch_2_Site_3__x__ScanEvents_4_Site_3
427)	Batch_2_Site_3__x__ScanEvents_5_Site_3
428)	Batch_2_Site_3__x__TE_rfMRI_Site_3
429)	Batch_3_Site_3__x__ScanEvents_1_Site_3
430)	Batch_3_Site_3__x__ScanEvents_2_Site_3
431)	Batch_3_Site_3__x__ScanEvents_3_Site_3
432)	Batch_3_Site_3__x__ScanEvents_4_Site_3
433)	Batch_3_Site_3__x__ScanEvents_5_Site_3
434)	Batch_3_Site_3__x__TE_rfMRI_Site_3
435)	CMRR_1_Site_1__x__SCALING_tfMRI_1_Site_1
436)	CMRR_1_Site_2__x__SCALING_SWI_1_Site_2
437)	Protocol_4_Site_1__x__Eddy_QC_Y_Site_1
438)	Service_Pack_1_Site_1__x__Eddy_QC_Y_Site_1
439)	ScanEvents_1_Site_2__x__SCALING_SWI_1_Site_2
440)	ScanEvents_2_Site_2__x__SCALING_SWI_1_Site_2
441)	ScanEvents_3_Site_2__x__SCALING_SWI_1_Site_2
442)	ScanEvents_5_Site_2__x__SCALING_SWI_1_Site_2
443)	ScanEvents_7_Site_2__x__SCALING_SWI_1_Site_2
444)	ScanEvents_7_Site_2__x__D_clean_median_Site_2_inormal
445)	ScanEvents_7_Site_2__x__D_clean_p90_Site_2_inormal
446)	ScanEvents_1_Site_3__x__Eddy_QC_Y_Site_3
447)	ScanEvents_2_Site_3__x__New_Eddy_1_Site_3
448)	ScanEvents_2_Site_3__x__Eddy_QC_Y_Site_3
449)	New_Eddy_1_Site_1__x__Eddy_QC_Y_Site_1
450)	SCALING_T2_FLAIR_1_Site_1__x__Eddy_QC_Y_Site_1
451)	SCALING_SWI_1_Site_1__x__Eddy_QC_Y_Site_1
452)	SCALING_dMRI_1_Site_1__x__Eddy_QC_Y_Site_1
453)	SCALING_rfMRI_1_Site_1__x__SCALING_tfMRI_1_Site_1
454)	S_clean_mean_Site_2__x__D_clean_median_Site_2
455)	S_clean_p90_Site_2__x__D_clean_median_Site_2
456)	D_clean_mean_Site_2__x__D_clean_median_Site_2
457)	D_clean_median_Site_2__x__SD_clean_mean_Site_2
458)	D_clean_median_Site_2__x__SD_clean_p90_Site_2
459)	S_clean_mean_Site_3__x__D_clean_mean_Site_3
460)	S_clean_p90_Site_3__x__D_clean_median_Site_3

461)	D_clean_median_Site_3__x__SD_clean_p90_Site_3
462)	D_clean_median_Site_3__x__D_clean_p90_Site_3_inormal
463)	HeadMotion_p90_tfMRI_abs_Site_2__x__HeadMotionST_p90_inplane_time_Site_2
464)	HeadMotion_p90_tfMRI_abs_Site_2__x__S_clean_median_Site_2_squared
465)	HeadMotion_p90_tfMRI_abs_Site_2__x__S_clean_median_Site_2_inormal
466)	HeadMotion_p90_tfMRI_abs_Site_2__x__S_dirty_p90_Site_2_squared_inormal
467)	HeadMotion_p90_tfMRI_abs_Site_2__x__SD_clean_median_Site_2_squared_inormal
468)	HeadMotion_p90_tfMRI_abs_Site_2__x__TablePos_COG_Z_Site_2_squared_inormal
469)	HeadMotion_mean_dMRI_abs_Site_3__x__Eddy_QC_Y_Site_3
470)	HeadMotion_median_dMRI_abs_Site_3__x__Eddy_QC_Y_Site_3
471)	TablePos_COG_Z_Site_1__x__Eddy_QC_Y_Site_1
472)	TablePos_Table_Site_1__x__Eddy_QC_Y_Site_1
473)	Eddy_QC_Y_Site_3__x__Eddy_QC_Y_Site_3_squared
474)	HeadSize_Site_3_squared__x__TablePos_COG_Z_Site_3_squared
475)	HeadSize_Site_3_inormal__x__TablePos_COG_Z_Site_3_inormal
476)	S_dirty_p90_Site_2_squared__x__D_clean_median_Site_2_inormal
477)	S_dirty_p90_Site_2_squared__x__D_clean_p90_Site_2_inormal
478)	SD_dirty_p90_Site_2_squared__x__D_clean_p90_Site_2_inormal
479)	D_clean_median_Site_2_inormal__x__D_clean_p90_Site_2_inormal
480)	ACQT_Site_1__01
481)	ACQT_Site_1__02
482)	ACQT_Site_1__03
483)	ACQT_Site_1__04
484)	ACQT_Site_1__05
485)	ACQT_Site_1__06
486)	ACQT_Site_1__07
487)	ACQT_Site_1__08
488)	ACQT_Site_1__09
489)	ACQT_Site_1__10
490)	ACQT_Site_1__11
491)	ACQT_Site_1__12
492)	ACQT_Site_1__13
493)	ACQT_Site_1__14
494)	ACQT_Site_1__15
495)	ACQT_Site_1__16
496)	ACQT_Site_1__17
497)	ACQT_Site_1__18
498)	ACQT_Site_1__19
499)	ACQT_Site_2__01
500)	ACQT_Site_2__02
501)	ACQT_Site_2__03
502)	ACQT_Site_2__04

503)	ACQT_Site_2__05
504)	ACQT_Site_2__06
505)	ACQT_Site_2__07
506)	ACQT_Site_2__08
507)	ACQT_Site_2__09
508)	ACQT_Site_2__10
509)	ACQT_Site_2__11
510)	ACQT_Site_2__12
511)	ACQT_Site_2__13
512)	ACQT_Site_2__14
513)	ACQT_Site_2__15
514)	ACQT_Site_2__16
515)	ACQT_Site_2__17
516)	ACQT_Site_2__18
517)	ACQT_Site_2__19
518)	ACQT_Site_2__20
519)	ACQT_Site_3__01
520)	ACQT_Site_3__02
521)	ACQT_Site_3__03
522)	ACQT_Site_3__04
523)	ACQT_Site_3__05
524)	ACQT_Site_3__06
525)	ACQT_Site_3__07
526)	ACQT_Site_3__08
527)	ACQT_Site_3__09
528)	ACQT_Site_3__10
529)	ACQT_Site_3__11
530)	ACQT_Site_3__12
531)	ACQT_Site_3__13
532)	ACQT_Site_3__14
533)	ACQT_Site_3__15
534)	ACQT_Site_3__16
535)	ACQT_Site_3__17
536)	ACQT_Site_3__18
537)	ACQT_Site_3__19
538)	ACQT_Site_3__20
539)	ACQT_Site_3__21
540)	DATE_Site_1__01
541)	DATE_Site_1__02
542)	DATE_Site_1__03
543)	DATE_Site_1__04
544)	DATE_Site_1__05

545)	DATE_Site_1__06
546)	DATE_Site_1__07
547)	DATE_Site_1__08
548)	DATE_Site_1__09
549)	DATE_Site_1__10
550)	DATE_Site_1__11
551)	DATE_Site_1__12
552)	DATE_Site_1__13
553)	DATE_Site_1__14
554)	DATE_Site_1__15
555)	DATE_Site_1__16
556)	DATE_Site_1__17
557)	DATE_Site_1__18
558)	DATE_Site_1__19
559)	DATE_Site_1__20
560)	DATE_Site_1__21
561)	DATE_Site_1__22
562)	DATE_Site_1__23
563)	DATE_Site_1__24
564)	DATE_Site_1__25
565)	DATE_Site_1__26
566)	DATE_Site_1__27
567)	DATE_Site_1__28
568)	DATE_Site_1__29
569)	DATE_Site_1__30
570)	DATE_Site_1__31
571)	DATE_Site_1__32
572)	DATE_Site_1__33
573)	DATE_Site_1__34
574)	DATE_Site_1__35
575)	DATE_Site_1__36
576)	DATE_Site_1__37
577)	DATE_Site_1__38
578)	DATE_Site_1__39
579)	DATE_Site_2__01
580)	DATE_Site_2__02
581)	DATE_Site_2__03
582)	DATE_Site_2__04
583)	DATE_Site_2__05
584)	DATE_Site_2__06
585)	DATE_Site_2__07
586)	DATE_Site_2__08

587)	DATE_Site_2__09
588)	DATE_Site_2__10
589)	DATE_Site_2__11
590)	DATE_Site_2__12
591)	DATE_Site_2__13
592)	DATE_Site_2__14
593)	DATE_Site_2__15
594)	DATE_Site_2__16
595)	DATE_Site_2__17
596)	DATE_Site_2__18
597)	DATE_Site_2__19
598)	DATE_Site_2__20
599)	DATE_Site_2__21
600)	DATE_Site_3__01
601)	DATE_Site_3__02
602)	DATE_Site_3__03
603)	DATE_Site_3__04
604)	DATE_Site_3__05
605)	DATE_Site_3__06
606)	DATE_Site_3__07
607)	DATE_Site_3__08
608)	DATE_Site_3__09
609)	DATE_Site_3__10
610)	DATE_Site_3__11
611)	DATE_Site_3__12
612)	DATE_Site_3__13
613)	DATE_Site_3__14

STable 4a: Demographic characteristics (missing vs non missing cannabis data)

**Variables (at time of imaging)**	**Missing cannabis data (n = 18,056)**	**Non missing cannabis data (n = 15,896)**	**Statistics**	
	**Mean (SD) or % (n)**		***t or χ^{2*}***	***p-value***
Age at 1st scan (years)	64.02 (7.78)	63.69 (7.56)	3.34	1.69e-03
Sex (male % (n))	48.90% (8,829)	45.89% (7,295)	112.89*	6.66e−27
Townsend deprivation index	-1.85 (2.74)	-2.01 (2.65)	4.10	4.12e-05
Employment status (current; % (n))	38.89% (7,022)	38.79% (6,166)	0.065*	4.80e−01
College degree (% (n))	47.10% (8,504)	50.71% (8,065)	29.348*	6.22e−08
BMI (kg/m2)	26.63 (4.20)	26.33 (4.33)	6.30	3.01e-10
Diastolic BP (mmHg)	78.96 (10.61)	78.66 (10.65)	3.39	6.9e-04
Systolic BP (mmHg)	141.42 (19.96)	140.31 (19.79)	6.54	6.54e-11
Alcohol use (current; % (n))	92.91% (16,775)	94.09% (14,955)	5.05*	2.45e−02
Smoking (current; % (n))	3.58% (647)	3.16% (506)	3.275	7.04e−02
Nerves, anxiety, tension, or depression status (% (n))	8.94% (1,614)	8.72% (1,383)	0.308	5.79e−01

Abbreviations: SD, standard deviation; BMI, body mass index; BP, blood pressure

STable 4b: Demographic characteristics

Variables (at time of imaging)	Cannabis users (n = 3,641)	Controls (n = 12,255)	Statistics	
	Mean (SD) or % (n)		*t or χ^{2*}*	*p-value*
Age at 1st scan (years)	61.00 (7.07)	64.49 (7.51)	24.94	2.20e-16
Sex (male % (n))	52.40% (1,908)	43.96% (5,387)	80.29*	2.20e-16
Townsend deprivation index	-1.23 (2.99)	-2.24 (2.50)	-20.31	2.20e-16
Employment status (current; % (n))	52.89% (1,962)	34.30% (4,204)	519.4*	2.20e-16
College degree (% (n))	65.42% (2,382)	46.37% (5,683)	477.89*	2.20e-16
BMI (kg/m^2)	26.21 (4.23)	26.37 (4.36)	1.97	0.05
Diastolic BP (mmHg)	78.54 (10.46)	78.69 (10.70)	0.76	0.45
Systolic BP (mmHg)	137.36 (19.18)	141.19 (19.88)	10.30	2.20e-16
Alcohol use (current; % (n))	96.26% (3,505)	93.43% (11,450)	93.47*	2.20e-16
Smoking (current; % (n))	7.39% (269)	1.93% (337)	1320.10*	2.20e-16
Nerves, anxiety, tension, or depression status (% (n))	11.40% (415)	7.90% (968)	42.83*	5.98e-11

Abbreviations: SD, standard deviation; BMI, body mass index; BP, blood pressure

STable 4c: Demographic characteristics (male and female)

**Variables (at time of imaging)**	**Male Cannabis users (n = 1,908)**	**Male Controls (n = 5,387)**	**Female Cannabis users (n = 1,733)**	**Female Controls (n = 6,868)**
	**Mean (SD) or % (n)**		**Mean (SD) or % (n)**	
Age at 1st scan (years)	61.44 (7.29)	65.61 (7.52)	60.52 (6.79)	63.62 (7.39)
Townsend deprivation index	-1.33 (2.98)	-2.27 (2.49)	-1.12 (2.99)	-2.21 (2.50)
Employment status (current; % (n))	53.93% (1,029)	32.67% (1,760)	53.84% (933)	35.59% (2,444)
College degree (% (n))	62.84% (1,199)	47.24% (2,545)	68.26% (1,183)	45.69% (3138)
BMI (kg/m2)	26.78 (3.89)	26.83 (3.82)	25.57 (4.49)	26.01 (4.71)
Diastolic BP (mmHg)	80.48 (10.03)	80.62 (10.54)	76.41 (10.52)	77.19 (10.59)
Systolic BP (mmHg)	140.99 (18.46)	144.34 (18.45)	133.36 (19.17)	138.72 (20.60)
Alcohol use (current; % (n))	96.59% (1,843)	95.01% (5,118)	95.90% (1,662)	92.20% (6,332)
Smoking (current; % (n))	8.07% (154)	2.32% (125)	6.64% (115)	1.63% (112)
Nerves, anxiety, tension, or depression status (% (n))	10.90% (208)	7.02% (378)	11.94% (207)	8.59% (590)

Abbreviations: SD, standard deviation; BMI, body mass index; BP, blood pressure

STable 5: Association between cannabis use and hippocampus volume

	All/Lifetime cannabis use vs. controls			
	**Beta**	**LCI**	**UCI**	**P-value uncorrected**
Hippocampus Left	0.026	-0.013	0.062	0.183
Hippocampus Right	0.010	-0.028	0.045	0.607

Estimates were generated using multiple linear regression models adjusted for: age, sex, Townsend deprivation index, employment status, educational qualifications, alcohol drinking status, smoking status, body mass index, systolic and diastolic blood pressure, assessment center, nerves/anxiety/tension/depression status and brain imaging confounds including assessment centre, intracranial volume, head motion, table position, and scanner acquisition parameters (site, scanner software, protocol, scan ramp, head coil).
Abbreviations: LCI, lower confidence interval; UCI, upper confidence interval

STable 6: Associations between cannabis use and individual image-derived phenotypes

Image-derived phenotype	estimate	std.error	statistic	p.value
aseg_rh_volume_Inf-Lat-Vent	0.064	0.019	3.481	5.009E-04
aparc-Desikan_lh_area_frontalpole	0.061	0.018	3.332	8.639E-04
aparc-a2009s_lh_thickness_G-cingul-Post-ventral	0.073	0.021	3.472	5.175E-04
aseg_rh_intensity_Pallidum	0.073	0.021	3.509	4.503E-04
IDP_dMRI_TBSS_FA_Genu_of_corpus_callosum	-0.098	0.019	-5.129	2.946E-07
IDP_dMRI_TBSS_FA_Body_of_corpus_callosum	-0.079	0.020	-3.976	7.048E-05
IDP_dMRI_TBSS_MD_Genu_of_corpus_callosum	0.080	0.018	4.442	8.989E-06
IDP_dMRI_TBSS_MD_Cingulum_cingulate_gyrus_L	0.062	0.018	3.443	5.778E-04
IDP_dMRI_TBSS_L2_Genu_of_corpus_callosum	0.086	0.019	4.549	5.426E-06
IDP_dMRI_TBSS_L2_Body_of_corpus_callosum	0.073	0.019	3.770	1.641E-04
IDP_dMRI_TBSS_L3_Genu_of_corpus_callosum	0.102	0.019	5.484	4.229E-08
IDP_dMRI_TBSS_L3_Body_of_corpus_callosum	0.079	0.019	4.051	5.131E-05
IDP_dMRI_TBSS_L3_Anterior_corona_radiata_R	0.066	0.018	3.595	3.250E-04
IDP_dMRI_TBSS_L3_Anterior_corona_radiata_L	0.064	0.018	3.532	4.141E-04
IDP_dMRI_ProbtrackX_L2_cgc_l	0.062	0.019	3.323	8.942E-04
IDP_dMRI_TBSS_ICVF_Genu_of_corpus_callosum	-0.089	0.020	-4.418	1.002E-05
IDP_tfMRI_90th-percentile_BOLD_faces-shapes	0.068	0.020	3.410	6.519E-04
rfMRI connectivity (ICA25 edge 21)	0.100	0.021	4.787	1.711E-06
rfMRI connectivity (ICA25 edge 73)	0.079	0.021	3.837	1.252E-04
rfMRI connectivity (ICA25 edge 133)	0.075	0.020	3.665	2.485E-04
rfMRI connectivity (ICA25 edge 172)	-0.087	0.020	-4.290	1.795E-05
rfMRI connectivity (ICA25 edge 178)	-0.077	0.021	-3.705	2.123E-04
rfMRI connectivity (ICA25 edge 179)	-0.091	0.021	-4.284	1.849E-05
rfMRI connectivity (ICA25 edge 191)	0.077	0.021	3.619	2.971E-04
rfMRI connectivity (ICA100 edge 55)	-0.074	0.021	-3.500	4.669E-04
rfMRI connectivity (ICA100 edge 84)	0.073	0.021	3.406	6.617E-04
rfMRI connectivity (ICA100 edge 132)	0.084	0.021	3.977	7.019E-05
rfMRI connectivity (ICA100 edge 180)	-0.076	0.021	-3.586	3.370E-04
rfMRI connectivity (ICA100 edge 189)	-0.087	0.021	-4.111	3.962E-05
rfMRI connectivity (ICA100 edge 201)	0.089	0.021	4.232	2.331E-05
rfMRI connectivity (ICA100 edge 208)	0.080	0.021	3.787	1.528E-04
rfMRI connectivity (ICA100 edge 322)	0.078	0.021	3.667	2.463E-04
rfMRI connectivity (ICA100 edge 336)	-0.076	0.021	-3.646	2.669E-04
rfMRI connectivity (ICA100 edge 408)	-0.075	0.020	-3.661	2.518E-04
rfMRI connectivity (ICA100 edge 465)	-0.078	0.021	-3.672	2.411E-04
rfMRI connectivity (ICA100 edge 606)	-0.069	0.021	-3.311	9.333E-04
rfMRI connectivity (ICA100 edge 642)	0.077	0.021	3.667	2.461E-04
rfMRI connectivity (ICA100 edge 827)	-0.071	0.021	-3.341	8.382E-04

rfMRI connectivity (ICA100 edge 957)	-0.074	0.021	-3.483	4.973E-04
rfMRI connectivity (ICA100 edge 1177)	0.073	0.021	3.413	6.453E-04

Estimates represent beta coefficients from multiple linear regression models surviving FDR correction and adjusted for: age, sex, Townsend deprivation index, employment status, educational qualifications, alcohol drinking status, smoking status, body mass index, systolic and diastolic blood pressure, assessment centre, nerves/anxiety/tension/depression status and brain imaging confounds including assessment centre, intracranial volume, head motion, table position, and scanner acquisition parameters (site, scanner software, protocol, scan ramp, head coil).

STable 7: ID, location, and network of resting-state functional connectivity association with cannabis use

ID	Edge	PC	Beta	Absolute Difference	Location	Network
Net25 Pair6_7	Edge 21	-0.09	0.100	weaker	(Inferior_frontal;Middle_frontal)<=>(Precuneus;Middle_occipital)	(Default_mode;Central_executive)<=>(Default_mode;Central_executive)
Net25 Pair7_13	Edge 73	-0.58	0.079	weaker	(Precuneus;Middle_occipital)<=>(Supp_motor_area;Middle_temporal;Inferior_frontal)	(Default_mode;Central_executive)<=>(Default_mode;Salience)
Net25 Pair13_17	Edge 133	-0.6	0.075	weaker	(Supp_motor_area;Middle_temporal;Inferior_frontal)<=>(Superior_temporal;Middle_temporal)	(Default_mode;Salience)<=>(Default_mode;Motor)
Net25 Pair1_20	Edge 172	-0.1	-0.087	stronger	(Cerebellum;Precuneus;Superior_frontal)<=>(Precuneus)	(Default_mode)<=>(Default_mode;Central_executive)
Net25 Pair7_20	Edge 178	1.96	-0.077	weaker	(Precuneus;Middle_occipital)<=>(Precuneus)	(Default_mode;Central_executive)<=>(Default_mode;Central_executive)
Net25 Pair8_20	Edge 179	-0.45	-0.091	stronger	(Lingual;Calcarine;Superior_occipital)<=>(Precuneus)	(Visual)<=>(Default_mode;Central_executive)
Net25 Pair1_21	Edge 191	-0.27	0.077	weaker	(Cerebellum;Precuneus;Superior_frontal)<=>(Inferior_frontal;Superior_frontal;Middle_temporal)	(Default_mode)<=>(Default_mode;Central_executive)
Net100 Pair10_11	Edge 55	0.93	-0.074	weaker	(Middle_occipital;Precuneus; Calcarine)<=>(Angular;Middle_temporal)	(Default_mode;Central_executive)<=>(Default_mode;Central_executive)
Net100 Pair6_14	Edge 84	-0.12	0.073	weaker	(Postcentral;Precentral)<=>(Cuneus;Superior_occipital)	(Motor)<=>(Visual)
Net100 Pair12_17	Edge 132	-0.8	0.084	weaker	(Middle_frontal;Cerebellum)<=>(Cerebellum)	(Central_executive;Default_mode)<=>(Subcortical-cerebellum)
Net100 Pair9_20	Edge 180	0.38	-0.076	weaker	(Middle_temporal;Temporal_pole)<=>(Postcentral;Precentral)	(Default_mode)<=>(Motor;Attention)
Net100 Pair18_20	Edge 189	1.42	-0.087	weaker	(Inferior_parietal;Postcentral)<=>(Postcentral;Precentral)	(Attention;Central_executive;Salience)<=>(Motor;Attention)
Net100 Pair11_21	Edge 201	1.21	0.089	stronger	(Angular;Middle_temporal)<=>(Superior_frontal)	(Default_mode;Central_executive)<=>(Default_mode;Central_executive)
Net100 Pair18_21	Edge 208	-0.45	0.080	weaker	(Inferior_parietal;Postcentral)<=>(Superior_frontal)	(Attention;Central_executive;Salience)<=>(Default_mode;Central_executive)
Net100 Pair22_26	Edge 322	-0.59	0.078	weaker	(Superior_temporal)<=>(Inferior_parietal;Inferior_frontal;SupraMarginal)	(Default_mode;Motor)<=>(Attention;Central_executive)
Net100 Pair11_27	Edge 336	-0.45	-0.076	stronger	(Angular;Middle_temporal)<=>(Precentral;Superior_frontal; Supp_motor_area)	(Default_mode;Central_executive)<=>(Attention;Salience;Motor)
Net100 Pair2_30	Edge 408	0.23	-0.075	weaker	(Rolandic_operculum;SupraMarginal;Insula)<=>(Paracentral)	(Salience;Motor)<=>(Motor)
Net100 Pair30_31	Edge 465	-0.15	-0.078	stronger	(Paracentral)<=>(Middel_frontal)	(Motor)<=>(Central_executive)
Net100 Pair11_36	Edge 606	1.68	-0.069	weaker	(Angular;Middle_temporal)<=>(Precuneus)	(Default_mode;Central_executive)<=>(Default_mode;Central_executive)

Net100 Pair12_37	Edge 642	0.6	0.077	stronger	(Middle_frontal;Cerebellum)<=>(Middle_frontal)	(Central_executive;Default_mode)<=>(Central_executive;Salience)
Net100 Pair7_42	Edge 827	0.05	-0.071	weaker	(Superior_frontal;Anterior_cingulate)<=>(Inferior_temporal;Inferior_occipital)	(Default_mode;Limbic)<=>(Attention;Visual)
Net100 Pair11_45	Edge 957	0.57	-0.074	weaker	(Angular;Middle_temporal)<=>(Superior_frontal;Middle_frontal)	(Default_mode;Central_executive)<=>(Salience;Default_mode)
Net100 Pair1_50	Edge 1177	-0.26	0.073	weaker	(Calcarine;Lingual;Cuneus)<=>(Orbital)	(Visual)<=>(Limbic)

STable 8: Associations between cannabis use and brain measures surviving Bonferroni correction

Brain measures	Beta	LCI	UCI	P-value
FA Genu of corpus callosum	-0.10	-0.13	-0.06	2.95e-07
MD Genu of corpus callosum	0.08	0.04	0.11	8.99e-06
L2 Genu of corpus callosum	0.09	0.05	0.12	5.43e-06
L3 Genu of corpus callosum	0.10	0.06	0.14	4.23e-08
ICVF Genu of corpus callosum	-0.09	-0.13	-0.05	1.00e-05
rfMRI connectivity (ICA25 edge 21)	0.10	0.06	0.14	1.71e-06

Estimates represent beta coefficients from multiple linear regression models adjusted for: age, sex, Townsend deprivation index, employment status, educational qualifications, alcohol drinking status, smoking status, body mass index, systolic and diastolic blood pressure, assessment centre, nerves/anxiety/tension/depression status and brain imaging confounds including assessment centre, intracranial volume, head motion, table position, and scanner acquisition parameters (site, scanner software, protocol, scan ramp, head coil).
Abbreviations: LCI, lower confidence interval; UCI, upper confidence interval; FA, fractional anisotropy; MD, mean diffusivity; L2 and L3, radial diffusivities; ICVF intracellular volume fraction; rfMRI, resting-state functional magnetic resonance imaging.

STable 9: Associations between cannabis use and brain measures in males surviving False Discovery Rate

Brain measures	Beta	SE	statistic	P-value
MO Fornix cres+Stria terminalis (Right)	0.101	0.028	3.574	3.543E-04
rfMRI connectivity (ICA25 edge 21)	0.110	0.030	3.625	2.907E-04
rfMRI connectivity (ICA25 edge 133)	0.112	0.029	3.834	1.272E-04
rfMRI connectivity (ICA25 edge 172)	-0.108	0.029	-3.656	2.582E-04
rfMRI connectivity (ICA100 edge 201)	0.111	0.030	3.649	2.655E-04
rfMRI connectivity (ICA100 edge 957)	-0.113	0.030	-3.700	2.171E-04

Estimates represent beta coefficients from multiple linear regression models adjusted for: age, Townsend deprivation index, employment status, educational qualifications, alcohol drinking status, smoking status, body mass index, systolic and diastolic blood pressure, assessment centre, nerves/anxiety/tension/depression status and brain imaging confounds including assessment centre, intracranial volume, head motion, table position, and scanner acquisition parameters (site, scanner software, protocol, scan ramp, head coil).
Abbreviations: SE, standard error; MO, diffusion tensor mode; rfMRI, resting-state functional magnetic resonance imaging.

STable 10: Associations between cannabis use and brain measures in females surviving False Discovery Rate

Brain measures	Beta	SE	statistic	P-value
FA Genu of corpus callosum*	-0.131	0.028	-4.730	2.284E-06
FA Body of corpus callosum	-0.109	0.029	-3.786	1.542E-04
MD Genu of corpus callosum	0.100	0.026	3.799	1.466E-04
MD Anterior corona radiata (Right)	0.092	0.026	3.528	4.211E-04
L2 Genu of corpus callosum	0.116	0.027	4.241	2.249E-05
L2 Body of corpus callosum	0.103	0.028	3.689	2.266E-04
L3 Genu of corpus callosum*	0.129	0.027	4.798	1.630E-06
L3 Body of corpus callosum	0.112	0.028	4.003	6.309E-05
L3 Anterior limb of internal capsule (Right)	0.107	0.026	4.164	3.154E-05
L3 Anterior corona radiata (Right)	0.105	0.027	3.913	9.173E-05
L3 Anterior corona radiata (Left)	0.098	0.026	3.723	1.984E-04
L3 Superior corona radiata (Right)	0.099	0.026	3.812	1.391E-04
L3 Superior corona radiata (Left)	0.093	0.026	3.555	3.797E-04
MD Inferior fronto-occipital fasciculus (Right)	0.101	0.027	3.794	1.491E-04
MD Posterior thalamic radiation (Right)	0.091	0.025	3.592	3.300E-04
L2 Inferior fronto-occipital fasciculus (Right)	0.099	0.028	3.599	3.209E-04
L2 Inferior longitudinal fasciculus (Left)	0.100	0.027	3.651	2.627E-04
L2 Inferior longitudinal fasciculus (Right)	0.096	0.027	3.545	3.946E-04
L2 Posterior thalamic radiation (Right)	0.092	0.026	3.504	4.602E-04
L3 Inferior fronto-occipital fasciculus (Right)	0.109	0.028	3.885	1.032E-04
L3 Posterior thalamic radiation (Right)	0.099	0.028	3.541	4.016E-04
ICVF Genu of corpus callosum	-0.109	0.029	-3.782	1.566E-04
rfMRI connectivity (ICA100 edge 694)	0.107	0.030	3.526	4.248E-04
rfMRI connectivity (ICA100 edge 1010)	-0.113	0.031	-3.694	2.224E-04

Estimates represent beta coefficients from multiple linear regression models adjusted for: age, Townsend deprivation index, employment status, educational qualifications, alcohol drinking status, smoking status, body mass index, systolic and diastolic blood pressure, assessment centre, nerves/anxiety/tension/depression status and brain imaging confounds including assessment centre, intracranial volume, head motion, table position, and scanner acquisition parameters (site, scanner software, protocol, scan ramp, head coil).
*Brain measures after surviving Bonferroni correction
Abbreviations: SE, standard error; FA, fractional anisotropy; MD, mean diffusivity; L2 and L3, radial diffusivities; ICVF intracellular volume fraction; rfMRI, resting-state functional magnetic resonance imaging.

STable 11a: Two-sample linear MR estimates for the causal effect of cannabis use on brain IDPs

Brain IDPs	SNPs	MR Tests	Estimates	SE	*P*-value
FA Genu of Corpus callosum	22*	MR Egger	-0.171	0.153	0.278
		Weighted mean	-0.001	0.055	0.986
		IVW	0.010	0.053	0.849
		Simple mode	-0.062	0.104	0.559
		Weighted mode	-0.094	0.092	0.318
	8**	MR Egger	-0.073	0.134	0.608
		Weighted mean	-0.066	0.065	0.309
		IVW	-0.048	0.049	0.329
		Simple mode	-0.072	0.094	0.469
		Weighted mode	-0.068	0.083	0.442
FA Body of Corpus callosum	22*	MR Egger	-0.042	0.124	0.735
		Weighted mean	0.031	0.051	0.539
		IVW	0.040	0.042	0.333
		Simple mode	-0.011	0.099	0.909
		Weighted mode	-0.019	0.088	0.831
	8**	MR Egger	-0.025	0.134	0.856
		Weighted mean	0.008	0.063	0.898
		IVW	0.012	0.049	0.804
		Simple mode	0.026	0.087	0.772
		Weighted mode	0.014	0.079	0.865
MD Cingulum cingulate gyrus (Left)	22*	MR Egger	0.188	0.110	0.101
		Weighted mean	0.046	0.050	0.360
		IVW	0.032	0.039	0.405
		Simple mode	0.184	0.104	0.093
		Weighted mode	0.173	0.087	0.061
	8**	MR Egger	0.183	0.134	0.220
		Weighted mean	-0.048	0.067	0.469
		IVW	-0.038	0.049	0.447
		Simple mode	-0.080	0.103	0.464
		Weighted mode	-0.070	0.096	0.492
L3 Anterior corona radiate (Right)	22*	MR Egger	0.160	0.124	0.212
		Weighted mean	-0.066	0.053	0.210
		IVW	-0.022	0.044	0.610
		Simple mode	-0.088	0.091	0.349
		Weighted mode	-0.080	0.098	0.426
	8**	MR Egger	0.181	0.165	0.315
		Weighted mean	0.099	0.069	0.148
		IVW	0.061	0.059	0.303
		Simple mode	0.175	0.127	0.211
		Weighted mode	0.164	0.103	0.155
L3 Anterior corona radiate (Left)	22*	MR Egger	0.170	0.122	0.178
		Weighted mean	-0.001	0.050	0.986
		IVW	0.025	0.042	0.559
		Simple mode	-0.014	0.094	0.883
		Weighted mode	-0.007	0.088	0.942
	8**	MR Egger	0.228	0.134	0.140
		Weighted mean	0.097	0.069	0.158
		IVW	0.044	0.049	0.369
		Simple mode	0.137	0.118	0.285
		Weighted mode	0.144	0.105	0.214
rfMRI connectivity (ICA25 edge 21)	22*	MR Egger	-0.114	0.145	0.441
		Weighted mean	-0.003	0.052	0.949

		IVW	0.025	0.049	0.606
		Simple mode	-0.019	0.094	0.839
		Weighted mode	-0.011	0.076	0.890
	8**	MR Egger	0.101	0.134	0.480
		Weighted mean	0.043	0.064	0.500
		IVW	0.025	0.049	0.607
		Simple mode	0.045	0.087	0.619
		Weighted mode	0.045	0.076	0.568
rfMRI connectivity (ICA100 edge 55)	22*	MR Egger	-0.095	0.100	0.354
		Weighted mean	-0.03	0.048	0.529
		IVW	-0.038	0.034	0.260
		Simple mode	-0.133	0.094	0.173
		Weighted mode	-0.128	0.091	0.172
	8**	MR Egger	0.054	0.134	0.702
		Weighted mean	0.007	0.063	0.914
		IVW	0.015	0.049	0.759
		Simple mode	-0.054	0.095	0.584
		Weighted mode	-0.056	0.088	0.541

* SNPs associated with *cannabis dependence and abuse*
** SNPs associated *with lifetime cannabis use*
Abbreviations: IDP, imaging-derived phenotype; SNP, single nucleotide polymorphism; SE, standard error; IVW, inverse-variance weighted.

STable 11b: Two-sample linear Reverse MR estimates for the causal effect of brain IDPs on cannabis use

Cannabis use	SNPs	Estimates	SE	*P*-value
Cannabis dependence or abuse	1*	-283[a]	266	0.915
	2**	-988[b]	261	0.706
	2***	-767[b]	956	0.423
Lifetime cannabis use	1*	-1837.928[a]	885.547	0.038
	1*	20.273[a]	40.283	0.615

[a] Estimates are from Wald ration
[b] Estimates are from Inverse-variance weighted (IVW) analysis
* SNPs associated with FA genu of corpus callosum
** SNPs associated with rfMRI connectivity (ICA25 edge 21)
*** SNPs associated with rfMRI connectivity (ICA100 edge 55)
Abbreviations: IDP, imaging-derived phenotype; SNP, single nucleotide polymorphism; SE, standard error.
